# Genome-wide analysis shows that RNase G plays a global role in the stability of mRNAs in *Stenotrophomonas maltophilia*

**DOI:** 10.1038/s41598-017-16091-0

**Published:** 2017-11-22

**Authors:** Alejandra Bernardini, José L. Martínez

**Affiliations:** Centro Nacional de Biotecnología, CSIC, Darwin 3, 28049, Madrid, Spain

## Abstract

Gene expression is determined by critical processes such as RNA synthesis and degradation. Ribonucleases participate in the coordinated and differential decay of messenger RNAs. We describe a suitable method of normalization and calculation of mRNAs half-life values quantified by RNA-Seq. We determined the mRNA half-lives of more than 2000 genes in *Stenotrophomonas maltophilia* D457 and in an isogenic RNase G deficient mutant. Median half-lives were 2,74 and 3 min in the wild-type and the *rng*-deficient strain, respectively. The absence of RNase G resulted in an overall enhancement of mRNA half-life times, showing that many RNAs are targets of RNase G in *S. maltophilia*. Around 40 genes are likely to be regulated directly by RNase G since their half-lives were more than two-fold higher in the *rng*-deficient mutant. Gene length, GC content or expression levels did not correlate with mRNAs lifetimes, although groups of genes with different functions showed different RNA half-lives. Further, we predicted 1542 gene pairs to be part of the same operons in *S. maltophilia*. In contrast to what was described for other bacteria, our data indicate that RNase G has a global role in mRNA stability and consequently in the regulation of *S. maltophilia* gene expression.

## Introduction

The expression of a gene is a dynamic process that is regulated at several levels (i.e. the transcription initiation, RNA processing and degradation, translation, and the processing and degradation of the protein product). Alteration in the rate of messenger decay is thus an important factor in the regulation of a gene’s steady-state mRNA expression level^1,2^, hence allowing bacterial adaptation to changing environmental conditions. The degradation of any mRNA may be influenced by various factors including its secondary structure and its rate of translation. These parameters can be affected by RNA-binding proteins and by regulatory RNAs. Therefore, it was suggested that a basal decay rate is mainly dictated by the mRNA molecule itself^3–6^. In addition, different RNases can degrade specific mRNAs, hence allowing a more functional-based regulation of RNA decay. The molecular machinery of mRNA turnover differs between pathogenic bacteria and their eukaryote hosts, suggesting that these differences could be used to define novel targets for the development of new antimicrobial drugs^7^.

*Escherichia. coli* is the best studied model microorganism for analysing mRNA decay. Many enzymes, including those forming part of the RNA degradosome (a multisubunit protein complex that functions as endoribonuclease and 3´-exoribonuclease) participate in the degradation of mRNA in *E. coli* and in Gram-negative bacteria in general^8^. RNase E is an essential endonuclease that forms part of the RNA degradosome^8^ and plays an important role in RNA metabolism in many bacteria^9^. Other independent endoribonucleases contribute to the turnover of mRNAs, as RNase G, RNase I and RNase III^10–12^. *E. coli* RNase G is an endonuclease possessing ∼50% sequence similarity to the RNase E catalytic region and overlapping, but not identical, cleavage specificity^13–15^. RNase G is found in Gram-negative pathogen microorganisms such as *Klebsiella pneumoniae*, *Acinetobacter baumannii*, *Pseudomonas aeruginosa*, *Enterobacter cloacae* and *E. coli*, and is not an essential protein^16^. RNase E and RNase G are present in *Stenotrophomonas maltophilia* ^17^. RNases G from *E. coli* and *S. maltophilia* have not significant differences in domains, sequence or length, except for few differences in single amino acids. We have previously shown that the absence of RNase G is associated with increased resistance to quinolones and with the overexpression of genes involved in the *S. maltophilia* heat shock response^18^, indicating that RNase G might be involved in the cellular response to stress.

*S. maltophilia* is an opportunistic pathogen, associated with nosocomial infections, which presents a low level of susceptibility to several antibiotics, including quinolones^19^. The low susceptibility of this pathogen is partly due to the presence in its genome of genes encoding a wide range of resistance determinants^18,20–28^. Although during the past years much knowledge on the genomic structure complexity of *S. maltophilia* was obtained, transcriptomic studies and mRNA degradation studies were not performed in this microorganism.

During the past decade, microarray technology was a useful tool for studies on global mRNA decay in a wide range of different organisms^3,29,30^. However, high throughput RNA sequencing (RNA-Seq) has much higher resolution and accuracy than microarrays. Some studies have performed genome-wide analysis of mRNA decay at single nucleotide resolution using RNA sequencing mostly in *E. coli* and in species of the *Bacillus* genus^5,31–33^. We present here a genome-wide analysis of mRNA decay using RNA-Seq in *S. maltophilia* and provide a suitable method for normalization and calculation of mRNAs half-life values. We report mRNA half-lives of more than 2000 genes in the *S. maltophilia* wild-type clinical strain D457 as well as in its isogenic *rng*-defective mutant ALB001. Further, the comparison of mRNA decay in both strains allowed us to find targets of RNase G and to analyse the correlation of mRNA half-lives with gene characteristics and functions.

## Results

### Data analysis and mapping of sequence reads

**Figure 1:**
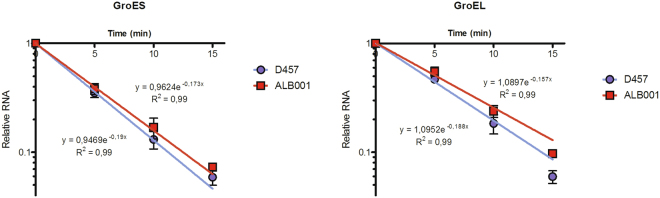
Relative rate of expression of *groES* and *groEL* validate the run-out of mRNA after rifampicin addition in D457 and ALB001 strains. Strains D457 and ALB001 were cultivated in LB medium at 37 °C. At 0.6 OD_600nm_ rifampicin was added to 200 mg/L to inhibit transcription by RNA polymerase. At different time points, aliquots were taken for RNA extraction and the relative amount of *groES* and *groEL* mRNAs was measured by real-time RT-PCR. Values indicate the decrease of the mRNA levels observed as a function of time after rifampicin addition (time 0) and correspond to the average of two independent assays; the standard error is shown.

Mid-log cultures of the *S. maltophilia* wild-type strain (D457) and a RNase G deficient strain (ALB001) were subjected to transcriptional arrest by rifampicin^34^, and RNA was isolated 0, 5, 10 and 15 min after rifampicin addition. The use of rRNA as reference for the RT-qPCR-based mRNA decay experiments was validated by measuring the transcript decay 15 min after rifampicin addition (Figure S1), confirming that 16S rRNA was not detectably degraded. In all cases, the mean values for relative mRNA expression obtained in two independent experiments, each one with two technical replicates were considered. To validate the decrease of mRNA levels as a function of time after rifampicin addition, the expression of *groES* and *groEL* was determined by RT-qPCR (Fig. 1). There was a 17 to 18- and 10 to 14 -fold decrease of *groES* and *groEL* mRNA levels, respectively, after 15 min of rifampicin addition. The mRNA decay of these genes illustrated the efficiency of mRNA run-out after rifampicin addition. For these analysed messengers, the RNA decay fits well with the logarithmic regression model described in Methods (R^2^ = 0,99 in all cases).

Following rRNA depletion, mRNA levels of all genes were determined by RNA sequencing (see Methods). After removing the adapter oligonucleotides with Ampure beads, cDNA samples were deep sequenced using the Illumina technology. Quality analysis of sequenced samples showed that 25% of the reads are over-represented (Table 1). Further refinement of the data was carried out to assure that these over-represented sequences are free from adapter and rRNA sequences. Samples were subjected to sequence trimming and quality filtering (Table 2). Just between 0.2 and 2.6% of over-represented reads were adapters, and consequently, they were removed from the analysis. The rest of abundant sequences could correspond to highly expressed genes as well as to multiple-copy genes, such as those encoding rRNAs and tRNAs. Indeed, just between 0.8 and 2.3% of the reads after discarding the adapters were sequences mapping to multiple positions in the genome, indicating that most of the over-represented sequences corresponded to *S. maltophilia* highly expressed genes. The low number of reads that mapped to multiple positions in the genome and the verification that most of them belong to tRNAs (data not shown) reflected a successful removal of rRNA molecules. The ORFs encoding tRNAs or ncRNA were not included in the analysis. After trimming and filtering procedures, nearly 6 out of more than 12 million reads (46%) and 7 out of 11 million reads (63%) produced by Illumina sequencing from *S. maltophilia* D457 and ALB001 strains, respectively, were unambiguously mapped -matched in a single position- to the *S. maltophilia* D457 annotated genome (Table 2).

**Table 1:** Over-represented reads in RNA deep sequencing raw data.

Strain	Time point (min)	Total number of reads	% over-represented reads	Total number of reads without over-represented reads
D457	0	2.197.512	3,14	2.128.510
	5	3.547.610	28	2.554.279
	10	2.340.126	29	1.661.489
	15	4.385.299	56,5	1.907.605
ALB001	0	2.160.193	5	2.052.183
	5	4.100.701	35	2.665.456
	10	2.776.957	7,8	2.560.354
	15	2.424.976	8,1	2.228.553
Total		23.933.374	25,8	17.758.430

**Table 2:** Trimming and quality filtering of raw data and mapping of reads to the genome.

Strain	Time point (min)	Total number of reads	Number of reads removed	%	Number of reads after filtering	%	Reads that could not be mapped^a^	%	Number of unambigu-ously mapped reads^b^	%	Reads that could not be unambiguously mapped^c^	%
**D457**	**0**	2.197.512	3.907	0,2	2.193.605	99,8	111.934	5,1	**2.036.212**	**92,7**	45.459	2,1
	**5**	3.547.610	57.622	1,6	3.489.988	98,4	1.702.223	48,0	**1.728.206**	**48,7**	59.559	1,7
	**10**	2.340.126	37.029	1,6	2.303.097	98,4	1.200.028	51,3	**1.060.112**	**45,3**	42.957	1,8
	**15**	4.385.299	115.386	2,6	4.269.913	97,4	3.287.232	75,0	**947.223**	**21,6**	35.458	0,8
	**Total**	12.470.547							**5.771.753**	**46,3**		
**ALB001**	**0**	2.160.193	6.042	0,3	2.154.151	99,7	154.031	7,1	**1.949.484**	**90,2**	50.636	2,3
	**5**	4.100.701	71.082	1,7	4.029.619	98,3	2.174.103	53,0	**1.776.612**	**43,3**	78.904	1,9
	**10**	2.776.957	11.994	0,4	2.764.963	99,6	793.794	28,6	**1.918.685**	**69,1**	52.484	1,9
	**15**	2.424.976	11.280	0,5	2.413.696	99,5	787.885	32,5	**1.573.193**	**64,9**	52.618	2,2
	**Total**	11.462.827							**7.217.974**	**63,0**		
**Total**		36.403.921	314.342		23.619.032		10.211.230		**18.761.480**		418.075	

^a^Reads that could not be mapped to the genome.

^b^Reads that could be mapped to a single position in the genome.

^c^Reads that could be mapped to several positions in the genome.

### Normalization of the data

To investigate the progressive decline of mRNA in *S. maltophilia* and the influence of RNase G in such decay, we performed a genome-wide determination of mRNA half-lives in the *S. maltophilia* wild-type D457 strain and the *S. maltophilia* D457 *rng*-insertion mutant ALB001 by RNA-Seq. Since variation in sequencing effectiveness, variable effectiveness in rRNA depletion or multiple sequencing of some samples could lead to sample-to-sample variation, normalization of the data was required. For this, we calculated by RT-qPCR (see Methods for details) the decay rate of *groES*, which presents a high expression level, as estimated by its reads per kilobase per million mapped reads (RPKM) value in mid-exponential phase and we used the *groES* RT-qPCR obtained expression data for normalization. The use of a similar methodology for normalization of RNA-Seq data was reported before^31^. *groES* RNA degradation was similar in D457 and ALB001 strains and there were no significant differences (*p* > 0,05) in the relative amount of RNA at any of the time points (Fig. 1). To test the validity of the normalization procedure, the half-lives of four mRNAs were determined for the D457 strain. When comparing the half-lives of these mRNAs, estimated either by RT-qPCR or by using the information derived from the RNA-Seq analysis, a Pearson correlation coefficient of 0.96 was obtained (Table 3). The reproducibility of the decline rates obtained by using independently both methods indicates that RNA-Seq, together with the normalization procedure of the data before described, is a suitable method for calculating mRNA half-lives in *S. maltophilia*.

**Table 3:** Comparison of mRNA half-lives as estimated by Illumina RNA-Seq. 1.9 and RT-qPCR for 4* S. maltophilia* D457 genes used for validation of normalization method.

Gene	Expression (RPKM)^a^	mRNA Half-life by RNA-Seq (min)	mRNA Half-life by RT-qPCR (min)
*rpoD* (SMD_3765)	658	2,34	2,50
*ftsZ* (SMD_0648)	384	3,58	4,03
*gyrA* (SMD_2717)	488	2,40	2,58
*groEL* (SMD_3813)	1879	3,49	3,67

^a^Expression in mid-exponential phase of *S. maltophilia* D457 culture growing in LB medium^18^.

### Genome-wide determination of mRNA decay rates in *S. maltophilia* D457 and ALB001

**Figure 2:**
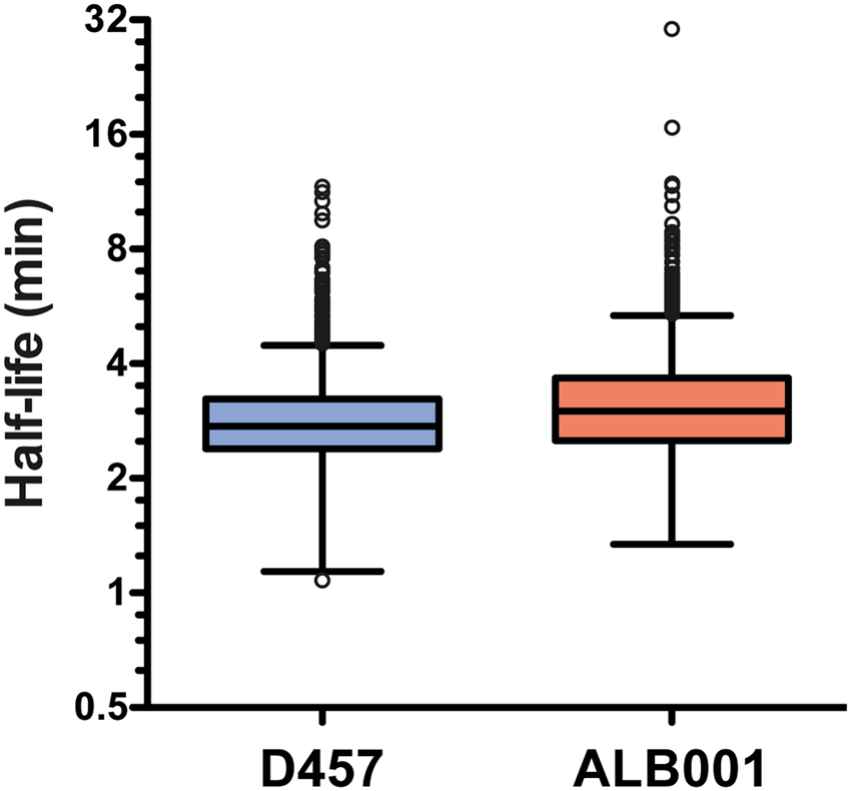
Distribution of global mRNA half-lives for *S. maltophilia* D457 and ALB001 determined by RNA-Seq using Illumina 1.9 technology and depicted by a boxplot at log scale. Each box comprises the 25 to 75 percentile of measured mRNA half-lives for the indicated strain (the 50% of the calculated half-life values). Whisker top bar corresponds to maximum half-life except from outlier values (over Q3 + 1,5(IQR)) and whisker bottom bar corresponds to minimum half-life except from outlier values (under Q1 − 1,5(IQR)). The bar inside each box marks the median mRNA half-life in each of the two bacterial strains. Q1: Quartile 1; Q3: Quartile 3; IQR: Interquartile Range.

Using the above described approach, we could determine the half-lives of 2674 mRNAs in D457, and 2449 in *S. maltophilia* ALB001. The half-lives of 2179 mRNAs were determined in both strains. Table 4 shows an overall review of the mRNA half-lives calculated for both strains, Fig. 2 shows the distribution of the mRNA half-lives calculated, and Table S1 (Supplemental material) shows a list of all mRNA half-lives. Half-lives had median values of 2.74 and 3 min and mean values of 2.88 and 3.24 min in D457 and ALB001 strain respectively (Table 4). In *S. maltophilia* D457, half of the estimated half-life values ranged between 2.39 and 3.23 min, while in the *rng* deficient *S. maltophilia* strain ALB001, half of the estimated half-lives values ranged between 2.51 and 3.67 min (Fig. 2). The shortest half-live observed in D457 was 1.08 min for SMD_3475 that codifies azurin and constitutes the only outlier of the lower limit. The shortest half-life observed in ALB001 was 1.34 min for *lspA*, which encodes the lipoprotein signal peptidase. SMD_3475 mRNA has a much higher half-life in the ALB001 mutant (2.74 min) than in the wild-type strain, indicating that RNase G is likely involved in its processing. In contrast, the *lspA* mRNA has similar half-lives in both strains, suggesting that RNase G has no role in its degradation. It should be noted that RNAs with extremely short half-lives are most likely excluded in the analysis because their fast degradation hampers the adjustment of the data to the logarithmic regression model, and they are discarded in the filtering. In *S. maltophilia* D457, 79 mRNAs showed half-lives longer than 4.49 minutes, the top whisker of the distribution. In ALB001, 86 mRNAs showed half-lives longer than 5.4 minutes, 47 of which are the same as in D457. These genes present the highest mRNA half-lives of all the set and are outliers of the distribution in Fig. 2 (see Table S1 for mRNA half-lives). By determining RNA half-lives of more than 2000 genes we observed that deficiency of RNase G affects normal lifetimes of mRNAs since general parameters are higher and overall distribution of mRNA half-lives is different in the *rng*-deficient mutant regarding to D457 wild-type strain.

**Table 4:** General features of *S. maltophilia* D457 genome and mRNA half-lives for D457 and ALB001 strains.

Chromosome information	*S. maltophilia* D457	
RefSeq	NC_017671.1	
IN SDC	HE798556.1	
Size (Mb)	4,77	
GC%	66,8	
Annotated ORF	4381	
ORF expressing proteins	4220	
rRNAs	13	
tRNAs	73	
Other RNAs	23	
Pseudogenes	52	
**Data analysis information**	**D457**	**ALB001**
Expressed genes (RPKM > 15)	3119	2926
Genes with mRNA half-lives that could not be determined^a^	445	477
Genes with mRNA half-lives determined	2674	2449
Median gene half-life (min)	2,74	3,00
Mean gene half-life (min)	2,88	3,24
Minimum gene half-life (min)	1,08	1,34
Maximum gene half-life (min)	11,67	30,29
Median gene expression level (RPKM)^b^	45	43
Mean gene expression level (RPKM)^b^	203,61	203,31
Minimum gene expression level (RPKM)^b^	1	1
Maximum gene expression level (RPKM)^b^	31718	36483

^a^RNA of genes which adjustment of the expression data did not throw a R^2^ > 0.7 to the logarithmic regression model.

^b^Expression in mid-exponential phase of *S. maltophilia* D457 culture growing in LB medium^18^.

### Operon prediction

Multi-gene operons were predicted from our RNA-Seq data by Rockhopper software. The operons are predicted by the distance of CDS in genome and by their data of expression in all the samples. In our RNA-Seq data from *S. maltophilia*, 1542 gene-pairs were predicted to be part of the same operons and 808 likely belong to multi-genes operons. Table S2 shows a list of genes grouped by their predicted operon, their RNA half-lives and their RPKM at the first time point of the experiment. RNAs of genes present in the same operon have to a certain degree similar half-lives. RNAs of the predicted operons SMD_4075/SMD_4076 and SMD_4077/SMD_4078 have very long half-lives in both strains and similar expression, and RNAs of predicted operon SMD_3354/SMD_3355 have short half-lives and high expression, making very likely that these gene-pairs are part of the same double-gene operon. However other RNAs of genes included in the same predicted operon have different half-lives, which may be due to an unequal degradation and regulation of the messenger molecule. A predicted triple-gene operon contained SMD_3812, *groEL* and *groES* genes. These genes presented similar RNA half-lives, but *groEL* and *groES* expression was much higher than SMD_3812 expression. Interestingly, the RNA half-lives of these genes were more variable in the ALB001 mutant than in the D457 wild-type strain. RNAs of genes included in the same predicted multi-gene operon had more variable half-lives in ALB001 than in D457, suggesting a deficiency in the correct timing of RNA degradation when RNase G is absent.

### Role of gene sequence and expression levels in mRNA half-life

**Figure 3:**
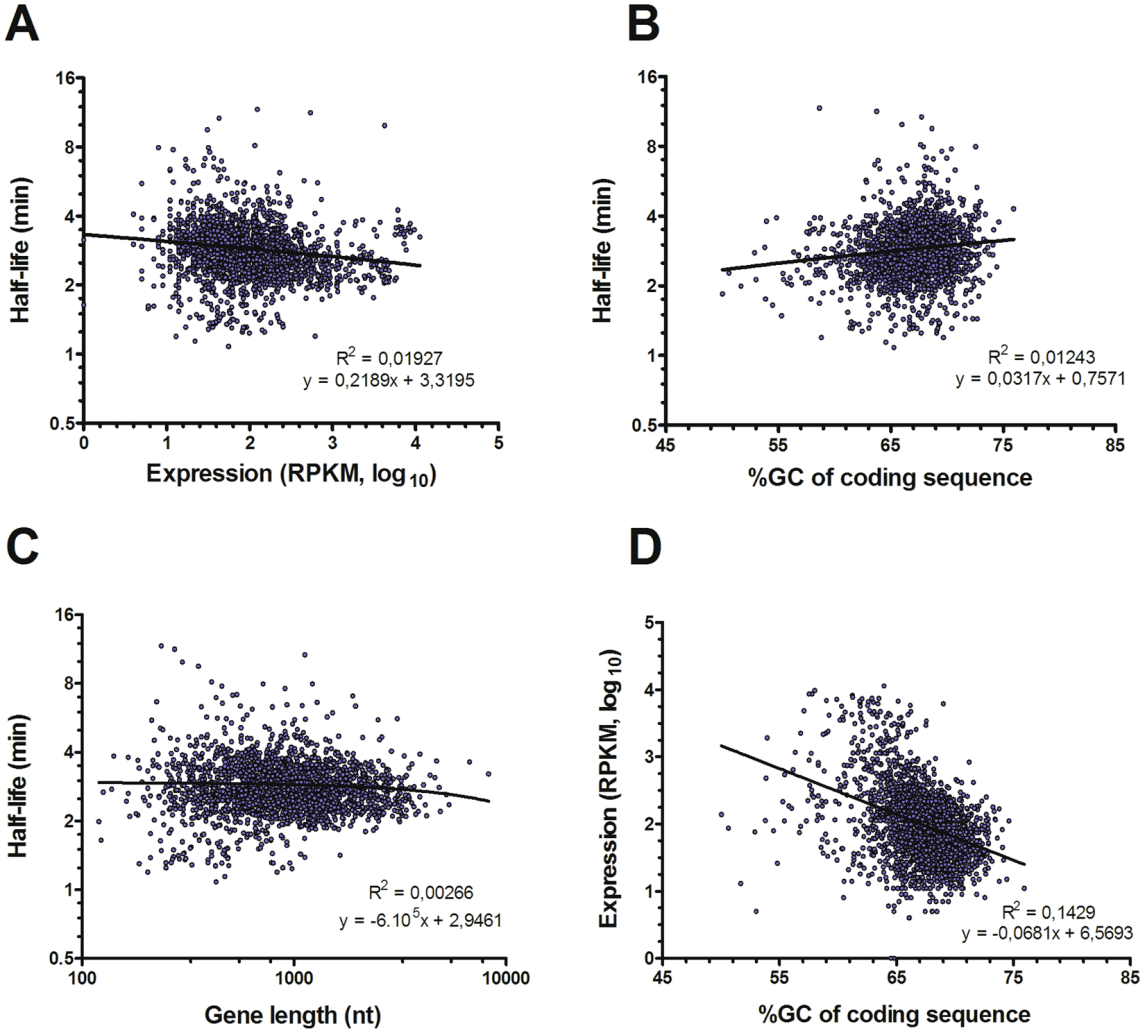
Correlation between mRNA half-life, gene expression and coding sequence characteristics of *S. maltophilia* D457 mRNAs. (**A**) Correlation between mRNA half-lives and gene expression. (**B**) Correlation between mRNA half-lives and G + C percentage of CDSs. (**C**) Correlation between mRNA half-lives and length of CDSs. (**D**) Correlation between gene expression and G + C percentage of CDSs. mRNA half-lives and gene length are plotted at log scale. Pearson coefficient of correlation and equation of the regression line are presented.

We investigated how different parameters of *S. maltophilia* genes may influence mRNA decay rates in *S. maltophilia* D457. We evaluated the influence of the level of expression of the genes in mid-exponential phase (transcriptome accession number SRR2128156^18^), and sequence attributes of the CDS as the gene GC percentage and the length of the ORF (Fig. 3). None of the parameters tested here showed a correlation with the mRNA half-live values, meaning that in *S. maltophilia* the rate of decay of a mRNA is not dependent on its level of expression, its GC content or its gene length. However, GC content may influence the level of expression of a gene negatively (Fig. 3D), since there is a weak negative linear relationship between the expression level of genes and their GC content (R^2^ = 0,14). We did not detect any relevant bias in coverage depending on the GC content when the whole genome of different *S. maltophilia* D457 mutants is sequenced^35^.

### Messenger RNA half-lives and the biological role of the gene

**Figure 4:**
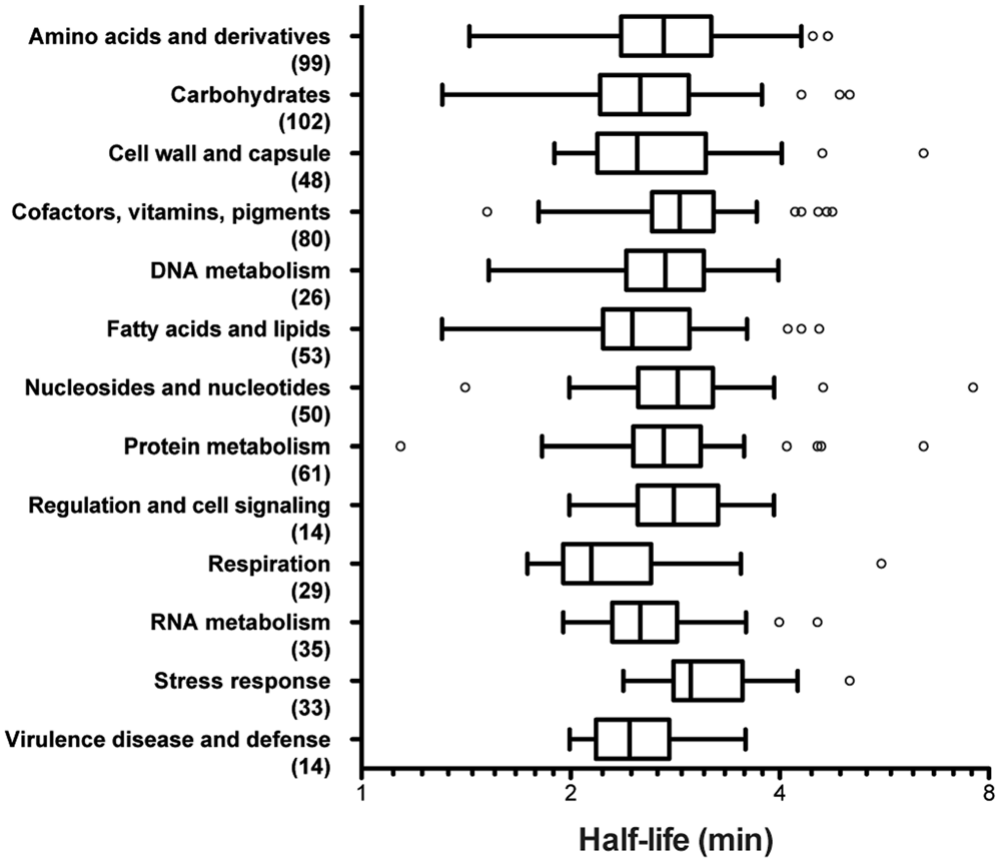
Distribution of mRNA half-lives for *S. maltophilia* D457 according to gene function and depicted by a boxplot at log scale. Each box comprises the 25 to 75 percentile of measured mRNA half-lives for the indicated gene function (the 50% of the calculated half-life values). Whisker top bar corresponds to maximum half-life except from outlier values (over Q3 + 1,5(IQR)) and whisker bottom bar corresponds to minimum half-life except from outlier values (under Q1 − 1,5(IQR)). The horizontal bar inside each box marks the median mRNA half-life in each group. Functions of genes were sorted by classifying the product of the gene by the EC nomenclature grouping them by known categories of biological processes. The number of genes in each category is shown in parentheses.  Statistical analysis is shown in the Table S5. Q1: Quartile 1; Q3: Quartile 3; IQR: Interquartile Range.

An analysis of genes grouped by their catalytic functions was carried out to determine if the involvement of a gene in a specific biological process influences the half-life of its mRNA in *S. maltophilia* D457. For this, we used the EC classification of enzymes (Enzyme Committee by the International Union of Biochemistry and Molecular Biology, http://www.chem.qmul.ac.uk/iubmb/enzyme/) to sort the genes of *S. maltophilia* genome, and CDS were grouped by categories and subcategories (Tables S3 and S4). Five hundred and forty genes were used for this study, some of which were classified in more than one subcategory (Fig. 4). Statistical analysis of the distribution of mRNA half-lives is shown in Table S5. Groups of genes whose products encode proteins involved in stress response; cofactors; nucleosides and nucleotides and regulation and cell signalling were associated with longer half-lives. Classes containing gene products involved in respiration; virulence, disease and defence; fatty acids and lipids; cell wall and capsule and carbohydrates were among those with the shortest half-lives (Fig. 4). However, by grouping genes in subcategories, some subgroups from different categories showed the longest half-lives, such as the subcategories riboflavin, FMN and FAD; oxidative stress and DNA repair (Table S3). In contrast, the subcategory of genes encoding proteins involved in electron donating reactions had the mRNAs’ shortest half-lives, as well as the genes involved in capsular and extracellular polysaccharide synthesis, monosaccharides metabolism and fatty acids synthesis. By analysing genes in subcategories, we could discriminate more specifically which groups of genes in each of the categories have the longest or the shortest half-life. This analysis suggests that, at least some mRNA half-lives, may have been selected depending on the functional role of the enzyme that the messengers encode.

### Effect of RNase G in *S. maltophilia* mRNA stability

**Figure 5:**
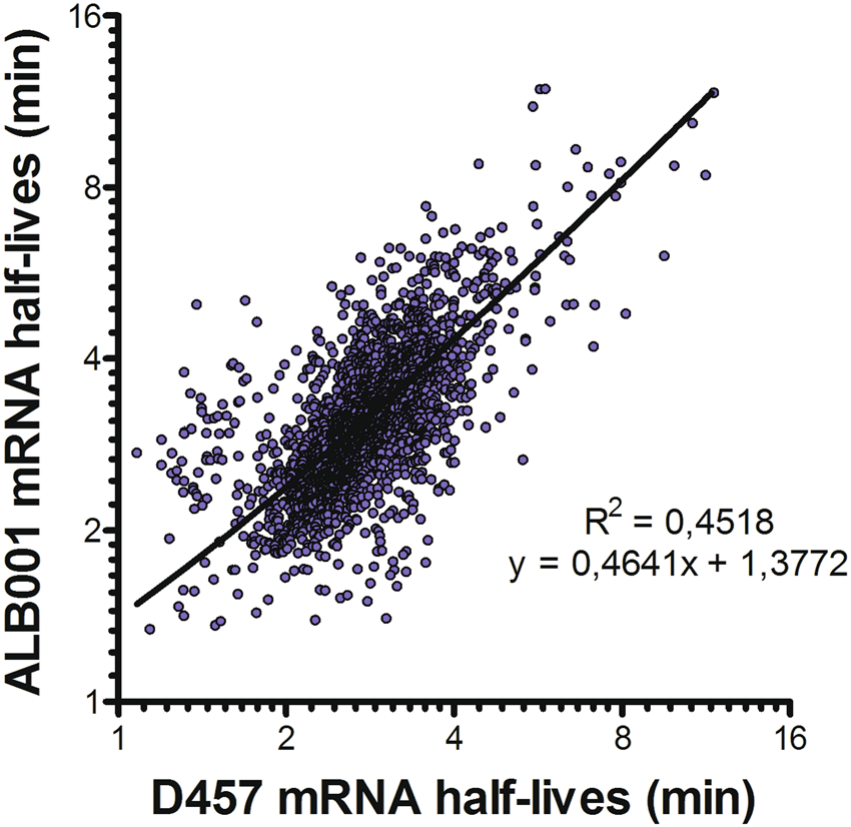
Comparison of mRNAs half-lives in *S. maltophilia* strains D457 and ALB001. Messenger RNA half-lives determined by RNA-Seq Illumina 1.9 in *S. maltophilia* strains D457 and ALB001 were correlated between them. Half-lives are shown at log scale.

To determine the potential mRNA targets of RNase G we have analysed the transcripts presenting higher mRNA half-lives in the *rng*-deficient *S. maltophilia* strain ALB001 than in the wild-type strain. The overall distribution of mRNA half-life values is different in ALB001 vs. D457. Fifty percent of the mRNA half-lives are in a narrower range in D457 than in ALB001 (0.84 and 1.16 interquartile range for D457 and ALB001 respectively) and they are overall higher in ALB001 than in D457 (Fig. 2). However, a comparison of mRNA half-lives in the two strains showed a correlation of 0.45 (Fig. 5) meaning that many mRNAs have different half-lives in one and in the other strain, although they have a positive linear relationship. As expected, in mutant ALB001 mRNA half-lives are generally higher than in the wild-type D457 strain. Indeed, Fig. 2 shows that there is a shift in values of mRNA half-lives in the mutant lacking *rng* as compared with these from the wild-type strain D457. As stated before, statistical quantifiers as median, mean and quartiles are higher in the case of the distribution of mRNAs half lives from of ALB001 than in the case of D457 (Fig. 2 and Table 4). Together, these data support an overall increase of half-life times of mRNAs in *S. maltophilia* when RNase G is absent.

In addition to this general trend, the stability of some mRNAs is largely improved in the ALB001 mutant. Indeed, 43 genes presented at least a 100% increase in their mRNA half-lives in the ALB001 mutant as compared with the wild-type D457 strain (Table S1). Among these genes, we can highlight 3 transcriptional regulators belonging to the LysR (SMD_0469), TetR (SMD_2350) and PadR (SMD_1499) families, a carbon storage regulator (SMD_1681), one DNA (SMD_2009) and one RNA binding protein (SMD_1667), one DNA-binding response regulator (SMD_1260) and a copper resistance protein (SMD_1486). However, most of the mRNAs presenting substantially higher half-lives in the ALB001 mutant than in the wild-type D457 strain, encode proteins with unknown function. To further verify the effect of RNase G deficiency over RNA stability of specific genes we studied by RT-qPCR the RNA levels of two genes, SMD_2876 and SMD_0264 that presented double RNA half-lives in ALB001 than in D457 by RNA-Seq analysis. In agreement with RNA-Seq results, RNA half-lives are about two-fold higher in ALB001 than in D457 strain for both genes (Figure S2). These data further suggest that RNase G specifically regulates mRNA degradation of some genes.

## Discussion

In this study, we report a genome-wide analysis of *S. maltophilia* mRNA half-lives including nearly 50% of the genes of this microorganism. We also propose a simple RT-qPCR-based method, modified from a previous study^31^, that might be applicable to other bacterial organisms, for the normalization of the information derived from RNA-Seq analysis, using a highly expressed gene. Using this method, we estimated the mRNA half-lives of 2674 genes in *S. maltophilia* D457 and 2449 genes in its derived *rng*-deficient mutant ALB001. In previous studies with other microorganisms, validation of globally-analysed mRNA half-lives was usually performed by Northern blot analysis^3,33,36^. However, in our method the correctness of the normalization procedure was verified by comparing the mRNA half-lives determined by RNA-Seq with mRNA half-lives determined by RT-qPCR for 4 independent genes (Table 3), which is a more robust approach for bacteria short-lived messengers.

The parameters of the distribution of mRNA half-lives determined in this study were in the range of those published for other microorganisms. The average of mRNA lifetimes determined by RNA-Seq for *E. coli* in exponential phase was 2.5 min^5^, similar to our 2.88 min. In the Gram-positive bacterium *Bacillus cereus*, a median of 2.4 and 2.6 min was reported for 2 strains respectively by a genome-wide RNA-Seq study, similar to our median of 2.74 min^31^. *Sulfolobus*, which belongs to the archaea domain and is a much slower growing microorganism, presents median mRNA half-lives of 5 min^29^. Despite structural and physiological differences among non-eukaryotic microorganisms, prokaryotic (and likely archaea) mRNA half-lives are rather similar among different taxa and the differences observed might, at least in occasions, be caused by differences in the methodologies (RNA-Seq or hybridization to microarrays) used in the analysis^3,30^. Contrary to this situation eukaryotic mRNAs have longer half-lives^36–38^ than prokaryotic ones. This situation likely reflects the time needed for transcript processing and transport outside the nucleus in eukaryotes as well as the need of prokaryotes for rapidly reprogramming gene expression upon sudden changes in environmental changing conditions^29^.

Multidrug efflux pumps are a paradigm of genes whose mRNAs are included in the same messenger molecule forming an operon, however just few studies have reported the operon structure of other genes of less studied bacteria genomes as those of *S. maltophilia* ^25,39^. We present here a prediction of the operon structure of *S. maltophilia* D457 based on proximity of CDS and expression values obtained by RNA-Seq. In accordance with our data, a *groESL* operon was confirmed previously based on sequence data and Northern blot analysis^39^. A *dnaK-dnaJ* operon was also described and *grpE* was discarded from being part of this operon due to promoter-like sequences upstream of *grpE* and *dnaK* ^25,39^; our analysis did not detect this operon. As expected, some RNAs from genes included in the same multi-gene operon have similar half-lives, but data are not conclusive in all cases. Detailed studies of promoter sequences, transcription starting and termination sites are need to confirm the *S. maltophilia* operon genome structure reported here.

Since the number of RNases in a cell is relatively low compared with the number of different mRNAs, it is possible that the decay of each mRNA is to some extent dictated by intrinsic factors of the RNA molecule itself^6^ as G + C content^31^ or gene length. Further, since the decay of a mRNA molecule is a very regulated process, it was proposed that steady state gene expression level itself is an important factor modulating the mRNA stability^2,38,40^. Notably, none of these factors seems to play a relevant role in *S. maltophilia* mRNA half-lives (Fig. 3), indicating that in this bacterial species regulation of mRNA stability could depend on other factors. Indeed, prior studies have shown that there is a correlation between gene function and mRNA half-life for many organisms^3,13,29–31,36,38^, although the molecular basis for this mRNA behaviour are not known. For instance, transcripts of genes under the categories of amino acid synthesis or macromolecule synthesis and modification have shorter half-lives than average, whereas those involved in cell envelope maintenance had longer-than-average half-lives^3^. Also, genes involved in carbohydrates metabolism, in energy metabolism and in translation generally had longer half-lives,^3,30,31^. In our study, mRNAs of genes related to monosaccharide’s metabolism presented shorter half-lives than others, while transcripts of genes encoding proteins of oxidative stress were found among the categories with the longest half-lives. Although stress responses are usually fast and transient, bacteria were not subject to any stress condition in our experimental conditions, which might justify these results. It should be noted that the different studies use different gene function classification schemes and hence comparisons are not always feasible. In addition, many *S. maltophilia* genes are not unequivocally annotated. Therefore, some categories are represented by just few genes precluding comparisons with other organisms.

The inactivation of RNase G leads to minor changes in mRNA half-lives in *E. coli* ^14^. However, increasing RNase G expression in a RNase E-deficient *E. coli* strain leads to a recovery of the decay of some, but not all of the mRNAs targeted by RNase E^13^. Further, single amino acid changes in *E. coli* RNase G complement RNase E deficient *E. coli* mutants^41^. It then seems that RNase G, a paralog of RNase E, is requested in *E. coli* to process just some mRNAs, which are targets as well of RNase E. Different to this situation; since an overall increase of mRNA half-lives is evident in *rng*-deficient *S. maltophilia* mutant, RNase G seems to have a much wider effect in *S. maltophilia* mRNA stability than in *E. coli*.

*E. coli* strains deficient in genes encoding the exonuclease PNPase and the metabolic enzyme enolase, and expressing a truncated C-terminal RNase E, which are three of the four main protein components of degradosome, overexpress *rng*, confirming functional complementarity of RNase G with degradosome components^42^. We also detected that the lack of RNase G causes a variation in the overall *S. maltophilia* mRNA half-lives, suggesting that mRNA decay becomes less efficient when RNase G is absent, as the decay machinery cannot degrade mRNAs at their normal timing. Together, these data suggest that RNase G plays an important role in mRNA regulated degradation in addition to the RNA degradosome.

Beyond this global effect, some gene transcripts could be exclusively targeted by RNase G, since inactivation of *rng* gene led to a large increase in the half-lives of the mRNAs of several genes. Some of these genes are transcriptional regulators that regulate a diverse set of genes. The increase in mRNA stability of these genes suggests that the turnover of these mRNAs may depend directly on RNase G, although more experiments are needed to verify the direct effect of RNase G over these RNAs. Lack of RNase G could generate an indirect response in the expression of other ribonucleases, in a similar way as it was described in the case of RNase R in *P. putida* ^43^ and with different RNases in *E. coli* ^42^, producing compensatory changes in the RNA decay machinery. These changes may facilitate normal growth despite not fully restored mRNA turnover, masking the identity of targets of the ribonuclease. However, although the ALB001 mutant has normal growth compared with the wild-type strain^18^, our transcriptome analysis showed that none of the other *S. maltophilia* ribonucleases was overexpressed when *rng* is inactivated (Table 5). Therefore, the lack of RNase G is not compensated through the increased expression of another RNase in *S. maltophilia*.

**Table 5:** Effect of the deletion of RNase G in the expression of the other *S. maltophilia* D457 ribonucleases.

Synonym	Name	Ribonuclease	Expression (RPKM)^a^		Fold change
			D457	ALB001	
SMD_3442	—	endoribonuclease L-PSP	548	428	0,78
SMD_2823	rnE	ribonuclease E	267	276	1,03
SMD_4238	rnpA	ribonuclease P protein component	258	255	0,99
SMD_1375	rnt	ribonuclease T	181	151	0,83
SMD_3126	rnc	ribonuclease III	179	146	0,82
SMD_0936	rnhA	ribonuclease HI	174	216	1,24
SMD_1367	rnr	3′-to-5′ exoribonuclease RNase R	161	163	1,01
SMD_3054	rnG	ribonuclease G	131	127	0,97
SMD_2617	orn	3′-to-5′ oligoribonuclease	126	128	1,02
SMD_3455	rph	ribonuclease PH	98	82	0,84
SMD_1324	rnhB	ribonuclease HII	94	71	0,76
SMD_2908	rnd	ribonuclease D	77	67	0,87
SMD_0745	—	ribonuclease BN	52	61	1,17
SMD_0372	—	ribonuclease BN	38	45	1,18
SMD_2815	—	ribonuclease	32	33	1,03
SMD_3815	—	Extracellular ribonuclease precursor	7	11	1,57

^a^Expression in mid-exponential phase of *S. maltophilia* D457 culture growing in LB medium^18^.

## Conclusions

We report a method for the normalization and quantification of bacterial mRNAs derived from global transcriptomics data. This method has allowed us to globally analyse, for the first time, the half-lives of the mRNAs of the relevant opportunistic pathogen *S. maltophilia*. In addition, we investigated the role that RNase G has in mRNA stability in this bacterial, antibiotic resistant, pathogen. Together, we show that not only *S. maltophilia* RNase G affects the stability of mRNAs of at least 40 genes, but also modulates at a global level the stability of a large number of mRNAs in *S. maltophilia*.

## Methods

### Bacterial growth and RNA isolation

The bacterial strains used in this work were *S. maltophilia* D457^44^ and the *S. maltophilia* D457 *rng*-defective mutant ALB001^18^. Flasks containing 25 ml of LB medium were inoculated with overnight cultures of either *S. maltophilia* D457 or ALB001 to 0.01 OD_600 nm_ and were subsequently grown at 37 °C, 250 rpm to mid-exponential phase (OD_600 nm_ = 0.6). To synchronize the cultures, new flasks containing 25 ml of LB medium were inoculated with the aforementioned cultures to 0.01 OD_600 nm_ and grown again at 37 °C, 250 rpm to mid-exponential phase (OD_600 nm_ = 0.6). A rifampicin run-out experiment was conducted as previously described^18^. Two and a half millilitres of cultures (1/10) were inoculated in flasks containing 22,5 ml of prewarmed LB and at a final concentration of 200 μg/ml rifampicin. This concentration of rifampicin was 4 times the minimal inhibitory concentration of this antibiotic against *S. maltophilia* D457 as tested by using the broth dilution method. Each rifampicin-arrested sample was used to extract RNA every five minutes. Five ml samples obtained at any given time were spun down for 20 min at 6,000 × *g*, 4 °C and pellets were immediately frozen on dry ice and stored at −80 °C. Total RNA extraction, DNA elimination, RNA integrity verification and confirmation of DNA absence in the samples were performed as described previously^45^. Total RNA was extracted and cleaned up from cell pellets using the RNeasy mini-kit (Qiagen) including the optional on-column DNase treatment according to the manufacturer’s instructions. To further eliminate any remaining DNA, Turbo DNA-free (Ambion) was used. RNA concentration was measured by spectrophotometer (NanoDrop ND-1000). RNA integrity was verified on a 1% agarose gel, and the absence of DNA was verified by PCR using *S. maltophilia* specific primers Sme27 (5′ TGCCAGCGACAGTGCAAAGGGTC 3′) and Sme48 (5′ CCGTGTTCATGGAAGCAGGC 3′) that amplify a *S. maltophilia* specific region^46^. The experiment was carried out twice for each strain.

### cDNA synthesis and RT-qPCR

Complementary DNA generation and real-time PCR were performed as described previously^45^ with modifications. Complementary DNA was obtained from 5 μg RNA (not depleted for rRNA) using a High Capacity cDNA reverse transcription (RT) kit (AB Applied Biosystems). Real -time PCR mixture was obtained using the Power SYBR green kit (Applied Biosystems) as indicated by the manufacturer. Fifty ng of 10x diluted cDNA (for gene-specific primers) or 1 ng of 500x-diluted cDNA (for 16S rRNA primers) were included in 25 μl of qPCR reaction. The primers used in the RT-qPCR reaction are shown in Table 6. All primers were used in a final concentration of 200 nM, except for rpoD1 and rpoD2 that were used at a final concentration of 500 nM. The reaction was performed as follows; a first denaturation step, 95 °C for 10 min, was followed by 40 cycles (95 °C for 15 s, 60 °C for 1 min). Differences in the relative amounts of mRNA for the different genes were determined according to the 2^−∆CCT^ method^47^ and by using 1/50 diluted 16S rRNA levels of expression for normalization.

**Table 6:** Primers used along this work for real time RT-PCR.

Primers	5′-3′ sequence	Source
**groEL 1**	AAGAAGGTGCAGGTCTCCAA	18
**groEL 2**	TCGTAATCCGAGGAGGTGTC	
**groES 1**	CCAAGGAAAAGTCCACCAAG	18
**groES 2**	CGTACTGGCCGTAGATGACC	
**gyrA(+)**	CCAGGGTAACTTCGGTTCGA	49
**gyrA(−)**	GCCTCGGTGTATCGCATTG	
**rnasaG 1**	GAGGACATCGCCTACCTGTC	18
**rnasaG 2**	ACCTTCACCTTGTCCACGTC	
**ftsZ1**	ATGCAGGTGGCGCTGAA	28
**ftsZ2**	GCTTCTCGTTCGGGATGGT	
**rpoD1**	GGTGCACATGATCGAAACGA	50
**rpoD2**	GCCGTACTGCTGGAGCATCT	
**16rDNAq-F**	GACCTTGCGCGATTGAATG	51
**16rDNAq-R**	CGGATCGTCGCCTTGGT	
**SMD_0264_1**	GTTATGCCCATTGCCTGAT	This work
**SMD_0264_2**	CATCACCGTTGGAGGACAC	
**SMD_2876_1**	GACGAAGAGGCCGTTGAG	This work
**SMD_2876_2**	ACGTAATCCACGTCGTAGGC	

### RNA-Seq, cDNA library preparation and sequencing

Duplicated RNAs from each strain and each time point were pooled to reduce biological variability (4 + 4 μg). Sample concentration and integrity was checked in a Bioanalyzer 2100 (Agilent) (RIN > 8.2 all samples). RNA samples (3.5 µg each) were rRNA depleted with RiboZero (Epicentre), and rRNA depletion of the recovered RNA was confirmed using Bioanalyzer 2100. RNA was then subjected to RNA library preparation using the Ultra Directional RNA Library Prep Kit (Cat no. E7420S, New England Biolabs) following manufacturer recommendations. Libraries were pooled and fragments with lengths between 200 and 500 bp were purified from agarose gels. Remaining adapter dimers were cleaned with AMPure XP beads (Beckman). Libraries were sequenced in a Miseq system (Illumina) using a v3 cartridge and a 150 bp, single-read format at the Parque Científico de Madrid.

### Informatic analysis of sequences and mRNA half-life analysis

To validate the rifampicin run-out experiment a RT-qPCR analysis was carried out with 2 genes presenting high expression levels at exponential growth phase: *groEL* and *groES*. Expression levels of these genes were calculated using the 2^−∆CCT^ method^47^, mRNA half-lives (T_1/2_) were calculated from the equation T_1/2_ = ln(2)/µ, where the constant µ was calculated from the mean decay rate obtained by the logarithmic regression model adjustment of the expression data. Coefficients of correlation of the expression data to the logarithmic regression model showing RNA decay were bigger than 0.98, confirming the suitable adjustment of the data.

To calculate the half-lives of all mRNAs of *S. maltophilia* D457 and ALB001, data of deep sequencing were used. Raw data from deep sequencing were trimmed, filtered by quality, mapped and indexed against the reference genome SMD457, with accession number HE798556^17^. The trimming and the quality filtering was performed using Fastq_trimmer and Alien_trimmer. The procedure consisted of removing the first 10 bp of all sequences, removing adapters (with no mismatches and with at least 10 bp), removing reads shorter than 30 bp and keeping reads with at least 28 of *c* quality. The fastq data files obtained from trimmed and filtered RNA deep sequencing data were analysed using Rockhopper software to map reads against the reference genome SMD457 and to obtain the values of gene expression. The bam archives were obtained mapping reads against reference genome with Bowtie version 0.11.3. To compare gene expression levels of different genes, the RNA-sequencing data were normalized as the total number of reads mapped to a CDS, and divided by the length of the CDS in kilobases (RPK: reads per kilobase). Values are generally normalized by the total number of unambiguously mapped reads in each sample, giving the value of RPKM^48^. Expression values obtained by Rockhopper for each transcript in each condition are similar to RPKM, but the expression levels are normalized by the upper quartile of gene expression, which is a more robust normalization approach. Since all expression data were analysed using this program and because results are equivalent for each kind of normalization in all cases, we have named as RPKM to the normalized values of expression obtained using Rockhopper.

To adjust for unequal amounts of cDNA in each sample and the expected fall in mRNA expression levels during the 15 minutes time-course, as well as for eliminating the putative bias due to potentially variable efficiency of rRNA depletion, the following procedure was carried out for normalization. Any rRNA remains and any ncRNA data were excluded from the analysis. Genes with RPKM data at t_0_ < 15 were excluded from the analysis. Since no rRNA is present in RNA samples subjected to deep sequencing, 16S rRNA could not be used for normalization. To normalize expression data of samples from different time points, data from *groES* RT-qPCR during the 15 min after rifampicin addition were used to calculate the expected values of *groES* mRNA decay from expression data of deep sequencing^31^. Afterwards, these *groES* expression data were used to normalize the expression data of all other genes. The µ statistic used to calculate the half-life of each CDS and the coefficient of the degree of the adjustment of the data (R^2^) were obtained by fitting the data into a logarithmic regression model. The output mRNA half-lives of genes which logarithmic regression adjustment of the data that had a R^2^ > 0.7 were considered. By using this approach, it was possible to determine the mRNA half-lives of 2674 genes for the D457 strain, and 2449 for the ALB001 strain. To validate mRNA half-lives calculation procedure, mRNA half-lives were determined by RT-qPCR for 4 independent genes, which levels of expression in exponential growth phase were bigger than 300 RPKM (*groEL*, *rpoD*, *gyrA* and *ftsZ*)^18^, by using *S. maltophilia* D457 non-rRNA depleted samples from time points t0, t5, t10 and t15.

### Prediction of operons

Rockhopper software has a tool to predict multi-gene operon. To estimate the probability that consecutive genes on the same strand are co-transcribed as part of a multi-gene operon, two features are taking into account: the distance in nucleotides between the genes and the similarity of the gene expression in the RNA-Seq data.

### RNA-Seq Data Accession Number

The RNA-Seq data described in this article were deposited in the GEO (Gene Expression Omnibus) database of NCBI (accession number GSE103467).

### Availability of Data and Material

The datasets generated during the current study are included in this published article (and its Supplementary Information files).

## Electronic supplementary material

Supplemental Material

## References

[1] Perez-Ortin JE, Alepuz PM, Moreno J (2007). Genomics and gene transcription kinetics in yeast. Trends Genet.

[2] Keene JD (2010). The global dynamics of RNA stability orchestrates responses to cellular activation. BMC Biol.

[3] Bernstein JA, Khodursky AB, Lin PH, Lin-Chao S, Cohen SN (2002). Global analysis of mRNA decay and abundance in Escherichia coli at single-gene resolution using two-color fluorescent DNA microarrays. Proc Natl Acad Sci USA.

[4] Wurtzel O (2010). A single-base resolution map of an archaeal transcriptome. Genome research.

[5] Chen H, Shiroguchi K, Ge H, Xie XS (2015). Genome-wide study of mRNA degradation and transcript elongation in Escherichia coli. Mol Syst Biol.

[6] Hambraeus G, von Wachenfeldt C, Hederstedt L (2003). Genome-wide survey of mRNA half-lives in Bacillus subtilis identifies extremely stable mRNAs. Mol Genet Genomics.

[7] Kushner SR (2004). mRNA decay in prokaryotes and eukaryotes: different approaches to a similar problem. IUBMB Life.

[8] Carpousis AJ (2002). The *Escherichia coli* RNA degradosome: structure, function and relationship in other ribonucleolytic multienzyme complexes. Biochem Soc Trans.

[9] Mackie GA (2013). RNase E: at the interface of bacterial RNA processing and decay. Nature reviews. Microbiology.

[10] Drider D, Condon C (2004). The continuing story of endoribonuclease III. J Mol Microbiol Biotechnol.

[11] Ito R, Ohnishi Y (1983). The roles of RNA polymerase and RNAase I in stable RNA degradation in *Escherichia coli* carrying the srnB+ gene. Biochim Biophys Acta.

[12] Umitsuki G, Wachi M, Takada A, Hikichi T, Nagai K (2001). Involvement of RNase G in *in vivo* mRNA metabolism in *Escherichia coli*. Genes Cells.

[13] Lee, K., Bernstein, J. A. & Cohen, S. N. RNase G complementation of *rne* null mutation identifies functional interrelationships with RNase E in *Escherichia coli*. *Mol Microbiol* **43** (2002). 10.1046/j.1365-2958.2002.02848.x 11952897

[14] Ow MC, Perwez T, Kushner SR (2003). RNase G of *Escherichia coli* exhibits only limited functional overlap with its essential homologue, RNase E. Mol Microbiol.

[15] Richards J, Belasco JG (2016). Distinct Requirements for 5′-Monophosphate-assisted RNA Cleavage by Escherichia coli RNase E and RNase G. J Biol Chem.

[16] Eidem TM, Roux CM, Dunman PM (2012). RNA decay: a novel therapeutic target in bacteria. Wiley Interdiscip Rev RNA.

[17] Lira F (2012). Whole-genome sequence of *Stenotrophomonas maltophilia* D457, a clinical isolate and a model strain. J Bacteriol.

[18] Bernardini A, Corona F, Dias R, Sanchez MB, Martinez JL (2015). The inactivation of RNase G reduces the *Stenotrophomonas maltophilia* susceptibility to quinolones by triggering the heat shock response. Front Microbiol.

[19] Brooke JS (2012). *Stenotrophomonas maltophilia*: an emerging global opportunistic pathogen. Clin Microbiol Rev.

[20] Walsh TR, MacGowan AP, Bennett PM (1997). Sequence analysis and enzyme kinetics of the L2 serine beta-lactamase from Stenotrophomonas maltophilia. Antimicrob Agents Chemother.

[21] Lambert T, Ploy MC, Denis F, Courvalin P (1999). Characterization of the chromosomal aac(6′)-Iz gene of Stenotrophomonas maltophilia. Antimicrob Agents Chemother.

[22] Alonso A, Martinez JL (2000). Cloning and characterization of SmeDEF, a novel multidrug efflux pump from *Stenotrophomonas maltophilia*. Antimicrob Agents Chemother.

[23] Avison MB (2002). Differential regulation of L1 and L2 beta-lactamase expression in *Stenotrophomonas maltophilia*. The Journal of antimicrobial chemotherapy.

[24] Okazaki A, Avison MB (2007). Aph(3′)-IIc, an aminoglycoside resistance determinant from *Stenotrophomonas maltophilia*. Antimicrob Agents Chemother.

[25] Crossman LC (2008). The complete genome, comparative and functional analysis of *Stenotrophomonas maltophilia* reveals an organism heavily shielded by drug resistance determinants. Genome biology.

[26] Sanchez MB, Hernandez A, Rodriguez-Martinez JM, Martinez-Martinez L, Martinez JL (2008). Predictive analysis of transmissible quinolone resistance indicates *Stenotrophomonas maltophilia* as a potential source of a novel family of Qnr determinants. BMC microbiology.

[27] Shimizu K (2008). Sm*qnr*, a new chromosome-carried quinolone resistance gene in *Stenotrophomonas maltophilia*. Antimicrob Agents Chemother.

[28] Garcia-Leon, G., Salgado, F., Oliveros, J. C., Sanchez, M. B. & Martinez, J. L. Interplay between intrinsic and acquired resistance to quinolones in *Stenotrophomonas maltophilia*. *Environmental microbiology* (2014). 10.1111/1462-2920.12408 24447641

[29] Andersson AF (2006). Global analysis of mRNA stability in the archaeon Sulfolobus. Genome biology.

[30] Selinger DW, Saxena RM, Cheung KJ, Church GM, Rosenow C (2003). Global RNA half-life analysis in Escherichia coli reveals positional patterns of transcript degradation. Genome research.

[31] Kristoffersen, S. M. *et al*. Global mRNA decay analysis at single nucleotide resolution reveals segmental and positional degradation patterns in a Gram-positive bacterium. *Genome biology* **13** (2012). 10.1186/gb-2012-13-4-r30 PMC3446304 22537947

[32] Ignatov DV (2015). Dormant non-culturable Mycobacterium tuberculosis retains stable low-abundant mRNA. BMC genomics.

[33] Liu B (2014). Global analysis of mRNA decay intermediates in Bacillus subtilis wild-type and polynucleotide phosphorylase-deletion strains. Mol Microbiol.

[34] Hartmann G, Honikel KO, Knusel F, Nuesch J (1967). The specific inhibition of the DNA-directed RNA synthesis by rifamycin. Biochim Biophys Acta.

[35] Lira, F., Berg, G. & Martinez, J. L. Double-face meets the bacterial world: the opportunistic pathogen *Stenotrophomonas maltophilia*. *Front Microbiol* **8**, 2190 (2017). 10.3389/fmicb.2017.02190 PMC5684188 29170656

[36] Wang, Y. *et al*. Precision and functional specificity in mRNA decay. *Proc Natl Acad Sci USA* **99**, 5860–5865 (2002). 10.1073/pnas.092538799 PMC122867 11972065

[37] Lam LT (2001). Genomic-scale measurement of mRNA turnover and the mechanisms of action of the anti-cancer drug flavopiridol. Genome biology.

[38] Yang E (2003). Decay rates of human mRNAs: correlation with functional characteristics and sequence attributes. Genome research.

[39] De Carolis E (2011). Analysis of heat-induced changes in protein expression of Stenotrophomonas maltophilia K279a reveals a role for GroEL in the host-temperature adaptation. Int J Med Microbiol.

[40] Papenfort K, Vogel J (2009). Multiple target regulation by small noncoding RNAs rewires gene expression at the post-transcriptional level. Res Microbiol.

[41] Chung DH, Min Z, Wang BC, Kushner SR (2010). Single amino acid changes in the predicted RNase H domain of Escherichia coli RNase G lead to complementation of RNase E deletion mutants. RNA.

[42] Zhou L, Zhang AB, Wang R, Marcotte EM, Vogel C (2013). The proteomic response to mutants of the Escherichia coli RNA degradosome. Molecular bioSystems.

[43] Fonseca P, Moreno R, Rojo F (2008). Genomic analysis of the role of RNase R in the turnover of Pseudomonas putida mRNAs. J Bacteriol.

[44] Alonso A, Martinez JL (1997). Multiple antibiotic resistance in *Stenotrophomonas maltophilia*. Antimicrob Agents Chemother.

[45] Olivares J (2012). Overproduction of the multidrug efflux pump MexEF-OprN does not impair *Pseudomonas aeruginosa* fitness in competition tests, but produces specific changes in bacterial regulatory networks. Environmental microbiology.

[46] Sanchez P, Alonso A, Martinez JL (2002). Cloning and characterization of SmeT, a repressor of the *Stenotrophomonas maltophilia* multidrug efflux pump SmeDEF. Antimicrob Agents Chemother.

[47] Livak KJ, Schmittgen TD (2001). Analysis of relative gene expression data using real-time quantitative PCR and the 2(-Delta Delta C(T)) Method. Methods.

[48] Mortazavi A, Williams BA, McCue K, Schaeffer L, Wold B (2008). Mapping and quantifying mammalian transcriptomes by RNA-Seq. Nat Methods.

[49] Hernandez A, Ruiz FM, Romero A, Martinez JL (2011). The binding of triclosan to SmeT, the repressor of the multidrug efflux pump SmeDEF, induces antibiotic resistance in *Stenotrophomonas maltophilia*. PLoS Pathog.

[50] Sanchez MB, Martinez JL (2010). SmQnr contributes to intrinsic resistance to quinolones in *Stenotrophomonas maltophilia*. Antimicrob Agents Chemother.

[51] Yang TC, Huang YW, Hu RM, Huang SC, Lin YT (2009). AmpDI is involved in expression of the chromosomal L1 and L2 beta-lactamases of Stenotrophomonas maltophilia. Antimicrob Agents Chemother.

