# Supplementary material for: Genome-wide analysis shows that RNase G plays a global role in the stability of mRNAs in Stenotrophomonas maltophilia
Source: Sci Rep. 2017 Nov 22;7:16016. doi: 10.1038/s41598-017-16091-0 (PMC5700063; doi:10.1038/s41598-017-16091-0)
Supplement: Supplementary file 1 — Supplemental Material [file 41598_2017_16091_MOESM1_ESM.pdf]

Genome-wide analysis shows that RNase G plays a global role in the stability of mRNAs in *Stenotrophomonas maltophilia.*

Alejandra Bernardini*, José L. Martínez**

Table S1. Level of expression and half-lives of RNAs from *S. maltophilia* D457 and ALB001

				D457		ALB001		
**Name**	**Synonym**	**Gene length (nt)**	**GC%**	**Expression mid-exponential phase (RPKM)**	**Half-life (min)**	**Expression mid-exponential phase (RPKM)**	**Half-life (min)**	**Half-life ratio ALB001/D457**
-	SMD_2568	225	63,56	28	6,66	32	30,29	4,55
-	SMD_3891	210	66,67	229	1,38	289	4,98	3,61
-	SMD_0469	882	70,41	14	1,69	15	5,06	3,00
-	SMD_3942	249	63,05	92	1,31	66	3,78	2,89
-	SMD_3757	366	68,58	11	6,31	11	16,66	2,64
-	SMD_3804	258	67,83	14	1,77	11	4,64	2,62
-	SMD_2709	393	65,39	53	1,35	56	3,48	2,58
-	SMD_3475	429	65,27	56	1,08	59	2,74	2,54
fkbP	SMD_1637	435	68,28	41	1,59	31	3,89	2,44
-	SMD_3016	396	66,92	11	1,61	5	3,92	2,44
-	SMD_3748	408	66,67	43	1,41	36	3,41	2,43
csrA	SMD_1681	204	58,82	617	1,19	646	2,89	2,42
-	SMD_2350	558	69,35	85	1,65	82	3,86	2,34
dut	SMD_0322	474	69,20	132	1,41	131	3,27	2,32
phaF	SMD_3918	285	63,16	61	1,41	65	3,22	2,28
-	SMD_3761	264	61,36	36	1,31	43	2,98	2,27
-	SMD_2009	783	66,92	87	1,31	86	2,96	2,26
-	SMD_1667	306	66,99	249	1,35	257	3,01	2,23
-	SMD_1591	246	64,23	31	2,42	51	5,35	2,22
-	SMD_0264	285	70,53	135	2,72	111	6,02	2,21
-	SMD_1993	249	69,08	151	1,25	119	2,73	2,19
-	SMD_2229	831	69,68	13	1,19	13	2,61	2,19
-	SMD_1260	684	70,76	54	1,68	45	3,65	2,18
-	SMD_2678	600	72,17	27	1,70	32	3,69	2,17
-	SMD_1499	303	65,02	27	1,45	25	3,13	2,17
xpsG	SMD_0582	444	68,24	57	1,54	55	3,32	2,16
-	SMD_4007	471	70,70	23	1,48	16	3,14	2,12
-	SMD_2991	681	68,14	59	1,51	55	3,17	2,10
copC	SMD_1486	387	62,79	42	5,70	37	11,90	2,09
DPM1	SMD_4143	723	68,19	117	3,56	125	7,41	2,08
-	SMD_0394	411	70,56	13	2,50	22	5,18	2,07
-	SMD_1978	300	72,67	138	1,44	141	2,97	2,06
cirA	SMD_3723	2046	66,23	13	3,06	13	6,26	2,05
-	SMD_1310	357	64,71	92	1,64	72	3,35	2,04
-	SMD_0560	600	66,00	54	5,83	79	11,90	2,04
-	SMD_0238	357	63,03	34	1,65	13	3,37	2,04
-	SMD_1670	186	66,67	61	1,30	79	2,65	2,04
-	SMD_1828	390	68,72	42	2,60	67	5,27	2,03
-	SMD_0418	927	66,99	35	1,56	26	3,17	2,03
-	SMD_2542	885	67,68	82	1,25	117	2,52	2,02
-	SMD_3430	645	70,39	26	2,94	25	5,91	2,01
-	SMD_2560	1008	68,45	5	5,54	4	11,09	2,00
cheW	SMD_2033	492	65,04	142	1,27	73	2,55	2,00
-	SMD_2876	564	68,97	298	4,42	284	8,79	1,99
-	SMD_3192	1410	67,66	23	2,08	18	4,13	1,98
-	SMD_0284	297	72,39	33	1,99	49	3,91	1,97
cdsA2	SMD_3841	996	65,36	48	2,28	35	4,47	1,96
-	SMD_1460	768	68,10	25	2,55	34	4,99	1,96
-	SMD_1022	396	71,46	75	3,65	86	7,11	1,95
folK	SMD_1715	486	69,96	92	1,52	96	2,96	1,95
-	SMD_0163	771	68,09	44	3,21	31	6,25	1,95
-	SMD_4058	747	66,93	29	1,77	18	3,42	1,93
-	SMD_4098	426	62,44	83	1,27	56	2,45	1,93
-	SMD_0521	1419	69,20	9	2,85	9	5,49	1,93
-	SMD_2273	414	68,84	14	1,94	17	3,74	1,92
-	SMD_1868	924	68,40	7	1,62	7	3,09	1,91
ptpS	SMD_0514	363	65,29	152	2,28	147	4,33	1,90
-	SMD_0129	375	69,60	14	2,53	17	4,77	1,89

-	SMD_1697	702	64,81	6	1,58	22	2,96	1,88
-	SMD_1889	468	67,74	19	1,44	18	2,70	1,87
-	SMD_2494	852	71,71	27	1,90	26	3,54	1,87
-	SMD_3197	573	68,06	29	2,30	43	4,30	1,87
-	SMD_3102	1593	67,73	34	2,99	27	5,57	1,87
cheY2	SMD_2045	393	61,58	253	1,31	151	2,44	1,86
-	SMD_2645	480	67,29	29	3,10	36	5,77	1,86
-	SMD_3913	486	64,61	51	2,67	68	4,95	1,85
-	SMD_2102	462	72,29	219	1,44	174	2,66	1,85
arsR	SMD_0133	330	66,97	16	3,15	18	5,77	1,83
-	SMD_1707	480	63,33	148	1,51	182	2,75	1,83
-	SMD_1459	285	68,77	27	3,55	26	6,49	1,83
-	SMD_2597	291	62,89	35	3,32	32	6,05	1,82
-	SMD_0448	408	66,67	41	1,46	35	2,66	1,82
-	SMD_3114	270	61,48	71	1,39	83	2,53	1,82
-	SMD_0999	675	70,37	29	2,23	35	4,04	1,81
-	SMD_2935	333	65,17	93	2,75	71	4,97	1,81
-	SMD_3558	780	71,03	54	2,95	57	5,28	1,79
-	SMD_4092	456	67,32	84	1,31	73	2,33	1,79
-	SMD_1874	765	66,27	17	2,24	16	3,98	1,78
-	SMD_1291	546	70,88	58	1,51	34	2,68	1,78
hslO	SMD_3211	894	69,91	51	2,18	82	3,83	1,75
-	SMD_2750	288	62,15	33	1,36	35	2,38	1,75
exbD5	SMD_3602	408	66,42	55	2,96	61	5,18	1,75
nagA2	SMD_4005	1116	71,42	21	2,27	29	3,97	1,75
-	SMD_2747	657	65,45	85	2,40	68	4,20	1,75
-	SMD_1169	597	66,00	354	2,26	368	3,95	1,75
-	SMD_3137	852	67,37	19	2,74	39	4,78	1,74
-	SMD_4233	771	70,56	19	3,82	13	6,66	1,74
-	SMD_0696	615	69,11	43	2,21	36	3,84	1,74
-	SMD_2929	327	61,16	14	1,54	4	2,66	1,73
-	SMD_3098	267	69,29	39	1,36	53	2,36	1,73
-	SMD_0060	828	69,93	74	2,14	112	3,70	1,73
-	SMD_0671	1068	69,10	29	2,29	29	3,95	1,73
kdpE	SMD_0324	687	66,96	31	3,48	41	6,01	1,72
-	SMD_1272	546	67,58	253	2,52	335	4,33	1,72
agp	SMD_1307	1269	67,06	24	2,71	24	4,65	1,71
-	SMD_1295	165	57,58	375	2,64	276	4,48	1,70
-	SMD_3929	876	66,10	52	2,81	46	4,76	1,69
-	SMD_4214	525	66,67	167	2,43	171	4,10	1,69
-	SMD_1406	1563	64,43	36	1,87	39	3,15	1,69
-	SMD_1469	489	69,33	39	1,38	51	2,32	1,69
-	SMD_3715	972	68,21	119	2,84	163	4,79	1,68
-	SMD_1302	948	68,25	65	4,02	67	6,74	1,68
tyrA	SMD_1799	1125	68,62	58	1,38	72	2,30	1,66
glnE	SMD_0399	2514	70,60	32	2,80	41	4,63	1,66
-	SMD_1055	270	67,41	118	1,49	121	2,46	1,65
qnrR	SMD_0997	777	71,56	15	1,64	35	2,70	1,65
-	SMD_1298	1629	70,84	19	2,87	12	4,71	1,64
-	SMD_4085	336	68,45	14	3,70	23	6,04	1,64
ubiA	SMD_3995	894	68,57	78	2,79	62	4,56	1,63
-	SMD_0294	603	69,32	18	2,35	17	3,82	1,63
-	SMD_1184	510	71,18	56	2,68	64	4,35	1,63
-	SMD_1059	732	68,44	39	1,80	37	2,93	1,62
-	SMD_3649	732	67,76	96	2,10	118	3,40	1,62
-	SMD_1641	498	63,86	36	2,72	37	4,42	1,62
-	SMD_3556	618	70,23	74	2,85	114	4,61	1,62
-	SMD_3707	819	63,13	22	3,63	37	5,88	1,62
I	SMD_3284	558	67,92	45	3,37	41	5,46	1,62
-	SMD_4073	858	67,37	51	3,03	41	4,90	1,62
rimP	SMD_2964	591	66,33	479	2,42	414	3,92	1,62
-	SMD_2688	729	67,22	31	2,78	34	4,49	1,61
-	SMD_0044	570	66,49	21	3,24	29	5,22	1,61
recF	SMD_0003	1095	66,48	54	2,89	68	4,65	1,61
-	SMD_3545	651	69,28	69	2,73	49	4,39	1,61

-	SMD_3675	897	69,23	48	3,55	53	5,69	1,61
ispG	SMD_1722	1152	65,19	259	2,33	242	3,74	1,60
-	SMD_1806	594	64,98	91	3,45	71	5,52	1,60
-	SMD_3762	1215	62,55	87	3,77	121	6,04	1,60
-	SMD_3564	1263	71,65	13	1,84	12	2,95	1,60
-	SMD_2480	2160	67,27	62	2,24	77	3,58	1,60
-	SMD_2038	309	66,67	181	1,37	83	2,18	1,60
-	SMD_1308	984	68,09	5	2,30	4	3,67	1,59
-	SMD_1241	369	67,48	44	2,66	31	4,25	1,59
-	SMD_4065	792	63,89	139	2,59	111	4,12	1,59
-	SMD_0388	1449	68,88	21	2,59	35	4,12	1,59
xpsJ	SMD_0585	633	71,25	18	2,27	21	3,61	1,59
-	SMD_3092	183	65,57	113	3,68	111	5,83	1,58
-	SMD_1990	789	71,61	44	1,44	53	2,28	1,58
kdtA	SMD_3533	1299	69,05	41	3,15	48	4,96	1,58
nudC	SMD_3883	906	68,76	118	2,76	139	4,34	1,57
fliG	SMD_2063	978	66,26	99	1,23	67	1,94	1,57
-	SMD_2820	417	69,54	121	2,26	114	3,54	1,57
-	SMD_1639	768	66,93	113	3,20	132	5,01	1,57
fliF	SMD_2064	1647	68,73	64	1,41	44	2,21	1,57
-	SMD_0672	828	66,18	51	2,71	61	4,23	1,56
-	SMD_2674	993	68,78	88	2,65	111	4,14	1,56
-	SMD_0633	480	64,79	71	5,60	71	8,75	1,56
-	SMD_1917	285	69,12	89	3,19	91	4,97	1,56
-	SMD_1082	597	69,18	47	3,66	61	5,71	1,56
-	SMD_3415	306	59,80	57	3,69	92	5,75	1,56
-	SMD_3769	501	72,85	22	2,49	15	3,87	1,56
-	SMD_0089	579	70,98	35	3,86	23	6,01	1,56
-	SMD_0463	930	71,18	23	2,72	29	4,22	1,55
-	SMD_1923	894	65,32	27	2,65	33	4,09	1,55
-	SMD_1211	336	68,45	69	3,35	69	5,17	1,54
-	SMD_4213	750	67,33	118	2,57	93	3,95	1,54
-	SMD_0315	468	65,38	161	3,69	144	5,67	1,54
flgE	SMD_2085	1224	63,73	135	2,62	116	4,02	1,54
adi	SMD_2577	2289	64,31	175	2,52	184	3,87	1,53
-	SMD_1053	711	67,23	56	1,82	51	2,79	1,53
-	SMD_1443	321	62,93	181	3,01	182	4,60	1,53
-	SMD_0260	375	69,33	356	2,90	334	4,44	1,53
aroQ	SMD_3832	450	68,67	191	2,54	178	3,89	1,53
-	SMD_0420	1014	65,68	41	2,22	31	3,39	1,53
-	SMD_0714	264	68,56	113	3,08	115	4,70	1,53
-	SMD_3691	1347	67,85	44	2,95	57	4,50	1,52
-	SMD_3802	618	66,34	55	4,13	55	6,29	1,52
fnr2	SMD_2434	660	66,82	61	1,78	45	2,70	1,52
-	SMD_2907	1887	70,43	45	3,07	54	4,67	1,52
-	SMD_3836	411	63,99	119	2,17	161	3,29	1,52
-	SMD_2170	909	61,94	25	2,62	33	3,98	1,52
-	SMD_1458	303	67,99	28	3,57	21	5,41	1,51
-	SMD_0120	939	69,54	65	3,11	74	4,70	1,51
-	SMD_0462	615	60,49	8	3,05	21	4,62	1,51
tatE	SMD_4173	228	65,79	342	3,46	341	5,24	1,51
-	SMD_0822	504	67,06	14	4,06	12	6,13	1,51
-	SMD_0395	753	65,74	251	2,59	234	3,91	1,51
obg	SMD_1193	1053	66,19	319	2,00	236	3,01	1,51
-	SMD_0076	474	69,62	51	2,50	41	3,76	1,50
-	SMD_1407	1593	60,33	43	2,97	39	4,47	1,50
-	SMD_2755	1185	65,49	27	1,89	23	2,84	1,50
-	SMD_0376	537	64,62	66	3,86	64	5,79	1,50
-	SMD_0058	921	67,75	153	2,53	131	3,79	1,50
ribD	SMD_0611	1119	70,60	81	3,08	81	4,61	1,49
agaR	SMD_4004	777	67,31	31	2,48	43	3,70	1,49
coaE	SMD_3362	612	70,75	62	2,77	36	4,14	1,49
-	SMD_0974	471	62,63	618	2,70	742	4,03	1,49
-	SMD_0382	453	66,89	78	2,67	81	3,97	1,49
-	SMD_0366	1167	67,87	116	3,29	131	4,89	1,49

-	SMD_3465	297	70,37	111	2,86	118	4,25	1,49
-	SMD_1934	1053	64,86	16	2,20	15	3,26	1,48
-	SMD_3487	579	68,74	91	2,25	111	3,34	1,48
-	SMD_2652	1524	69,95	25	3,40	32	5,04	1,48
pilO	SMD_3426	657	61,64	77	2,57	41	3,80	1,48
-	SMD_0629	1734	72,84	99	2,73	112	4,04	1,48
-	SMD_2639	1041	64,55	118	2,52	158	3,72	1,48
ccmA	SMD_2842	630	70,16	64	2,97	58	4,38	1,48
-	SMD_0556	1122	67,47	91	3,11	87	4,60	1,48
maf	SMD_3055	615	71,38	99	2,81	111	4,15	1,48
-	SMD_3860	1575	67,30	19	4,00	21	5,90	1,47
xseB	SMD_3047	261	65,52	328	2,98	325	4,38	1,47
-	SMD_3817	2382	69,35	42	2,61	62	3,84	1,47
yegD	SMD_3090	1362	70,70	32	3,40	28	5,00	1,47
-	SMD_2601	183	53,01	5	2,12	5	3,12	1,47
exo	SMD_0729	936	69,23	65	2,61	78	3,83	1,47
nusG	SMD_0773	561	62,21	1184	2,67	1154	3,92	1,47
-	SMD_4016	885	70,85	46	2,87	51	4,21	1,47
-	SMD_1619	258	65,12	87	2,25	77	3,29	1,47
trpE	SMD_3907	1476	67,07	112	4,46	111	6,54	1,47
mtnA	SMD_2718	1065	69,11	171	2,70	148	3,96	1,46
-	SMD_3178	123	54,47	79	1,65	88	2,41	1,46
-	SMD_1706	1401	64,88	125	2,04	138	2,98	1,46
-	SMD_1786	258	73,26	178	3,17	188	4,61	1,45
-	SMD_3671	324	75,93	11	4,29	2	6,24	1,45
-	SMD_0028	1041	68,01	129	3,35	135	4,87	1,45
-	SMD_0745	1266	65,80	52	2,76	61	4,01	1,45
rstA	SMD_0511	690	68,26	125	2,91	143	4,23	1,45
-	SMD_2767	606	66,67	63	2,02	56	2,94	1,45
-	SMD_2651	1737	65,28	21	3,10	21	4,50	1,45
-	SMD_2517	711	67,37	61	3,33	44	4,83	1,45
-	SMD_1301	1161	69,08	47	4,30	35	6,24	1,45
-	SMD_1780	864	65,74	41	2,74	45	3,98	1,45
-	SMD_0385	762	70,08	31	2,93	32	4,24	1,45
-	SMD_0006	807	66,79	141	3,00	152	4,34	1,45
-	SMD_3621	738	69,92	41	3,52	48	5,09	1,44
-	SMD_1530	501	57,29	111	2,56	118	3,70	1,44
-	SMD_3949	1320	69,62	15	2,37	12	3,42	1,44
-	SMD_2379	402	68,91	42	3,12	61	4,51	1,44
-	SMD_2923	204	68,14	154	2,78	115	4,00	1,44
actP	SMD_1960	2502	67,91	9	1,99	18	2,87	1,44
-	SMD_3334	801	67,17	23	2,08	24	3,00	1,44
folB	SMD_0377	354	63,84	125	2,57	151	3,70	1,44
-	SMD_2695	2109	68,28	26	4,63	16	6,66	1,44
-	SMD_3684	432	62,96	134	2,47	143	3,54	1,44
-	SMD_1470	1413	70,42	79	1,90	92	2,73	1,44
-	SMD_0136	687	66,67	71	2,77	156	3,98	1,44
smpB	SMD_1785	822	63,87	173	2,58	184	3,70	1,44
-	SMD_1507	633	65,09	55	3,38	67	4,85	1,43
-	SMD_0050	3933	58,73	37	3,92	38	5,62	1,43
-	SMD_0676	1299	64,74	143	2,62	155	3,75	1,43
dusA	SMD_0259	1032	71,41	94	3,16	118	4,52	1,43
tldD	SMD_3052	1446	69,29	95	2,53	59	3,62	1,43
-	SMD_2177	2034	53,79	112	2,92	112	4,18	1,43
-	SMD_0334	1128	72,07	48	2,63	46	3,76	1,43
nadD	SMD_3063	666	72,82	85	1,80	74	2,57	1,43
-	SMD_2758	243	65,02	315	1,92	269	2,75	1,43
-	SMD_2496	2130	67,89	78	2,72	41	3,88	1,43
ssuE2	SMD_0100	534	63,30	63	2,47	54	3,52	1,43
-	SMD_2891	924	66,99	51	3,30	37	4,71	1,43
scpA	SMD_2834	909	65,02	83	3,38	83	4,81	1,42
-	SMD_1935	816	69,85	45	3,03	54	4,32	1,42
trmB	SMD_3379	735	69,25	171	2,37	157	3,37	1,42
-	SMD_1092	534	68,16	54	2,34	33	3,33	1,42
-	SMD_3033	1584	67,17	26	3,05	23	4,33	1,42

-	SMD_1627	987	69,81	111	3,20	97	4,55	1,42
lexA	SMD_1677	636	67,14	263	3,98	242	5,66	1,42
-	SMD_3034	672	66,07	116	3,18	124	4,51	1,42
-	SMD_1721	1227	70,17	91	3,42	111	4,85	1,42
-	SMD_4027	531	65,54	113	2,71	72	3,84	1,42
phhA	SMD_0029	894	65,66	518	3,32	455	4,69	1,41
-	SMD_0239	660	67,12	51	3,41	32	4,82	1,41
fabG3	SMD_3551	741	67,88	154	1,63	178	2,31	1,41
selB	SMD_3451	1932	69,36	18	2,85	21	4,04	1,41
-	SMD_4229	1242	67,31	113	2,79	85	3,94	1,41
-	SMD_2729	543	65,38	79	3,37	87	4,76	1,41
-	SMD_4161	408	68,14	81	2,31	78	3,26	1,41
-	SMD_4066	774	64,34	39	3,44	26	4,86	1,41
modA	SMD_2433	744	70,03	42	2,26	36	3,19	1,41
Q	SMD_3270	1008	65,77	33	2,86	33	4,03	1,41
-	SMD_0522	1284	66,43	17	6,61	13	9,31	1,41
-	SMD_3074	684	66,81	111	3,02	86	4,25	1,41
-	SMD_1031	951	65,83	45	2,59	59	3,65	1,41
-	SMD_1675	501	67,47	114	2,60	129	3,66	1,41
-	SMD_2723	1125	70,13	31	2,82	37	3,97	1,41
-	SMD_3108	411	70,32	49	2,39	65	3,36	1,41
-	SMD_3824	900	69,78	18	3,05	29	4,28	1,40
ampDI	SMD_1393	567	68,96	119	3,52	111	4,94	1,40
-	SMD_1708	561	66,31	233	2,84	299	3,98	1,40
clpB	SMD_3336	2586	65,70	93	2,23	197	3,13	1,40
tesB	SMD_1185	921	69,27	111	2,71	111	3,79	1,40
tatD	SMD_4069	828	67,15	84	2,85	65	4,00	1,40
-	SMD_3587	1365	66,08	97	2,89	76	4,05	1,40
purE	SMD_1439	504	68,85	318	2,75	271	3,85	1,40
-	SMD_1186	777	70,01	32	2,02	31	2,82	1,40
glpQ	SMD_4234	1107	67,66	33	4,88	33	6,81	1,40
-	SMD_2099	1191	67,42	142	2,04	78	2,84	1,39
-	SMD_3672	603	65,34	134	2,80	164	3,91	1,39
-	SMD_3784	510	72,35	51	3,09	51	4,30	1,39
mltA	SMD_0108	837	69,53	45	4,01	44	5,58	1,39
-	SMD_2459	2265	70,46	58	2,11	58	2,94	1,39
-	SMD_2530	930	69,25	23	2,35	15	3,27	1,39
-	SMD_3590	972	71,19	28	3,22	25	4,48	1,39
-	SMD_1243	1188	68,94	33	2,46	24	3,42	1,39
-	SMD_4206	1461	71,87	24	3,40	18	4,72	1,39
kgdA	SMD_1725	660	68,64	45	1,70	69	2,37	1,39
katA	SMD_0296	1104	68,66	33	3,48	34	4,83	1,39
-	SMD_3368	678	65,49	129	3,69	159	5,12	1,39
-	SMD_1634	1623	65,25	74	2,34	87	3,25	1,39
-	SMD_3811	1164	67,10	14	3,68	16	5,11	1,39
-	SMD_3042	774	68,60	212	3,75	316	5,21	1,39
lgt	SMD_0693	888	66,67	114	2,78	115	3,85	1,39
-	SMD_3674	1008	67,96	54	3,45	44	4,77	1,38
-	SMD_0263	792	68,81	61	4,02	75	5,56	1,38
GCDH	SMD_0170	1164	67,01	71	2,98	61	4,12	1,38
-	SMD_2620	768	68,10	8	3,21	7	4,45	1,38
-	SMD_1222	795	68,18	58	2,66	58	3,67	1,38
-	SMD_2871	1329	65,91	69	2,06	48	2,84	1,38
-	SMD_3057	651	70,66	18	2,99	26	4,13	1,38
-	SMD_3028	2979	65,02	19	2,37	14	3,27	1,38
phaAB	SMD_3914	2829	67,90	34	2,76	31	3,80	1,38
rlpA	SMD_3639	1269	70,53	111	3,33	164	4,57	1,37
-	SMD_2569	507	66,47	41	4,44	42	6,08	1,37
ftsY	SMD_1743	1032	69,67	112	2,24	129	3,07	1,37
-	SMD_4228	1428	66,95	97	2,57	85	3,53	1,37
-	SMD_1650	219	66,21	87	4,51	115	6,19	1,37
-	SMD_0751	1695	70,62	91	2,64	127	3,61	1,37
-	SMD_1834	888	68,47	218	2,40	234	3,28	1,37
-	SMD_3881	426	68,54	146	2,59	159	3,54	1,37
nfi	SMD_1267	675	71,11	44	3,72	45	5,10	1,37

-	SMD_2478	1110	69,46	343	3,23	324	4,41	1,37
recQ	SMD_3255	1728	68,52	117	3,34	117	4,56	1,37
glmU	SMD_3694	1368	67,32	123	2,37	141	3,24	1,37
-	SMD_0213	1101	66,76	113	2,59	121	3,54	1,37
-	SMD_3525	774	65,12	11	2,64	11	3,61	1,37
rnG	SMD_3054	1488	65,79	131	2,66	127	3,63	1,36
-	SMD_4128	1422	70,96	113	3,45	115	4,70	1,36
D	SMD_3295	996	65,76	24	2,41	18	3,28	1,36
-	SMD_3091	252	69,44	211	3,54	159	4,82	1,36
-	SMD_3247	537	69,27	111	2,37	142	3,22	1,36
-	SMD_1744	300	71,33	61	2,25	121	3,06	1,36
-	SMD_2818	600	67,67	272	3,55	285	4,83	1,36
-	SMD_1643	1314	65,45	271	3,07	283	4,17	1,36
-	SMD_3380	1848	66,72	81	2,89	75	3,92	1,36
-	SMD_3506	1122	69,34	48	2,51	42	3,41	1,36
-	SMD_3369	462	64,72	155	2,72	134	3,69	1,36
-	SMD_0267	855	74,15	29	3,49	31	4,73	1,36
-	SMD_1142	1260	70,40	21	2,82	21	3,82	1,35
-	SMD_1705	507	70,41	88	1,82	111	2,47	1,35
-	SMD_1160	1755	67,29	64	2,99	54	4,05	1,35
plsB	SMD_0057	2628	67,73	162	3,06	159	4,14	1,35
pilQ	SMD_3424	1923	66,30	51	2,82	33	3,82	1,35
trxA	SMD_3857	330	63,94	491	1,93	556	2,62	1,35
-	SMD_0210	1446	70,68	41	2,53	33	3,42	1,35
-	SMD_2451	333	71,47	25	3,10	28	4,18	1,35
-	SMD_0519	990	66,97	161	2,29	183	3,09	1,35
-	SMD_0540	264	61,36	29	2,57	39	3,47	1,35
kdsC	SMD_1038	549	71,04	116	2,60	118	3,51	1,35
grxC	SMD_0854	291	68,04	115	2,27	142	3,06	1,35
kefB	SMD_4086	1779	68,52	33	2,81	31	3,79	1,35
-	SMD_0709	1068	69,38	97	2,08	85	2,80	1,35
-	SMD_1821	1566	68,20	25	2,18	34	2,93	1,35
-	SMD_0181	636	70,13	142	2,23	134	3,00	1,34
-	SMD_1252	1065	67,14	338	1,88	212	2,53	1,34
ctpA	SMD_0351	1485	67,74	135	3,33	195	4,47	1,34
-	SMD_0554	927	67,75	56	2,65	51	3,56	1,34
-	SMD_0730	582	72,34	96	3,50	113	4,70	1,34
tnpA	SMD_2147	2985	62,24	22	2,23	29	2,99	1,34
smeN	SMD_3391	3126	67,85	31	2,33	24	3,13	1,34
-	SMD_1527	348	62,93	114	2,50	118	3,35	1,34
-	SMD_0261	684	64,18	399	3,69	468	4,95	1,34
-	SMD_0739	498	64,66	256	3,59	244	4,80	1,34
nudE	SMD_3230	564	66,84	211	2,32	192	3,10	1,34
-	SMD_0156	975	64,62	96	3,09	89	4,13	1,34
-	SMD_3560	540	65,93	81	2,76	88	3,68	1,33
lhr	SMD_0075	2490	67,43	21	5,55	23	7,40	1,33
-	SMD_2889	1017	71,68	84	3,10	75	4,13	1,33
dnaG	SMD_0371	1740	67,47	119	2,76	112	3,67	1,33
-	SMD_3458	1227	66,91	65	2,59	56	3,44	1,33
-	SMD_1079	456	67,98	35	3,39	43	4,50	1,33
-	SMD_3141	390	65,38	45	2,42	35	3,22	1,33
-	SMD_0030	486	66,67	122	3,83	127	5,07	1,33
-	SMD_0764	2739	65,24	25	3,37	14	4,46	1,33
-	SMD_0938	705	68,94	61	2,33	54	3,09	1,33
ubiB	SMD_0182	1656	67,93	158	2,70	133	3,58	1,32
-	SMD_2815	480	71,25	32	2,60	33	3,44	1,32
-	SMD_0572	414	71,50	43	2,73	34	3,61	1,32
-	SMD_0370	501	70,46	27	2,85	25	3,77	1,32
-	SMD_3262	423	66,19	18	3,77	24	4,99	1,32
dxs	SMD_2922	1911	67,03	225	2,76	231	3,64	1,32
-	SMD_1622	834	68,35	31	3,56	29	4,71	1,32
-	SMD_2032	384	65,10	352	2,19	248	2,89	1,32
-	SMD_3228	840	67,86	115	2,24	133	2,96	1,32
-	SMD_3747	573	57,77	268	2,46	226	3,25	1,32
-	SMD_3065	501	66,87	263	2,23	254	2,94	1,32

-	SMD_0747	783	64,50	34	2,93	35	3,87	1,32
osmC	SMD_1086	432	66,44	66	2,74	91	3,61	1,32
-	SMD_0398	1515	65,68	196	2,43	216	3,21	1,32
-	SMD_1984	393	65,65	88	1,64	71	2,16	1,32
-	SMD_4106	798	68,92	81	3,04	62	4,00	1,32
-	SMD_2518	387	68,22	15	4,64	25	6,11	1,32
-	SMD_0653	585	67,86	38	2,87	37	3,78	1,32
mqo	SMD_1155	1689	65,78	26	3,30	19	4,35	1,32
-	SMD_0218	402	67,91	57	2,14	46	2,82	1,32
-	SMD_1422	1071	65,17	132	1,91	125	2,51	1,32
-	SMD_3705	366	67,49	312	4,66	497	6,13	1,31
-	SMD_4047	459	71,46	77	3,19	94	4,19	1,31
cyoD	SMD_1216	342	60,53	747	1,73	662	2,28	1,31
czcD	SMD_1566	549	63,21	61	3,28	41	4,30	1,31
-	SMD_3956	636	69,81	25	3,42	21	4,49	1,31
-	SMD_0310	705	68,51	196	3,53	133	4,63	1,31
-	SMD_0303	366	66,12	214	3,13	297	4,11	1,31
ispF	SMD_1658	498	68,88	117	1,79	115	2,34	1,31
-	SMD_3214	603	64,34	154	1,95	178	2,56	1,31
-	SMD_3483	5235	62,69	43	2,60	21	3,41	1,31
-	SMD_1245	1401	69,38	64	3,13	65	4,09	1,31
radA	SMD_1218	1377	69,57	54	3,50	55	4,57	1,31
fhuE	SMD_3599	2139	65,08	61	2,85	47	3,72	1,31
-	SMD_3306	666	68,77	111	2,61	118	3,41	1,30
-	SMD_1483	225	58,22	184	3,52	213	4,59	1,30
parB	SMD_4146	924	68,94	218	2,63	186	3,43	1,30
-	SMD_2735	663	63,20	116	2,17	121	2,82	1,30
-	SMD_3338	711	66,10	4	3,01	5	3,92	1,30
-	SMD_2225	2511	70,61	64	2,09	92	2,72	1,30
msrA2	SMD_0715	687	67,39	59	2,59	121	3,38	1,30
-	SMD_0329	663	67,42	317	2,13	328	2,78	1,30
-	SMD_3200	996	65,86	27	2,93	24	3,82	1,30
-	SMD_3500	2919	66,84	15	2,75	13	3,57	1,30
rpfN	SMD_2228	1254	65,87	36	1,93	53	2,51	1,30
-	SMD_3872	1332	67,79	37	3,05	53	3,97	1,30
-	SMD_3614	1101	68,21	47	2,46	49	3,20	1,30
-	SMD_1174	810	69,63	46	2,34	52	3,04	1,30
-	SMD_4165	1827	64,20	129	2,62	135	3,40	1,30
-	SMD_0734	330	68,79	12	3,54	19	4,60	1,30
ivd	SMD_0207	1164	66,49	197	2,27	197	2,95	1,30
pilB	SMD_3358	1731	65,11	73	3,09	56	4,01	1,30
-	SMD_1956	1023	70,09	62	2,31	58	2,99	1,30
-	SMD_1653	330	67,88	77	2,06	78	2,67	1,30
recC	SMD_4211	3342	70,08	51	2,76	56	3,57	1,30
-	SMD_1391	762	69,29	75	2,85	79	3,68	1,29
-	SMD_2623	657	64,54	58	1,96	69	2,53	1,29
trpA	SMD_2990	810	72,47	39	2,15	44	2,78	1,29
-	SMD_0498	792	67,93	112	3,24	88	4,19	1,29
-	SMD_3580	1149	68,32	17	3,10	16	4,01	1,29
-	SMD_0431	843	67,73	37	3,61	36	4,67	1,29
-	SMD_3212	849	67,26	175	4,21	194	5,44	1,29
-	SMD_1322	870	66,90	192	1,72	153	2,22	1,29
lspA	SMD_1198	525	65,33	187	1,82	239	2,35	1,29
-	SMD_4033	1299	71,13	34	2,55	26	3,28	1,29
-	SMD_3146	1650	66,24	28	2,60	41	3,35	1,29
-	SMD_0169	1686	67,08	82	2,64	88	3,40	1,29
infC	SMD_2951	480	58,96	2257	1,86	2298	2,39	1,29
-	SMD_0772	414	62,08	1751	2,65	1625	3,41	1,29
-	SMD_1120	966	66,56	29	2,72	31	3,50	1,29
ftsJ	SMD_1668	633	63,51	224	3,11	213	4,00	1,29
-	SMD_2687	378	64,02	44	3,51	77	4,51	1,28
-	SMD_0991	1098	64,03	38	2,79	53	3,58	1,28
-	SMD_2817	1767	72,10	53	3,29	53	4,22	1,28
-	SMD_4222	1008	69,64	9	2,75	8	3,53	1,28
-	SMD_0501	579	68,74	138	2,86	121	3,67	1,28

-	SMD_1257	360	66,11	284	2,47	154	3,17	1,28
-	SMD_0119	1296	71,06	48	2,90	51	3,72	1,28
-	SMD_0152	1791	52,88	76	2,77	75	3,55	1,28
-	SMD_1462	1344	68,45	21	2,75	22	3,52	1,28
-	SMD_2476	336	70,54	169	2,48	152	3,17	1,28
-	SMD_0541	846	55,79	185	2,05	212	2,62	1,28
-	SMD_3329	1866	68,44	157	3,13	118	4,01	1,28
fruA	SMD_2227	1707	67,37	28	2,04	59	2,61	1,28
mltB	SMD_3640	1122	67,74	234	3,42	315	4,36	1,28
-	SMD_4125	555	65,77	154	2,34	146	2,99	1,28
-	SMD_0091	630	67,30	78	3,57	111	4,55	1,28
-	SMD_3485	663	68,33	71	2,50	87	3,18	1,28
rodA	SMD_3641	1104	68,03	14	2,77	11	3,54	1,27
-	SMD_2897	633	64,61	132	2,78	136	3,54	1,27
-	SMD_1665	489	67,48	114	2,42	121	3,09	1,27
-	SMD_2003	1041	69,45	34	2,52	35	3,21	1,27
-	SMD_0095	435	69,89	115	1,90	88	2,42	1,27
-	SMD_1425	1113	66,94	27	2,51	26	3,20	1,27
-	SMD_3007	651	69,28	33	3,12	31	3,97	1,27
thiG	SMD_3378	795	67,92	242	2,61	211	3,32	1,27
-	SMD_3389	1509	63,75	61	2,59	61	3,29	1,27
-	SMD_0848	2940	65,48	49	3,05	53	3,88	1,27
-	SMD_3058	2796	65,67	63	3,34	112	4,24	1,27
-	SMD_3043	1335	66,14	51	3,32	36	4,22	1,27
dnaJ	SMD_1795	1125	66,67	217	1,93	427	2,45	1,27
-	SMD_2318	1089	68,32	55	3,40	55	4,33	1,27
-	SMD_1018	756	66,27	67	3,16	72	4,02	1,27
amn	SMD_3561	777	63,84	158	3,19	162	4,05	1,27
apt	SMD_1822	537	69,09	126	1,99	118	2,53	1,27
-	SMD_3177	1191	66,92	61	2,36	78	3,00	1,27
-	SMD_0516	2595	67,32	11	4,66	4	5,91	1,27
prfA	SMD_0749	1083	68,61	82	2,47	89	3,14	1,27
-	SMD_3660	993	67,67	54	2,83	61	3,59	1,27
-	SMD_3478	873	64,83	61	2,74	65	3,48	1,27
engC	SMD_3554	1167	68,81	111	3,55	98	4,49	1,27
-	SMD_0177	417	69,54	52	2,55	63	3,23	1,27
-	SMD_0311	390	62,56	168	4,76	162	6,04	1,27
-	SMD_1586	450	66,22	82	1,62	61	2,05	1,27
-	SMD_1081	543	63,17	38	3,99	51	5,05	1,27
-	SMD_2773	897	71,57	59	2,27	73	2,87	1,27
-	SMD_1832	1221	68,80	91	2,25	115	2,85	1,27
-	SMD_2899	930	69,46	48	2,26	46	2,86	1,27
-	SMD_3782	930	68,28	78	2,82	89	3,57	1,27
-	SMD_0417	753	59,63	38	2,50	31	3,16	1,27
-	SMD_2150	3480	65,46	39	2,50	17	3,16	1,27
lon	SMD_0872	2448	64,75	154	2,06	299	2,61	1,26
-	SMD_3655	423	62,41	85	2,43	114	3,07	1,26
-	SMD_4048	1800	68,17	71	3,12	64	3,95	1,26
ppx	SMD_0849	1497	68,14	87	4,15	95	5,24	1,26
lepB	SMD_3128	795	63,52	274	2,07	226	2,61	1,26
-	SMD_0941	1047	67,05	115	3,05	129	3,85	1,26
smeC	SMD_4021	1416	69,56	11	4,00	9	5,05	1,26
exoD	SMD_1642	660	67,58	141	2,76	139	3,48	1,26
purU	SMD_0222	858	67,48	111	3,19	91	4,03	1,26
-	SMD_2165	2238	68,28	31	2,51	29	3,17	1,26
-	SMD_0372	894	66,22	38	3,65	45	4,61	1,26
bcd	SMD_2751	1149	64,75	186	2,15	174	2,72	1,26
-	SMD_3669	1194	68,59	78	3,26	86	4,11	1,26
-	SMD_3011	1506	67,80	75	2,98	81	3,76	1,26
gst5	SMD_3746	642	68,22	114	2,97	63	3,74	1,26
-	SMD_0226	1050	66,76	96	4,68	117	5,89	1,26
-	SMD_3760	864	65,74	14	2,89	22	3,64	1,26
prmA	SMD_3838	921	69,27	84	3,20	77	4,03	1,26
nadC	SMD_1437	852	71,01	171	2,30	162	2,89	1,26
glnB2	SMD_2138	324	63,89	115	1,52	127	1,91	1,26

-	SMD_3476	603	66,83	49	2,73	51	3,44	1,26
rep	SMD_0046	1977	65,55	88	2,62	82	3,30	1,26
-	SMD_4190	546	68,13	48	2,46	24	3,10	1,26
clpA	SMD_2106	2286	65,62	84	2,67	142	3,35	1,26
-	SMD_3235	465	67,53	47	2,26	25	2,84	1,26
-	SMD_0189	528	66,86	45	3,22	47	4,04	1,26
ycgL	SMD_4088	261	64,37	196	4,60	192	5,78	1,26
pilP	SMD_3425	537	67,78	55	2,51	31	3,15	1,26
pgk	SMD_3403	1176	67,94	179	2,33	176	2,92	1,25
xpsD	SMD_0590	2208	67,48	45	2,26	41	2,84	1,25
-	SMD_2640	423	65,01	213	2,94	282	3,68	1,25
infA	SMD_2108	219	56,16	642	2,02	774	2,53	1,25
-	SMD_4029	993	69,08	47	2,81	48	3,52	1,25
-	SMD_0015	1092	64,01	49	6,39	51	8,01	1,25
-	SMD_2489	2628	68,04	5	3,51	6	4,39	1,25
fliP	SMD_2054	780	65,38	31	1,66	22	2,08	1,25
-	SMD_3120	696	68,10	61	2,50	88	3,13	1,25
-	SMD_1754	480	67,92	71	3,01	67	3,77	1,25
-	SMD_0705	585	68,72	139	3,43	145	4,29	1,25
-	SMD_0298	720	68,06	232	3,06	232	3,82	1,25
-	SMD_3373	1245	60,40	43	2,70	51	3,38	1,25
corC	SMD_1418	882	65,65	356	2,12	345	2,65	1,25
-	SMD_3267	429	63,87	46	6,94	39	8,67	1,25
prlC	SMD_3679	2031	67,06	28	2,31	54	2,89	1,25
xpsF	SMD_0581	1242	69,57	31	2,45	43	3,06	1,25
-	SMD_0166	639	66,51	48	3,99	54	4,97	1,25
-	SMD_0123	426	67,61	221	3,29	194	4,10	1,25
-	SMD_3039	1218	67,41	49	2,69	35	3,36	1,25
oprP	SMD_3543	1170	67,52	87	3,10	43	3,87	1,25
hflX	SMD_1674	1311	68,04	197	2,15	237	2,68	1,25
-	SMD_3682	516	66,28	145	2,58	111	3,21	1,25
motA	SMD_0478	855	65,61	79	3,33	51	4,15	1,25
-	SMD_3725	2532	68,44	31	4,53	13	5,65	1,25
-	SMD_2040	783	71,52	36	2,37	23	2,95	1,25
-	SMD_4097	603	68,16	45	2,18	58	2,71	1,24
-	SMD_1856	1182	70,56	35	2,00	36	2,48	1,24
-	SMD_0698	384	65,63	194	2,00	198	2,48	1,24
-	SMD_2880	1227	66,42	357	2,61	416	3,24	1,24
-	SMD_0211	858	69,11	18	2,82	16	3,51	1,24
-	SMD_3812	657	67,28	48	3,51	64	4,36	1,24
-	SMD_4107	744	72,85	51	3,49	46	4,34	1,24
-	SMD_0045	672	72,77	36	4,40	54	5,47	1,24
-	SMD_2339	1488	69,83	43	2,21	28	2,74	1,24
topA	SMD_3783	2493	65,66	161	2,47	168	3,06	1,24
-	SMD_0204	1167	70,27	59	2,03	37	2,51	1,24
rlmN	SMD_1850	1206	64,68	337	2,14	299	2,65	1,24
-	SMD_0472	1650	67,58	49	3,36	61	4,16	1,24
-	SMD_3409	801	67,29	133	2,68	132	3,31	1,24
trmE	SMD_4235	1350	69,85	31	3,03	31	3,75	1,24
-	SMD_4198	780	66,15	37	2,20	23	2,72	1,24
-	SMD_1847	2373	66,75	94	1,82	117	2,25	1,24
sdhC	SMD_1732	333	63,66	768	3,17	821	3,91	1,24
-	SMD_1202	708	67,80	48	2,27	39	2,81	1,24
-	SMD_1273	858	69,11	111	2,88	94	3,57	1,24
-	SMD_1143	1020	69,02	183	2,29	144	2,83	1,24
mrcA	SMD_3429	2424	68,56	79	3,08	87	3,81	1,23
-	SMD_0005	780	68,59	115	2,77	114	3,42	1,23
fliC2	SMD_2075	1179	67,35	719	2,47	436	3,05	1,23
-	SMD_2783	561	65,78	36	2,41	43	2,97	1,23
nreB	SMD_0144	1173	61,89	18	3,15	21	3,88	1,23
-	SMD_4156	666	67,42	119	3,99	118	4,92	1,23
-	SMD_1054	1026	68,03	67	2,28	61	2,80	1,23
aroC	SMD_3005	1104	69,38	171	2,58	159	3,18	1,23
rph	SMD_3455	726	70,11	98	2,52	82	3,10	1,23
-	SMD_3581	678	63,42	75	2,79	79	3,44	1,23

-	SMD_0843	1458	67,97	14	3,04	12	3,74	1,23
corA	SMD_1416	1023	63,93	77	2,12	68	2,61	1,23
hslU	SMD_3661	1374	65,14	124	2,53	269	3,12	1,23
dsbC	SMD_0574	786	68,45	129	3,38	117	4,16	1,23
cadC	SMD_3510	2325	69,25	111	3,28	128	4,03	1,23
ispA	SMD_3048	879	70,53	142	2,98	165	3,67	1,23
-	SMD_3382	282	69,15	174	4,52	268	5,56	1,23
hisB	SMD_1949	1074	66,11	25	1,43	17	1,76	1,23
-	SMD_3367	1329	65,09	76	2,82	82	3,46	1,23
-	SMD_0409	834	68,11	34	4,34	32	5,33	1,23
ribF	SMD_1196	948	69,20	134	2,56	141	3,14	1,23
-	SMD_3509	900	67,00	151	2,66	165	3,27	1,23
-	SMD_1358	444	66,67	21	2,58	79	3,17	1,23
-	SMD_1711	258	67,05	139	2,85	131	3,49	1,23
gshB	SMD_3242	948	68,99	84	3,80	91	4,67	1,23
aqpZ	SMD_3135	720	67,36	155	3,18	127	3,90	1,23
copB2	SMD_3258	996	67,17	13	3,44	4	4,22	1,23
pheS	SMD_2948	996	67,37	169	2,93	161	3,60	1,23
mdoD	SMD_0042	1605	67,60	115	3,06	113	3,75	1,23
-	SMD_3869	513	65,69	38	3,30	59	4,05	1,23
-	SMD_1927	825	65,70	91	2,04	71	2,51	1,23
tatC	SMD_4171	750	65,33	86	3,39	113	4,15	1,23
-	SMD_1159	1989	67,27	81	3,40	66	4,17	1,23
-	SMD_3020	345	66,38	31	4,53	26	5,54	1,23
-	SMD_3946	435	66,21	12	5,63	14	6,89	1,23
rluC	SMD_2822	930	68,92	87	2,45	81	3,00	1,23
ostA	SMD_0702	2448	65,89	215	2,24	242	2,75	1,22
-	SMD_2230	1038	65,41	9	2,40	12	2,94	1,22
hsp90xo	SMD_1219	1860	69,14	91	2,81	92	3,44	1,22
tctD	SMD_3548	690	67,54	71	2,98	74	3,65	1,22
chiA2	SMD_2957	1191	68,09	23	2,43	19	2,97	1,22
-	SMD_0447	1959	70,50	27	3,18	31	3,88	1,22
-	SMD_1075	1257	67,30	113	1,95	93	2,39	1,22
lipB	SMD_3635	711	70,46	294	3,20	354	3,91	1,22
smmP	SMD_4139	1206	71,64	36	3,30	32	4,03	1,22
-	SMD_0373	444	65,77	313	3,00	271	3,67	1,22
-	SMD_2879	1248	68,51	69	2,22	73	2,72	1,22
-	SMD_2884	705	72,91	75	2,11	61	2,57	1,22
-	SMD_3184	453	65,78	51	2,69	48	3,29	1,22
-	SMD_2457	1809	65,17	25	2,66	18	3,25	1,22
mrdA	SMD_3643	2076	69,12	54	2,14	51	2,61	1,22
-	SMD_1807	1497	69,07	83	2,28	125	2,79	1,22
-	SMD_3755	333	58,56	45	2,23	53	2,73	1,22
parE	SMD_1648	1890	65,34	231	2,00	212	2,44	1,22
gloB	SMD_0934	765	65,10	224	5,04	261	6,16	1,22
-	SMD_3097	594	67,34	78	2,92	86	3,57	1,22
-	SMD_0225	1410	68,16	21	4,54	21	5,55	1,22
pdhB	SMD_3931	1068	68,82	394	2,03	365	2,47	1,22
pcm2	SMD_3530	648	67,75	486	2,59	518	3,15	1,22
-	SMD_3653	261	65,13	21	2,26	16	2,75	1,22
-	SMD_3908	930	69,14	28	3,22	37	3,91	1,22
-	SMD_3882	888	69,71	76	3,70	72	4,50	1,22
ftsH	SMD_1669	1926	66,04	232	2,48	271	3,02	1,22
-	SMD_0485	567	67,90	145	2,42	151	2,95	1,22
-	SMD_3123	729	68,86	225	3,27	183	3,98	1,22
-	SMD_0886	1746	58,65	59	2,35	66	2,85	1,21
etfA	SMD_0542	939	67,73	287	2,56	345	3,11	1,21
smf	SMD_3779	1128	72,07	15	2,99	24	3,63	1,21
secG	SMD_2982	438	67,35	512	3,32	432	4,02	1,21
-	SMD_4120	1521	68,57	51	3,32	35	4,03	1,21
lctP	SMD_2546	1659	65,58	13	3,84	24	4,65	1,21
-	SMD_0257	594	67,85	177	3,47	165	4,21	1,21
-	SMD_3559	957	68,03	311	2,80	315	3,40	1,21
-	SMD_1311	816	69,49	57	3,28	52	3,98	1,21
-	SMD_3140	1251	66,03	67	2,83	33	3,43	1,21

-	SMD_3582	417	67,39	14	2,80	15	3,39	1,21
-	SMD_3623	744	66,80	16	2,66	22	3,23	1,21
-	SMD_2791	825	66,42	154	3,10	159	3,76	1,21
mscL	SMD_3384	405	63,46	171	2,45	151	2,96	1,21
hemK	SMD_0721	858	71,10	47	3,52	61	4,25	1,21
-	SMD_0512	1902	69,87	212	2,29	198	2,77	1,21
-	SMD_0840	780	68,72	111	2,78	118	3,36	1,21
-	SMD_0565	822	69,34	42	2,76	34	3,34	1,21
-	SMD_0537	792	64,77	5	3,91	3	4,74	1,21
-	SMD_2105	813	70,11	26	2,59	21	3,13	1,21
-	SMD_2661	399	65,41	11	2,58	7	3,11	1,21
-	SMD_3889	2241	68,90	49	3,41	51	4,12	1,21
-	SMD_2808	855	67,84	133	2,55	98	3,08	1,21
guaB	SMD_1859	1464	66,53	494	2,60	451	3,14	1,21
purK	SMD_1440	1149	69,54	251	2,51	221	3,03	1,21
engA	SMD_1855	1398	65,52	246	2,03	194	2,45	1,21
-	SMD_0282	933	67,63	76	3,00	68	3,62	1,21
-	SMD_3681	1113	66,58	97	2,84	96	3,42	1,21
-	SMD_0124	438	67,35	472	2,45	463	2,95	1,21
-	SMD_3307	702	66,95	96	2,53	78	3,05	1,20
kdsD	SMD_1039	1002	70,66	87	3,01	74	3,62	1,20
-	SMD_3468	480	62,92	94	3,30	79	3,98	1,20
otsA	SMD_3346	1365	67,25	24	2,87	28	3,46	1,20
-	SMD_3924	1113	62,80	227	2,72	241	3,27	1,20
fruK	SMD_2226	957	70,43	36	1,31	73	1,57	1,20
-	SMD_0244	1152	61,28	25	2,94	17	3,53	1,20
-	SMD_0684	687	64,77	239	2,76	348	3,31	1,20
-	SMD_1479	864	65,74	17	2,85	19	3,42	1,20
gltX	SMD_1279	1404	66,74	334	2,43	318	2,92	1,20
deaD	SMD_1296	1956	68,20	415	3,31	257	3,97	1,20
-	SMD_2685	492	68,70	66	3,05	73	3,66	1,20
-	SMD_3049	372	60,75	271	2,80	226	3,36	1,20
-	SMD_3220	1401	68,81	233	3,44	293	4,13	1,20
pilA	SMD_3359	420	58,33	743	2,53	291	3,04	1,20
-	SMD_4049	516	70,16	67	2,53	73	3,03	1,20
lpxD	SMD_1328	1023	69,11	226	2,24	239	2,68	1,20
-	SMD_1330	1359	68,29	126	2,12	126	2,54	1,20
virB9	SMD_2638	780	58,97	214	2,27	239	2,72	1,20
-	SMD_1224	1077	71,96	31	2,62	31	3,14	1,20
-	SMD_1583	1140	66,32	97	2,15	71	2,57	1,20
hmgA	SMD_3923	1299	67,28	117	2,13	125	2,56	1,20
-	SMD_0302	471	71,13	116	2,57	136	3,08	1,20
-	SMD_0733	453	71,96	34	3,98	45	4,76	1,20
-	SMD_0731	2253	69,02	124	2,89	115	3,46	1,20
mtnB	SMD_1972	669	66,22	77	2,54	79	3,03	1,20
-	SMD_2710	1431	62,12	44	2,61	36	3,12	1,20
thrB	SMD_1940	915	70,71	11	2,29	11	2,74	1,20
-	SMD_4177	2106	69,66	33	3,21	18	3,84	1,20
hydG	SMD_3357	1374	69,14	114	3,13	116	3,73	1,19
-	SMD_4083	2727	69,16	45	3,31	45	3,95	1,19
-	SMD_0823	1056	68,28	59	3,36	66	4,01	1,19
-	SMD_0844	1254	65,71	44	2,88	41	3,44	1,19
waaE	SMD_3772	807	69,27	53	3,19	51	3,81	1,19
mviM	SMD_0052	651	72,04	74	3,83	75	4,57	1,19
-	SMD_0079	2169	70,31	14	2,80	11	3,34	1,19
fadD	SMD_2017	1677	63,69	114	2,97	114	3,55	1,19
rumA	SMD_3122	1335	66,74	121	2,91	113	3,47	1,19
recO	SMD_3124	720	72,08	57	2,43	54	2,90	1,19
-	SMD_3901	762	69,03	112	3,04	115	3,62	1,19
-	SMD_0524	366	73,77	167	2,73	213	3,25	1,19
rpsO	SMD_2959	309	61,17	1611	2,45	1455	2,92	1,19
proP	SMD_2380	1491	66,60	32	3,65	35	4,35	1,19
smeK	SMD_3875	3123	66,70	21	3,10	21	3,69	1,19
-	SMD_0860	621	66,51	14	3,49	21	4,15	1,19
-	SMD_0520	669	69,66	92	2,91	113	3,46	1,19

-	SMD_0510	1353	70,51	53	2,99	42	3,56	1,19
rpoN2	SMD_2069	1410	70,50	61	3,18	44	3,78	1,19
pncA	SMD_0223	627	71,29	85	3,01	54	3,58	1,19
-	SMD_1187	321	68,54	53	2,48	42	2,96	1,19
-	SMD_3722	1920	69,06	29	3,59	29	4,27	1,19
ftsK	SMD_2113	2361	66,96	111	2,58	128	3,06	1,19
fabH2	SMD_0976	978	64,01	524	2,34	458	2,78	1,19
mraZ	SMD_0635	402	63,18	414	3,40	546	4,03	1,19
-	SMD_0474	543	65,93	133	3,15	138	3,74	1,19
-	SMD_3133	2064	68,65	45	2,96	61	3,51	1,19
-	SMD_0458	1650	67,58	85	2,24	87	2,66	1,19
smc	SMD_2725	3504	67,84	71	2,51	81	2,98	1,19
-	SMD_0061	867	68,74	45	3,38	41	4,01	1,19
clpX	SMD_0871	1290	62,87	597	2,34	623	2,78	1,19
-	SMD_3491	390	68,72	39	2,88	44	3,42	1,19
wbpO	SMD_1633	1287	66,90	121	2,65	141	3,15	1,19
-	SMD_2665	1326	64,03	28	2,34	25	2,77	1,19
-	SMD_0815	1611	71,32	38	3,30	37	3,91	1,19
-	SMD_3194	3207	69,66	19	2,99	11	3,54	1,19
ptrB	SMD_3668	2112	65,20	51	2,73	94	3,23	1,18
-	SMD_1060	609	67,49	43	2,82	62	3,35	1,18
-	SMD_2993	888	74,44	15	2,41	14	2,85	1,18
-	SMD_3056	738	68,43	62	3,71	67	4,39	1,18
-	SMD_3654	336	64,88	37	2,39	56	2,83	1,18
-	SMD_2298	1140	65,53	131	2,61	136	3,09	1,18
ispD	SMD_1657	699	70,39	154	2,14	143	2,54	1,18
-	SMD_1974	1476	66,19	95	2,39	98	2,83	1,18
-	SMD_3093	867	68,63	37	4,69	55	5,55	1,18
-	SMD_2236	1059	68,37	21	2,54	19	3,01	1,18
-	SMD_4162	1125	67,56	69	2,39	63	2,83	1,18
himA	SMD_2946	300	58,33	818	2,34	871	2,76	1,18
-	SMD_0221	942	69,75	42	3,34	41	3,95	1,18
rpoZ	SMD_3444	300	62,33	468	3,58	561	4,22	1,18
fabF	SMD_0981	1263	64,45	444	2,25	386	2,66	1,18
pbpC	SMD_3174	2382	70,49	14	2,13	12	2,51	1,18
-	SMD_0258	2415	67,54	113	2,82	111	3,32	1,18
ffh	SMD_1223	1377	66,01	219	2,71	218	3,20	1,18
ampN	SMD_0331	663	65,46	112	2,80	114	3,30	1,18
sufB	SMD_1078	1476	64,91	73	3,38	74	3,99	1,18
argH	SMD_2868	1296	68,52	66	2,04	57	2,40	1,18
-	SMD_2127	789	65,65	164	2,48	186	2,92	1,18
cbpA	SMD_3171	888	68,36	119	2,35	143	2,77	1,18
-	SMD_4192	891	68,46	97	2,64	116	3,11	1,18
prfB	SMD_2008	927	66,02	532	2,07	519	2,44	1,18
metF	SMD_0654	828	65,82	15	3,71	13	4,36	1,18
lspA2	SMD_2136	471	64,54	22	1,14	32	1,34	1,18
-	SMD_0727	798	68,92	72	2,46	85	2,90	1,18
hscC	SMD_4160	1689	67,38	67	3,20	69	3,76	1,18
gap	SMD_3406	1005	62,49	793	2,52	764	2,96	1,17
-	SMD_3648	621	69,57	97	2,64	96	3,10	1,17
-	SMD_3928	1500	64,33	267	2,20	254	2,59	1,17
-	SMD_1036	567	65,26	158	2,75	156	3,22	1,17
lpsI	SMD_0550	735	64,08	156	2,74	174	3,22	1,17
-	SMD_3321	522	68,58	234	2,23	229	2,61	1,17
-	SMD_2986	1146	68,41	138	2,30	121	2,70	1,17
-	SMD_4042	864	74,07	45	2,99	48	3,51	1,17
tdk	SMD_0043	621	66,83	85	3,93	81	4,60	1,17
cysQ	SMD_3229	804	67,41	161	2,82	157	3,31	1,17
-	SMD_3997	4698	62,62	17	3,65	11	4,28	1,17
ipk	SMD_0753	870	69,20	184	2,43	293	2,84	1,17
-	SMD_2933	234	64,10	85	5,09	83	5,95	1,17
-	SMD_1281	1155	67,45	27	2,53	25	2,95	1,17
-	SMD_1515	768	67,45	5	2,94	12	3,44	1,17
ribE	SMD_0615	627	66,19	63	3,56	53	4,17	1,17
fliC	SMD_2076	1185	66,33	633	2,56	363	2,99	1,17

-	SMD_1290	1182	67,17	91	3,47	98	4,05	1,17
dxr	SMD_1331	1191	69,94	166	2,01	151	2,35	1,17
-	SMD_3620	1185	69,11	65	3,28	81	3,83	1,17
-	SMD_1421	486	67,70	132	2,20	154	2,57	1,17
prfC	SMD_3205	1605	65,92	172	2,71	164	3,16	1,17
-	SMD_3617	2664	63,48	48	2,89	45	3,37	1,17
-	SMD_0047	357	70,59	9	3,43	4	4,01	1,17
-	SMD_3335	516	67,44	77	2,68	151	3,13	1,17
-	SMD_1578	1107	68,47	16	3,00	16	3,50	1,17
lctD	SMD_2544	1140	67,54	32	1,91	41	2,23	1,17
-	SMD_2995	702	73,79	14	2,22	11	2,59	1,17
pilT	SMD_1013	1038	63,01	61	3,66	54	4,27	1,17
dhaA	SMD_0176	891	66,67	99	3,06	71	3,57	1,17
-	SMD_1306	1833	65,25	39	2,48	36	2,89	1,16
rnr	SMD_1367	2460	67,24	161	2,20	163	2,56	1,16
-	SMD_3692	1353	66,59	41	2,95	44	3,44	1,16
-	SMD_2860	1206	68,49	34	3,20	31	3,72	1,16
trwI	SMD_2630	1122	58,65	185	1,68	215	1,95	1,16
-	SMD_0086	1632	67,03	63	2,27	56	2,64	1,16
-	SMD_0316	753	68,26	99	3,12	112	3,63	1,16
mrkC	SMD_0597	2586	67,17	341	2,55	281	2,96	1,16
-	SMD_1924	360	65,00	51	3,00	66	3,49	1,16
-	SMD_1392	903	71,21	49	2,63	59	3,05	1,16
atpC	SMD_3697	423	66,19	1152	1,87	1187	2,18	1,16
-	SMD_3121	486	66,05	69	2,97	77	3,45	1,16
hmgcL	SMD_1988	897	70,23	48	2,55	66	2,96	1,16
yaiO	SMD_0183	786	66,28	51	2,62	43	3,04	1,16
-	SMD_2768	426	63,38	71	2,53	87	2,93	1,16
-	SMD_1309	1809	66,00	217	2,13	175	2,48	1,16
-	SMD_0598	1038	65,41	418	2,11	334	2,45	1,16
ubiF	SMD_0707	1170	71,62	91	2,83	99	3,28	1,16
-	SMD_1236	603	70,48	43	2,85	52	3,31	1,16
proB	SMD_2866	1158	72,11	36	1,92	22	2,23	1,16
-	SMD_0299	333	65,47	76	3,69	67	4,28	1,16
-	SMD_2998	675	66,07	52	2,13	43	2,46	1,16
-	SMD_1162	1965	67,28	123	2,58	126	2,99	1,16
-	SMD_2193	2646	68,33	21	2,79	13	3,24	1,16
efp2	SMD_1986	567	63,67	369	2,13	377	2,47	1,16
-	SMD_3627	1818	67,60	78	3,16	111	3,65	1,16
rpfF	SMD_2016	873	66,78	31	3,36	31	3,90	1,16
-	SMD_3837	972	69,03	81	2,87	117	3,32	1,16
pabA	SMD_3905	588	65,82	75	2,98	82	3,45	1,16
polA	SMD_4122	2775	68,22	134	2,83	125	3,28	1,16
fur	SMD_1788	417	61,87	332	2,66	351	3,08	1,16
ung	SMD_3851	714	66,81	137	2,45	152	2,84	1,16
-	SMD_0454	1197	66,92	142	4,41	79	5,10	1,16
-	SMD_3243	885	71,86	56	3,01	62	3,48	1,16
-	SMD_3236	1332	72,15	54	2,33	23	2,69	1,15
-	SMD_0378	1272	67,30	73	2,91	81	3,36	1,15
-	SMD_1131	1128	68,17	48	3,54	34	4,09	1,15
ilvK	SMD_2012	1641	66,97	21	2,88	26	3,32	1,15
-	SMD_1827	462	67,53	66	2,68	61	3,09	1,15
-	SMD_3155	1368	67,84	58	3,13	45	3,61	1,15
-	SMD_0606	426	71,36	134	4,46	186	5,14	1,15
xpsN	SMD_0589	789	72,62	26	2,44	21	2,81	1,15
hsdR	SMD_1862	2766	56,80	92	2,72	85	3,13	1,15
-	SMD_2111	1584	59,91	29	2,86	38	3,29	1,15
lipA	SMD_3634	1011	66,37	329	2,89	378	3,33	1,15
-	SMD_3536	1116	71,15	82	3,56	94	4,09	1,15
mltD	SMD_0933	1143	71,39	141	4,61	157	5,30	1,15
mreC	SMD_3645	1146	72,60	71	2,71	81	3,11	1,15
suhB	SMD_2893	828	67,03	239	3,63	192	4,17	1,15
-	SMD_2743	945	69,31	62	2,90	77	3,33	1,15
tolR	SMD_3312	429	65,50	178	2,46	187	2,83	1,15
dnaA	SMD_0001	1332	64,64	216	3,13	221	3,60	1,15

wbiI	SMD_1844	1914	65,94	133	2,29	129	2,63	1,15
-	SMD_0856	1005	64,88	711	2,07	787	2,38	1,15
aroB	SMD_3189	1113	71,88	85	2,81	119	3,23	1,15
glyS	SMD_4166	2076	69,27	215	2,54	194	2,92	1,15
RRM2	SMD_0216	1020	60,69	411	2,17	415	2,50	1,15
lpsJ	SMD_0551	633	65,40	144	2,20	176	2,52	1,15
-	SMD_0084	1335	64,79	111	2,41	74	2,76	1,15
-	SMD_0992	567	63,67	46	3,74	56	4,29	1,15
-	SMD_1781	1242	66,18	26	2,62	37	3,00	1,15
selD	SMD_3453	1080	70,19	45	4,54	56	5,20	1,15
tyrS	SMD_0336	1212	65,51	171	2,70	191	3,10	1,15
-	SMD_1654	330	69,09	32	1,28	21	1,47	1,15
fliI	SMD_2061	1386	70,06	51	1,87	29	2,14	1,15
fdx	SMD_1749	282	62,77	286	2,33	313	2,67	1,15
-	SMD_1051	1170	66,67	118	2,90	135	3,32	1,15
rimM	SMD_1227	513	64,13	579	2,69	462	3,08	1,14
-	SMD_0419	486	61,73	36	1,37	33	1,57	1,14
-	SMD_3632	597	69,35	56	4,09	83	4,67	1,14
-	SMD_1724	648	66,51	124	2,48	111	2,83	1,14
-	SMD_0301	456	71,27	69	2,67	85	3,05	1,14
trmJ	SMD_2892	828	69,57	94	3,35	82	3,83	1,14
metG	SMD_3080	2082	67,68	155	2,47	154	2,83	1,14
-	SMD_0561	378	69,58	168	2,87	211	3,28	1,14
-	SMD_1293	1179	70,23	431	2,94	449	3,36	1,14
-	SMD_3144	1704	69,13	38	2,86	42	3,26	1,14
frr	SMD_1334	555	61,08	915	2,29	833	2,62	1,14
-	SMD_1977	1389	67,03	55	2,98	67	3,40	1,14
-	SMD_0882	321	61,37	116	3,13	132	3,57	1,14
-	SMD_4063	750	66,27	144	2,93	151	3,34	1,14
-	SMD_3332	738	68,16	27	3,08	27	3,51	1,14
piuC	SMD_1068	678	67,85	64	1,78	55	2,03	1,14
-	SMD_0077	675	68,44	74	2,72	66	3,10	1,14
-	SMD_0285	1335	66,29	41	2,94	32	3,35	1,14
-	SMD_0887	1542	56,68	74	2,33	68	2,65	1,14
-	SMD_3331	1737	67,18	94	3,44	111	3,92	1,14
ccmG	SMD_2848	618	66,50	137	2,19	156	2,49	1,14
dnaX	SMD_0964	2067	69,86	141	2,70	157	3,08	1,14
-	SMD_4212	789	69,33	245	3,80	246	4,33	1,14
sufA	SMD_2730	339	68,44	173	2,97	171	3,38	1,14
-	SMD_1730	1083	66,85	43	3,29	45	3,74	1,14
-	SMD_1180	1215	67,82	85	2,77	99	3,15	1,14
rpmB	SMD_4132	204	60,29	5661	3,31	4987	3,77	1,14
uvrA	SMD_1190	2994	66,73	125	2,50	137	2,84	1,14
-	SMD_3858	432	72,69	251	2,62	238	2,98	1,14
amiC	SMD_2901	1569	71,06	166	3,93	211	4,47	1,14
frzE	SMD_3237	6732	68,27	81	3,63	46	4,13	1,14
-	SMD_3470	447	65,77	131	1,92	142	2,18	1,14
hflC	SMD_3167	864	65,16	291	2,55	297	2,89	1,14
-	SMD_1787	393	64,63	352	3,27	354	3,71	1,14
aas	SMD_3878	1890	65,93	55	3,48	57	3,95	1,14
-	SMD_3774	1710	70,00	38	3,04	32	3,45	1,14
glyQ	SMD_4167	912	66,01	236	3,14	221	3,56	1,14
-	SMD_0961	1056	62,78	116	2,31	142	2,62	1,14
-	SMD_0942	777	69,11	133	2,86	159	3,25	1,14
-	SMD_2985	1878	68,74	53	3,35	54	3,80	1,14
-	SMD_3460	375	68,27	51	3,13	62	3,55	1,14
-	SMD_3696	2214	67,89	61	2,29	42	2,60	1,13
ftsW	SMD_0642	1326	65,84	219	2,04	213	2,31	1,13
-	SMD_1994	462	62,99	81	2,09	68	2,37	1,13
-	SMD_1885	840	65,48	11	2,40	11	2,73	1,13
-	SMD_2809	303	66,01	87	2,86	82	3,24	1,13
-	SMD_0609	450	69,33	36	2,72	32	3,08	1,13
katA2	SMD_1237	1644	65,15	41	3,18	51	3,60	1,13
-	SMD_0384	1302	67,74	16	2,99	21	3,39	1,13
-	SMD_1913	888	68,58	37	1,93	33	2,19	1,13

wzm	SMD_0529	795	57,74	317	2,06	311	2,34	1,13
-	SMD_1526	471	60,08	199	2,65	177	3,00	1,13
-	SMD_3636	279	64,52	614	4,11	786	4,65	1,13
smeZ	SMD_1983	3150	68,25	118	1,98	49	2,24	1,13
-	SMD_3265	465	67,74	31	4,11	14	4,65	1,13
-	SMD_0973	570	70,18	38	2,56	49	2,90	1,13
-	SMD_2477	1149	64,93	311	2,95	317	3,34	1,13
uvrD	SMD_4127	2193	68,17	194	3,11	175	3,51	1,13
rmlC	SMD_0546	558	63,26	569	1,97	577	2,23	1,13
-	SMD_3555	1299	68,75	134	3,62	151	4,09	1,13
psd	SMD_3008	843	67,26	51	2,48	52	2,81	1,13
-	SMD_3009	348	63,22	227	2,11	256	2,39	1,13
gidB	SMD_4148	639	72,61	92	4,21	73	4,76	1,13
-	SMD_1061	1605	69,78	55	2,48	54	2,80	1,13
-	SMD_1802	597	67,67	51	2,74	53	3,10	1,13
ccmB	SMD_2843	696	69,68	53	3,38	42	3,82	1,13
-	SMD_0314	888	69,59	51	3,19	27	3,61	1,13
-	SMD_1853	639	65,88	388	2,50	415	2,82	1,13
-	SMD_3879	288	68,06	314	3,03	336	3,41	1,13
aroG	SMD_3803	1074	66,67	133	4,69	134	5,29	1,13
yidC	SMD_4237	1716	65,33	351	2,76	299	3,11	1,13
serC	SMD_2682	1086	67,50	127	3,17	167	3,57	1,13
-	SMD_1532	594	59,09	54	2,65	57	2,98	1,13
-	SMD_3842	630	67,14	23	2,72	17	3,07	1,13
-	SMD_3175	4845	67,22	57	2,80	42	3,15	1,13
purH	SMD_3847	1584	67,99	132	2,86	121	3,22	1,13
-	SMD_0242	1329	63,88	23	3,31	14	3,72	1,13
-	SMD_2810	162	64,20	191	2,44	119	2,74	1,12
-	SMD_3014	1362	64,90	144	3,10	84	3,48	1,12
rfbD	SMD_0547	894	68,57	313	2,05	332	2,31	1,12
-	SMD_1071	339	72,86	52	2,98	56	3,35	1,12
cstA	SMD_3138	2082	65,56	36	2,94	32	3,30	1,12
uvrC	SMD_1471	1845	68,94	55	1,90	72	2,14	1,12
-	SMD_1852	855	71,93	163	2,64	164	2,97	1,12
-	SMD_1006	2022	65,68	55	2,78	47	3,13	1,12
bfr3	SMD_1355	489	63,80	141	2,41	193	2,71	1,12
htpX	SMD_2894	867	65,63	121	3,61	186	4,06	1,12
-	SMD_1973	1428	66,32	57	1,87	56	2,09	1,12
-	SMD_3088	1152	68,84	52	2,97	61	3,33	1,12
hrpA	SMD_3253	4083	68,72	57	2,87	61	3,22	1,12
-	SMD_3309	519	65,13	2463	2,14	1992	2,40	1,12
rdgC	SMD_0059	906	64,79	261	2,77	278	3,10	1,12
-	SMD_3147	1965	64,73	159	2,36	119	2,64	1,12
-	SMD_3223	495	60,20	378	2,60	431	2,91	1,12
-	SMD_2686	1005	70,15	33	4,76	41	5,32	1,12
carB	SMD_2000	3243	65,90	317	2,11	279	2,36	1,12
-	SMD_1666	375	66,67	151	2,13	121	2,38	1,12
ribA	SMD_3768	1113	70,44	39	3,01	72	3,37	1,12
virD4	SMD_2641	1680	62,62	74	3,11	81	3,48	1,12
-	SMD_2789	216	65,74	95	3,68	132	4,12	1,12
cheZ	SMD_2044	606	70,30	159	1,75	94	1,96	1,12
mutL	SMD_2900	1905	68,56	111	3,94	115	4,40	1,12
-	SMD_0812	2124	66,48	31	3,12	31	3,48	1,12
serA	SMD_1976	1242	66,67	79	2,80	91	3,12	1,12
-	SMD_3622	486	70,37	82	3,64	83	4,06	1,12
rnhA	SMD_0936	453	66,00	174	2,94	216	3,28	1,12
pilZ	SMD_0986	354	68,08	112	2,83	117	3,16	1,12
-	SMD_1007	1422	69,90	12	2,66	17	2,97	1,12
-	SMD_3252	525	67,05	63	2,75	85	3,07	1,12
sdhA	SMD_1734	1791	66,72	851	2,22	853	2,47	1,12
mutX	SMD_2610	489	66,26	95	2,85	99	3,18	1,11
leuC	SMD_3522	1419	69,34	13	2,59	18	2,89	1,11
-	SMD_0596	729	68,18	653	3,39	516	3,77	1,11
rlmI	SMD_4230	1170	64,79	426	2,97	419	3,31	1,11
-	SMD_2574	714	72,55	8	7,96	3	8,86	1,11

argC	SMD_2869	954	68,03	51	2,16	46	2,40	1,11
-	SMD_4164	3858	69,10	86	2,20	91	2,45	1,11
-	SMD_2890	2127	68,31	39	3,21	27	3,57	1,11
-	SMD_4077	906	69,54	33	7,60	32	8,45	1,11
atpB	SMD_3704	801	60,80	742	3,60	887	4,01	1,11
-	SMD_2671	2754	65,50	41	2,63	42	2,92	1,11
-	SMD_4169	747	72,02	38	3,62	55	4,03	1,11
-	SMD_3603	1986	69,99	57	3,59	68	4,00	1,11
mraY	SMD_0641	1086	64,73	212	1,90	217	2,11	1,11
gyrA	SMD_2717	2727	64,54	488	2,67	459	2,97	1,11
-	SMD_0128	666	65,77	41	4,28	42	4,76	1,11
-	SMD_2898	963	68,22	62	2,48	57	2,75	1,11
hemE	SMD_3191	1074	70,30	115	3,20	91	3,56	1,11
-	SMD_2374	1374	70,23	15	2,77	8	3,07	1,11
prpE	SMD_0821	1881	66,24	63	2,61	75	2,89	1,11
mltD	SMD_3010	1611	69,21	146	2,53	143	2,80	1,11
-	SMD_3019	2166	65,28	17	3,22	11	3,57	1,11
-	SMD_0117	867	68,51	126	3,62	129	4,01	1,11
-	SMD_3254	549	64,12	272	3,59	445	3,98	1,11
-	SMD_0456	687	67,39	719	2,03	325	2,25	1,11
lolA	SMD_2115	639	66,04	179	2,21	162	2,45	1,11
-	SMD_2621	438	67,81	78	2,70	74	2,99	1,11
-	SMD_0832	336	63,10	198	2,06	219	2,29	1,11
-	SMD_3341	894	66,78	38	2,74	37	3,04	1,11
-	SMD_2663	1323	64,55	74	2,56	75	2,84	1,11
-	SMD_0893	237	55,27	215	1,48	214	1,64	1,11
cadA	SMD_1565	2913	65,67	25	2,10	41	2,33	1,11
-	SMD_2271	696	69,68	55	2,86	53	3,16	1,11
dapF	SMD_3665	852	69,25	215	2,82	254	3,12	1,11
hslV	SMD_3662	552	66,67	95	2,68	229	2,96	1,11
-	SMD_3482	684	71,64	49	2,51	42	2,78	1,10
-	SMD_0834	696	64,94	243	3,36	214	3,72	1,10
-	SMD_3633	2178	67,17	159	3,07	188	3,39	1,10
crp	SMD_3900	666	66,82	399	3,11	425	3,44	1,10
-	SMD_3647	939	64,64	191	3,52	197	3,89	1,10
-	SMD_3922	543	71,82	18	2,12	21	2,35	1,10
cyoE	SMD_0367	894	64,77	125	2,78	124	3,07	1,10
pth	SMD_0757	579	65,63	282	2,28	275	2,52	1,10
-	SMD_3423	1026	70,96	81	3,77	111	4,16	1,10
priA	SMD_3793	2178	70,25	54	3,59	64	3,96	1,10
kbl	SMD_0833	1257	64,68	161	2,70	171	2,98	1,10
-	SMD_0445	777	70,14	23	3,50	24	3,86	1,10
-	SMD_2996	5388	67,48	42	2,36	24	2,60	1,10
smeP	SMD_3527	3162	65,91	33	2,85	33	3,14	1,10
nudH	SMD_3892	621	69,24	133	3,11	144	3,43	1,10
recN	SMD_1791	1662	70,10	71	2,89	75	3,19	1,10
-	SMD_3714	795	64,91	131	2,68	171	2,95	1,10
mfd	SMD_1271	3465	68,34	161	2,56	152	2,83	1,10
feoB	SMD_1992	1866	68,86	94	2,36	91	2,60	1,10
-	SMD_3539	3345	69,39	43	2,44	57	2,69	1,10
murG	SMD_0643	1089	71,17	137	2,09	149	2,30	1,10
tesA	SMD_0683	1314	69,94	27	3,67	31	4,05	1,10
-	SMD_1203	957	67,08	53	2,45	44	2,70	1,10
astC	SMD_3477	1227	68,70	212	2,84	251	3,13	1,10
pilW	SMD_1449	1170	67,44	41	3,09	29	3,41	1,10
fabH	SMD_0175	1017	64,70	413	3,31	356	3,64	1,10
lpxA	SMD_1326	792	67,05	314	2,22	314	2,44	1,10
dadA	SMD_0483	1305	69,04	227	3,52	257	3,87	1,10
-	SMD_3625	1512	66,87	117	2,70	149	2,98	1,10
-	SMD_1441	342	68,42	79	2,53	91	2,78	1,10
-	SMD_1979	1770	69,77	56	2,43	62	2,67	1,10
pgi	SMD_1719	1515	67,39	231	2,46	261	2,71	1,10
-	SMD_0286	906	69,09	51	4,93	54	5,43	1,10
yciL	SMD_2833	300	71,67	172	2,14	179	2,35	1,10
-	SMD_1963	3075	64,20	264	2,55	182	2,80	1,10

-	SMD_3481	819	67,64	47	2,89	51	3,18	1,10
potG	SMD_1412	1137	65,44	138	2,24	143	2,46	1,10
pilC	SMD_3360	1224	59,72	115	2,88	89	3,17	1,10
-	SMD_4046	249	69,08	71	2,81	68	3,08	1,10
-	SMD_1663	615	64,23	194	2,32	241	2,54	1,10
-	SMD_0534	1308	57,42	221	2,05	216	2,25	1,10
hel	SMD_0053	924	69,48	221	2,64	171	2,90	1,10
-	SMD_2475	564	71,81	52	3,11	49	3,41	1,10
ahpC	SMD_0720	564	62,59	735	4,72	896	5,18	1,10
-	SMD_1090	1353	66,22	178	2,05	191	2,25	1,10
-	SMD_0885	1749	56,03	172	2,16	163	2,37	1,10
-	SMD_0227	1893	68,78	17	7,05	15	7,73	1,10
-	SMD_0413	369	65,58	19	1,73	7	1,90	1,10
gcvT	SMD_3225	1113	68,01	241	2,47	222	2,70	1,10
-	SMD_3650	1815	65,29	215	2,45	212	2,69	1,10
-	SMD_1965	1050	69,33	129	2,12	112	2,32	1,10
-	SMD_3885	465	65,81	85	2,54	85	2,78	1,10
-	SMD_4201	1470	68,57	152	2,45	131	2,68	1,09
-	SMD_1073	540	69,07	137	2,87	156	3,14	1,09
smmJ2	SMD_2141	1545	64,92	91	1,86	124	2,03	1,09
-	SMD_1025	789	69,46	23	4,77	17	5,21	1,09
mraW	SMD_0636	969	68,63	211	3,05	292	3,34	1,09
batA	SMD_3420	1005	69,95	31	2,56	45	2,80	1,09
-	SMD_3250	1566	72,16	38	2,74	36	2,99	1,09
-	SMD_3366	864	65,74	39	2,89	48	3,16	1,09
-	SMD_4158	3459	68,83	24	2,59	23	2,83	1,09
-	SMD_0459	1260	68,10	12	2,58	14	2,82	1,09
lpxB	SMD_1325	1260	69,52	225	2,38	214	2,60	1,09
rnE	SMD_2823	3258	68,35	267	2,48	276	2,71	1,09
mutS	SMD_1238	2592	68,06	62	3,18	51	3,47	1,09
htpG	SMD_1745	1893	65,40	118	2,42	314	2,65	1,09
gst	SMD_0824	612	68,14	83	2,96	117	3,23	1,09
cdsA	SMD_1332	837	68,94	257	2,40	223	2,62	1,09
-	SMD_2631	741	57,89	251	1,75	319	1,91	1,09
-	SMD_4087	3339	73,47	115	3,10	116	3,38	1,09
-	SMD_2683	744	72,04	186	3,90	173	4,26	1,09
lpxH	SMD_0845	747	67,20	191	3,81	177	4,15	1,09
ribB	SMD_0616	1101	69,12	48	3,20	38	3,49	1,09
accD	SMD_2989	876	66,10	412	2,43	419	2,65	1,09
-	SMD_2765	1029	66,28	57	2,37	58	2,58	1,09
asnB	SMD_1352	1692	67,20	28	3,35	35	3,65	1,09
leuB	SMD_3524	1068	68,54	31	2,21	35	2,40	1,09
-	SMD_0085	1635	65,38	91	2,38	88	2,59	1,09
-	SMD_0531	1491	61,44	461	2,25	521	2,45	1,09
-	SMD_2114	1944	67,80	127	2,09	135	2,28	1,09
groES	SMD_3814	288	64,24	1217	3,46	4639	3,77	1,09
rpsI	SMD_3895	393	65,14	2971	2,40	2548	2,62	1,09
dnaE	SMD_1323	3591	66,86	159	2,40	142	2,61	1,09
nuoK	SMD_2970	306	61,44	872	1,92	841	2,09	1,09
lctR	SMD_2545	765	70,07	21	2,35	25	2,55	1,09
manA	SMD_0548	1404	66,67	193	2,12	219	2,30	1,09
murB	SMD_1914	1062	67,42	85	1,94	117	2,11	1,09
aspS	SMD_3323	1752	66,50	258	2,89	251	3,15	1,09
rpoD	SMD_3765	1854	62,24	658	2,69	592	2,92	1,09
nbaC	SMD_2736	531	65,35	72	2,06	85	2,24	1,09
-	SMD_3418	1716	70,86	26	2,62	29	2,84	1,09
cydC	SMD_2853	1680	70,83	98	2,16	94	2,35	1,09
-	SMD_0613	885	67,57	152	2,96	111	3,21	1,09
ex7L	SMD_2905	1332	69,82	68	2,79	64	3,03	1,09
-	SMD_3161	2124	67,00	131	3,05	52	3,31	1,09
surE	SMD_1661	807	66,29	282	2,99	311	3,24	1,08
-	SMD_0614	621	66,67	232	4,02	95	4,37	1,08
-	SMD_0007	1194	63,23	1134	2,96	1189	3,21	1,08
-	SMD_0766	1017	67,85	23	3,11	31	3,38	1,08
pheA	SMD_2681	1200	66,08	153	2,63	173	2,85	1,08

pdhA	SMD_3930	1083	65,65	511	2,31	482	2,50	1,08
guaA	SMD_1861	1566	64,56	148	2,02	134	2,19	1,08
sbcB	SMD_2739	1440	68,68	62	2,31	67	2,50	1,08
-	SMD_2994	1110	66,67	68	2,41	57	2,61	1,08
-	SMD_1801	1773	69,37	29	2,83	34	3,07	1,08
-	SMD_4101	771	68,09	62	2,76	53	2,99	1,08
phoA	SMD_3044	1707	68,72	87	2,31	82	2,51	1,08
speA	SMD_3951	1890	66,61	184	2,75	171	2,98	1,08
yjjV	SMD_2790	774	69,90	81	3,34	111	3,61	1,08
-	SMD_2637	1254	65,63	157	2,32	188	2,51	1,08
-	SMD_3083	348	64,66	376	2,84	384	3,07	1,08
-	SMD_2787	543	68,51	53	3,41	57	3,69	1,08
coaD	SMD_1747	510	67,65	243	2,86	231	3,10	1,08
-	SMD_0094	2058	68,80	61	3,52	65	3,80	1,08
glmM	SMD_2988	1362	68,80	279	2,46	286	2,66	1,08
grpE	SMD_1793	516	68,41	213	2,20	421	2,38	1,08
mrcB	SMD_3248	2439	69,25	95	2,89	111	3,12	1,08
-	SMD_1796	798	69,17	17	1,31	21	1,42	1,08
-	SMD_0205	1983	69,64	217	2,17	191	2,34	1,08
-	SMD_4144	966	67,70	83	3,65	83	3,95	1,08
-	SMD_0535	624	53,85	482	1,75	486	1,89	1,08
-	SMD_0410	798	68,80	27	3,62	27	3,91	1,08
ppk	SMD_0850	2058	67,10	36	3,03	51	3,27	1,08
yrbG	SMD_3920	960	70,52	37	3,06	32	3,30	1,08
-	SMD_2531	1509	66,14	17	2,90	19	3,13	1,08
-	SMD_1814	2070	68,60	67	2,62	71	2,83	1,08
ppc	SMD_0666	2736	69,41	29	2,96	35	3,20	1,08
eno	SMD_1655	1293	64,35	383	2,60	418	2,81	1,08
aspC	SMD_0018	1203	66,58	229	2,92	254	3,16	1,08
-	SMD_4215	669	66,67	313	2,63	294	2,84	1,08
ahpF	SMD_0719	1593	66,79	55	3,58	79	3,86	1,08
-	SMD_3514	1719	65,79	22	3,65	18	3,94	1,08
leuS	SMD_3066	2694	66,96	366	2,86	344	3,08	1,08
-	SMD_0710	561	68,09	78	2,57	114	2,77	1,08
kdsA	SMD_1652	831	65,58	212	2,64	243	2,85	1,08
nuoB	SMD_2979	549	65,03	793	2,73	763	2,95	1,08
-	SMD_0836	2166	66,30	215	2,26	159	2,43	1,08
-	SMD_2541	297	67,34	255	2,64	327	2,84	1,08
pyrH	SMD_1335	729	67,63	291	2,49	313	2,68	1,08
recA	SMD_1678	1038	63,39	534	2,71	564	2,91	1,08
dld	SMD_2543	1701	67,72	29	1,93	36	2,08	1,08
-	SMD_0208	618	71,68	23	2,41	22	2,59	1,08
-	SMD_1015	984	68,39	28	3,04	22	3,27	1,08
-	SMD_2021	1551	66,34	14	2,07	11	2,22	1,08
glnD	SMD_1346	2628	69,25	118	2,44	116	2,63	1,08
aroH	SMD_0813	1392	66,31	82	3,43	89	3,68	1,08
RRM1	SMD_0215	2403	64,25	451	2,87	433	3,09	1,08
-	SMD_4036	573	68,76	412	3,14	414	3,37	1,07
-	SMD_0016	711	64,14	111	3,05	81	3,28	1,07
holA	SMD_3064	1038	71,19	137	2,99	139	3,22	1,07
-	SMD_2737	1275	69,10	66	2,64	75	2,83	1,07
-	SMD_0255	768	67,84	11	2,81	5	3,02	1,07
-	SMD_0093	618	69,42	37	3,94	56	4,23	1,07
pheT	SMD_2947	2382	70,53	161	2,51	149	2,69	1,07
-	SMD_3319	732	65,30	479	2,51	486	2,69	1,07
emrB	SMD_1361	1488	65,26	62	2,49	156	2,67	1,07
-	SMD_0563	504	74,60	111	3,76	113	4,04	1,07
-	SMD_3385	1422	69,55	79	2,96	83	3,18	1,07
-	SMD_4017	633	68,09	68	3,14	74	3,37	1,07
rbfA	SMD_2961	384	65,10	198	2,23	171	2,39	1,07
-	SMD_2829	1605	64,80	35	2,52	38	2,71	1,07
tolB	SMD_3310	1320	66,82	278	2,36	435	2,53	1,07
nuoL	SMD_2969	2175	62,94	927	1,80	811	1,92	1,07
-	SMD_2812	1566	64,94	175	2,45	164	2,63	1,07
rubA	SMD_3472	192	66,15	111	2,96	127	3,17	1,07

rsmE	SMD_3232	738	69,78	82	2,57	76	2,76	1,07
-	SMD_0333	231	70,13	76	3,44	48	3,68	1,07
kup	SMD_3316	1920	65,52	75	2,47	66	2,65	1,07
-	SMD_3773	1257	69,21	134	3,20	157	3,43	1,07
-	SMD_1740	450	66,22	41	2,70	41	2,89	1,07
cheA	SMD_2036	1989	68,63	132	2,30	73	2,47	1,07
rplM	SMD_3896	429	62,94	4361	2,33	3541	2,49	1,07
rplT	SMD_2949	360	61,94	3739	2,29	3367	2,45	1,07
-	SMD_4236	2673	67,15	33	3,17	28	3,39	1,07
-	SMD_1221	450	67,56	89	2,19	82	2,34	1,07
secA	SMD_0651	2733	65,02	296	2,60	357	2,78	1,07
folP	SMD_1671	897	68,90	74	2,49	81	2,67	1,07
glnA	SMD_0103	1410	63,19	39	3,71	86	3,97	1,07
-	SMD_0097	1035	68,99	68	3,53	78	3,78	1,07
potI	SMD_1414	843	67,02	87	2,44	64	2,61	1,07
pncB	SMD_0593	1182	65,65	113	3,59	111	3,84	1,07
rhlE	SMD_0517	1434	70,08	455	5,70	228	6,09	1,07
nuoA	SMD_2980	357	57,70	1124	3,35	939	3,58	1,07
rsuA	SMD_0490	702	67,66	149	2,79	134	2,98	1,07
-	SMD_4030	1617	70,62	45	2,59	38	2,77	1,07
valS	SMD_0566	2829	66,42	211	2,33	212	2,49	1,07
-	SMD_4193	1248	71,63	46	4,03	53	4,30	1,07
-	SMD_1256	384	67,45	187	2,87	78	3,06	1,07
-	SMD_2909	2295	64,92	211	2,83	117	3,02	1,07
-	SMD_4141	1773	66,61	69	3,34	59	3,56	1,07
-	SMD_1040	231	69,70	219	2,56	241	2,73	1,07
xpsH	SMD_0583	495	72,32	32	2,71	26	2,89	1,07
tktA	SMD_3416	1998	67,02	378	2,36	328	2,52	1,06
prs	SMD_0755	963	65,32	366	3,48	447	3,70	1,06
-	SMD_3208	411	64,96	81	2,67	99	2,84	1,06
-	SMD_3383	717	69,87	45	3,16	61	3,36	1,06
-	SMD_3045	540	69,81	61	1,60	58	1,70	1,06
wxocA	SMD_0532	3369	63,40	513	2,09	516	2,23	1,06
zupT	SMD_2821	810	64,20	51	2,65	53	2,82	1,06
folC	SMD_0838	1272	71,38	76	2,80	67	2,97	1,06
ssb	SMD_1108	576	66,67	398	2,47	376	2,62	1,06
-	SMD_2142	2325	66,71	114	2,16	154	2,30	1,06
trpS	SMD_0280	1296	66,98	246	3,12	211	3,32	1,06
nadE	SMD_3353	1635	68,44	131	2,85	135	3,02	1,06
-	SMD_2759	1350	65,63	595	2,26	565	2,40	1,06
-	SMD_1784	432	68,06	67	4,04	72	4,29	1,06
-	SMD_3758	2262	60,52	14	3,35	14	3,56	1,06
maeB	SMD_3541	2292	65,40	541	2,55	546	2,70	1,06
lpdA	SMD_3708	1803	67,05	341	2,40	357	2,54	1,06
fliA	SMD_2046	747	68,54	96	2,23	58	2,37	1,06
panE	SMD_3787	777	65,89	67	3,10	61	3,29	1,06
folD	SMD_1858	879	70,53	91	2,76	92	2,93	1,06
pyrC	SMD_2882	1347	66,74	119	2,61	162	2,77	1,06
mreB	SMD_3646	1047	67,05	219	3,21	238	3,40	1,06
ftsQ	SMD_0646	750	68,00	215	1,89	233	2,01	1,06
ftsB	SMD_1656	354	65,25	448	2,46	465	2,61	1,06
-	SMD_0251	579	64,08	16	2,61	7	2,77	1,06
fabG	SMD_0979	744	65,73	639	2,72	588	2,89	1,06
-	SMD_1158	636	69,03	231	6,17	247	6,54	1,06
-	SMD_3959	435	62,99	41	3,10	25	3,28	1,06
thyA	SMD_0694	795	65,03	161	2,72	174	2,88	1,06
rmlA	SMD_0545	888	64,30	551	2,19	542	2,32	1,06
-	SMD_1751	837	64,99	68	2,60	155	2,75	1,06
-	SMD_1980	1077	68,99	34	2,44	41	2,59	1,06
acnA	SMD_3180	2619	66,93	116	2,13	85	2,26	1,06
sdhD	SMD_1733	387	67,18	594	2,73	571	2,90	1,06
cydD	SMD_2854	1746	71,71	97	3,17	98	3,36	1,06
gpmA	SMD_1268	750	67,47	237	3,09	199	3,27	1,06
iunH	SMD_3440	939	67,20	193	2,74	213	2,90	1,06
bioF	SMD_4013	1224	71,49	66	3,06	53	3,24	1,06

-	SMD_3626	441	63,95	171	2,87	223	3,03	1,06
-	SMD_3051	588	69,05	194	2,29	132	2,42	1,06
phaE	SMD_3917	507	65,48	54	2,50	44	2,64	1,06
hflK	SMD_3168	1134	68,78	298	3,06	338	3,24	1,06
pyrE	SMD_0330	660	67,27	371	2,35	365	2,49	1,06
purA	SMD_3164	1293	66,13	362	3,36	337	3,56	1,06
-	SMD_2346	2286	65,40	16	2,73	22	2,89	1,06
-	SMD_0350	1302	70,89	92	3,75	86	3,97	1,06
-	SMD_1204	2799	69,85	15	2,93	19	3,10	1,06
-	SMD_3932	354	66,10	416	2,07	412	2,18	1,06
ccmF	SMD_2847	1920	69,06	44	2,49	53	2,63	1,06
pilX	SMD_1450	522	65,71	63	2,33	42	2,46	1,06
-	SMD_4133	1215	69,38	21	4,38	16	4,63	1,06
nudF	SMD_0728	555	67,03	84	2,71	84	2,86	1,06
rpsG	SMD_0781	474	61,18	4212	2,04	3711	2,16	1,06
rmuC	SMD_4220	1569	66,99	111	2,71	92	2,86	1,06
wzt	SMD_0530	1422	60,55	361	2,20	361	2,32	1,06
dnaK	SMD_1794	1923	64,27	471	2,37	1188	2,50	1,06
-	SMD_3143	714	69,19	116	3,60	113	3,80	1,05
cutC	SMD_1958	732	72,27	31	2,08	28	2,19	1,05
-	SMD_0859	759	67,98	21	3,98	21	4,20	1,05
smmI	SMD_1831	3087	65,21	7	2,59	11	2,73	1,05
minD	SMD_1167	810	65,19	558	2,35	614	2,48	1,05
-	SMD_1113	297	64,31	6	1,62	14	1,70	1,05
-	SMD_2825	3168	67,17	41	2,65	35	2,79	1,05
smeI	SMD_3873	1245	67,87	59	3,84	48	4,04	1,05
metH	SMD_2754	2685	66,74	24	2,42	31	2,55	1,05
-	SMD_0031	1038	70,04	71	5,03	73	5,29	1,05
-	SMD_1172	1074	62,57	1246	2,47	1213	2,60	1,05
rho	SMD_3859	1335	65,32	531	2,51	461	2,64	1,05
yceG	SMD_0983	1062	68,93	61	2,94	61	3,09	1,05
uup	SMD_1826	1884	66,24	145	2,65	143	2,78	1,05
-	SMD_3069	1626	71,46	63	4,70	58	4,94	1,05
murJ	SMD_1195	1605	67,98	57	2,64	53	2,78	1,05
-	SMD_0078	1809	72,64	18	3,00	18	3,15	1,05
rluF2	SMD_3101	699	70,96	76	3,45	64	3,62	1,05
-	SMD_1258	837	68,10	314	2,47	149	2,59	1,05
sppA	SMD_3789	1911	68,24	131	3,09	139	3,24	1,05
thrS	SMD_2952	1902	64,46	546	2,66	516	2,79	1,05
metX	SMD_2851	1113	68,01	119	3,27	117	3,43	1,05
truA	SMD_3000	771	68,61	98	2,29	86	2,40	1,05
-	SMD_3343	2436	66,58	13	3,26	14	3,42	1,05
-	SMD_3193	3507	68,55	47	2,79	42	2,93	1,05
fliC3	SMD_2077	1218	67,16	216	3,50	116	3,67	1,05
-	SMD_2713	990	67,07	61	2,56	73	2,68	1,05
-	SMD_1559	825	64,73	11	3,08	13	3,23	1,05
surA	SMD_0701	1347	67,19	513	2,59	494	2,72	1,05
proS	SMD_0559	1704	67,55	225	3,00	242	3,15	1,05
groEL	SMD_3813	1650	64,30	1879	3,64	6422	3,82	1,05
purF	SMD_0841	1467	66,39	187	2,77	212	2,90	1,05
-	SMD_1200	1884	66,35	36	2,33	39	2,44	1,05
fabG5	SMD_4091	714	67,23	117	2,15	78	2,25	1,05
yajC	SMD_1810	345	62,32	651	2,24	553	2,34	1,05
hpt	SMD_3118	555	67,39	241	2,40	225	2,51	1,05
-	SMD_1397	1638	66,97	14	3,12	9	3,27	1,05
-	SMD_3244	714	70,59	84	3,18	98	3,33	1,05
-	SMD_3630	906	68,32	77	2,62	118	2,74	1,05
parC	SMD_1356	2244	66,67	256	2,44	267	2,55	1,05
purM	SMD_1049	1059	68,84	198	3,48	219	3,64	1,05
secY	SMD_0805	1332	64,41	776	2,26	682	2,36	1,05
-	SMD_3938	927	67,31	55	3,05	69	3,19	1,05
cusB2	SMD_2140	1176	66,84	118	1,83	157	1,91	1,04
nuoD	SMD_2977	1308	63,38	924	2,19	846	2,29	1,04
rpmG	SMD_4131	165	53,94	1911	3,79	1773	3,96	1,04
glnS	SMD_0713	1749	66,50	179	3,04	217	3,17	1,04

acsA	SMD_4155	1944	66,56	149	3,48	162	3,63	1,04
dapA	SMD_1709	894	70,58	185	3,31	227	3,46	1,04
aceE2	SMD_0412	2688	64,29	893	2,30	881	2,40	1,04
tsf	SMD_1337	876	64,95	1874	2,52	1727	2,63	1,04
-	SMD_1987	771	67,19	144	2,95	161	3,08	1,04
-	SMD_1805	2796	68,17	36	2,29	32	2,39	1,04
lpxO	SMD_3953	909	65,57	119	3,33	112	3,48	1,04
pefL	SMD_0587	1146	70,77	34	2,51	41	2,62	1,04
fabF2	SMD_4090	1239	68,28	81	2,11	63	2,20	1,04
-	SMD_2187	423	59,10	33	2,88	43	3,00	1,04
exbB	SMD_0009	762	64,57	2956	2,10	2945	2,19	1,04
trpC	SMD_3903	795	69,18	121	3,25	123	3,39	1,04
-	SMD_0829	1092	63,92	11314	3,24	14418	3,37	1,04
-	SMD_0407	486	63,37	171	2,74	187	2,85	1,04
accC	SMD_3835	1368	65,42	695	1,94	631	2,02	1,04
-	SMD_1647	594	69,19	61	2,73	45	2,84	1,04
murF	SMD_0640	1395	69,68	147	2,18	175	2,27	1,04
ftsI	SMD_0638	1845	68,29	154	2,81	177	2,93	1,04
motB	SMD_0477	951	66,98	82	3,23	37	3,36	1,04
hns	SMD_0558	384	65,63	369	2,82	435	2,93	1,04
rpmF	SMD_0975	195	64,10	1769	2,54	1694	2,64	1,04
-	SMD_2224	984	67,17	172	2,73	195	2,84	1,04
lptB	SMD_1035	720	66,25	186	2,50	213	2,60	1,04
rplK	SMD_0774	429	62,70	4817	2,31	4275	2,40	1,04
nusA	SMD_2963	1512	65,94	618	2,81	512	2,92	1,04
-	SMD_1964	2466	69,02	131	2,32	115	2,41	1,04
-	SMD_0256	876	67,47	34	3,73	24	3,87	1,04
-	SMD_4057	1176	68,03	26	2,66	16	2,76	1,04
greA	SMD_2001	459	63,83	474	2,21	438	2,29	1,04
tolC	SMD_3531	1359	67,40	524	2,63	559	2,73	1,04
hisS	SMD_1943	1398	66,09	217	2,63	213	2,73	1,04
-	SMD_3619	393	67,43	42	2,18	39	2,27	1,04
-	SMD_4216	312	71,15	123	2,59	121	2,69	1,04
-	SMD_3404	1377	70,37	57	2,61	47	2,71	1,04
smeF	SMD_3656	1386	69,05	233	2,39	298	2,48	1,04
rpiA	SMD_3469	648	66,36	153	2,14	196	2,22	1,04
-	SMD_0397	1365	69,01	58	2,91	65	3,01	1,04
rplY	SMD_0756	618	63,27	2792	2,72	2524	2,83	1,04
panD	SMD_1718	381	61,94	491	2,62	561	2,72	1,04
xthA3	SMD_4151	768	65,89	217	3,26	169	3,38	1,04
potH	SMD_1413	933	63,24	89	2,38	81	2,47	1,04
moeA	SMD_2439	1254	70,81	28	2,52	32	2,61	1,04
dcd	SMD_3025	573	64,22	211	3,05	217	3,16	1,04
wecB	SMD_0599	1176	69,05	21	3,01	13	3,12	1,04
recB	SMD_4210	3678	69,44	48	2,31	59	2,39	1,03
-	SMD_3884	393	65,39	199	4,13	221	4,27	1,03
uvrB	SMD_1455	2025	64,40	66	2,71	62	2,80	1,03
infB	SMD_2962	2646	66,29	668	2,16	589	2,23	1,03
sphB	SMD_3105	2646	70,18	26	2,78	22	2,87	1,03
acn	SMD_2022	2754	66,16	75	2,49	113	2,57	1,03
ihfB	SMD_1840	306	63,07	321	2,41	295	2,49	1,03
-	SMD_1400	879	67,69	117	2,89	99	2,99	1,03
-	SMD_3719	732	69,13	42	2,78	43	2,87	1,03
-	SMD_0312	729	68,04	19	5,16	14	5,34	1,03
-	SMD_0051	525	66,48	29	3,79	26	3,92	1,03
rnk	SMD_0717	405	66,42	231	2,53	214	2,61	1,03
tig	SMD_0869	1296	66,98	614	2,85	574	2,94	1,03
-	SMD_1738	1242	65,70	129	2,57	141	2,66	1,03
-	SMD_1063	804	66,67	167	2,35	161	2,43	1,03
rpsP	SMD_1226	261	65,52	2878	2,64	2596	2,72	1,03
StmPr1	SMD_0741	1701	69,37	26	3,19	29	3,29	1,03
-	SMD_2143	1932	67,86	77	2,63	112	2,71	1,03
phoQ	SMD_0262	1425	68,70	92	3,61	119	3,72	1,03
-	SMD_0503	483	67,70	214	4,04	237	4,17	1,03
-	SMD_1164	1326	65,84	52	2,50	61	2,58	1,03

-	SMD_1854	1194	68,09	383	2,31	352	2,39	1,03
alaS	SMD_1680	2649	67,16	234	2,73	247	2,81	1,03
fliK	SMD_2059	1170	73,16	28	2,06	17	2,12	1,03
-	SMD_2163	2400	67,71	32	2,32	31	2,39	1,03
-	SMD_3511	672	65,18	24	3,45	22	3,55	1,03
lysA	SMD_1089	2595	68,09	158	2,79	176	2,87	1,03
sodA	SMD_2814	612	62,42	711	2,98	1298	3,06	1,03
-	SMD_2481	525	70,86	46	2,48	61	2,55	1,03
carA	SMD_1999	1128	66,49	295	2,78	297	2,86	1,03
-	SMD_1508	366	63,11	42	2,91	48	2,99	1,03
cspA	SMD_1819	210	61,43	3113	1,76	2514	1,81	1,03
upp	SMD_1755	633	68,72	123	2,87	133	2,95	1,03
murE	SMD_0639	1497	70,61	184	2,33	215	2,40	1,03
-	SMD_1926	3114	68,24	74	2,42	56	2,48	1,03
-	SMD_0083	708	65,11	43	2,38	41	2,45	1,03
nagA	SMD_3612	1149	69,45	43	2,15	47	2,20	1,03
ppiD	SMD_0879	1956	66,51	255	2,72	285	2,80	1,03
galU	SMD_1845	804	64,80	294	2,19	311	2,25	1,03
hemC	SMD_3718	912	71,16	69	4,76	81	4,89	1,03
rtcB	SMD_4076	1221	67,90	32	7,95	21	8,16	1,03
rsmB	SMD_3775	1332	71,02	118	2,88	76	2,96	1,03
-	SMD_0202	2490	68,63	41	3,08	21	3,16	1,03
folE	SMD_4202	612	66,83	355	2,94	292	3,02	1,03
-	SMD_3213	2877	63,68	68	4,04	94	4,14	1,03
-	SMD_1929	1134	67,11	94	2,34	81	2,40	1,02
-	SMD_0220	1101	71,39	72	3,50	53	3,59	1,02
clpP	SMD_0870	591	64,47	459	2,56	487	2,62	1,02
-	SMD_0605	1665	64,26	421	2,99	366	3,07	1,02
-	SMD_0677	972	69,86	58	2,91	52	2,98	1,02
-	SMD_3492	504	68,65	49	3,71	65	3,80	1,02
-	SMD_1390	702	65,24	977	2,43	1131	2,49	1,02
recG	SMD_3441	2112	69,65	147	2,51	161	2,57	1,02
-	SMD_3695	351	67,24	57	2,36	38	2,42	1,02
-	SMD_3856	369	66,12	68	3,95	56	4,04	1,02
-	SMD_0088	3687	66,97	122	2,82	111	2,89	1,02
gyrB	SMD_0004	2460	64,39	334	2,60	345	2,66	1,02
aroA	SMD_2680	1308	68,88	61	2,59	79	2,65	1,02
fumC	SMD_2769	1383	67,75	49	3,05	71	3,12	1,02
rluB2	SMD_0831	723	69,71	92	2,93	116	2,99	1,02
-	SMD_1163	2079	66,28	178	2,41	217	2,46	1,02
ilvE	SMD_0740	1095	67,31	216	2,75	237	2,81	1,02
rplO	SMD_0804	444	66,22	4698	2,06	3848	2,11	1,02
dcp	SMD_0192	2157	65,60	198	3,06	216	3,12	1,02
-	SMD_2340	1422	69,69	36	2,08	21	2,12	1,02
-	SMD_1030	885	67,91	46	2,65	63	2,71	1,02
-	SMD_3308	819	67,40	611	3,02	591	3,09	1,02
-	SMD_2137	1209	65,34	63	1,86	81	1,90	1,02
-	SMD_2635	984	64,74	159	2,21	195	2,25	1,02
-	SMD_4191	771	69,00	71	2,86	73	2,91	1,02
nadR	SMD_0355	1074	67,78	18	2,34	17	2,39	1,02
cgb	SMD_2554	2796	68,03	34	2,48	42	2,53	1,02
-	SMD_2528	654	67,89	11	2,39	12	2,44	1,02
hsdM	SMD_1863	1683	58,29	135	2,22	118	2,26	1,02
gst6	SMD_4051	690	66,23	42	3,97	47	4,04	1,02
gph	SMD_0499	669	66,67	148	3,64	136	3,71	1,02
-	SMD_4103	552	69,20	136	2,76	116	2,81	1,02
rlmB	SMD_1369	744	68,68	131	2,74	126	2,79	1,02
hemF	SMD_4119	897	68,78	63	3,50	52	3,56	1,02
ispH	SMD_1199	951	66,88	249	2,20	254	2,24	1,02
-	SMD_2830	3081	63,16	262	2,20	281	2,24	1,02
-	SMD_1130	1512	67,00	29	2,78	28	2,83	1,02
-	SMD_3399	1143	67,28	363	2,46	273	2,51	1,02
flgH	SMD_2082	693	67,97	62	1,94	34	1,97	1,02
secD	SMD_1811	1857	65,75	363	2,49	325	2,53	1,02
putA	SMD_0356	3219	68,97	251	2,30	231	2,34	1,02

fabA	SMD_0493	516	66,28	451	2,19	389	2,23	1,02
-	SMD_0612	378	61,90	111	3,05	89	3,10	1,02
-	SMD_4056	711	62,59	13	2,40	13	2,44	1,02
-	SMD_1348	867	70,59	69	2,34	77	2,38	1,02
fliD	SMD_2074	1416	63,91	154	2,24	91	2,27	1,02
rsmC	SMD_0489	1065	72,49	82	3,86	73	3,92	1,02
-	SMD_0746	576	67,01	92	2,81	121	2,85	1,02
-	SMD_2793	495	66,06	211	2,64	238	2,68	1,02
-	SMD_3099	558	67,74	121	3,40	131	3,45	1,02
-	SMD_3902	675	69,48	115	3,38	119	3,43	1,02
purL	SMD_0576	3921	69,73	216	2,55	168	2,59	1,01
-	SMD_1640	996	70,08	17	3,24	23	3,28	1,01
rplA	SMD_0775	699	65,38	3854	2,32	3357	2,35	1,01
accA	SMD_1321	960	66,67	437	2,20	353	2,24	1,01
nuoG	SMD_2974	2235	68,32	934	2,02	842	2,05	1,01
-	SMD_0685	1272	69,10	45	3,52	55	3,57	1,01
tolA	SMD_3311	1056	69,41	144	2,49	161	2,52	1,01
spgM	SMD_0549	1347	65,78	271	2,47	281	2,51	1,01
pefK	SMD_0586	864	67,94	34	2,71	34	2,74	1,01
-	SMD_0317	897	73,36	155	2,82	131	2,86	1,01
-	SMD_3486	1947	69,03	111	3,63	116	3,68	1,01
-	SMD_2135	273	62,64	57	2,40	62	2,43	1,01
-	SMD_3004	1017	69,22	181	2,69	172	2,72	1,01
virB4	SMD_2632	2442	59,79	217	1,87	281	1,89	1,01
-	SMD_1957	996	67,97	52	2,41	52	2,44	1,01
-	SMD_2634	405	57,53	893	2,90	952	2,93	1,01
dfp	SMD_0321	1290	69,61	116	3,37	95	3,41	1,01
-	SMD_3711	636	64,94	1354	3,74	1343	3,79	1,01
-	SMD_2461	384	68,75	64	2,24	67	2,26	1,01
-	SMD_3031	2007	67,91	294	2,37	232	2,39	1,01
algC	SMD_0323	2286	70,47	65	2,59	55	2,61	1,01
exbD	SMD_0010	426	59,15	2412	2,18	2234	2,20	1,01
-	SMD_0484	1068	68,35	221	2,96	248	2,99	1,01
sodC	SMD_0114	570	69,65	141	2,79	179	2,82	1,01
-	SMD_0681	936	64,96	32	1,95	32	1,97	1,01
rluA	SMD_0820	615	67,80	29	3,18	31	3,21	1,01
-	SMD_2867	264	64,02	79	1,83	74	1,85	1,01
-	SMD_1189	408	63,73	214	2,29	223	2,31	1,01
-	SMD_0658	585	66,15	99	2,96	81	2,99	1,01
-	SMD_1269	2094	65,57	211	2,18	151	2,20	1,01
-	SMD_1813	1290	64,65	351	2,50	345	2,52	1,01
htrB	SMD_3534	918	66,45	121	3,69	123	3,72	1,01
pykA	SMD_3401	1467	66,67	111	3,12	138	3,14	1,01
ndk	SMD_1849	426	64,79	1254	2,49	971	2,51	1,01
serS	SMD_2677	1281	67,76	213	3,04	218	3,07	1,01
dsbA2	SMD_3594	834	70,02	181	2,39	182	2,41	1,01
pps	SMD_2613	2379	64,99	546	2,94	577	2,96	1,01
rnt	SMD_1375	585	65,98	181	3,51	151	3,54	1,01
recJ	SMD_2004	1758	69,68	73	3,06	64	3,08	1,01
hsdS	SMD_1864	1623	50,03	138	1,84	126	1,85	1,01
-	SMD_1370	1440	69,72	18	2,72	14	2,73	1,01
-	SMD_2744	843	68,09	47	2,76	44	2,78	1,01
-	SMD_3413	558	65,77	11	6,38	8	6,42	1,01
fabD	SMD_0978	894	71,59	416	3,06	394	3,08	1,01
-	SMD_0508	720	67,78	25	2,53	25	2,55	1,01
-	SMD_0526	237	58,65	124	11,67	211	11,74	1,01
rlmL	SMD_1395	2139	68,40	81	3,00	54	3,02	1,01
hemL	SMD_3474	1290	68,76	111	2,90	131	2,92	1,01
dnaN	SMD_0002	1101	63,67	314	2,40	392	2,42	1,01
-	SMD_0667	507	66,47	113	3,29	97	3,31	1,01
-	SMD_3349	771	74,06	82	2,71	79	2,73	1,01
betA	SMD_2018	1683	67,32	26	2,84	31	2,85	1,01
tex	SMD_2599	2388	69,05	94	2,71	115	2,72	1,01
-	SMD_0500	1842	67,64	119	3,03	71	3,05	1,00
secF	SMD_1812	981	64,02	522	2,10	475	2,11	1,00

-	SMD_3818	564	69,86	24	2,71	33	2,72	1,00
hfq	SMD_1673	276	60,14	331	2,54	411	2,54	1,00
asnC	SMD_2731	1395	65,30	466	2,87	417	2,88	1,00
-	SMD_0842	825	69,45	66	2,55	71	2,56	1,00
acnB	SMD_2025	2592	66,20	411	2,27	521	2,28	1,00
-	SMD_4068	885	69,94	51	3,72	41	3,73	1,00
rpsB	SMD_1338	807	64,06	2411	2,78	2216	2,78	1,00
mdh	SMD_0819	987	65,45	981	2,41	1227	2,42	1,00
argG	SMD_2873	1197	65,66	156	2,25	126	2,26	1,00
dgkA	SMD_0687	396	68,43	89	2,90	119	2,90	1,00
sucD	SMD_3354	876	64,16	2126	1,94	1812	1,94	1,00
-	SMD_3457	597	67,00	113	2,89	111	2,89	1,00
rpoC	SMD_0779	4224	64,49	1262	2,11	1179	2,11	1,00
fliM	SMD_2057	1005	64,78	81	2,20	52	2,21	1,00
thrC	SMD_1941	1287	69,15	21	2,96	21	2,96	1,00
-	SMD_0214	837	68,82	72	2,67	62	2,68	1,00
-	SMD_4199	3381	67,35	218	2,23	191	2,23	1,00
murA	SMD_1041	1272	66,67	237	2,88	265	2,88	1,00
scrK	SMD_1967	996	69,88	116	2,04	93	2,04	1,00
ccmE	SMD_2846	462	68,61	54	2,69	71	2,69	1,00
-	SMD_4157	576	63,89	19	3,31	21	3,30	1,00
pilY1	SMD_1451	3753	63,87	68	2,14	42	2,14	1,00
rpoN	SMD_1034	1422	68,21	88	2,64	112	2,64	1,00
-	SMD_1165	480	67,71	219	2,32	235	2,32	1,00
-	SMD_2704	1161	66,67	47	2,99	38	2,99	1,00
pnp	SMD_2958	2109	63,58	1564	2,22	1456	2,22	1,00
mutY	SMD_1742	1125	69,07	49	2,95	61	2,95	1,00
orn	SMD_2617	573	65,62	126	3,33	128	3,33	1,00
fabZ	SMD_1327	459	63,62	512	2,46	464	2,46	1,00
nlpD	SMD_1664	759	70,36	228	2,24	219	2,23	1,00
-	SMD_3664	675	68,74	181	2,55	256	2,55	1,00
fdnG	SMD_3446	3069	64,84	24	5,60	37	5,59	1,00
proC	SMD_1011	822	69,59	59	2,66	62	2,66	1,00
-	SMD_0304	1434	68,55	113	3,69	141	3,68	1,00
slyD	SMD_3040	483	63,77	564	2,44	422	2,43	1,00
lysS	SMD_2013	1512	65,34	358	2,67	414	2,66	1,00
-	SMD_1329	2364	63,62	426	2,20	425	2,19	1,00
-	SMD_2997	2019	65,82	98	2,37	71	2,37	1,00
ddlB	SMD_0645	963	68,85	261	2,00	299	2,00	1,00
-	SMD_0972	1836	67,54	58	3,61	59	3,60	1,00
-	SMD_2495	921	67,43	155	2,91	156	2,90	1,00
sodC2	SMD_0115	627	69,54	161	2,64	195	2,63	1,00
mrdB	SMD_3642	1113	67,21	79	2,46	73	2,45	1,00
-	SMD_2002	918	69,83	147	2,23	113	2,22	1,00
-	SMD_2121	1047	69,44	7	1,98	8	1,97	1,00
parA	SMD_4147	798	67,79	235	3,18	234	3,17	1,00
ppiB	SMD_0818	492	64,02	1136	2,54	1236	2,53	0,99
-	SMD_0853	1647	70,07	37	3,35	56	3,33	0,99
-	SMD_2719	732	68,03	44	3,06	64	3,05	0,99
atpH	SMD_3701	528	71,21	826	2,52	773	2,51	0,99
rpsL	SMD_0780	375	64,80	3112	2,14	2674	2,13	0,99
prpD	SMD_3176	1461	67,83	19	3,32	23	3,30	0,99
-	SMD_2733	315	68,57	149	3,27	138	3,26	0,99
rpmD	SMD_0803	168	61,90	5631	2,10	4493	2,08	0,99
fabB	SMD_0492	1209	67,66	386	2,33	354	2,32	0,99
RL35	SMD_2950	198	60,61	4625	2,45	4212	2,43	0,99
minC	SMD_1168	783	69,73	214	3,02	259	3,00	0,99
rpoB	SMD_0778	4155	64,16	1117	2,35	1117	2,33	0,99
nahB	SMD_3618	2358	67,22	24	3,45	25	3,42	0,99
atpG	SMD_3699	864	61,69	1139	2,05	1126	2,03	0,99
gpsA	SMD_0126	1026	70,57	411	2,41	461	2,40	0,99
mgtE	SMD_1026	1362	67,47	42	3,52	41	3,50	0,99
-	SMD_0726	1419	70,54	13	3,16	12	3,13	0,99
rluD	SMD_3350	990	68,69	151	2,52	155	2,50	0,99
-	SMD_4078	456	70,39	12	7,78	11	7,72	0,99

-	SMD_4096	2355	70,40	49	2,45	43	2,43	0,99
estB	SMD_3721	660	68,94	99	3,76	117	3,72	0,99
-	SMD_0230	1167	67,27	244	2,58	314	2,56	0,99
ecnA	SMD_0277	138	60,14	315	2,75	371	2,73	0,99
rpsA	SMD_1839	1686	61,92	4422	2,06	3878	2,04	0,99
rpsT	SMD_1194	270	65,19	2173	2,50	1887	2,47	0,99
-	SMD_0467	2112	67,71	121	2,68	154	2,65	0,99
-	SMD_2484	327	67,28	61	3,05	53	3,02	0,99
purC	SMD_3912	927	67,10	69	4,62	91	4,58	0,99
-	SMD_0008	666	66,82	931	2,75	1136	2,73	0,99
-	SMD_1220	2307	69,44	113	2,26	111	2,24	0,99
prpC	SMD_3181	1158	64,25	289	2,62	275	2,60	0,99
-	SMD_1285	414	68,60	21	2,45	16	2,42	0,99
-	SMD_0825	459	66,67	77	4,26	146	4,22	0,99
-	SMD_1085	399	63,41	1714	2,60	2166	2,57	0,99
pilH	SMD_3170	372	66,13	127	2,65	112	2,62	0,99
rpoA	SMD_0809	999	63,36	3883	2,32	3351	2,29	0,99
-	SMD_1050	1140	70,96	111	3,82	115	3,78	0,99
oxyR	SMD_0718	963	68,22	53	3,56	66	3,52	0,99
rbsK	SMD_0675	966	69,88	231	2,59	215	2,56	0,99
scpB	SMD_2835	876	68,04	122	3,17	95	3,14	0,99
rfbB	SMD_0544	1056	63,92	553	2,35	523	2,32	0,99
cusA3	SMD_2133	3162	66,26	8	2,42	8	2,39	0,99
-	SMD_0457	1632	68,87	286	2,33	137	2,30	0,99
sucC	SMD_3355	1170	62,14	2413	1,95	2142	1,92	0,99
-	SMD_3408	819	69,47	138	3,35	121	3,30	0,99
-	SMD_3926	1140	72,54	21	4,40	18	4,34	0,99
ggt	SMD_1750	1731	67,59	136	2,38	131	2,35	0,99
cydB	SMD_2856	1152	66,41	862	1,91	821	1,88	0,99
recD	SMD_4209	1881	71,50	35	2,84	36	2,80	0,99
-	SMD_1024	492	62,20	65	3,19	77	3,15	0,99
rpfC	SMD_2015	2178	67,72	71	2,63	58	2,59	0,99
nuoC	SMD_2978	750	66,13	767	2,59	689	2,56	0,99
-	SMD_3303	1197	62,91	18	2,92	18	2,88	0,99
-	SMD_0174	318	69,81	128	4,67	148	4,60	0,99
-	SMD_0742	495	62,83	228	3,38	287	3,33	0,99
adk	SMD_3489	549	67,76	438	2,47	642	2,43	0,99
talB	SMD_0716	957	68,65	215	2,49	218	2,46	0,99
argS	SMD_0318	1644	67,88	286	3,27	258	3,23	0,99
rpsM	SMD_0806	357	63,31	4182	2,08	3798	2,05	0,98
hnsB	SMD_3749	375	64,80	84	1,75	79	1,72	0,98
yfcB	SMD_3006	930	64,73	261	3,03	244	2,98	0,98
queA	SMD_1808	1068	69,38	61	3,11	55	3,06	0,98
murC	SMD_0644	1437	69,10	292	2,05	311	2,01	0,98
-	SMD_3562	3390	68,17	36	3,16	16	3,11	0,98
-	SMD_0935	576	71,88	124	3,43	156	3,37	0,98
lpxC	SMD_0649	912	64,69	311	2,18	353	2,14	0,98
rplQ	SMD_0810	384	65,89	1993	2,09	1791	2,05	0,98
-	SMD_3735	3351	68,04	51	2,59	51	2,55	0,98
-	SMD_0665	2517	68,97	83	2,19	81	2,15	0,98
-	SMD_1438	270	68,15	268	2,96	275	2,90	0,98
-	SMD_1415	1365	67,84	41	2,62	37	2,57	0,98
rluB	SMD_2836	1638	71,43	184	2,52	141	2,48	0,98
gtrB	SMD_3209	1020	66,27	233	3,37	228	3,31	0,98
nuoJ	SMD_2971	657	65,75	724	2,12	672	2,08	0,98
-	SMD_4034	660	69,39	117	2,87	142	2,82	0,98
-	SMD_1761	696	65,23	223	2,64	176	2,59	0,98
lepA	SMD_3129	1809	64,07	327	2,92	277	2,86	0,98
-	SMD_2831	1038	66,86	96	2,83	122	2,78	0,98
dapB	SMD_1998	672	72,32	83	2,37	112	2,32	0,98
rpsR	SMD_2727	231	60,17	3514	2,54	2974	2,49	0,98
rplF	SMD_0800	525	63,62	4712	2,49	3611	2,44	0,98
-	SMD_4093	1185	70,46	74	2,34	71	2,29	0,98
lpxK	SMD_1467	1020	69,51	111	2,10	89	2,06	0,98
pgsA	SMD_1472	627	66,35	189	2,31	174	2,27	0,98

purN	SMD_1045	660	71,06	37	3,46	36	3,38	0,98
-	SMD_0034	840	70,24	88	2,72	99	2,66	0,98
-	SMD_0814	1092	67,86	7	3,91	8	3,83	0,98
-	SMD_2766	888	69,03	44	2,31	42	2,26	0,98
-	SMD_2606	1080	69,91	71	2,54	73	2,48	0,98
-	SMD_2491	381	62,20	93	3,30	81	3,23	0,98
-	SMD_0283	1653	68,97	151	3,06	148	2,99	0,98
-	SMD_3624	324	69,75	45	3,08	54	3,01	0,98
mpl	SMD_3488	1365	68,42	127	3,26	151	3,18	0,98
-	SMD_3419	1839	69,49	25	2,52	31	2,46	0,98
-	SMD_1985	693	65,51	218	2,34	237	2,29	0,98
relA	SMD_3251	2034	69,32	93	3,03	117	2,96	0,98
-	SMD_2746	120	51,67	13	1,99	22	1,94	0,98
edd	SMD_1726	1917	70,06	41	2,33	56	2,28	0,98
-	SMD_3305	957	66,35	14	3,83	12	3,74	0,98
-	SMD_1182	561	70,41	15	4,58	19	4,47	0,98
rplS	SMD_1229	402	62,94	2123	2,88	2229	2,81	0,98
-	SMD_2788	846	65,84	353	3,46	411	3,38	0,98
gidA	SMD_4020	1890	69,74	119	3,74	94	3,65	0,98
pcnB	SMD_1714	1386	67,32	175	2,72	175	2,65	0,98
rpoH	SMD_3850	876	64,84	287	4,52	422	4,41	0,98
-	SMD_0881	366	60,93	112	3,77	131	3,68	0,97
-	SMD_2129	1059	66,19	44	3,51	117	3,42	0,97
uppS	SMD_1333	711	70,46	238	2,49	198	2,42	0,97
ftsE	SMD_3854	687	70,01	192	3,34	199	3,25	0,97
-	SMD_3792	1005	72,84	119	4,14	115	4,03	0,97
-	SMD_4053	1014	72,29	41	4,02	37	3,91	0,97
cspA2	SMD_2794	210	60,00	468	2,57	423	2,50	0,97
-	SMD_3716	603	72,97	31	4,61	32	4,49	0,97
-	SMD_2745	4908	66,61	681	2,20	588	2,14	0,97
accB	SMD_3834	480	66,88	667	2,20	517	2,14	0,97
-	SMD_3127	384	58,33	261	2,66	181	2,58	0,97
-	SMD_0400	561	68,45	44	3,70	41	3,59	0,97
-	SMD_3001	363	66,39	145	2,52	141	2,45	0,97
cheA2	SMD_2043	1845	69,38	81	2,36	52	2,29	0,97
rpsE	SMD_0802	543	64,46	4189	2,19	3532	2,13	0,97
typA	SMD_0817	1848	63,10	815	2,57	591	2,50	0,97
sdhB	SMD_1735	786	62,85	732	1,97	788	1,92	0,97
ychF	SMD_0758	1092	65,66	333	2,40	281	2,33	0,97
-	SMD_4075	1125	67,73	43	10,69	37	10,37	0,97
nuoM	SMD_2968	1509	64,41	665	1,91	621	1,85	0,97
fadL	SMD_0353	1428	66,67	239	2,37	232	2,30	0,97
pyrD	SMD_1915	1056	69,51	72	2,73	87	2,65	0,97
nuoH	SMD_2973	1095	62,19	911	2,06	819	2,00	0,97
argE	SMD_2872	1089	68,60	92	2,68	74	2,60	0,97
-	SMD_0994	2010	66,82	29	2,58	26	2,50	0,97
-	SMD_1445	3201	62,01	938	2,16	568	2,09	0,97
-	SMD_0592	840	68,57	81	3,19	99	3,08	0,97
thiE	SMD_3473	627	71,93	43	2,72	41	2,63	0,97
sucA	SMD_2774	2832	65,36	738	2,38	757	2,31	0,97
rplL	SMD_0777	369	62,06	7262	3,03	6211	2,93	0,97
-	SMD_3508	2850	64,46	1697	3,00	1748	2,90	0,97
-	SMD_3421	450	70,00	27	3,39	41	3,28	0,97
nuoN	SMD_2967	1464	66,67	582	1,98	536	1,91	0,97
-	SMD_3165	840	71,31	41	3,03	41	2,93	0,97
-	SMD_4099	1530	69,28	58	2,15	52	2,08	0,97
dapE	SMD_1350	1119	69,62	73	2,42	79	2,34	0,97
-	SMD_0837	645	69,46	118	2,79	111	2,70	0,97
-	SMD_1919	1533	66,08	279	2,40	314	2,32	0,97
era	SMD_3125	897	65,89	338	2,41	299	2,32	0,97
-	SMD_0248	1137	65,44	15	2,83	12	2,73	0,97
-	SMD_1842	1179	66,75	161	2,63	166	2,53	0,97
-	SMD_3897	645	71,16	128	3,32	131	3,20	0,96
gmk	SMD_3445	666	68,47	132	3,37	116	3,25	0,96
-	SMD_3611	1059	68,18	25	2,67	23	2,58	0,96

pdxK	SMD_1798	909	68,87	92	2,95	95	2,84	0,96
hrcA	SMD_1792	1065	69,39	38	3,29	64	3,18	0,96
purB	SMD_2770	1368	67,18	182	3,24	165	3,13	0,96
-	SMD_0968	528	68,18	115	3,50	123	3,37	0,96
pitA	SMD_1645	1122	66,04	288	2,53	281	2,44	0,96
-	SMD_3516	975	65,54	22	2,62	26	2,52	0,96
-	SMD_3567	339	68,14	61	2,90	71	2,79	0,96
trpB	SMD_2992	1218	67,65	46	3,53	45	3,39	0,96
-	SMD_4035	1179	66,67	341	2,89	353	2,78	0,96
rpsH	SMD_0799	399	60,40	3918	2,58	3165	2,49	0,96
ileS	SMD_1197	2832	67,73	215	2,39	224	2,29	0,96
potF	SMD_1411	1110	63,87	171	2,31	226	2,22	0,96
-	SMD_2600	216	63,43	121	3,07	144	2,95	0,96
cbsB	SMD_0527	1371	64,92	397	2,67	414	2,57	0,96
gltA	SMD_3438	1278	63,85	1431	2,18	1395	2,10	0,96
nrdR	SMD_0610	522	63,60	168	3,73	145	3,58	0,96
-	SMD_1966	1314	66,44	131	2,01	128	1,93	0,96
exbD3	SMD_0011	381	61,42	1923	2,10	1734	2,02	0,96
acpP	SMD_0980	240	57,08	4836	2,16	4916	2,08	0,96
-	SMD_4114	2358	67,60	119	2,91	86	2,80	0,96
fimV	SMD_3002	2013	72,18	121	2,70	131	2,59	0,96
-	SMD_3264	864	69,56	43	4,46	28	4,29	0,96
tuf	SMD_0770	1191	64,32	4421	2,56	4765	2,46	0,96
-	SMD_0634	789	70,72	21	6,31	25	6,06	0,96
pyrB	SMD_1023	951	70,35	151	3,07	152	2,95	0,96
-	SMD_1052	708	70,62	92	2,43	117	2,33	0,96
-	SMD_2772	1413	68,65	115	2,59	121	2,49	0,96
-	SMD_3249	2100	69,14	51	3,34	51	3,21	0,96
-	SMD_4050	2226	67,83	111	2,92	129	2,80	0,96
-	SMD_2840	807	66,67	145	2,78	162	2,66	0,96
-	SMD_3374	1899	58,93	71	3,49	64	3,35	0,96
atpE	SMD_3703	306	66,67	2546	2,63	3222	2,52	0,96
dacC	SMD_3638	1221	67,32	313	2,39	341	2,30	0,96
tpiA	SMD_2983	756	68,25	222	3,14	217	3,01	0,96
-	SMD_4019	477	70,44	116	3,87	111	3,72	0,96
-	SMD_4102	1686	72,54	92	2,94	72	2,82	0,96
-	SMD_3130	1533	69,41	77	2,68	219	2,57	0,96
-	SMD_2714	1632	68,26	25	2,76	27	2,65	0,96
ptsI	SMD_1027	1770	69,04	65	2,81	81	2,69	0,96
alf1	SMD_3400	1005	64,78	898	2,55	783	2,44	0,96
-	SMD_2375	2352	66,16	65	2,93	79	2,81	0,96
-	SMD_2293	348	64,94	21	4,35	16	4,17	0,96
-	SMD_0167	3600	69,03	14	3,54	15	3,40	0,96
-	SMD_0335	1473	67,55	116	3,69	216	3,53	0,96
-	SMD_4159	2148	68,48	194	2,56	197	2,45	0,96
flgF	SMD_2084	750	66,67	122	2,06	72	1,97	0,96
rpsD	SMD_0808	630	62,54	5842	2,15	4918	2,06	0,96
odhL	SMD_2776	1437	64,79	872	1,99	876	1,90	0,96
-	SMD_2626	855	69,82	24	2,58	34	2,47	0,96
zwf	SMD_1729	1437	68,06	74	3,30	111	3,15	0,96
-	SMD_0571	1092	67,86	115	3,30	121	3,15	0,96
-	SMD_0480	375	72,53	33	4,30	31	4,11	0,96
nadE2	SMD_2145	1668	66,73	64	2,20	83	2,10	0,96
-	SMD_4018	963	73,42	119	4,06	111	3,88	0,96
dat	SMD_1253	1401	64,88	771	2,14	519	2,05	0,96
fusA	SMD_0782	2142	62,75	2751	2,20	2491	2,11	0,96
-	SMD_2144	441	63,27	112	1,91	147	1,83	0,96
-	SMD_3778	1131	67,90	88	5,58	55	5,33	0,95
ampR	SMD_3328	867	70,93	21	2,50	23	2,39	0,95
cyoB	SMD_1214	1941	61,77	1214	2,19	1193	2,09	0,95
nuoF	SMD_2975	1341	65,03	1123	2,01	1127	1,91	0,95
phnA	SMD_3089	336	61,90	376	2,64	471	2,51	0,95
icd	SMD_3868	2223	66,31	311	2,89	314	2,75	0,95
-	SMD_3670	2097	66,29	159	2,51	151	2,39	0,95
atpD	SMD_3698	1407	64,04	1636	2,09	1778	1,99	0,95

yhbH	SMD_1033	318	61,64	412	2,73	579	2,59	0,95
cusA2	SMD_2139	3126	63,28	138	2,03	177	1,93	0,95
tolQ	SMD_3313	780	66,79	185	2,91	221	2,77	0,95
pilE	SMD_1452	408	63,73	63	2,36	51	2,24	0,95
minE	SMD_1166	261	60,54	559	2,28	724	2,16	0,95
-	SMD_4064	948	65,82	132	3,64	126	3,45	0,95
bioA	SMD_3231	1392	70,11	75	2,88	73	2,74	0,95
-	SMD_2841	1155	66,67	134	2,65	137	2,51	0,95
gltB	SMD_0064	4389	68,33	27	3,18	41	3,01	0,95
gcvH	SMD_3224	396	63,64	789	2,44	796	2,32	0,95
rnc	SMD_3126	681	66,81	179	2,24	146	2,13	0,95
-	SMD_2800	1518	66,01	283	2,57	257	2,44	0,95
wbpL	SMD_1843	990	64,95	113	2,88	117	2,73	0,95
-	SMD_3678	795	70,31	53	2,71	43	2,57	0,95
sspA	SMD_1433	618	63,92	154	2,57	214	2,43	0,95
-	SMD_1710	516	67,64	422	2,51	494	2,38	0,95
atpA	SMD_3700	1548	64,66	1772	2,29	1762	2,17	0,95
-	SMD_4071	375	69,07	111	3,35	111	3,17	0,95
-	SMD_0309	573	61,26	7685	2,84	6844	2,69	0,95
-	SMD_0153	576	62,85	661	5,58	833	5,27	0,95
-	SMD_1524	234	64,53	1	1,64	2	1,55	0,95
-	SMD_2146	1272	66,35	38	2,32	65	2,19	0,94
-	SMD_3781	765	69,02	148	4,28	151	4,04	0,94
ftsA	SMD_0647	1236	68,37	221	1,93	251	1,82	0,94
-	SMD_2628	537	65,18	73	3,35	71	3,17	0,94
-	SMD_4028	690	71,01	63	3,38	56	3,19	0,94
-	SMD_3326	1215	68,31	36	3,79	42	3,58	0,94
-	SMD_3479	945	65,08	66	3,27	76	3,08	0,94
-	SMD_1835	1038	67,73	93	2,90	69	2,74	0,94
glyA	SMD_0607	1254	66,27	395	3,32	381	3,13	0,94
-	SMD_0122	468	69,44	163	5,11	162	4,81	0,94
-	SMD_0082	984	69,72	38	2,54	35	2,39	0,94
glmS	SMD_3685	1839	66,83	179	2,74	177	2,58	0,94
btuE	SMD_4218	546	67,03	217	2,32	215	2,18	0,94
-	SMD_3830	828	70,53	21	4,11	21	3,87	0,94
metH2	SMD_2753	1092	68,22	48	3,25	45	3,05	0,94
-	SMD_3376	1854	67,53	176	2,56	185	2,41	0,94
-	SMD_1173	372	68,55	54	2,38	45	2,24	0,94
-	SMD_3030	2106	65,24	198	2,18	159	2,05	0,94
tatB	SMD_4172	453	69,32	161	3,95	185	3,71	0,94
mmsB	SMD_0233	891	68,13	92	2,94	111	2,77	0,94
-	SMD_0139	864	65,74	21	3,15	19	2,96	0,94
tdh	SMD_0835	1038	64,16	211	2,57	219	2,41	0,94
-	SMD_4150	252	61,11	181	3,29	173	3,09	0,94
arcA	SMD_0276	921	67,32	138	2,59	85	2,43	0,94
-	SMD_2920	1791	68,45	63	2,68	76	2,52	0,94
-	SMD_0475	276	71,74	171	2,72	172	2,55	0,94
-	SMD_2110	1125	66,93	49	3,55	51	3,33	0,94
DLAT	SMD_3709	1329	66,59	516	2,81	514	2,64	0,94
-	SMD_1748	492	63,62	775	2,26	735	2,12	0,94
-	SMD_0533	921	60,59	519	1,96	475	1,84	0,94
truC	SMD_0188	744	70,30	111	3,99	85	3,75	0,94
-	SMD_1064	519	68,59	99	2,78	113	2,61	0,94
-	SMD_1300	1071	65,08	47	3,86	45	3,62	0,94
speD	SMD_3899	795	56,35	1165	2,79	1114	2,61	0,94
-	SMD_4145	759	64,30	126	2,67	111	2,50	0,94
rpmA	SMD_1192	264	65,53	2971	2,46	2579	2,31	0,94
metC	SMD_0528	1173	66,50	212	2,64	248	2,47	0,94
rpfG	SMD_2014	1062	65,54	121	2,61	126	2,45	0,94
speE	SMD_3952	855	63,98	253	2,67	227	2,50	0,94
sahH	SMD_0664	1446	63,62	1189	2,37	916	2,22	0,94
-	SMD_0206	1611	67,97	215	2,19	196	2,05	0,94
-	SMD_4052	591	62,94	198	2,88	223	2,69	0,94
panC	SMD_1717	840	68,10	169	2,78	153	2,60	0,94
-	SMD_3693	471	67,09	71	2,99	87	2,80	0,94

tuf2	SMD_0783	1191	64,15	3943	2,18	4639	2,04	0,93
-	SMD_3954	792	64,77	1	3,14	1	2,94	0,93
motB2	SMD_2041	1017	69,12	55	2,57	26	2,40	0,93
-	SMD_0914	1257	64,28	25	2,89	36	2,70	0,93
rpsF	SMD_2728	438	62,10	3321	2,46	2871	2,30	0,93
atpF	SMD_3702	522	61,69	1461	2,62	1517	2,45	0,93
rpsK	SMD_0807	390	63,08	4682	2,16	3883	2,02	0,93
-	SMD_1305	1539	66,34	43	3,57	32	3,33	0,93
-	SMD_0660	486	66,67	33	3,42	45	3,20	0,93
dapD	SMD_1347	1080	67,04	231	2,12	232	1,98	0,93
-	SMD_3463	549	69,03	175	2,57	182	2,39	0,93
-	SMD_1374	4614	70,63	24	3,10	21	2,89	0,93
bfr2	SMD_3890	471	58,60	219	3,36	211	3,13	0,93
pepA	SMD_0569	1479	70,72	269	2,92	274	2,72	0,93
hmsS	SMD_2864	471	68,37	17	2,24	19	2,08	0,93
ampDII	SMD_0107	822	66,79	59	3,61	54	3,36	0,93
map	SMD_1345	843	64,53	488	2,41	414	2,24	0,93
-	SMD_1056	1494	67,07	248	2,51	267	2,34	0,93
cyoC	SMD_1215	639	63,54	1134	2,14	918	1,99	0,93
sufD	SMD_1076	1242	70,61	93	2,56	76	2,38	0,93
nuoE	SMD_2976	528	64,02	1132	1,97	886	1,83	0,93
panB	SMD_1716	816	68,26	194	2,68	212	2,49	0,93
plsC	SMD_0096	750	67,87	166	4,11	164	3,81	0,93
-	SMD_0173	2850	68,00	64	2,95	75	2,74	0,93
-	SMD_3442	387	68,22	548	2,90	428	2,69	0,93
-	SMD_3540	735	68,03	191	3,02	191	2,80	0,93
-	SMD_1037	576	67,01	81	2,41	92	2,24	0,93
rpsU	SMD_0374	216	63,89	3177	3,49	3651	3,23	0,93
-	SMD_3352	1203	69,99	15	3,23	16	3,00	0,93
ppa	SMD_3505	537	64,99	852	2,49	842	2,30	0,93
flgJ	SMD_2078	1203	65,50	71	2,04	51	1,89	0,93
pepQ	SMD_3461	1332	67,49	99	2,96	112	2,74	0,93
-	SMD_3826	927	68,39	18	6,44	23	5,96	0,93
-	SMD_1968	1227	65,44	178	2,01	157	1,86	0,93
cysS	SMD_2877	1377	68,85	166	3,21	168	2,97	0,92
-	SMD_0977	783	66,92	189	3,48	133	3,22	0,92
fimA	SMD_0595	540	65,56	3141	2,45	2193	2,27	0,92
-	SMD_4200	702	68,80	126	2,65	119	2,45	0,92
-	SMD_3852	843	66,31	158	2,71	215	2,50	0,92
-	SMD_0662	1344	66,22	27	3,30	36	3,05	0,92
bglX	SMD_0379	2175	68,69	59	2,56	61	2,36	0,92
-	SMD_0252	3489	65,86	16	3,40	8	3,14	0,92
-	SMD_0491	684	70,47	169	2,38	168	2,20	0,92
-	SMD_1789	1308	71,87	9	3,32	6	3,06	0,92
-	SMD_3871	819	68,38	125	3,71	99	3,42	0,92
sugE2	SMD_3898	321	64,17	179	3,24	134	2,99	0,92
-	SMD_2624	654	61,47	83	2,89	98	2,66	0,92
tgt	SMD_1809	1131	68,79	98	2,83	86	2,61	0,92
ribH	SMD_0617	468	69,02	249	3,30	229	3,04	0,92
-	SMD_2633	267	55,43	546	2,12	717	1,96	0,92
ubiE	SMD_3651	762	65,49	413	3,67	391	3,38	0,92
-	SMD_3387	987	68,49	21	2,76	25	2,54	0,92
flhF	SMD_2048	1617	71,68	48	2,29	31	2,11	0,92
birA	SMD_0265	969	70,59	119	4,21	115	3,87	0,92
-	SMD_1741	270	63,33	66	2,86	62	2,63	0,92
-	SMD_2100	615	69,43	96	2,33	113	2,14	0,92
kdsB	SMD_1468	774	70,03	64	2,28	82	2,10	0,92
-	SMD_0970	924	69,59	21	3,12	32	2,87	0,92
smeE	SMD_3657	3123	65,19	321	2,74	447	2,51	0,92
-	SMD_1188	726	64,88	147	2,52	142	2,31	0,92
rplJ	SMD_0776	597	63,48	7422	3,25	5882	2,98	0,92
sucB	SMD_2775	1203	66,83	912	2,21	925	2,02	0,92
smeG	SMD_2748	1269	69,98	183	2,39	263	2,19	0,92
rpmH	SMD_4239	141	65,96	2298	3,84	2166	3,52	0,92
mtaP	SMD_3117	747	67,74	121	2,88	129	2,63	0,92

rarD	SMD_0287	897	65,55	58	3,41	43	3,13	0,92
-	SMD_0759	3309	68,96	7	3,86	3	3,53	0,92
-	SMD_0348	1710	66,43	131	2,22	123	2,03	0,92
-	SMD_2164	468	65,17	48	1,49	38	1,36	0,91
-	SMD_0245	1077	64,16	14	3,45	11	3,16	0,91
fliS	SMD_2073	417	68,59	178	2,21	117	2,02	0,91
hrpB	SMD_4070	2505	72,14	33	3,65	31	3,34	0,91
-	SMD_1251	885	67,46	176	2,32	121	2,12	0,91
-	SMD_4217	1011	68,55	172	2,68	177	2,45	0,91
-	SMD_0408	402	70,90	41	3,44	29	3,14	0,91
rnfB	SMD_3082	414	69,32	77	2,72	112	2,48	0,91
-	SMD_2887	1344	67,63	182	2,46	164	2,24	0,91
-	SMD_0033	2271	66,18	129	3,07	117	2,79	0,91
-	SMD_0752	660	70,76	123	2,67	153	2,43	0,91
ksgA	SMD_0699	804	67,29	124	2,65	135	2,41	0,91
nth	SMD_1387	693	67,53	41	1,52	59	1,39	0,91
bmnA	SMD_1969	2619	67,12	29	2,28	27	2,06	0,91
-	SMD_3015	8289	66,64	11	3,21	5	2,91	0,91
cyoA	SMD_1213	1041	61,29	1146	2,45	1131	2,22	0,91
hupB	SMD_0873	273	60,44	3517	2,34	3612	2,12	0,91
-	SMD_2039	1266	71,80	31	2,51	21	2,27	0,90
secB	SMD_0125	519	65,90	848	2,46	953	2,22	0,90
trpD	SMD_3904	1032	67,83	51	2,95	53	2,66	0,90
gcvP	SMD_3153	2868	67,43	185	2,73	184	2,47	0,90
-	SMD_3933	1389	71,06	258	2,14	257	1,93	0,90
-	SMD_0570	1089	68,87	84	4,10	96	3,70	0,90
rnhB	SMD_1324	660	70,76	94	2,43	71	2,19	0,90
-	SMD_1865	1749	55,40	111	2,25	99	2,03	0,90
-	SMD_0990	381	60,89	21	2,61	32	2,35	0,90
-	SMD_3575	831	67,51	43	2,94	71	2,65	0,90
-	SMD_2070	639	68,23	124	3,37	96	3,03	0,90
-	SMD_1234	240	67,50	61	5,01	67	4,51	0,90
-	SMD_1070	2295	65,71	326	2,38	277	2,13	0,90
-	SMD_2253	903	65,89	54	3,20	41	2,86	0,90
yqgE	SMD_1021	567	67,90	238	3,96	245	3,54	0,90
rplI	SMD_2726	450	66,22	2324	2,96	2159	2,65	0,90
mnmA	SMD_2101	1128	68,44	135	2,49	137	2,23	0,89
rplU	SMD_1191	309	59,22	4163	2,45	3571	2,19	0,89
-	SMD_3027	2790	63,76	1131	3,06	854	2,73	0,89
-	SMD_3676	483	62,53	289	2,55	275	2,28	0,89
-	SMD_2796	1155	66,58	72	2,96	79	2,64	0,89
-	SMD_2072	294	68,37	115	2,49	65	2,22	0,89
ftsZ	SMD_0648	1236	67,23	384	3,28	444	2,92	0,89
ubiH	SMD_0706	1209	71,79	113	3,17	118	2,82	0,89
cmk	SMD_1838	678	66,08	751	2,62	744	2,33	0,89
-	SMD_0269	864	65,74	18	3,61	16	3,22	0,89
ruvC	SMD_3318	522	67,62	173	2,68	168	2,39	0,89
-	SMD_1592	267	67,42	12	3,54	2	3,16	0,89
apaH	SMD_0697	1017	70,11	181	2,74	156	2,44	0,89
crt2	SMD_1389	783	70,11	69	3,48	69	3,09	0,89
-	SMD_0828	1020	68,24	31	4,65	47	4,13	0,89
codA	SMD_3652	453	67,55	89	3,19	79	2,83	0,89
-	SMD_0381	1167	67,87	47	3,40	42	3,03	0,89
fimU	SMD_1447	474	65,19	113	3,62	72	3,22	0,89
-	SMD_2576	903	66,78	59	2,64	45	2,34	0,89
-	SMD_4006	1779	68,58	19	3,96	15	3,50	0,89
-	SMD_3880	279	65,95	794	2,74	891	2,42	0,89
-	SMD_0090	1485	69,70	41	3,75	41	3,32	0,89
-	SMD_1846	1209	68,40	112	2,27	119	2,00	0,88
-	SMD_0711	723	68,05	34	2,83	23	2,50	0,88
-	SMD_0297	1368	70,54	222	3,26	231	2,88	0,88
msrB	SMD_0479	468	69,23	16	3,43	47	3,03	0,88
-	SMD_2540	660	70,15	51	3,15	62	2,78	0,88
etfB	SMD_0543	747	66,53	321	2,59	413	2,29	0,88
-	SMD_0608	420	69,52	114	2,77	86	2,45	0,88

rhlB	SMD_3855	1782	68,74	269	3,22	257	2,84	0,88
-	SMD_1446	1395	67,46	169	2,63	159	2,31	0,88
-	SMD_1057	297	65,99	4251	9,92	2712	8,73	0,88
-	SMD_3417	1320	65,23	117	2,24	119	1,97	0,88
smmQ	SMD_4140	3210	67,26	41	2,86	63	2,52	0,88
-	SMD_3081	606	64,69	159	3,02	157	2,66	0,88
-	SMD_0883	981	58,21	113	3,93	123	3,45	0,88
trxB	SMD_2112	972	68,11	195	2,85	231	2,50	0,88
-	SMD_2806	2169	64,50	395	2,84	419	2,50	0,88
asd	SMD_3003	1029	65,79	223	2,80	239	2,46	0,88
fpr	SMD_2804	780	64,62	291	3,01	288	2,63	0,88
-	SMD_1918	729	69,82	78	2,48	85	2,17	0,88
fliH	SMD_2062	642	71,03	62	2,36	43	2,06	0,87
xerC	SMD_3663	897	69,90	88	2,72	89	2,38	0,87
-	SMD_1593	651	66,05	4	4,07	5	3,56	0,87
-	SMD_3393	2064	68,17	37	3,46	35	3,02	0,87
pyrG	SMD_1651	1665	65,95	253	2,75	237	2,40	0,87
efp	SMD_2888	567	62,26	923	2,61	836	2,28	0,87
-	SMD_1336	1539	70,57	146	3,63	164	3,17	0,87
-	SMD_2316	1080	67,31	48	2,96	27	2,58	0,87
-	SMD_1585	915	68,96	113	2,38	98	2,07	0,87
-	SMD_2338	1197	67,08	71	2,62	45	2,28	0,87
-	SMD_0305	816	63,48	796	2,10	1176	1,82	0,87
-	SMD_1731	876	70,09	75	3,76	83	3,26	0,87
-	SMD_3839	768	72,79	63	3,96	55	3,43	0,87
-	SMD_4116	561	70,23	26	3,48	19	3,02	0,87
-	SMD_0476	489	64,21	584	2,46	521	2,13	0,87
murD	SMD_1091	1410	69,50	118	2,19	113	1,89	0,87
rplD	SMD_0786	606	63,04	6444	3,34	5939	2,88	0,86
selA	SMD_3450	1425	69,82	21	4,09	28	3,53	0,86
-	SMD_2740	687	67,39	62	2,45	61	2,11	0,86
pyrF	SMD_0054	726	69,42	213	3,32	168	2,86	0,86
truD	SMD_1659	1032	68,41	81	2,30	77	1,98	0,86
-	SMD_2120	1269	67,22	16	2,92	9	2,52	0,86
emrA	SMD_1360	1182	68,02	54	2,72	168	2,34	0,86
-	SMD_0191	603	66,17	119	3,80	94	3,27	0,86
-	SMD_1080	468	62,82	152	4,01	175	3,45	0,86
proA	SMD_2865	1272	68,71	41	2,72	35	2,34	0,86
rplN	SMD_0795	369	59,89	7188	3,34	6277	2,87	0,86
rpmE	SMD_3439	243	56,79	2219	2,68	1911	2,30	0,86
-	SMD_4045	918	68,63	117	3,75	95	3,21	0,86
flgC	SMD_2087	408	64,46	87	2,26	31	1,93	0,85
acrD	SMD_2749	3174	64,71	281	2,19	385	1,87	0,85
-	SMD_1790	918	68,63	31	3,21	27	2,73	0,85
-	SMD_0965	321	62,93	318	2,65	481	2,26	0,85
-	SMD_4118	1614	70,94	33	3,86	21	3,29	0,85
holB	SMD_0985	957	68,76	112	2,29	117	1,95	0,85
hutH	SMD_2691	1542	69,07	18	3,58	26	3,05	0,85
tag	SMD_1019	546	66,67	56	3,75	43	3,19	0,85
-	SMD_3106	1176	68,03	146	2,94	152	2,51	0,85
metK	SMD_0668	1212	65,35	323	2,61	341	2,22	0,85
ispB	SMD_1096	999	69,57	241	3,51	226	2,98	0,85
vdh	SMD_3104	1098	65,76	115	3,24	84	2,75	0,85
-	SMD_4089	426	66,90	98	2,25	72	1,91	0,85
flgG	SMD_2083	786	63,61	112	2,17	75	1,84	0,85
-	SMD_3107	342	69,01	6122	4,25	5945	3,60	0,85
rplX	SMD_0796	318	62,89	7437	3,43	6528	2,90	0,85
-	SMD_2987	858	66,32	111	2,57	92	2,18	0,85
-	SMD_2390	927	69,15	16	3,74	15	3,16	0,85
truB	SMD_2960	909	70,74	93	2,38	74	2,01	0,84
rplC	SMD_0785	651	65,13	6711	3,53	5925	2,98	0,84
cspD	SMD_3116	207	62,80	1443	2,34	1519	1,97	0,84
pfk	SMD_3490	1257	69,29	46	3,77	65	3,17	0,84
cydA	SMD_2855	1590	63,65	1861	2,61	1688	2,20	0,84
-	SMD_1886	945	67,83	11	3,71	14	3,12	0,84

smeM	SMD_3392	1107	68,56	31	3,16	29	2,66	0,84
fleN	SMD_2047	888	69,03	96	2,45	72	2,06	0,84
-	SMD_3586	1059	66,19	51	2,45	81	2,06	0,84
flgL	SMD_2079	1881	67,41	86	2,23	49	1,88	0,84
-	SMD_1359	1494	71,15	34	3,30	94	2,77	0,84
-	SMD_0118	1281	67,68	133	4,30	166	3,61	0,84
-	SMD_2299	1302	64,13	84	2,73	88	2,29	0,84
-	SMD_1464	663	69,38	161	3,54	168	2,97	0,84
-	SMD_3613	1029	69,48	46	2,62	51	2,20	0,84
-	SMD_4095	1251	66,59	65	2,52	57	2,11	0,84
adhC	SMD_3577	1110	65,50	121	3,10	161	2,60	0,84
-	SMD_3595	738	69,11	139	2,80	154	2,34	0,84
-	SMD_0288	2826	65,85	681	3,15	413	2,64	0,84
-	SMD_0349	663	66,37	111	2,38	121	1,99	0,84
-	SMD_1354	2307	70,48	21	3,63	21	3,03	0,84
-	SMD_0101	828	67,51	273	2,84	285	2,37	0,83
rpsC	SMD_0791	735	62,59	6582	3,55	5759	2,95	0,83
nuoI	SMD_2972	489	61,15	738	1,99	615	1,66	0,83
-	SMD_1644	627	64,11	296	2,64	299	2,20	0,83
-	SMD_0099	612	68,30	179	4,11	152	3,41	0,83
-	SMD_1981	717	69,04	46	3,01	56	2,50	0,83
-	SMD_3876	2178	68,50	25	3,55	25	2,95	0,83
smmD2	SMD_1476	1074	65,74	14	3,39	9	2,81	0,83
rpsQ	SMD_0794	270	57,78	8574	3,43	7394	2,84	0,83
-	SMD_0232	1176	66,92	133	2,56	171	2,11	0,83
-	SMD_1866	1077	50,60	87	2,27	75	1,87	0,83
rpmC	SMD_0793	186	57,53	8653	3,41	7849	2,81	0,82
-	SMD_3073	1317	70,39	18	3,41	24	2,81	0,82
-	SMD_1368	483	68,94	48	2,07	55	1,70	0,82
-	SMD_0650	957	66,67	125	3,42	215	2,81	0,82
rplE	SMD_0797	543	58,01	9681	3,48	8561	2,86	0,82
rplV	SMD_0790	336	61,90	6466	3,58	5896	2,94	0,82
-	SMD_0455	1101	66,94	271	2,81	129	2,30	0,82
phaD	SMD_3916	1536	68,95	45	3,08	32	2,51	0,82
-	SMD_3320	618	72,17	83	3,15	76	2,58	0,82
recX	SMD_1679	492	69,11	153	2,97	174	2,42	0,82
metE	SMD_2261	1035	64,15	11	4,69	12	3,83	0,82
flgA	SMD_2090	657	68,65	35	1,86	18	1,51	0,81
-	SMD_4225	438	68,95	51	4,20	45	3,42	0,81
-	SMD_0513	348	68,10	31	3,43	36	2,79	0,81
-	SMD_0969	1950	70,56	11	3,25	21	2,65	0,81
rplP	SMD_0792	414	61,84	6713	3,51	5934	2,86	0,81
-	SMD_2984	936	65,17	324	2,92	314	2,37	0,81
rlmH	SMD_3061	471	67,30	82	3,47	57	2,82	0,81
leuD	SMD_3523	579	66,49	12	1,77	22	1,43	0,81
rpsJ	SMD_0784	312	61,22	8248	3,56	7251	2,88	0,81
rplW	SMD_0787	300	59,00	6842	3,57	6144	2,89	0,81
-	SMD_0178	1722	70,62	77	3,68	62	2,98	0,81
ohr	SMD_0234	426	67,14	62	3,88	47	3,14	0,81
fpg	SMD_0041	813	69,86	45	3,85	36	3,11	0,81
-	SMD_1232	408	65,69	18	4,29	21	3,47	0,81
lepB2	SMD_0195	627	69,22	18	4,59	31	3,71	0,81
hemB	SMD_4044	990	67,07	246	2,72	237	2,20	0,81
apbE	SMD_1062	1023	69,89	98	2,59	112	2,09	0,81
-	SMD_4174	894	68,79	161	5,37	221	4,33	0,81
rplR	SMD_0801	354	67,51	2959	2,77	2264	2,23	0,80
-	SMD_2741	981	69,62	39	3,09	47	2,48	0,80
-	SMD_1403	1464	68,24	125	3,03	79	2,44	0,80
rplB	SMD_0788	828	66,18	5814	3,55	5231	2,85	0,80
-	SMD_3339	1611	68,09	26	3,99	25	3,19	0,80
-	SMD_0830	507	68,05	213	5,37	229	4,29	0,80
mtnD	SMD_1971	558	65,77	157	3,29	151	2,62	0,80
-	SMD_1954	1590	67,11	71	3,16	66	2,51	0,80
-	SMD_1286	180	67,78	811	2,15	961	1,71	0,80
-	SMD_1032	426	71,83	74	3,34	129	2,65	0,79

-	SMD_3888	1788	69,91	81	3,92	113	3,11	0,79
aroE	SMD_4041	846	70,80	129	3,72	115	2,95	0,79
-	SMD_3018	354	63,56	15	3,83	6	3,03	0,79
masA	SMD_1970	696	66,24	158	2,92	188	2,31	0,79
-	SMD_3927	606	69,80	163	2,49	156	1,97	0,79
smeD	SMD_3658	1185	69,70	188	3,26	328	2,57	0,79
-	SMD_0552	588	69,05	75	2,60	81	2,05	0,79
-	SMD_0525	885	71,86	181	3,47	183	2,73	0,79
flhA	SMD_2049	2109	68,14	21	2,30	11	1,81	0,79
acpP2	SMD_4104	258	62,02	263	2,56	229	2,01	0,78
-	SMD_0306	687	70,89	32	2,67	21	2,09	0,78
-	SMD_3677	567	64,73	121	2,58	96	2,03	0,78
ilvD	SMD_4038	1839	68,95	31	3,36	36	2,63	0,78
-	SMD_0396	489	65,64	147	4,13	131	3,24	0,78
-	SMD_0962	1608	66,73	21	3,41	23	2,67	0,78
-	SMD_0446	1521	67,98	45	5,94	42	4,65	0,78
-	SMD_2479	657	68,95	86	2,57	74	2,01	0,78
-	SMD_3414	1953	68,10	42	6,36	45	4,97	0,78
rpsS	SMD_0789	270	57,78	8619	3,70	7692	2,89	0,78
-	SMD_2220	570	65,79	9	2,18	8	1,70	0,78
rpmJ2	SMD_1837	126	58,73	2152	2,68	2491	2,09	0,78
ubiG	SMD_2886	717	69,46	116	2,43	97	1,89	0,78
-	SMD_1420	576	69,10	46	3,04	43	2,37	0,78
rpsN	SMD_0798	306	63,40	6184	3,85	5638	2,99	0,78
rpe	SMD_3910	678	66,81	124	2,46	152	1,91	0,78
-	SMD_4032	987	68,79	58	2,44	52	1,89	0,78
glnB3	SMD_3957	339	62,24	139	3,42	188	2,65	0,78
-	SMD_0861	759	63,90	249	3,41	284	2,65	0,78
-	SMD_3717	777	65,51	258	3,48	253	2,69	0,77
dsbB	SMD_0811	507	68,05	121	3,45	91	2,65	0,77
-	SMD_0025	1029	67,54	3416	3,27	3433	2,51	0,77
-	SMD_2616	873	68,50	43	4,22	47	3,24	0,77
hisA	SMD_1951	735	70,61	15	2,35	17	1,80	0,77
-	SMD_3021	459	67,97	37	6,54	22	4,99	0,76
prpB	SMD_3182	939	65,81	121	2,47	86	1,88	0,76
-	SMD_0940	405	62,22	282	2,94	268	2,24	0,76
-	SMD_2035	2250	67,78	22	3,52	13	2,65	0,75
def	SMD_3777	513	62,77	248	3,56	284	2,68	0,75
-	SMD_3342	483	67,49	27	2,86	33	2,15	0,75
-	SMD_0281	297	69,70	83	3,88	95	2,91	0,75
-	SMD_0137	273	63,74	543	11,29	1129	8,40	0,74
phzF	SMD_1087	879	71,44	64	3,41	64	2,53	0,74
sspB	SMD_1434	453	67,77	237	3,19	212	2,34	0,73
dsbA	SMD_3593	657	69,71	81	3,50	98	2,53	0,72
-	SMD_3576	357	70,59	85	3,41	77	2,46	0,72
-	SMD_4208	399	64,41	61	3,47	38	2,50	0,72
gst4	SMD_2798	636	67,14	26	4,24	43	3,06	0,72
-	SMD_0406	591	67,34	81	4,01	68	2,88	0,72
-	SMD_2134	312	65,06	23	2,77	29	1,98	0,71
-	SMD_0428	681	69,16	25	3,85	23	2,74	0,71
-	SMD_1410	1398	67,53	34	2,48	39	1,75	0,71
sodA2	SMD_2474	630	66,51	18	3,88	21	2,74	0,70
leuA	SMD_3520	1587	67,55	11	3,50	11	2,46	0,70
-	SMD_3733	342	68,13	28	3,95	22	2,77	0,70
-	SMD_0464	588	69,56	17	3,00	18	2,10	0,70
pcm	SMD_1662	678	68,73	158	3,10	162	2,17	0,70
-	SMD_1836	330	67,88	93	3,07	63	2,14	0,70
-	SMD_1225	813	68,88	16	3,29	28	2,29	0,70
-	SMD_1043	684	64,91	96	2,89	94	2,01	0,70
pssA2	SMD_2607	615	65,85	67	3,31	69	2,30	0,70
-	SMD_3266	537	68,16	37	7,15	45	4,97	0,70
-	SMD_1249	213	68,08	86	5,52	74	3,83	0,69
-	SMD_3050	1368	70,03	33	2,30	31	1,57	0,68
-	SMD_2760	861	66,55	656	2,92	634	1,99	0,68
-	SMD_3827	561	68,81	85	5,12	69	3,49	0,68

-	SMD_2488	978	68,20	15	3,33	36	2,26	0,68
-	SMD_2454	675	66,81	28	3,64	31	2,46	0,68
-	SMD_1351	885	67,68	19	2,35	19	1,57	0,67
-	SMD_2256	1152	65,89	8	2,94	11	1,96	0,67
-	SMD_1480	264	61,36	32	3,95	32	2,63	0,67
pssA	SMD_0562	777	68,34	231	4,56	227	3,03	0,67
-	SMD_3145	648	65,28	24	3,17	42	2,10	0,66
-	SMD_2684	276	67,03	76	3,38	96	2,23	0,66
-	SMD_4094	558	71,86	55	3,25	55	2,14	0,66
-	SMD_1506	633	64,93	45	4,01	61	2,63	0,66
smg	SMD_3780	474	63,50	297	4,84	333	3,16	0,65
-	SMD_2431	669	68,01	14	2,95	23	1,91	0,65
pai2	SMD_2734	639	69,17	74	3,37	87	2,18	0,65
trbP	SMD_2323	666	68,47	16	2,68	22	1,71	0,64
-	SMD_1379	354	68,64	31	9,52	29	6,07	0,64
yfiF	SMD_3388	834	73,62	62	2,91	63	1,84	0,63
-	SMD_0967	360	64,44	98	3,76	96	2,37	0,63
-	SMD_2575	435	67,59	22	3,84	16	2,41	0,63
-	SMD_3407	630	65,24	3751	3,75	4163	2,34	0,62
bfr	SMD_3173	576	63,89	42	2,97	49	1,84	0,62
-	SMD_2832	276	66,67	149	2,25	163	1,39	0,62
hisF	SMD_1952	777	66,80	22	2,50	21	1,52	0,61
thiL	SMD_0619	1026	69,49	88	3,48	73	2,10	0,60
feoA	SMD_1991	255	68,63	174	2,87	183	1,73	0,60
-	SMD_0254	228	54,82	26	3,93	5	2,36	0,60
-	SMD_2592	381	67,72	76	2,57	75	1,54	0,60
-	SMD_0161	408	67,65	115	8,12	89	4,80	0,59
-	SMD_0236	513	69,98	29	7,12	22	4,20	0,59
-	SMD_3032	495	61,82	24	2,83	12	1,62	0,57
soxR	SMD_1008	435	67,59	19	3,11	16	1,76	0,57
-	SMD_0692	495	68,28	17	3,28	21	1,84	0,56
-	SMD_3549	1176	68,96	215	3,24	274	1,81	0,56
-	SMD_1887	588	66,67	39	3,12	48	1,71	0,55
moaA	SMD_2441	981	69,62	32	3,08	36	1,67	0,54
moaBC	SMD_2440	987	69,91	21	2,80	23	1,50	0,54
cdh	SMD_2539	765	69,02	51	3,59	58	1,92	0,54
-	SMD_2107	381	66,93	14	3,28	12	1,69	0,52
-	SMD_3325	642	67,76	61	3,16	76	1,60	0,51
gcp	SMD_0375	984	72,66	112	5,31	115	2,66	0,50
agaS	SMD_4001	1155	70,04	11	3,55	21	1,76	0,49
-	SMD_2071	579	71,85	83	3,02	39	1,40	0,46

Table S2. Predicted operons and RNAs half-lives in *S. maltophilia* genome

		D457		ALB001	
Name	Synonym	Expression mid-exponential phase (RPKM) at t0 rifampicin addition	Half-life (min)	Expression mid-exponential phase (RPKM) at t0 rifampicin addition	Half-life (min)
dnaA	SMD_0001	157	3,13	116	3,60
dnaN	SMD_0002	312	2,40	585	2,42
recF	SMD_0003	36	2,89	30	4,65
gyrB	SMD_0004	186	2,60	237	2,66
-	SMD_0005	111	2,77	102	3,42
-	SMD_0006	161	3,00	112	4,34
-	SMD_0007	432	2,96	399	3,21
-	SMD_0008	1009	2,75	1261	2,73
exbB	SMD_0009	3475	2,10	6605	2,19
exbD	SMD_0010	2167	2,18	3857	2,20
exbD3	SMD_0011	4074	2,10	7317	2,02
-	SMD_0015	26	6,39	33	8,01
-	SMD_0016	281	3,05	272	3,28
aspC	SMD_0018	138	2,92	157	3,16
-	SMD_0025	262	3,27	547	2,51
-	SMD_0028	38	3,35	31	4,87
phhA	SMD_0029	119	3,32	103	4,69
-	SMD_0030	70	3,83	52	5,07
-	SMD_0031	41	5,03	43	5,29
-	SMD_0033	122	3,07	165	2,79
-	SMD_0034	127	2,72	303	2,66
fpg	SMD_0041	32	3,85	48	3,11
mdoD	SMD_0042	105	3,06	95	3,75
tdk	SMD_0043	47	3,93	43	4,60
-	SMD_0044	40	3,24	23	5,22
-	SMD_0045	24	4,40	18	5,47
rep	SMD_0046	72	2,62	62	3,30
-	SMD_0047	33	3,43	36	4,01
-	SMD_0050	36	3,92	23	5,62

-	SMD_0051	54	3,79	38	3,92
mviM	SMD_0052	52	3,83	53	4,57
hel	SMD_0053	315	2,64	417	2,90
pyrF	SMD_0054	99	3,32	136	2,86
plsB	SMD_0057	120	3,06	94	4,14
-	SMD_0058	72	2,53	29	3,79
rdgC	SMD_0059	142	2,77	194	3,10
-	SMD_0060	113	2,14	73	3,70
-	SMD_0061	46	3,38	43	4,01
gltB	SMD_0064	50	3,18	51	3,01
lhr	SMD_0075	22	5,55	21	7,40
-	SMD_0076	47	2,50	70	3,76
-	SMD_0077	104	2,72	119	3,10
-	SMD_0078	54	3,00	64	3,15
-	SMD_0079	29	2,80	31	3,34
-	SMD_0082	90	2,54	67	2,39
-	SMD_0083	74	2,38	93	2,45
-	SMD_0084	162	2,41	195	2,76
-	SMD_0085	216	2,38	204	2,59
-	SMD_0086	105	2,27	81	2,64
-	SMD_0088	124	2,82	143	2,89
-	SMD_0089	44	3,86	25	6,01
-	SMD_0090	21	3,75	24	3,32
-	SMD_0091	32	3,57	26	4,55
-	SMD_0093	23	3,94	29	4,23
-	SMD_0094	51	3,52	42	3,80
-	SMD_0095	266	1,90	273	2,42
plsC	SMD_0096	95	4,11	85	3,81
-	SMD_0097	48	3,53	45	3,78
-	SMD_0099	91	4,11	148	3,41
ssuE2	SMD_0100	230	2,47	71	3,52
-	SMD_0101	388	2,84	662	2,37
glnA	SMD_0103	48	3,71	64	3,97
ampDII	SMD_0107	57	3,61	49	3,36
mltA	SMD_0108	33	4,01	19	5,58

sodC	SMD_0114	98	2,79	134	2,82
sodC2	SMD_0115	232	2,64	279	2,63
-	SMD_0117	45	3,62	48	4,01
-	SMD_0118	27	4,30	27	3,61
-	SMD_0119	44	2,90	33	3,72
-	SMD_0120	40	3,11	34	4,70
-	SMD_0122	32	5,11	64	4,81
-	SMD_0123	171	3,29	128	4,10
-	SMD_0124	482	2,45	420	2,95
secB	SMD_0125	554	2,46	1042	2,22
gpsA	SMD_0126	394	2,41	540	2,40
-	SMD_0128	25	4,28	22	4,76
-	SMD_0129	107	2,53	56	4,77
arsR	SMD_0133	34	3,15	20	5,77
-	SMD_0136	135	2,77	109	3,98
-	SMD_0137	387	11,29	771	8,40
-	SMD_0139	91	3,15	73	2,96
nreB	SMD_0144	30	3,15	30	3,88
-	SMD_0152	74	2,77	55	3,55
-	SMD_0153	450	5,58	659	5,27
-	SMD_0156	76	3,09	48	4,13
-	SMD_0161	25	8,12	48	4,80
-	SMD_0163	29	3,21	24	6,25
-	SMD_0166	17	3,99	22	4,97
-	SMD_0167	29	3,54	31	3,40
-	SMD_0169	65	2,64	62	3,40
GCDH	SMD_0170	47	2,98	24	4,12
-	SMD_0173	74	2,95	92	2,74
-	SMD_0174	148	4,67	191	4,60
fabH	SMD_0175	93	3,31	124	3,64
dhaA	SMD_0176	124	3,06	121	3,57
-	SMD_0177	154	2,55	156	3,23
-	SMD_0178	69	3,68	89	2,98
-	SMD_0181	55	2,23	55	3,00
ubiB	SMD_0182	72	2,70	80	3,58

yaiO	SMD_0183	182	2,62	168	3,04
truC	SMD_0188	75	3,99	61	3,75
-	SMD_0189	115	3,22	45	4,04
-	SMD_0191	93	3,80	131	3,27
dcp	SMD_0192	165	3,06	324	3,12
lepB2	SMD_0195	20	4,59	21	3,71
-	SMD_0202	64	3,08	68	3,16
-	SMD_0204	339	2,03	217	2,51
-	SMD_0205	322	2,17	285	2,34
-	SMD_0206	281	2,19	248	2,05
ivd	SMD_0207	224	2,27	228	2,95
-	SMD_0208	25	2,41	32	2,59
-	SMD_0210	17	2,53	28	3,42
-	SMD_0211	33	2,82	32	3,51
-	SMD_0213	96	2,59	55	3,54
-	SMD_0214	102	2,67	76	2,68
RRM1	SMD_0215	186	2,87	235	3,09
RRM2	SMD_0216	475	2,17	643	2,50
-	SMD_0218	109	2,14	88	2,82
-	SMD_0220	87	3,50	98	3,59
-	SMD_0221	120	3,34	100	3,95
purU	SMD_0222	65	3,19	51	4,03
pncA	SMD_0223	125	3,01	100	3,58
-	SMD_0225	41	4,54	30	5,55
-	SMD_0226	96	4,68	63	5,89
-	SMD_0227	19	7,05	22	7,73
-	SMD_0230	56	2,58	87	2,56
-	SMD_0232	90	2,56	168	2,11
mmsB	SMD_0233	45	2,94	107	2,77
ohr	SMD_0234	43	3,88	135	3,14
-	SMD_0236	18	7,12	46	4,20
-	SMD_0238	21	1,65	43	3,37
-	SMD_0239	32	3,41	82	4,82
-	SMD_0242	35	3,31	69	3,72
-	SMD_0244	30	2,94	51	3,53

-	SMD_0245	30	3,45	52	3,16
-	SMD_0248	26	2,83	59	2,73
-	SMD_0251	20	2,61	65	2,77
-	SMD_0252	16	3,40	39	3,14
-	SMD_0254	16	3,93	52	2,36
-	SMD_0255	16	2,81	17	3,02
-	SMD_0256	36	3,73	51	3,87
-	SMD_0257	118	3,47	92	4,21
-	SMD_0258	91	2,82	97	3,32
dusA	SMD_0259	65	3,16	49	4,52
-	SMD_0260	243	2,90	176	4,44
-	SMD_0261	150	3,69	127	4,95
phoQ	SMD_0262	112	3,61	132	3,72
-	SMD_0263	83	4,02	61	5,56
-	SMD_0264	37	2,72	28	6,02
birA	SMD_0265	61	4,21	61	3,87
-	SMD_0267	17	3,49	17	4,73
-	SMD_0269	54	3,61	70	3,22
arcA	SMD_0276	114	2,59	103	2,43
ecnA	SMD_0277	389	2,75	800	2,73
trpS	SMD_0280	140	3,12	128	3,32
-	SMD_0281	62	3,88	77	2,91
-	SMD_0282	42	3,00	48	3,62
-	SMD_0283	96	3,06	59	2,99
-	SMD_0284	109	1,99	65	3,91
-	SMD_0285	45	2,94	46	3,35
-	SMD_0286	40	4,93	28	5,43
rarD	SMD_0287	99	3,41	79	3,13
-	SMD_0288	592	3,15	1629	2,64
-	SMD_0294	28	2,35	21	3,82
katA	SMD_0296	35	3,48	18	4,83
-	SMD_0297	172	3,26	170	2,88
-	SMD_0298	44	3,06	46	3,82
-	SMD_0299	65	3,69	48	4,28
-	SMD_0301	212	2,67	212	3,05

-	SMD_0302	161	2,57	199	3,08
-	SMD_0303	248	3,13	331	4,11
-	SMD_0304	80	3,69	121	3,68
-	SMD_0305	417	2,10	930	1,82
-	SMD_0306	47	2,67	44	2,09
-	SMD_0309	2879	2,84	5187	2,69
-	SMD_0310	194	3,53	153	4,63
-	SMD_0311	232	4,76	222	6,04
-	SMD_0312	41	5,16	50	5,34
-	SMD_0314	78	3,19	79	3,61
-	SMD_0315	93	3,69	71	5,67
-	SMD_0316	56	3,12	69	3,63
-	SMD_0317	199	2,82	221	2,86
argS	SMD_0318	162	3,27	227	3,23
dfp	SMD_0321	56	3,37	42	3,41
dut	SMD_0322	65	1,41	63	3,27
algC	SMD_0323	127	2,59	132	2,61
kdpE	SMD_0324	47	3,48	17	6,01
-	SMD_0329	330	2,13	329	2,78
pyrE	SMD_0330	268	2,35	378	2,49
ampN	SMD_0331	95	2,80	77	3,30
-	SMD_0333	148	3,44	192	3,68
-	SMD_0334	79	2,63	50	3,76
-	SMD_0335	42	3,69	44	3,53
tyrS	SMD_0336	143	2,70	182	3,10
-	SMD_0348	204	2,22	342	2,03
-	SMD_0349	149	2,38	197	1,99
-	SMD_0350	38	3,75	44	3,97
ctpA	SMD_0351	71	3,33	49	4,47
fadL	SMD_0353	207	2,37	411	2,30
nadR	SMD_0355	47	2,34	52	2,39
putA	SMD_0356	272	2,30	461	2,34
-	SMD_0366	47	3,29	46	4,89
cyoE	SMD_0367	123	2,78	115	3,07
-	SMD_0370	42	2,85	26	3,77

dnaG	SMD_0371	57	2,76	36	3,67
-	SMD_0372	36	3,65	22	4,61
-	SMD_0373	180	3,00	252	3,67
rpsU	SMD_0374	1000	3,49	1301	3,23
gcp	SMD_0375	26	5,31	27	2,66
-	SMD_0376	114	3,86	77	5,79
folB	SMD_0377	84	2,57	67	3,70
-	SMD_0378	82	2,91	110	3,36
bglX	SMD_0379	74	2,56	88	2,36
-	SMD_0381	74	3,40	90	3,03
-	SMD_0382	100	2,67	42	3,97
-	SMD_0384	26	2,99	22	3,39
-	SMD_0385	21	2,93	29	4,24
-	SMD_0388	25	2,59	22	4,12
-	SMD_0394	35	2,50	29	5,18
-	SMD_0395	109	2,59	84	3,91
-	SMD_0396	42	4,13	96	3,24
-	SMD_0397	78	2,91	77	3,01
-	SMD_0398	102	2,43	88	3,21
glnE	SMD_0399	43	2,80	21	4,63
-	SMD_0400	96	3,70	102	3,59
-	SMD_0406	44	4,01	64	2,88
-	SMD_0407	149	2,74	319	2,85
-	SMD_0408	58	3,44	54	3,14
-	SMD_0409	39	4,34	18	5,33
-	SMD_0410	38	3,62	38	3,91
aceE2	SMD_0412	790	2,30	1121	2,40
-	SMD_0413	25	1,73	20	1,90
-	SMD_0417	32	2,50	31	3,16
-	SMD_0418	39	1,56	28	3,17
-	SMD_0419	102	1,37	43	1,57
-	SMD_0420	72	2,22	55	3,39
-	SMD_0428	36	3,85	30	2,74
-	SMD_0431	41	3,61	18	4,67
-	SMD_0445	36	3,50	47	3,86

-	SMD_0446	19	5,94	27	4,65
-	SMD_0447	37	3,18	25	3,88
-	SMD_0448	53	1,46	43	2,66
-	SMD_0454	62	4,41	63	5,10
-	SMD_0455	228	2,81	379	2,30
-	SMD_0456	579	2,03	899	2,25
-	SMD_0457	625	2,33	719	2,30
-	SMD_0458	73	2,24	133	2,66
-	SMD_0459	33	2,58	34	2,82
-	SMD_0462	18	3,05	16	4,62
-	SMD_0463	34	2,72	27	4,22
-	SMD_0464	31	3,00	29	2,10
-	SMD_0467	75	2,68	148	2,65
-	SMD_0469	25	1,69	18	5,06
-	SMD_0472	73	3,36	65	4,16
-	SMD_0474	213	3,15	193	3,74
-	SMD_0475	176	2,72	207	2,55
-	SMD_0476	561	2,46	1081	2,13
motB	SMD_0477	20	3,23	21	3,36
motA	SMD_0478	37	3,33	28	4,15
msrB	SMD_0479	47	3,43	53	3,03
-	SMD_0480	22	4,30	17	4,11
dadA	SMD_0483	59	3,52	57	3,87
-	SMD_0484	166	2,96	142	2,99
-	SMD_0485	228	2,42	169	2,95
rsmC	SMD_0489	37	3,86	51	3,92
rsuA	SMD_0490	200	2,79	212	2,98
-	SMD_0491	337	2,38	352	2,20
fabB	SMD_0492	265	2,33	434	2,32
fabA	SMD_0493	251	2,19	438	2,23
-	SMD_0498	62	3,24	87	4,19
gph	SMD_0499	122	3,64	92	3,71
-	SMD_0500	174	3,03	247	3,05
-	SMD_0501	110	2,86	68	3,67
-	SMD_0503	97	4,04	87	4,17

-	SMD_0508	32	2,53	29	2,55
-	SMD_0510	50	2,99	42	3,56
rstA	SMD_0511	138	2,91	53	4,23
-	SMD_0512	162	2,29	76	2,77
-	SMD_0513	135	3,43	80	2,79
ptpS	SMD_0514	110	2,28	58	4,33
-	SMD_0516	20	4,66	20	5,91
rhlE	SMD_0517	97	5,70	74	6,09
-	SMD_0519	123	2,29	140	3,09
-	SMD_0520	157	2,91	148	3,46
-	SMD_0521	77	2,85	30	5,49
-	SMD_0522	20	6,61	18	9,31
-	SMD_0524	69	2,73	89	3,25
-	SMD_0525	86	3,47	124	2,73
-	SMD_0526	75	11,67	96	11,74
cbsB	SMD_0527	243	2,67	341	2,57
metC	SMD_0528	278	2,64	396	2,47
wzm	SMD_0529	289	2,06	364	2,34
wzt	SMD_0530	763	2,20	1003	2,32
-	SMD_0531	689	2,25	936	2,45
wxocA	SMD_0532	838	2,09	966	2,23
-	SMD_0533	1054	1,96	1415	1,84
-	SMD_0534	458	2,05	449	2,25
-	SMD_0535	514	1,75	688	1,89
-	SMD_0537	18	3,91	31	4,74
-	SMD_0540	47	2,57	45	3,47
-	SMD_0541	240	2,05	217	2,62
etfA	SMD_0542	171	2,56	314	3,11
etfB	SMD_0543	217	2,59	399	2,29
rfbB	SMD_0544	488	2,35	613	2,32
rmlA	SMD_0545	573	2,19	646	2,32
rmlC	SMD_0546	996	1,97	1119	2,23
rfbD	SMD_0547	558	2,05	638	2,31
manA	SMD_0548	473	2,12	523	2,30
spgM	SMD_0549	160	2,47	313	2,51

lpsI	SMD_0550	164	2,74	190	3,22
lpsJ	SMD_0551	214	2,20	335	2,52
-	SMD_0552	226	2,60	302	2,05
-	SMD_0554	59	2,65	46	3,56
-	SMD_0556	44	3,11	28	4,60
hns	SMD_0558	123	2,82	320	2,93
proS	SMD_0559	174	3,00	210	3,15
-	SMD_0560	399	5,83	435	11,90
-	SMD_0561	141	2,87	117	3,28
pssA	SMD_0562	54	4,56	53	3,03
-	SMD_0563	27	3,76	38	4,04
-	SMD_0565	68	2,76	67	3,34
valS	SMD_0566	258	2,33	389	2,49
pepA	SMD_0569	302	2,92	452	2,72
-	SMD_0570	44	4,10	57	3,70
-	SMD_0571	84	3,30	79	3,15
-	SMD_0572	115	2,73	52	3,61
dsbC	SMD_0574	32	3,38	38	4,16
purL	SMD_0576	144	2,55	171	2,59
xpsF	SMD_0581	73	2,45	48	3,06
xpsG	SMD_0582	69	1,54	62	3,32
xpsH	SMD_0583	49	2,71	58	2,89
xpsJ	SMD_0585	44	2,27	31	3,61
pefK	SMD_0586	88	2,71	71	2,74
pefL	SMD_0587	95	2,51	104	2,62
xpsN	SMD_0589	56	2,44	46	2,81
xpsD	SMD_0590	75	2,26	83	2,84
-	SMD_0592	58	3,19	52	3,08
pncB	SMD_0593	56	3,59	48	3,84
fimA	SMD_0595	2106	2,45	3844	2,27
-	SMD_0596	442	3,39	362	3,77
mrkC	SMD_0597	464	2,55	404	2,96
-	SMD_0598	613	2,11	530	2,45
wecB	SMD_0599	62	3,01	50	3,12
-	SMD_0605	194	2,99	247	3,07

-	SMD_0606	128	4,46	213	5,14
glyA	SMD_0607	161	3,32	185	3,13
-	SMD_0608	122	2,77	105	2,45
-	SMD_0609	82	2,72	54	3,08
nrdR	SMD_0610	36	3,73	29	3,58
ribD	SMD_0611	87	3,08	48	4,61
-	SMD_0612	117	3,05	131	3,10
-	SMD_0613	138	2,96	182	3,21
-	SMD_0614	113	4,02	98	4,37
ribE	SMD_0615	18	3,56	20	4,17
ribB	SMD_0616	24	3,20	50	3,49
ribH	SMD_0617	198	3,30	209	3,04
thiL	SMD_0619	42	3,48	74	2,10
-	SMD_0629	30	2,73	21	4,04
-	SMD_0633	45	5,60	20	8,75
-	SMD_0634	28	6,31	38	6,06
mraZ	SMD_0635	232	3,40	147	4,03
mraW	SMD_0636	149	3,05	102	3,34
ftsI	SMD_0638	191	2,81	157	2,93
murE	SMD_0639	242	2,33	269	2,40
murF	SMD_0640	372	2,18	368	2,27
mraY	SMD_0641	305	1,90	405	2,11
ftsW	SMD_0642	369	2,04	452	2,31
murG	SMD_0643	244	2,09	300	2,30
murC	SMD_0644	540	2,05	827	2,01
ddlB	SMD_0645	774	2,00	1124	2,00
ftsQ	SMD_0646	628	1,89	930	2,01
ftsA	SMD_0647	794	1,93	1116	1,82
ftsZ	SMD_0648	888	3,28	1631	2,92
lpxC	SMD_0649	369	2,18	388	2,14
-	SMD_0650	98	3,42	125	2,81
secA	SMD_0651	234	2,60	217	2,78
-	SMD_0653	49	2,87	56	3,78
metF	SMD_0654	16	3,71	28	4,36
-	SMD_0658	79	2,96	89	2,99

-	SMD_0660	30	3,42	33	3,20
-	SMD_0662	34	3,30	29	3,05
sahH	SMD_0664	860	2,37	1775	2,22
-	SMD_0665	93	2,19	131	2,15
ppc	SMD_0666	65	2,96	47	3,20
-	SMD_0667	138	3,29	100	3,31
metK	SMD_0668	299	2,61	662	2,22
-	SMD_0671	62	2,29	44	3,95
-	SMD_0672	38	2,71	22	4,23
rbsK	SMD_0675	195	2,59	281	2,56
-	SMD_0676	90	2,62	93	3,75
-	SMD_0677	26	2,91	38	2,98
-	SMD_0681	109	1,95	92	1,97
tesA	SMD_0683	42	3,67	42	4,05
-	SMD_0684	244	2,76	223	3,31
-	SMD_0685	61	3,52	51	3,57
dgkA	SMD_0687	52	2,90	72	2,90
-	SMD_0692	32	3,28	20	1,84
lgt	SMD_0693	92	2,78	66	3,85
thyA	SMD_0694	107	2,72	120	2,88
-	SMD_0696	141	2,21	83	3,84
apaH	SMD_0697	295	2,74	299	2,44
-	SMD_0698	309	2,00	224	2,48
ksgA	SMD_0699	93	2,65	127	2,41
surA	SMD_0701	514	2,59	602	2,72
ostA	SMD_0702	229	2,24	219	2,75
-	SMD_0705	64	3,43	70	4,29
ubiH	SMD_0706	76	3,17	60	2,82
ubiF	SMD_0707	134	2,83	108	3,28
-	SMD_0709	113	2,08	164	2,80
-	SMD_0710	99	2,57	167	2,77
-	SMD_0711	81	2,83	103	2,50
glnS	SMD_0713	120	3,04	167	3,17
-	SMD_0714	35	3,08	46	4,70
msrA2	SMD_0715	47	2,59	62	3,38

talB	SMD_0716	263	2,49	505	2,46
rnk	SMD_0717	185	2,53	273	2,61
oxyR	SMD_0718	58	3,56	66	3,52
ahpF	SMD_0719	68	3,58	68	3,86
ahpC	SMD_0720	929	4,72	1522	5,18
hemK	SMD_0721	18	3,52	34	4,25
-	SMD_0726	56	3,16	40	3,13
-	SMD_0727	122	2,46	120	2,90
nudF	SMD_0728	110	2,71	125	2,86
exo	SMD_0729	49	2,61	46	3,83
-	SMD_0730	21	3,50	40	4,70
-	SMD_0731	69	2,89	53	3,46
-	SMD_0733	28	3,98	31	4,76
-	SMD_0734	22	3,54	23	4,60
-	SMD_0739	129	3,59	73	4,80
ilvE	SMD_0740	191	2,75	241	2,81
StmPr1	SMD_0741	114	3,19	120	3,29
-	SMD_0742	164	3,38	201	3,33
-	SMD_0745	46	2,76	28	4,01
-	SMD_0746	145	2,81	221	2,85
-	SMD_0747	61	2,93	48	3,87
prfA	SMD_0749	96	2,47	86	3,14
-	SMD_0751	67	2,64	51	3,61
-	SMD_0752	140	2,67	209	2,43
ipk	SMD_0753	172	2,43	172	2,84
prs	SMD_0755	92	3,48	67	3,70
rplY	SMD_0756	1913	2,72	3220	2,83
pth	SMD_0757	651	2,28	500	2,52
ychF	SMD_0758	561	2,40	961	2,33
-	SMD_0759	18	3,86	27	3,53
-	SMD_0764	28	3,37	27	4,46
-	SMD_0766	55	3,11	62	3,38
tuf	SMD_0770	1951	2,56	3723	2,46
-	SMD_0772	1049	2,65	630	3,41
nusG	SMD_0773	630	2,67	421	3,92

rplK	SMD_0774	6335	2,31	9792	2,40
rplA	SMD_0775	3986	2,32	7552	2,35
rplJ	SMD_0776	5485	3,25	10351	2,98
rplL	SMD_0777	4959	3,03	8896	2,93
rpoB	SMD_0778	1626	2,35	3016	2,33
rpoC	SMD_0779	1386	2,11	2672	2,11
rpsL	SMD_0780	924	2,14	1681	2,13
rpsG	SMD_0781	3084	2,04	5774	2,16
fusA	SMD_0782	2995	2,20	5438	2,11
tuf2	SMD_0783	3393	2,18	8966	2,04
rpsJ	SMD_0784	5023	3,56	11156	2,88
rplC	SMD_0785	7618	3,53	14448	2,98
rplD	SMD_0786	9670	3,34	17878	2,88
rplW	SMD_0787	6214	3,57	13221	2,89
rplB	SMD_0788	6089	3,55	13677	2,85
rpsS	SMD_0789	3555	3,70	7768	2,89
rplV	SMD_0790	4270	3,58	8238	2,94
rpsC	SMD_0791	5553	3,55	10846	2,95
rplP	SMD_0792	7295	3,51	15078	2,86
rpmC	SMD_0793	7953	3,41	17482	2,81
rpsQ	SMD_0794	2944	3,43	5621	2,84
rplN	SMD_0795	5560	3,34	11827	2,87
rplX	SMD_0796	4741	3,43	8982	2,90
rplE	SMD_0797	7128	3,48	14344	2,86
rpsN	SMD_0798	2022	3,85	4662	2,99
rpsH	SMD_0799	3126	2,58	3918	2,49
rplF	SMD_0800	5294	2,49	7208	2,44
rplR	SMD_0801	898	2,77	1525	2,23
rpsE	SMD_0802	3556	2,19	5586	2,13
rpmD	SMD_0803	7366	2,10	13787	2,08
rplO	SMD_0804	5802	2,06	9956	2,11
secY	SMD_0805	872	2,26	1073	2,36
rpsM	SMD_0806	2591	2,08	5240	2,05
rpsK	SMD_0807	4871	2,16	10334	2,02
rpsD	SMD_0808	4241	2,15	7180	2,06

rpoA	SMD_0809	1923	2,32	3177	2,29
rplQ	SMD_0810	2811	2,09	4174	2,05
dsbB	SMD_0811	77	3,45	104	2,65
-	SMD_0812	111	3,12	96	3,48
aroH	SMD_0813	54	3,43	57	3,68
-	SMD_0814	28	3,91	24	3,83
-	SMD_0815	45	3,30	50	3,91
typA	SMD_0817	526	2,57	696	2,50
ppiB	SMD_0818	651	2,54	1599	2,53
mdh	SMD_0819	969	2,41	1879	2,42
rluA	SMD_0820	41	3,18	33	3,21
prpE	SMD_0821	78	2,61	101	2,89
-	SMD_0822	51	4,06	39	6,13
-	SMD_0823	104	3,36	92	4,01
gst	SMD_0824	112	2,96	155	3,23
-	SMD_0825	91	4,26	76	4,22
-	SMD_0828	17	4,65	25	4,13
-	SMD_0829	10827	3,24	29153	3,37
-	SMD_0830	20	5,37	54	4,29
rluB2	SMD_0831	75	2,93	99	2,99
-	SMD_0832	450	2,06	749	2,29
kbl	SMD_0833	262	2,70	323	2,98
-	SMD_0834	223	3,36	265	3,72
tdh	SMD_0835	147	2,57	215	2,41
-	SMD_0836	135	2,26	191	2,43
-	SMD_0837	120	2,79	105	2,70
folC	SMD_0838	74	2,80	83	2,97
-	SMD_0840	170	2,78	153	3,36
purF	SMD_0841	151	2,77	174	2,90
-	SMD_0842	76	2,55	68	2,56
-	SMD_0843	47	3,04	29	3,74
-	SMD_0844	139	2,88	117	3,44
lpxH	SMD_0845	44	3,81	52	4,15
-	SMD_0848	59	3,05	39	3,88
ppx	SMD_0849	71	4,15	54	5,24

ppk	SMD_0850	44	3,03	37	3,27
-	SMD_0853	33	3,35	38	3,33
grxC	SMD_0854	144	2,27	179	3,06
-	SMD_0856	380	2,07	838	2,38
-	SMD_0859	33	3,98	27	4,20
-	SMD_0860	42	3,49	25	4,15
-	SMD_0861	146	3,41	320	2,65
tig	SMD_0869	404	2,85	505	2,94
clpP	SMD_0870	307	2,56	492	2,62
clpX	SMD_0871	579	2,34	589	2,78
lon	SMD_0872	296	2,06	259	2,61
hupB	SMD_0873	1382	2,34	1876	2,12
ppiD	SMD_0879	234	2,72	298	2,80
-	SMD_0881	61	3,77	59	3,68
-	SMD_0882	47	3,13	27	3,57
-	SMD_0883	62	3,93	77	3,45
-	SMD_0885	178	2,16	146	2,37
-	SMD_0886	230	2,35	167	2,85
-	SMD_0887	61	2,33	36	2,65
-	SMD_0893	51	1,48	36	1,64
-	SMD_0914	20	2,89	29	2,70
mltD	SMD_0933	89	4,61	76	5,30
gloB	SMD_0934	44	5,04	26	6,16
-	SMD_0935	80	3,43	90	3,37
rnhA	SMD_0936	143	2,94	125	3,28
-	SMD_0938	153	2,33	136	3,09
-	SMD_0940	165	2,94	126	2,24
-	SMD_0941	153	3,05	93	3,85
-	SMD_0942	45	2,86	49	3,25
-	SMD_0961	81	2,31	128	2,62
-	SMD_0962	20	3,41	48	2,67
dnaX	SMD_0964	109	2,70	79	3,08
-	SMD_0965	394	2,65	1018	2,26
-	SMD_0967	17	3,76	85	2,37
-	SMD_0968	105	3,50	133	3,37

-	SMD_0969	32	3,25	29	2,65
-	SMD_0970	25	3,12	16	2,87
-	SMD_0972	39	3,61	37	3,60
-	SMD_0973	97	2,56	98	2,90
-	SMD_0974	414	2,70	167	4,03
rpmF	SMD_0975	1121	2,54	1905	2,64
fabH2	SMD_0976	389	2,34	317	2,78
-	SMD_0977	140	3,48	123	3,22
fabD	SMD_0978	207	3,06	170	3,08
fabG	SMD_0979	375	2,72	360	2,89
acpP	SMD_0980	1570	2,16	2557	2,08
fabF	SMD_0981	441	2,25	551	2,66
yceG	SMD_0983	122	2,94	86	3,09
holB	SMD_0985	108	2,29	95	1,95
pilZ	SMD_0986	105	2,83	120	3,16
-	SMD_0990	121	2,61	38	2,35
-	SMD_0991	47	2,79	33	3,58
-	SMD_0992	77	3,74	67	4,29
-	SMD_0994	26	2,58	69	2,50
qnrR	SMD_0997	20	1,64	18	2,70
-	SMD_0999	33	2,23	30	4,04
-	SMD_1006	52	2,78	50	3,13
-	SMD_1007	51	2,66	27	2,97
soxR	SMD_1008	21	3,11	19	1,76
proC	SMD_1011	37	2,66	39	2,66
pilT	SMD_1013	32	3,66	38	4,27
-	SMD_1015	32	3,04	40	3,27
-	SMD_1018	92	3,16	49	4,02
tag	SMD_1019	70	3,75	79	3,19
yqgE	SMD_1021	45	3,96	54	3,54
-	SMD_1022	44	3,65	27	7,11
pyrB	SMD_1023	101	3,07	99	2,95
-	SMD_1024	154	3,19	217	3,15
-	SMD_1025	41	4,77	41	5,21
mgtE	SMD_1026	38	3,52	69	3,50

ptsI	SMD_1027	64	2,81	66	2,69
-	SMD_1030	79	2,65	97	2,71
-	SMD_1031	82	2,59	32	3,65
-	SMD_1032	82	3,34	59	2,65
yhbH	SMD_1033	305	2,73	455	2,59
rpoN	SMD_1034	105	2,64	85	2,64
lptB	SMD_1035	136	2,50	182	2,60
-	SMD_1036	118	2,75	122	3,22
-	SMD_1037	41	2,41	36	2,24
kdsC	SMD_1038	87	2,60	62	3,51
kdsD	SMD_1039	69	3,01	65	3,62
-	SMD_1040	83	2,56	67	2,73
murA	SMD_1041	92	2,88	68	2,88
-	SMD_1043	93	2,89	63	2,01
purN	SMD_1045	43	3,46	51	3,38
purM	SMD_1049	85	3,48	105	3,64
-	SMD_1050	39	3,82	49	3,78
-	SMD_1051	114	2,90	102	3,32
-	SMD_1052	153	2,43	128	2,33
-	SMD_1053	90	1,82	99	2,79
-	SMD_1054	79	2,28	100	2,80
-	SMD_1055	54	1,49	133	2,46
-	SMD_1056	127	2,51	288	2,34
-	SMD_1057	4146	9,92	7805	8,73
-	SMD_1059	31	1,80	26	2,93
-	SMD_1060	145	2,82	66	3,35
-	SMD_1061	81	2,48	74	2,80
apbE	SMD_1062	141	2,59	203	2,09
-	SMD_1063	120	2,35	200	2,43
-	SMD_1064	67	2,78	103	2,61
piuC	SMD_1068	101	1,78	65	2,03
-	SMD_1070	320	2,38	497	2,13
-	SMD_1071	41	2,98	26	3,35
-	SMD_1073	45	2,87	63	3,14
-	SMD_1075	119	1,95	148	2,39

sufD	SMD_1076	91	2,56	140	2,38
sufB	SMD_1078	66	3,38	53	3,99
-	SMD_1079	23	3,39	16	4,50
-	SMD_1080	81	4,01	107	3,45
-	SMD_1081	71	3,99	42	5,05
-	SMD_1082	25	3,66	19	5,71
-	SMD_1085	1688	2,60	2448	2,57
osmC	SMD_1086	245	2,74	232	3,61
phzF	SMD_1087	57	3,41	58	2,53
lysA	SMD_1089	136	2,79	126	2,87
-	SMD_1090	231	2,05	269	2,25
murD	SMD_1091	168	2,19	195	1,89
-	SMD_1092	183	2,34	85	3,33
ispB	SMD_1096	49	3,51	80	2,98
ssb	SMD_1108	446	2,47	467	2,62
-	SMD_1113	26	1,62	25	1,70
-	SMD_1120	23	2,72	17	3,50
-	SMD_1130	39	2,78	47	2,83
-	SMD_1131	44	3,54	36	4,09
-	SMD_1142	63	2,82	30	3,82
-	SMD_1143	195	2,29	210	2,83
mqo	SMD_1155	30	3,30	21	4,35
-	SMD_1158	35	6,17	52	6,54
-	SMD_1159	125	3,40	105	4,17
-	SMD_1160	64	2,99	56	4,05
-	SMD_1162	87	2,58	118	2,99
-	SMD_1163	172	2,41	269	2,46
-	SMD_1164	238	2,50	197	2,58
-	SMD_1165	269	2,32	321	2,32
minE	SMD_1166	669	2,28	878	2,16
minD	SMD_1167	746	2,35	955	2,48
minC	SMD_1168	134	3,02	135	3,00
-	SMD_1169	49	2,26	35	3,95
-	SMD_1172	835	2,47	996	2,60
-	SMD_1173	74	2,38	50	2,24

-	SMD_1174	86	2,34	63	3,04
-	SMD_1180	85	2,77	89	3,15
-	SMD_1182	17	4,58	103	4,47
-	SMD_1184	67	2,68	70	4,35
tesB	SMD_1185	42	2,71	35	3,79
-	SMD_1186	47	2,02	71	2,82
-	SMD_1187	244	2,48	138	2,96
-	SMD_1188	291	2,52	259	2,31
-	SMD_1189	353	2,29	512	2,31
uvrA	SMD_1190	165	2,50	158	2,84
rplU	SMD_1191	4568	2,45	7731	2,19
rpmA	SMD_1192	2549	2,46	4726	2,31
obg	SMD_1193	302	2,00	276	3,01
rpsT	SMD_1194	553	2,50	809	2,47
murJ	SMD_1195	157	2,64	115	2,78
ribF	SMD_1196	108	2,56	120	3,14
ileS	SMD_1197	219	2,39	377	2,29
lspA	SMD_1198	251	1,82	294	2,35
ispH	SMD_1199	369	2,20	371	2,24
-	SMD_1200	55	2,33	53	2,44
-	SMD_1202	175	2,27	160	2,81
-	SMD_1203	68	2,45	71	2,70
-	SMD_1204	38	2,93	37	3,10
-	SMD_1211	80	3,35	21	5,17
cyoA	SMD_1213	542	2,45	956	2,22
cyoB	SMD_1214	1127	2,19	2155	2,09
cyoC	SMD_1215	1006	2,14	2031	1,99
cyoD	SMD_1216	337	1,73	675	2,28
radA	SMD_1218	64	3,50	74	4,57
hsp90xo	SMD_1219	62	2,81	62	3,44
-	SMD_1220	218	2,26	255	2,24
-	SMD_1221	351	2,19	321	2,34
-	SMD_1222	98	2,66	38	3,67
ffh	SMD_1223	121	2,71	112	3,20
-	SMD_1224	23	2,62	22	3,14

-	SMD_1225	29	3,29	23	2,29
rpsP	SMD_1226	938	2,64	1133	2,72
rimM	SMD_1227	203	2,69	198	3,08
rplS	SMD_1229	1081	2,88	1658	2,81
-	SMD_1232	20	4,29	24	3,47
-	SMD_1234	54	5,01	39	4,51
-	SMD_1236	179	2,85	178	3,31
katA2	SMD_1237	93	3,18	86	3,60
mutS	SMD_1238	45	3,18	52	3,47
-	SMD_1241	182	2,66	63	4,25
-	SMD_1243	29	2,46	22	3,42
-	SMD_1245	39	3,13	32	4,09
-	SMD_1249	25	5,52	32	3,83
-	SMD_1251	118	2,32	68	2,12
-	SMD_1252	169	1,88	157	2,53
dat	SMD_1253	365	2,14	313	2,05
-	SMD_1256	57	2,87	31	3,06
-	SMD_1257	62	2,47	24	3,17
-	SMD_1258	77	2,47	49	2,59
-	SMD_1260	26	1,68	20	3,65
nfi	SMD_1267	26	3,72	24	5,10
gpmA	SMD_1268	164	3,09	171	3,27
-	SMD_1269	255	2,18	376	2,20
mfd	SMD_1271	121	2,56	106	2,83
-	SMD_1272	100	2,52	105	4,33
-	SMD_1273	36	2,88	36	3,57
gltX	SMD_1279	255	2,43	259	2,92
-	SMD_1281	97	2,53	94	2,95
-	SMD_1285	38	2,45	52	2,42
-	SMD_1286	1079	2,15	4131	1,71
-	SMD_1290	35	3,47	24	4,05
-	SMD_1291	34	1,51	34	2,68
-	SMD_1293	84	2,94	84	3,36
-	SMD_1295	515	2,64	176	4,48
deaD	SMD_1296	103	3,31	84	3,97

-	SMD_1298	32	2,87	20	4,71
-	SMD_1300	23	3,86	18	3,62
-	SMD_1301	42	4,30	26	6,24
-	SMD_1302	55	4,02	34	6,74
-	SMD_1305	25	3,57	36	3,33
-	SMD_1306	57	2,48	50	2,89
agp	SMD_1307	33	2,71	23	4,65
-	SMD_1308	22	2,30	16	3,67
-	SMD_1309	192	2,13	275	2,48
-	SMD_1310	22	1,64	39	3,35
-	SMD_1311	50	3,28	38	3,98
accA	SMD_1321	374	2,20	507	2,24
-	SMD_1322	220	1,72	270	2,22
dnaE	SMD_1323	181	2,40	171	2,61
rnhB	SMD_1324	211	2,43	278	2,19
lpxB	SMD_1325	185	2,38	210	2,60
lpxA	SMD_1326	329	2,22	485	2,44
fabZ	SMD_1327	507	2,46	621	2,46
lpxD	SMD_1328	200	2,24	192	2,68
-	SMD_1329	419	2,20	539	2,19
-	SMD_1330	190	2,12	167	2,54
dxr	SMD_1331	182	2,01	214	2,35
cdsA	SMD_1332	210	2,40	275	2,62
uppS	SMD_1333	249	2,49	362	2,42
frr	SMD_1334	562	2,29	791	2,62
pyrH	SMD_1335	268	2,49	374	2,68
-	SMD_1336	43	3,63	46	3,17
tsf	SMD_1337	760	2,52	1721	2,63
rpsB	SMD_1338	1396	2,78	2407	2,78
map	SMD_1345	281	2,41	433	2,24
glnD	SMD_1346	123	2,44	121	2,63
dapD	SMD_1347	435	2,12	702	1,98
-	SMD_1348	133	2,34	201	2,38
dapE	SMD_1350	79	2,42	73	2,34
-	SMD_1351	39	2,35	40	1,57

asnB	SMD_1352	83	3,35	101	3,65
-	SMD_1354	33	3,63	24	3,03
bfr3	SMD_1355	173	2,41	318	2,71
parC	SMD_1356	261	2,44	350	2,55
-	SMD_1358	30	2,58	77	3,17
-	SMD_1359	25	3,30	103	2,77
emrA	SMD_1360	74	2,72	227	2,34
emrB	SMD_1361	78	2,49	234	2,67
rnr	SMD_1367	102	2,20	128	2,56
-	SMD_1368	104	2,07	139	1,70
rlmB	SMD_1369	142	2,74	200	2,79
-	SMD_1370	75	2,72	74	2,73
-	SMD_1374	29	3,10	34	2,89
rnt	SMD_1375	64	3,51	53	3,54
-	SMD_1379	21	9,52	53	6,07
nth	SMD_1387	23	1,52	36	1,39
crt2	SMD_1389	49	3,48	70	3,09
-	SMD_1390	1209	2,43	1588	2,49
-	SMD_1391	85	2,85	57	3,68
-	SMD_1392	72	2,63	59	3,05
ampDI	SMD_1393	61	3,52	50	4,94
rlmL	SMD_1395	59	3,00	56	3,02
-	SMD_1397	27	3,12	23	3,27
-	SMD_1400	75	2,89	68	2,99
-	SMD_1403	25	3,03	28	2,44
-	SMD_1406	45	1,87	21	3,15
-	SMD_1407	59	2,97	33	4,47
-	SMD_1410	18	2,48	21	1,75
potF	SMD_1411	239	2,31	604	2,22
potG	SMD_1412	141	2,24	176	2,46
potH	SMD_1413	125	2,38	172	2,47
potI	SMD_1414	133	2,44	119	2,61
-	SMD_1415	70	2,62	50	2,57
corA	SMD_1416	68	2,12	32	2,61
corC	SMD_1418	169	2,12	142	2,65

-	SMD_1420	86	3,04	78	2,37
-	SMD_1421	97	2,20	160	2,57
-	SMD_1422	71	1,91	95	2,51
-	SMD_1425	88	2,51	77	3,20
sspA	SMD_1433	64	2,57	50	2,43
sspB	SMD_1434	80	3,19	53	2,34
nadC	SMD_1437	104	2,30	117	2,89
-	SMD_1438	109	2,96	149	2,90
purE	SMD_1439	52	2,75	57	3,85
purK	SMD_1440	87	2,51	144	3,03
-	SMD_1441	83	2,53	90	2,78
-	SMD_1443	56	3,01	48	4,60
-	SMD_1445	515	2,16	915	2,09
-	SMD_1446	178	2,63	328	2,31
fimU	SMD_1447	61	3,62	39	3,22
pilW	SMD_1449	38	3,09	30	3,41
pilX	SMD_1450	86	2,33	95	2,46
pilY1	SMD_1451	169	2,14	146	2,14
pilE	SMD_1452	164	2,36	202	2,24
uvrB	SMD_1455	32	2,71	25	2,80
-	SMD_1458	54	3,57	27	5,41
-	SMD_1459	31	3,55	31	6,49
-	SMD_1460	57	2,55	42	4,99
-	SMD_1462	37	2,75	38	3,52
-	SMD_1464	100	3,54	99	2,97
lpxK	SMD_1467	117	2,10	161	2,06
kdsB	SMD_1468	102	2,28	139	2,10
-	SMD_1469	79	1,38	77	2,32
-	SMD_1470	93	1,90	79	2,73
uvrC	SMD_1471	111	1,90	79	2,14
pgsA	SMD_1472	136	2,31	146	2,27
smmD2	SMD_1476	21	3,39	18	2,81
-	SMD_1479	73	2,85	58	3,42
-	SMD_1480	35	3,95	44	2,63
-	SMD_1483	76	3,52	31	4,59

copC	SMD_1486	17	5,70	17	11,90
-	SMD_1499	31	1,45	30	3,13
-	SMD_1506	33	4,01	47	2,63
-	SMD_1507	47	3,38	40	4,85
-	SMD_1508	69	2,91	29	2,99
-	SMD_1515	44	2,94	27	3,44
-	SMD_1524	24	1,64	19	1,55
-	SMD_1526	97	2,65	93	3,00
-	SMD_1527	167	2,50	132	3,35
-	SMD_1530	181	2,56	148	3,70
-	SMD_1532	165	2,65	139	2,98
-	SMD_1559	48	3,08	54	3,23
cadA	SMD_1565	49	2,10	66	2,33
czcD	SMD_1566	52	3,28	22	4,30
-	SMD_1578	33	3,00	22	3,50
-	SMD_1583	107	2,15	70	2,57
-	SMD_1585	125	2,38	91	2,07
-	SMD_1586	370	1,62	260	2,05
-	SMD_1591	126	2,42	25	5,35
-	SMD_1592	52	3,54	62	3,16
-	SMD_1593	21	4,07	27	3,56
-	SMD_1619	91	2,25	85	3,29
-	SMD_1622	28	3,56	17	4,71
-	SMD_1627	24	3,20	24	4,55
wbpO	SMD_1633	77	2,65	71	3,15
-	SMD_1634	54	2,34	53	3,25
fkbP	SMD_1637	32	1,59	41	3,89
-	SMD_1639	47	3,20	22	5,01
-	SMD_1640	26	3,24	20	3,28
-	SMD_1641	107	2,72	45	4,42
exoD	SMD_1642	55	2,76	35	3,48
-	SMD_1643	116	3,07	39	4,17
-	SMD_1644	144	2,64	257	2,20
pitA	SMD_1645	184	2,53	268	2,44
-	SMD_1647	53	2,73	28	2,84

parE	SMD_1648	167	2,00	233	2,44
-	SMD_1650	299	4,51	195	6,19
pyrG	SMD_1651	88	2,75	162	2,40
kdsA	SMD_1652	151	2,64	202	2,85
-	SMD_1653	99	2,06	116	2,67
-	SMD_1654	44	1,28	50	1,47
eno	SMD_1655	233	2,60	262	2,81
ftsB	SMD_1656	309	2,46	431	2,61
ispD	SMD_1657	196	2,14	241	2,54
ispF	SMD_1658	140	1,79	131	2,34
truD	SMD_1659	146	2,30	186	1,98
surE	SMD_1661	156	2,99	142	3,24
pcm	SMD_1662	84	3,10	79	2,17
-	SMD_1663	162	2,32	135	2,54
nlpD	SMD_1664	469	2,24	709	2,23
-	SMD_1665	225	2,42	144	3,09
-	SMD_1666	153	2,13	88	2,38
-	SMD_1667	123	1,35	90	3,01
ftsJ	SMD_1668	102	3,11	37	4,00
ftsH	SMD_1669	119	2,48	118	3,02
-	SMD_1670	62	1,30	80	2,65
folP	SMD_1671	56	2,49	80	2,67
hfq	SMD_1673	289	2,54	325	2,54
hflX	SMD_1674	369	2,15	434	2,68
-	SMD_1675	98	2,60	56	3,66
lexA	SMD_1677	140	3,98	61	5,66
recA	SMD_1678	351	2,71	469	2,91
recX	SMD_1679	208	2,97	226	2,42
alaS	SMD_1680	271	2,73	284	2,81
csrA	SMD_1681	245	1,19	382	2,89
-	SMD_1697	19	1,58	31	2,96
-	SMD_1705	152	1,82	108	2,47
-	SMD_1706	158	2,04	107	2,98
-	SMD_1707	64	1,51	57	2,75
-	SMD_1708	59	2,84	35	3,98

dapA	SMD_1709	76	3,31	70	3,46
-	SMD_1710	457	2,51	734	2,38
-	SMD_1711	118	2,85	121	3,49
pcnB	SMD_1714	75	2,72	60	2,65
folK	SMD_1715	59	1,52	33	2,96
panB	SMD_1716	188	2,68	206	2,49
panC	SMD_1717	163	2,78	180	2,60
panD	SMD_1718	412	2,62	562	2,72
pgi	SMD_1719	219	2,46	252	2,71
-	SMD_1721	46	3,42	26	4,85
ispG	SMD_1722	117	2,33	97	3,74
-	SMD_1724	170	2,48	125	2,83
kgdA	SMD_1725	278	1,70	401	2,37
edd	SMD_1726	76	2,33	91	2,28
zwf	SMD_1729	19	3,30	29	3,15
-	SMD_1730	35	3,29	18	3,74
-	SMD_1731	36	3,76	23	3,26
sdhC	SMD_1732	139	3,17	181	3,91
sdhD	SMD_1733	354	2,73	239	2,90
sdhA	SMD_1734	700	2,22	840	2,47
sdhB	SMD_1735	699	1,97	1045	1,92
-	SMD_1738	95	2,57	97	2,66
-	SMD_1740	192	2,70	158	2,89
-	SMD_1741	145	2,86	99	2,63
mutY	SMD_1742	66	2,95	49	2,95
ftsY	SMD_1743	82	2,24	96	3,07
-	SMD_1744	740	2,25	467	3,06
htpG	SMD_1745	675	2,42	555	2,65
coaD	SMD_1747	124	2,86	103	3,10
-	SMD_1748	643	2,26	1329	2,12
fdx	SMD_1749	413	2,33	385	2,67
ggt	SMD_1750	181	2,38	219	2,35
-	SMD_1751	64	2,60	82	2,75
-	SMD_1754	173	3,01	134	3,77
upp	SMD_1755	84	2,87	110	2,95

-	SMD_1761	129	2,64	238	2,59
-	SMD_1780	140	2,74	90	3,98
-	SMD_1781	40	2,62	28	3,00
-	SMD_1784	47	4,04	22	4,29
smpB	SMD_1785	125	2,58	60	3,70
-	SMD_1786	145	3,17	115	4,61
-	SMD_1787	129	3,27	118	3,71
fur	SMD_1788	54	2,66	88	3,08
-	SMD_1789	29	3,32	34	3,06
-	SMD_1790	48	3,21	51	2,73
recN	SMD_1791	50	2,89	45	3,19
hrcA	SMD_1792	35	3,29	21	3,18
grpE	SMD_1793	656	2,20	691	2,38
dnaK	SMD_1794	1689	2,37	1515	2,50
dnaJ	SMD_1795	623	1,93	479	2,45
-	SMD_1796	117	1,31	122	1,42
pdxK	SMD_1798	52	2,95	63	2,84
tyrA	SMD_1799	81	1,38	73	2,30
-	SMD_1801	33	2,83	25	3,07
-	SMD_1802	49	2,74	24	3,10
-	SMD_1805	46	2,29	51	2,39
-	SMD_1806	71	3,45	28	5,52
-	SMD_1807	68	2,28	89	2,79
queA	SMD_1808	57	3,11	49	3,06
tgt	SMD_1809	100	2,83	130	2,61
yajC	SMD_1810	231	2,24	367	2,34
secD	SMD_1811	261	2,49	403	2,53
secF	SMD_1812	455	2,10	535	2,11
-	SMD_1813	338	2,50	368	2,52
-	SMD_1814	125	2,62	104	2,83
cspA	SMD_1819	2378	1,76	4507	1,81
-	SMD_1821	39	2,18	25	2,93
apt	SMD_1822	107	1,99	86	2,53
uup	SMD_1826	99	2,65	83	2,78
-	SMD_1827	99	2,68	56	3,09

-	SMD_1828	26	2,60	35	5,27
smmI	SMD_1831	24	2,59	19	2,73
-	SMD_1832	76	2,25	71	2,85
-	SMD_1834	116	2,40	101	3,28
-	SMD_1835	109	2,90	147	2,74
-	SMD_1836	43	3,07	55	2,14
rpmJ2	SMD_1837	416	2,68	1444	2,09
cmk	SMD_1838	621	2,62	971	2,33
rpsA	SMD_1839	4557	2,06	7217	2,04
ihfB	SMD_1840	829	2,41	496	2,49
-	SMD_1842	211	2,63	171	2,53
wbpL	SMD_1843	149	2,88	141	2,73
wbiI	SMD_1844	146	2,29	120	2,63
galU	SMD_1845	345	2,19	434	2,25
-	SMD_1846	166	2,27	226	2,00
-	SMD_1847	161	1,82	191	2,25
ndk	SMD_1849	767	2,49	1815	2,51
rlmN	SMD_1850	109	2,14	121	2,65
-	SMD_1852	120	2,64	92	2,97
-	SMD_1853	139	2,50	97	2,82
-	SMD_1854	339	2,31	361	2,39
engA	SMD_1855	241	2,03	288	2,45
-	SMD_1856	54	2,00	26	2,48
folD	SMD_1858	47	2,76	66	2,93
guaB	SMD_1859	117	2,60	97	3,14
guaA	SMD_1861	177	2,02	307	2,19
hsdR	SMD_1862	52	2,72	45	3,13
hsdM	SMD_1863	121	2,22	162	2,26
hsdS	SMD_1864	149	1,84	192	1,85
-	SMD_1865	100	2,25	145	2,03
-	SMD_1866	138	2,27	143	1,87
-	SMD_1868	18	1,62	21	3,09
-	SMD_1874	47	2,24	34	3,98
-	SMD_1885	27	2,40	27	2,73
-	SMD_1886	18	3,71	28	3,12

-	SMD_1887	26	3,12	21	1,71
-	SMD_1889	17	1,44	28	2,70
-	SMD_1913	114	1,93	107	2,19
murB	SMD_1914	137	1,94	184	2,11
pyrD	SMD_1915	53	2,73	98	2,65
-	SMD_1917	60	3,19	23	4,97
-	SMD_1918	65	2,48	57	2,17
-	SMD_1919	196	2,40	441	2,32
-	SMD_1923	76	2,65	34	4,09
-	SMD_1924	57	3,00	36	3,49
-	SMD_1926	76	2,42	60	2,48
-	SMD_1927	197	2,04	158	2,51
-	SMD_1929	144	2,34	172	2,40
-	SMD_1934	36	2,20	30	3,26
-	SMD_1935	51	3,03	17	4,32
thrB	SMD_1940	19	2,29	29	2,74
thrC	SMD_1941	32	2,96	51	2,96
hisS	SMD_1943	177	2,63	184	2,73
hisB	SMD_1949	31	1,43	29	1,76
hisA	SMD_1951	25	2,35	72	1,80
hisF	SMD_1952	57	2,50	61	1,52
-	SMD_1954	46	3,16	45	2,51
-	SMD_1956	52	2,31	42	2,99
-	SMD_1957	133	2,41	130	2,44
cutC	SMD_1958	58	2,08	67	2,19
actP	SMD_1960	22	1,99	24	2,87
-	SMD_1963	209	2,55	191	2,80
-	SMD_1964	116	2,32	97	2,41
-	SMD_1965	182	2,12	127	2,32
-	SMD_1966	159	2,01	207	1,93
scrK	SMD_1967	296	2,04	343	2,04
-	SMD_1968	305	2,01	370	1,86
bmnA	SMD_1969	89	2,28	92	2,06
masA	SMD_1970	179	2,92	255	2,31
mtnD	SMD_1971	71	3,29	84	2,62

mtnB	SMD_1972	83	2,54	75	3,03
-	SMD_1973	73	1,87	105	2,09
-	SMD_1974	72	2,39	85	2,83
serA	SMD_1976	72	2,80	62	3,12
-	SMD_1977	26	2,98	25	3,40
-	SMD_1978	65	1,44	46	2,97
-	SMD_1979	64	2,43	79	2,67
-	SMD_1980	109	2,44	92	2,59
-	SMD_1981	37	3,01	42	2,50
smeZ	SMD_1983	104	1,98	75	2,24
-	SMD_1984	140	1,64	91	2,16
-	SMD_1985	440	2,34	385	2,29
efp2	SMD_1986	553	2,13	730	2,47
-	SMD_1987	124	2,95	103	3,08
hmgcL	SMD_1988	37	2,55	58	2,96
-	SMD_1990	20	1,44	30	2,28
feoA	SMD_1991	40	2,87	21	1,73
feoB	SMD_1992	62	2,36	40	2,60
-	SMD_1993	77	1,25	65	2,73
-	SMD_1994	422	2,09	233	2,37
dapB	SMD_1998	58	2,37	54	2,32
carA	SMD_1999	144	2,78	132	2,86
carB	SMD_2000	266	2,11	349	2,36
greA	SMD_2001	563	2,21	866	2,29
-	SMD_2002	201	2,23	246	2,22
-	SMD_2003	65	2,52	36	3,21
recJ	SMD_2004	30	3,06	23	3,08
prfB	SMD_2008	366	2,07	463	2,44
-	SMD_2009	100	1,31	95	2,96
ilvK	SMD_2012	35	2,88	30	3,32
lysS	SMD_2013	271	2,67	321	2,66
rpfG	SMD_2014	102	2,61	121	2,45
rpfC	SMD_2015	93	2,63	93	2,59
rpfF	SMD_2016	264	3,36	213	3,90
fadD	SMD_2017	63	2,97	61	3,55

betA	SMD_2018	46	2,84	55	2,85
-	SMD_2021	68	2,07	41	2,22
acn	SMD_2022	162	2,49	170	2,57
acnB	SMD_2025	726	2,27	1326	2,28
-	SMD_2032	183	2,19	486	2,89
cheW	SMD_2033	70	1,27	91	2,55
-	SMD_2035	22	3,52	27	2,65
cheA	SMD_2036	62	2,30	75	2,47
-	SMD_2038	93	1,37	132	2,18
-	SMD_2039	69	2,51	101	2,27
-	SMD_2040	50	2,37	65	2,95
motB2	SMD_2041	47	2,57	82	2,40
cheA2	SMD_2043	105	2,36	145	2,29
cheZ	SMD_2044	246	1,75	450	1,96
cheY2	SMD_2045	239	1,31	355	2,44
fliA	SMD_2046	98	2,23	210	2,37
fleN	SMD_2047	161	2,45	314	2,06
flhF	SMD_2048	99	2,29	190	2,11
flhA	SMD_2049	28	2,30	46	1,81
fliP	SMD_2054	34	1,66	77	2,08
fliM	SMD_2057	96	2,20	206	2,21
fliK	SMD_2059	48	2,06	128	2,12
fliI	SMD_2061	74	1,87	151	2,14
fliH	SMD_2062	97	2,36	158	2,06
fliG	SMD_2063	213	1,23	333	1,94
fliF	SMD_2064	67	1,41	134	2,21
rpoN2	SMD_2069	31	3,18	24	3,78
-	SMD_2070	31	3,37	44	3,03
-	SMD_2071	31	3,02	75	1,40
-	SMD_2072	121	2,49	173	2,22
fliS	SMD_2073	128	2,21	192	2,02
fliD	SMD_2074	81	2,24	156	2,27
fliC2	SMD_2075	299	2,47	650	3,05
fliC+M24	SMD_2076	195	2,56	308	2,99
fliC3	SMD_2077	42	3,50	38	3,67

flgJ	SMD_2078	89	2,04	185	1,89
flgL	SMD_2079	130	2,23	329	1,88
flgH	SMD_2082	138	1,94	229	1,97
flgG	SMD_2083	204	2,17	435	1,84
flgF	SMD_2084	90	2,06	248	1,97
flgE	SMD_2085	151	2,62	553	4,02
flgC	SMD_2087	70	2,26	111	1,93
flgA	SMD_2090	30	1,86	45	1,51
-	SMD_2099	45	2,04	32	2,84
-	SMD_2100	69	2,33	81	2,14
mnmA	SMD_2101	167	2,49	161	2,23
-	SMD_2102	69	1,44	97	2,66
-	SMD_2105	49	2,59	21	3,13
clpA	SMD_2106	71	2,67	68	3,35
-	SMD_2107	31	3,28	20	1,69
infA	SMD_2108	255	2,02	231	2,53
-	SMD_2110	41	3,55	33	3,33
-	SMD_2111	79	2,86	57	3,29
trxB	SMD_2112	178	2,85	267	2,50
ftsK	SMD_2113	97	2,58	85	3,06
-	SMD_2114	206	2,09	236	2,28
lolA	SMD_2115	186	2,21	264	2,45
-	SMD_2120	32	2,92	19	2,52
-	SMD_2121	24	1,98	17	1,97
-	SMD_2127	306	2,48	254	2,92
-	SMD_2129	93	3,51	90	3,42
cusA3	SMD_2133	29	2,42	28	2,39
-	SMD_2134	60	2,77	69	1,98
-	SMD_2135	168	2,40	178	2,43
lspA2	SMD_2136	99	1,14	65	1,34
-	SMD_2137	207	1,86	227	1,90
glnB2	SMD_2138	332	1,52	415	1,91
cusA2	SMD_2139	298	2,03	379	1,93
cusB2	SMD_2140	332	1,83	318	1,91
smmJ2	SMD_2141	238	1,86	262	2,03

-	SMD_2142	229	2,16	152	2,30
-	SMD_2143	102	2,63	60	2,71
-	SMD_2144	65	1,91	52	1,83
nadE2	SMD_2145	111	2,20	73	2,10
-	SMD_2146	62	2,32	78	2,19
tnpA	SMD_2147	37	2,23	26	2,99
-	SMD_2150	28	2,50	19	3,16
-	SMD_2163	45	2,32	45	2,39
-	SMD_2164	48	1,49	50	1,36
-	SMD_2165	29	2,51	30	3,17
-	SMD_2170	31	2,62	17	3,98
-	SMD_2177	65	2,92	30	4,18
-	SMD_2187	23	2,88	50	3,00
-	SMD_2193	25	2,79	27	3,24
-	SMD_2220	88	2,18	19	1,70
-	SMD_2224	106	2,73	39	2,84
-	SMD_2225	82	2,09	19	2,72
fruK	SMD_2226	109	1,31	30	1,57
fruA	SMD_2227	194	2,04	49	2,61
rpfN	SMD_2228	229	1,93	51	2,51
-	SMD_2229	269	1,19	41	2,61
-	SMD_2230	39	2,40	20	2,94
-	SMD_2236	37	2,54	36	3,01
-	SMD_2253	39	3,20	24	2,86
-	SMD_2256	33	2,94	36	1,96
metE	SMD_2261	24	4,69	36	3,83
-	SMD_2271	33	2,86	65	3,16
-	SMD_2273	42	1,94	43	3,74
-	SMD_2293	81	4,35	111	4,17
-	SMD_2298	112	2,61	71	3,09
-	SMD_2299	75	2,73	83	2,29
-	SMD_2316	57	2,96	19	2,58
-	SMD_2318	39	3,40	29	4,33
trbP	SMD_2323	23	2,68	23	1,71
-	SMD_2338	52	2,62	49	2,28

-	SMD_2339	42	2,21	40	2,74
-	SMD_2340	54	2,08	50	2,12
-	SMD_2346	22	2,73	18	2,89
-	SMD_2350	18	1,65	39	3,86
-	SMD_2374	45	2,77	18	3,07
-	SMD_2375	29	2,93	30	2,81
-	SMD_2379	119	3,12	63	4,51
proP	SMD_2380	64	3,65	48	4,35
-	SMD_2390	26	3,74	26	3,16
-	SMD_2431	30	2,95	29	1,91
modA	SMD_2433	48	2,26	38	3,19
fnr2	SMD_2434	50	1,78	41	2,70
moeA	SMD_2439	73	2,52	63	2,61
moaBC	SMD_2440	55	2,80	50	1,50
moaA	SMD_2441	27	3,08	23	1,67
-	SMD_2451	21	3,10	30	4,18
-	SMD_2454	26	3,64	28	2,46
-	SMD_2457	66	2,66	66	3,25
-	SMD_2459	23	2,11	29	2,94
-	SMD_2461	92	2,24	95	2,26
sodA2	SMD_2474	69	3,88	94	2,74
-	SMD_2475	300	3,11	293	3,41
-	SMD_2476	491	2,48	244	3,17
-	SMD_2477	99	2,95	79	3,34
-	SMD_2478	83	3,23	53	4,41
-	SMD_2479	45	2,57	39	2,01
-	SMD_2480	56	2,24	53	3,58
-	SMD_2481	63	2,48	36	2,55
-	SMD_2484	55	3,05	49	3,02
-	SMD_2488	32	3,33	26	2,26
-	SMD_2489	25	3,51	16	4,39
-	SMD_2491	29	3,30	25	3,23
-	SMD_2494	85	1,90	64	3,54
-	SMD_2495	94	2,91	149	2,90
-	SMD_2496	56	2,72	16	3,88

-	SMD_2517	26	3,33	21	4,83
-	SMD_2518	30	4,64	20	6,11
-	SMD_2528	31	2,39	27	2,44
-	SMD_2530	31	2,35	22	3,27
-	SMD_2531	27	2,90	21	3,13
cdh	SMD_2539	58	3,59	50	1,92
-	SMD_2540	63	3,15	49	2,78
-	SMD_2541	357	2,64	366	2,84
-	SMD_2542	198	1,25	138	2,52
dld	SMD_2543	442	1,93	464	2,08
lctD	SMD_2544	372	1,91	376	2,23
lctR	SMD_2545	131	2,35	72	2,55
lctP	SMD_2546	61	3,84	34	4,65
cgb	SMD_2554	37	2,48	45	2,53
-	SMD_2560	32	5,54	19	11,09
-	SMD_2568	33	6,66	20	30,29
-	SMD_2569	93	4,44	56	6,08
-	SMD_2574	37	7,96	46	8,86
-	SMD_2575	32	3,84	44	2,41
-	SMD_2576	45	2,64	169	2,34
adi	SMD_2577	86	2,52	391	3,87
-	SMD_2592	44	2,57	32	1,54
-	SMD_2597	111	3,32	44	6,05
tex	SMD_2599	103	2,71	101	2,72
-	SMD_2600	129	3,07	115	2,95
-	SMD_2601	37	2,12	34	3,12
-	SMD_2606	52	2,54	57	2,48
pssA2	SMD_2607	27	3,31	23	2,30
mutX	SMD_2610	67	2,85	39	3,18
pps	SMD_2613	207	2,94	233	2,96
-	SMD_2616	25	4,22	27	3,24
orn	SMD_2617	52	3,33	51	3,33
-	SMD_2620	33	3,21	27	4,45
-	SMD_2621	107	2,70	112	2,99
-	SMD_2623	78	1,96	67	2,53

-	SMD_2624	135	2,89	150	2,66
-	SMD_2626	112	2,58	86	2,47
-	SMD_2628	64	3,35	40	3,17
trwI	SMD_2630	416	1,68	480	1,95
-	SMD_2631	480	1,75	643	1,91
virB4	SMD_2632	540	1,87	642	1,89
-	SMD_2633	529	2,12	884	1,96
-	SMD_2634	988	2,90	1508	2,93
-	SMD_2635	340	2,21	382	2,25
-	SMD_2637	349	2,32	398	2,51
virB9	SMD_2638	314	2,27	313	2,72
-	SMD_2639	91	2,52	47	3,72
-	SMD_2640	91	2,94	42	3,68
virD4	SMD_2641	64	3,11	33	3,48
-	SMD_2645	37	3,10	29	5,77
-	SMD_2651	63	3,10	49	4,50
-	SMD_2652	52	3,40	52	5,04
-	SMD_2661	38	2,58	39	3,11
-	SMD_2663	171	2,56	103	2,84
-	SMD_2665	51	2,34	36	2,77
-	SMD_2671	34	2,63	27	2,92
-	SMD_2674	61	2,65	64	4,14
serS	SMD_2677	79	3,04	105	3,07
-	SMD_2678	25	1,70	17	3,69
aroA	SMD_2680	95	2,59	149	2,65
pheA	SMD_2681	129	2,63	141	2,85
serC	SMD_2682	89	3,17	77	3,57
-	SMD_2683	87	3,90	68	4,26
-	SMD_2684	49	3,38	90	2,23
-	SMD_2685	46	3,05	49	3,66
-	SMD_2686	37	4,76	25	5,32
-	SMD_2687	89	3,51	52	4,51
-	SMD_2688	18	2,78	27	4,49
hutH	SMD_2691	19	3,58	32	3,05
-	SMD_2695	51	4,63	32	6,66

-	SMD_2704	43	2,99	32	2,99
-	SMD_2709	83	1,35	41	3,48
-	SMD_2710	49	2,61	33	3,12
-	SMD_2713	66	2,56	97	2,68
-	SMD_2714	55	2,76	39	2,65
gyrA	SMD_2717	289	2,67	302	2,97
mtnA	SMD_2718	79	2,70	66	3,96
-	SMD_2719	88	3,06	85	3,05
-	SMD_2723	109	2,82	88	3,97
smc	SMD_2725	73	2,51	70	2,98
rplI	SMD_2726	1549	2,96	3022	2,65
rpsR	SMD_2727	1578	2,54	2414	2,49
rpsF	SMD_2728	2735	2,46	4630	2,30
-	SMD_2729	64	3,37	32	4,76
sufA	SMD_2730	52	2,97	46	3,38
asnC	SMD_2731	151	2,87	169	2,88
-	SMD_2733	102	3,27	143	3,26
pai2	SMD_2734	61	3,37	96	2,18
-	SMD_2735	167	2,17	195	2,82
nbaC	SMD_2736	108	2,06	146	2,24
-	SMD_2737	46	2,64	30	2,83
sbcB	SMD_2739	85	2,31	99	2,50
-	SMD_2740	133	2,45	147	2,11
-	SMD_2741	62	3,09	72	2,48
-	SMD_2743	63	2,90	49	3,33
-	SMD_2744	63	2,76	62	2,78
-	SMD_2745	657	2,20	1141	2,14
-	SMD_2746	184	1,99	121	1,94
-	SMD_2747	34	2,40	34	4,20
smeG	SMD_2748	212	2,39	559	2,19
acrD	SMD_2749	325	2,19	882	1,87
-	SMD_2750	111	1,36	43	2,38
bcd	SMD_2751	161	2,15	183	2,72
metH2	SMD_2753	55	3,25	58	3,05
metH	SMD_2754	49	2,42	63	2,55

-	SMD_2755	104	1,89	57	2,84
-	SMD_2758	442	1,92	363	2,75
-	SMD_2759	469	2,26	574	2,40
-	SMD_2760	254	2,92	449	1,99
-	SMD_2765	60	2,37	55	2,58
-	SMD_2766	68	2,31	76	2,26
-	SMD_2767	63	2,02	76	2,94
-	SMD_2768	147	2,53	151	2,93
fumC	SMD_2769	40	3,05	45	3,12
purB	SMD_2770	62	3,24	73	3,13
-	SMD_2772	62	2,59	100	2,49
-	SMD_2773	50	2,27	76	2,87
sucA	SMD_2774	598	2,38	842	2,31
sucB	SMD_2775	1170	2,21	2224	2,02
odhL	SMD_2776	1369	1,99	2388	1,90
-	SMD_2783	38	2,41	53	2,97
-	SMD_2787	71	3,41	52	3,69
-	SMD_2788	114	3,46	163	3,38
-	SMD_2789	114	3,68	90	4,12
yjjV	SMD_2790	202	3,34	198	3,61
-	SMD_2791	86	3,10	84	3,76
-	SMD_2793	282	2,64	377	2,68
cspA2	SMD_2794	291	2,57	399	2,50
-	SMD_2796	65	2,96	71	2,64
gst4	SMD_2798	36	4,24	72	3,06
-	SMD_2800	269	2,57	362	2,44
fpr	SMD_2804	139	3,01	185	2,63
-	SMD_2806	385	2,84	936	2,50
-	SMD_2808	120	2,55	121	3,08
-	SMD_2809	79	2,86	48	3,24
-	SMD_2810	415	2,44	243	2,74
-	SMD_2812	145	2,45	171	2,63
sodA	SMD_2814	1219	2,98	1283	3,06
-	SMD_2815	49	2,60	26	3,44
-	SMD_2817	56	3,29	56	4,22

-	SMD_2818	138	3,55	113	4,83
-	SMD_2820	113	2,26	107	3,54
zupT	SMD_2821	144	2,65	112	2,82
rluC	SMD_2822	96	2,45	74	3,00
rnE	SMD_2823	244	2,48	279	2,71
-	SMD_2825	54	2,65	67	2,79
-	SMD_2829	53	2,52	52	2,71
-	SMD_2830	472	2,20	712	2,24
-	SMD_2831	120	2,83	134	2,78
-	SMD_2832	99	2,25	127	1,39
yciL	SMD_2833	653	2,14	890	2,35
scpA	SMD_2834	22	3,38	18	4,81
scpB	SMD_2835	96	3,17	137	3,14
rluB	SMD_2836	211	2,52	355	2,48
-	SMD_2840	103	2,78	161	2,66
-	SMD_2841	61	2,65	103	2,51
ccmA	SMD_2842	26	2,97	23	4,38
ccmB	SMD_2843	32	3,38	31	3,82
ccmE	SMD_2846	106	2,69	116	2,69
ccmF	SMD_2847	96	2,49	97	2,63
ccmG	SMD_2848	245	2,19	309	2,49
metX	SMD_2851	94	3,27	102	3,43
cydC	SMD_2853	80	2,16	77	2,35
cydD	SMD_2854	19	3,17	19	3,36
cydA	SMD_2855	181	2,61	379	2,20
cydB	SMD_2856	216	1,91	430	1,88
-	SMD_2860	37	3,20	25	3,72
hmsS	SMD_2864	135	2,24	83	2,08
proA	SMD_2865	49	2,72	75	2,34
proB	SMD_2866	44	1,92	94	2,23
-	SMD_2867	317	1,83	414	1,85
argH	SMD_2868	101	2,04	114	2,40
argC	SMD_2869	139	2,16	186	2,40
-	SMD_2871	104	2,06	117	2,84
argE	SMD_2872	93	2,68	183	2,60

argG	SMD_2873	210	2,25	372	2,26
-	SMD_2876	88	4,42	47	8,79
cysS	SMD_2877	86	3,21	85	2,97
-	SMD_2879	43	2,22	70	2,72
-	SMD_2880	295	2,61	322	3,24
pyrC	SMD_2882	123	2,61	104	2,77
-	SMD_2884	222	2,11	200	2,57
ubiG	SMD_2886	104	2,43	116	1,89
-	SMD_2887	151	2,46	146	2,24
efp	SMD_2888	439	2,61	722	2,28
-	SMD_2889	112	3,10	55	4,13
-	SMD_2890	55	3,21	39	3,57
-	SMD_2891	46	3,30	36	4,71
trmJ	SMD_2892	38	3,35	41	3,83
suhB	SMD_2893	58	3,63	26	4,17
htpX	SMD_2894	86	3,61	73	4,06
-	SMD_2897	70	2,78	71	3,54
-	SMD_2898	96	2,48	101	2,75
-	SMD_2899	131	2,26	131	2,86
mutL	SMD_2900	61	3,94	61	4,40
amiC	SMD_2901	113	3,93	110	4,47
ex7L	SMD_2905	23	2,79	30	3,03
-	SMD_2907	35	3,07	21	4,67
-	SMD_2909	98	2,83	125	3,02
-	SMD_2920	60	2,68	75	2,52
dxs	SMD_2922	129	2,76	130	3,64
-	SMD_2923	221	2,78	90	4,00
-	SMD_2929	23	1,54	30	2,66
-	SMD_2933	18	5,09	38	5,95
-	SMD_2935	31	2,75	21	4,97
himA	SMD_2946	857	2,34	809	2,76
pheT	SMD_2947	190	2,51	255	2,69
pheS	SMD_2948	198	2,93	186	3,60
rplT	SMD_2949	1407	2,29	1861	2,45
RL35	SMD_2950	2246	2,45	3648	2,43

infC	SMD_2951	593	1,86	805	2,39
thrS	SMD_2952	293	2,66	413	2,79
chiA2	SMD_2957	32	2,43	36	2,97
pnp	SMD_2958	1198	2,22	2480	2,22
rpsO	SMD_2959	700	2,45	983	2,92
truB	SMD_2960	216	2,38	269	2,01
rbfA	SMD_2961	389	2,23	520	2,39
infB	SMD_2962	798	2,16	1141	2,23
nusA	SMD_2963	269	2,81	290	2,92
rimP	SMD_2964	146	2,42	82	3,92
nuoN	SMD_2967	657	1,98	1079	1,91
nuoM	SMD_2968	923	1,91	1370	1,85
nuoL	SMD_2969	1153	1,80	1864	1,92
nuoK	SMD_2970	710	1,92	1538	2,09
nuoJ	SMD_2971	1371	2,12	2562	2,08
nuoI	SMD_2972	555	1,99	1012	1,66
nuoH	SMD_2973	1133	2,06	1524	2,00
nuoG	SMD_2974	1249	2,02	2006	2,05
nuoF	SMD_2975	1981	2,01	2887	1,91
nuoE	SMD_2976	2270	1,97	3241	1,83
nuoD	SMD_2977	1044	2,19	1323	2,29
nuoC	SMD_2978	449	2,59	556	2,56
nuoB	SMD_2979	417	2,73	385	2,95
nuoA	SMD_2980	176	3,35	120	3,58
secG	SMD_2982	163	3,32	181	4,02
tpiA	SMD_2983	245	3,14	301	3,01
-	SMD_2984	199	2,92	348	2,37
-	SMD_2985	43	3,35	36	3,80
-	SMD_2986	142	2,30	127	2,70
-	SMD_2987	286	2,57	211	2,18
glmM	SMD_2988	457	2,46	584	2,66
accD	SMD_2989	324	2,43	355	2,65
trpA	SMD_2990	54	2,15	56	2,78
-	SMD_2991	47	1,51	49	3,17
trpB	SMD_2992	35	3,53	32	3,39

-	SMD_2993	52	2,41	38	2,85
-	SMD_2994	90	2,41	98	2,61
-	SMD_2995	29	2,22	40	2,59
-	SMD_2996	86	2,36	85	2,60
-	SMD_2997	146	2,37	179	2,37
-	SMD_2998	71	2,13	77	2,46
truA	SMD_3000	289	2,29	325	2,40
-	SMD_3001	278	2,52	365	2,45
fimV	SMD_3002	214	2,70	223	2,59
asd	SMD_3003	255	2,80	363	2,46
-	SMD_3004	157	2,69	149	2,72
aroC	SMD_3005	200	2,58	204	3,18
yfcB	SMD_3006	88	3,03	98	2,98
-	SMD_3007	40	3,12	26	3,97
psd	SMD_3008	82	2,48	66	2,81
-	SMD_3009	315	2,11	289	2,39
mltD	SMD_3010	85	2,53	71	2,80
-	SMD_3011	52	2,98	38	3,76
-	SMD_3014	128	3,10	95	3,48
-	SMD_3015	16	3,21	35	2,91
-	SMD_3016	20	1,61	18	3,92
-	SMD_3018	26	3,83	50	3,03
-	SMD_3019	36	3,22	55	3,57
-	SMD_3020	41	4,53	42	5,54
-	SMD_3021	18	6,54	35	4,99
dcd	SMD_3025	152	3,05	180	3,16
-	SMD_3027	1335	3,06	2769	2,73
-	SMD_3028	38	2,37	33	3,27
-	SMD_3030	227	2,18	342	2,05
-	SMD_3031	254	2,37	320	2,39
-	SMD_3032	51	2,83	45	1,62
-	SMD_3033	84	3,05	52	4,33
-	SMD_3034	64	3,18	36	4,51
-	SMD_3039	43	2,69	36	3,36
slyD	SMD_3040	501	2,44	630	2,43

-	SMD_3042	35	3,75	16	5,21
-	SMD_3043	19	3,32	26	4,22
phoA	SMD_3044	103	2,31	128	2,51
-	SMD_3045	27	1,60	48	1,70
xseB	SMD_3047	168	2,98	98	4,38
ispA	SMD_3048	126	2,98	97	3,67
-	SMD_3049	239	2,80	196	3,36
-	SMD_3050	121	2,30	50	1,57
-	SMD_3051	468	2,29	165	2,42
tldD	SMD_3052	169	2,53	78	3,62
rnG	SMD_3054	93	2,66	57	3,63
maf	SMD_3055	46	2,81	48	4,15
-	SMD_3056	74	3,71	147	4,39
-	SMD_3057	40	2,99	16	4,13
-	SMD_3058	70	3,34	46	4,24
rlmH	SMD_3061	112	3,47	172	2,82
nadD	SMD_3063	122	1,80	122	2,57
holA	SMD_3064	148	2,99	165	3,22
-	SMD_3065	235	2,23	204	2,94
leuS	SMD_3066	257	2,86	312	3,08
-	SMD_3069	49	4,70	57	4,94
-	SMD_3073	35	3,41	33	2,81
-	SMD_3074	17	3,02	24	4,25
metG	SMD_3080	108	2,47	165	2,83
-	SMD_3081	249	3,02	421	2,66
rnfB	SMD_3082	150	2,72	126	2,48
-	SMD_3083	382	2,84	567	3,07
-	SMD_3088	146	2,97	160	3,33
phnA	SMD_3089	222	2,64	657	2,51
yegD	SMD_3090	29	3,40	21	5,00
-	SMD_3091	92	3,54	76	4,82
-	SMD_3092	74	3,68	79	5,83
-	SMD_3093	78	4,69	93	5,55
-	SMD_3097	115	2,92	80	3,57
-	SMD_3098	102	1,36	27	2,36

-	SMD_3099	46	3,40	42	3,45
rluF2	SMD_3101	49	3,45	43	3,62
-	SMD_3102	36	2,99	20	5,57
vdh	SMD_3104	81	3,24	79	2,75
sphB	SMD_3105	37	2,78	36	2,87
-	SMD_3106	67	2,94	124	2,51
-	SMD_3107	3735	4,25	8058	3,60
-	SMD_3108	52	2,39	72	3,36
-	SMD_3114	90	1,39	46	2,53
cspD	SMD_3116	806	2,34	1751	1,97
mtaP	SMD_3117	152	2,88	294	2,63
hpt	SMD_3118	195	2,40	356	2,51
-	SMD_3120	100	2,50	80	3,13
-	SMD_3121	28	2,97	29	3,45
rumA	SMD_3122	76	2,91	68	3,47
-	SMD_3123	168	3,27	139	3,98
recO	SMD_3124	114	2,43	108	2,90
era	SMD_3125	605	2,41	749	2,32
rnc	SMD_3126	321	2,24	303	2,13
-	SMD_3127	379	2,66	338	2,58
lepB	SMD_3128	260	2,07	264	2,61
lepA	SMD_3129	153	2,92	126	2,86
-	SMD_3130	265	2,68	505	2,57
-	SMD_3133	33	2,96	31	3,51
aqpZ	SMD_3135	141	3,18	131	3,90
-	SMD_3137	62	2,74	32	4,78
cstA	SMD_3138	68	2,94	46	3,30
-	SMD_3140	65	2,83	65	3,43
-	SMD_3141	143	2,42	58	3,22
-	SMD_3143	47	3,60	19	3,80
-	SMD_3144	50	2,86	67	3,26
-	SMD_3145	25	3,17	19	2,10
-	SMD_3146	47	2,60	36	3,35
-	SMD_3147	231	2,36	239	2,64
gcvP	SMD_3153	110	2,73	190	2,47

-	SMD_3155	51	3,13	33	3,61
-	SMD_3161	88	3,05	97	3,31
purA	SMD_3164	122	3,36	107	3,56
-	SMD_3165	268	3,03	256	2,93
hflC	SMD_3167	193	2,55	230	2,89
hflK	SMD_3168	183	3,06	189	3,24
pilH	SMD_3170	224	2,65	249	2,62
cbpA	SMD_3171	166	2,35	206	2,77
bfr	SMD_3173	45	2,97	55	1,84
pbpC	SMD_3174	38	2,13	35	2,51
-	SMD_3175	71	2,80	53	3,15
prpD	SMD_3176	64	3,32	52	3,30
-	SMD_3177	97	2,36	91	3,00
-	SMD_3178	68	1,65	104	2,41
acnA	SMD_3180	164	2,13	188	2,26
prpC	SMD_3181	452	2,62	623	2,60
prpB	SMD_3182	71	2,47	99	1,88
-	SMD_3184	77	2,69	39	3,29
aroB	SMD_3189	57	2,81	42	3,23
hemE	SMD_3191	81	3,20	59	3,56
-	SMD_3192	47	2,08	24	4,13
-	SMD_3193	67	2,79	70	2,93
-	SMD_3194	17	2,99	21	3,54
-	SMD_3197	54	2,30	32	4,30
-	SMD_3200	27	2,93	24	3,82
prfC	SMD_3205	123	2,71	140	3,16
-	SMD_3208	114	2,67	118	2,84
gtrB	SMD_3209	124	3,37	107	3,31
hslO	SMD_3211	57	2,18	66	3,83
-	SMD_3212	37	4,21	38	5,44
-	SMD_3213	63	4,04	59	4,14
-	SMD_3214	60	1,95	53	2,56
-	SMD_3220	78	3,44	56	4,13
-	SMD_3223	361	2,60	453	2,91
gcvH	SMD_3224	444	2,44	750	2,32

gcvT	SMD_3225	161	2,47	212	2,70
-	SMD_3228	107	2,24	85	2,96
cysQ	SMD_3229	106	2,82	94	3,31
nudE	SMD_3230	105	2,32	80	3,10
bioA	SMD_3231	43	2,88	45	2,74
rsmE	SMD_3232	101	2,57	112	2,76
-	SMD_3235	156	2,26	130	2,84
-	SMD_3236	77	2,33	77	2,69
frzE	SMD_3237	108	3,63	101	4,13
gshB	SMD_3242	34	3,80	34	4,67
-	SMD_3243	119	3,01	85	3,48
-	SMD_3244	47	3,18	52	3,33
-	SMD_3247	182	2,37	132	3,22
mrcB	SMD_3248	71	2,89	69	3,12
-	SMD_3249	39	3,34	56	3,21
-	SMD_3250	79	2,74	67	2,99
relA	SMD_3251	66	3,03	68	2,96
-	SMD_3252	227	2,75	195	3,07
hrpA	SMD_3253	93	2,87	84	3,22
-	SMD_3254	386	3,59	709	3,98
recQ	SMD_3255	44	3,34	33	4,56
copB2	SMD_3258	16	3,44	21	4,22
-	SMD_3262	72	3,77	89	4,99
-	SMD_3264	50	4,46	90	4,29
-	SMD_3265	81	4,11	108	4,65
-	SMD_3266	42	7,15	104	4,97
-	SMD_3267	35	6,94	67	8,67
Q	SMD_3270	117	2,86	69	4,03
I	SMD_3284	17	3,37	16	5,46
D	SMD_3295	115	2,41	89	3,28
-	SMD_3303	35	2,92	35	2,88
-	SMD_3305	90	3,83	86	3,74
-	SMD_3306	109	2,61	135	3,41
-	SMD_3307	109	2,53	131	3,05
-	SMD_3308	172	3,02	192	3,09

-	SMD_3309	1265	2,14	1850	2,40
tolB	SMD_3310	272	2,36	484	2,53
tolA	SMD_3311	355	2,49	434	2,52
tolR	SMD_3312	166	2,46	239	2,83
tolQ	SMD_3313	138	2,91	187	2,77
kup	SMD_3316	101	2,47	113	2,65
ruvC	SMD_3318	161	2,68	176	2,39
-	SMD_3319	520	2,51	488	2,69
-	SMD_3320	122	3,15	127	2,58
-	SMD_3321	254	2,23	268	2,61
aspS	SMD_3323	162	2,89	202	3,15
-	SMD_3325	26	3,16	39	1,60
-	SMD_3326	23	3,79	31	3,58
ampR	SMD_3328	68	2,50	66	2,39
-	SMD_3329	99	3,13	72	4,01
-	SMD_3331	77	3,44	52	3,92
-	SMD_3332	28	3,08	22	3,51
-	SMD_3334	237	2,08	78	3,00
-	SMD_3335	311	2,68	160	3,13
clpB	SMD_3336	217	2,23	101	3,13
-	SMD_3338	36	3,01	30	3,92
-	SMD_3339	17	3,99	21	3,19
-	SMD_3341	31	2,74	38	3,04
-	SMD_3342	45	2,86	45	2,15
-	SMD_3343	21	3,26	32	3,42
otsA	SMD_3346	72	2,87	45	3,46
-	SMD_3349	63	2,71	104	2,73
rluD	SMD_3350	165	2,52	208	2,50
-	SMD_3352	68	3,23	61	3,00
nadE	SMD_3353	93	2,85	91	3,02
sucD	SMD_3354	2571	1,94	4023	1,94
sucC	SMD_3355	2170	1,95	3694	1,92
hydG	SMD_3357	74	3,13	66	3,73
pilB	SMD_3358	76	3,09	50	4,01
pilA	SMD_3359	159	2,53	108	3,04

pilC	SMD_3360	68	2,88	34	3,17
coaE	SMD_3362	117	2,77	72	4,14
-	SMD_3366	161	2,89	135	3,16
-	SMD_3367	77	2,82	60	3,46
-	SMD_3368	53	3,69	37	5,12
-	SMD_3369	147	2,72	71	3,69
-	SMD_3373	59	2,70	52	3,38
-	SMD_3374	49	3,49	47	3,35
-	SMD_3376	230	2,56	399	2,41
thiG	SMD_3378	142	2,61	141	3,32
trmB	SMD_3379	109	2,37	121	3,37
-	SMD_3380	48	2,89	34	3,92
-	SMD_3382	22	4,52	32	5,56
-	SMD_3383	71	3,16	85	3,36
mscL	SMD_3384	187	2,45	219	2,96
-	SMD_3385	47	2,96	62	3,18
-	SMD_3387	32	2,76	45	2,54
yfiF	SMD_3388	44	2,91	71	1,84
-	SMD_3389	55	2,59	44	3,29
smeN	SMD_3391	47	2,33	38	3,13
smeM	SMD_3392	22	3,16	23	2,66
-	SMD_3393	36	3,46	104	3,02
-	SMD_3399	468	2,46	518	2,51
alf1	SMD_3400	768	2,55	1302	2,44
pykA	SMD_3401	94	3,12	94	3,14
pgk	SMD_3403	91	2,33	162	2,92
-	SMD_3404	105	2,61	129	2,71
gap	SMD_3406	240	2,52	351	2,96
-	SMD_3407	354	3,75	1167	2,34
-	SMD_3408	82	3,35	103	3,30
-	SMD_3409	128	2,68	103	3,31
-	SMD_3413	16	6,38	27	6,42
-	SMD_3414	27	6,36	46	4,97
-	SMD_3415	84	3,69	48	5,75
tktA	SMD_3416	236	2,36	408	2,52

-	SMD_3417	118	2,24	209	1,97
-	SMD_3418	71	2,62	73	2,84
-	SMD_3419	74	2,52	63	2,46
batA	SMD_3420	66	2,56	69	2,80
-	SMD_3421	16	3,39	24	3,28
-	SMD_3423	56	3,77	53	4,16
pilQ	SMD_3424	95	2,82	67	3,82
pilP	SMD_3425	147	2,51	98	3,15
pilO	SMD_3426	93	2,57	75	3,80
mrcA	SMD_3429	48	3,08	38	3,81
-	SMD_3430	35	2,94	17	5,91
gltA	SMD_3438	745	2,18	1262	2,10
rpmE	SMD_3439	1235	2,68	1650	2,30
iunH	SMD_3440	150	2,74	168	2,90
recG	SMD_3441	119	2,51	151	2,57
-	SMD_3442	750	2,90	1287	2,69
rpoZ	SMD_3444	180	3,58	241	4,22
gmk	SMD_3445	44	3,37	35	3,25
fdnG	SMD_3446	49	5,60	60	5,59
selA	SMD_3450	22	4,09	22	3,53
selB	SMD_3451	22	2,85	23	4,04
selD	SMD_3453	53	4,54	42	5,20
rph	SMD_3455	70	2,52	83	3,10
-	SMD_3457	122	2,89	160	2,89
-	SMD_3458	86	2,59	68	3,44
-	SMD_3460	68	3,13	47	3,55
pepQ	SMD_3461	88	2,96	82	2,74
-	SMD_3463	135	2,57	177	2,39
-	SMD_3465	175	2,86	101	4,25
-	SMD_3468	62	3,30	34	3,98
rpiA	SMD_3469	98	2,14	329	2,22
-	SMD_3470	108	1,92	309	2,18
rubA	SMD_3472	127	2,96	117	3,17
thiE	SMD_3473	48	2,72	63	2,63
hemL	SMD_3474	117	2,90	161	2,92

-	SMD_3475	204	1,08	187	2,74
-	SMD_3476	101	2,73	50	3,44
astC	SMD_3477	120	2,84	109	3,13
-	SMD_3478	64	2,74	53	3,48
-	SMD_3479	97	3,27	117	3,08
-	SMD_3481	89	2,89	56	3,18
-	SMD_3482	282	2,51	298	2,78
-	SMD_3483	59	2,60	41	3,41
-	SMD_3485	53	2,50	54	3,18
-	SMD_3486	110	3,63	80	3,68
-	SMD_3487	169	2,25	108	3,34
mpl	SMD_3488	87	3,26	112	3,18
adk	SMD_3489	326	2,47	726	2,43
pfk	SMD_3490	42	3,77	60	3,17
-	SMD_3491	73	2,88	54	3,42
-	SMD_3492	67	3,71	31	3,80
-	SMD_3500	49	2,75	45	3,57
ppa	SMD_3505	773	2,49	1213	2,30
-	SMD_3506	44	2,51	47	3,41
-	SMD_3508	2020	3,00	3741	2,90
-	SMD_3509	123	2,66	107	3,27
cadC	SMD_3510	120	3,28	86	4,03
-	SMD_3511	19	3,45	19	3,55
-	SMD_3514	17	3,65	17	3,94
-	SMD_3516	32	2,62	39	2,52
leuA	SMD_3520	27	3,50	30	2,46
leuC	SMD_3522	38	2,59	31	2,89
leuD	SMD_3523	34	1,77	57	1,43
leuB	SMD_3524	71	2,21	70	2,40
-	SMD_3525	54	2,64	51	3,61
smeP	SMD_3527	43	2,85	46	3,14
pcm2	SMD_3530	490	2,59	606	3,15
tolC	SMD_3531	314	2,63	501	2,73
kdtA	SMD_3533	30	3,15	17	4,96
htrB	SMD_3534	66	3,69	62	3,72

-	SMD_3536	82	3,56	68	4,09
-	SMD_3539	75	2,44	83	2,69
-	SMD_3540	148	3,02	121	2,80
maeB	SMD_3541	379	2,55	535	2,70
oprP	SMD_3543	42	3,10	42	3,87
-	SMD_3545	56	2,73	32	4,39
tctD	SMD_3548	27	2,98	40	3,65
-	SMD_3549	17	3,24	46	1,81
fabG3	SMD_3551	25	1,63	99	2,31
engC	SMD_3554	37	3,55	33	4,49
-	SMD_3555	65	3,62	52	4,09
-	SMD_3556	64	2,85	66	4,61
-	SMD_3558	66	2,95	22	5,28
-	SMD_3559	181	2,80	179	3,40
-	SMD_3560	126	2,76	123	3,68
amn	SMD_3561	152	3,19	105	4,05
-	SMD_3562	32	3,16	23	3,11
-	SMD_3564	43	1,84	48	2,95
-	SMD_3567	108	2,90	152	2,79
-	SMD_3575	71	2,94	141	2,65
-	SMD_3576	35	3,41	145	2,46
adhC	SMD_3577	163	3,10	352	2,60
-	SMD_3580	106	3,10	48	4,01
-	SMD_3581	139	2,79	70	3,44
-	SMD_3582	39	2,80	36	3,39
-	SMD_3586	61	2,45	52	2,06
-	SMD_3587	55	2,89	27	4,05
-	SMD_3590	39	3,22	26	4,48
dsbA	SMD_3593	51	3,50	106	2,53
dsbA2	SMD_3594	202	2,39	359	2,41
-	SMD_3595	195	2,80	265	2,34
fhuE	SMD_3599	67	2,85	39	3,72
exbD5	SMD_3602	53	2,96	43	5,18
-	SMD_3603	35	3,59	27	4,00
-	SMD_3611	80	2,67	50	2,58

nagA	SMD_3612	68	2,15	93	2,20
-	SMD_3613	62	2,62	88	2,20
-	SMD_3614	39	2,46	22	3,20
-	SMD_3617	54	2,89	43	3,37
nahB	SMD_3618	38	3,45	33	3,42
-	SMD_3619	120	2,18	98	2,27
-	SMD_3620	50	3,28	41	3,83
-	SMD_3621	101	3,52	59	5,09
-	SMD_3622	53	3,64	59	4,06
-	SMD_3623	94	2,66	40	3,23
-	SMD_3624	299	3,08	275	3,01
-	SMD_3625	160	2,70	146	2,98
-	SMD_3626	197	2,87	236	3,03
-	SMD_3627	67	3,16	53	3,65
-	SMD_3630	138	2,62	210	2,74
-	SMD_3632	25	4,09	29	4,67
-	SMD_3633	155	3,07	139	3,39
lipA	SMD_3634	259	2,89	150	3,33
lipB	SMD_3635	163	3,20	162	3,91
-	SMD_3636	178	4,11	206	4,65
dacC	SMD_3638	275	2,39	493	2,30
rlpA	SMD_3639	102	3,33	68	4,57
mltB	SMD_3640	119	3,42	97	4,36
rodA	SMD_3641	55	2,77	16	3,54
mrdB	SMD_3642	182	2,46	210	2,45
mrdA	SMD_3643	117	2,14	81	2,61
mreC	SMD_3645	81	2,71	82	3,11
mreB	SMD_3646	97	3,21	82	3,40
-	SMD_3647	101	3,52	109	3,89
-	SMD_3648	207	2,64	200	3,10
-	SMD_3649	125	2,10	99	3,40
-	SMD_3650	203	2,45	282	2,69
ubiE	SMD_3651	146	3,67	155	3,38
codA	SMD_3652	61	3,19	111	2,83
-	SMD_3653	142	2,26	175	2,75

-	SMD_3654	239	2,39	277	2,83
-	SMD_3655	637	2,43	684	3,07
smeF	SMD_3656	319	2,39	662	2,48
smeE	SMD_3657	489	2,74	1171	2,51
smeD	SMD_3658	190	3,26	842	2,57
-	SMD_3660	84	2,83	44	3,59
hslU	SMD_3661	236	2,53	170	3,12
hslV	SMD_3662	135	2,68	128	2,96
xerC	SMD_3663	107	2,72	97	2,38
-	SMD_3664	259	2,55	290	2,55
dapF	SMD_3665	227	2,82	253	3,12
ptrB	SMD_3668	73	2,73	59	3,23
-	SMD_3669	111	3,26	58	4,11
-	SMD_3670	151	2,51	268	2,39
-	SMD_3671	40	4,29	21	6,24
-	SMD_3672	88	2,80	62	3,91
-	SMD_3674	44	3,45	59	4,77
-	SMD_3675	47	3,55	34	5,69
-	SMD_3676	180	2,55	280	2,28
-	SMD_3677	127	2,58	148	2,03
-	SMD_3678	125	2,71	92	2,57
prlC	SMD_3679	55	2,31	59	2,89
-	SMD_3681	70	2,84	56	3,42
-	SMD_3682	109	2,58	165	3,21
-	SMD_3684	133	2,47	112	3,54
glmS	SMD_3685	120	2,74	195	2,58
-	SMD_3691	34	2,95	22	4,50
-	SMD_3692	109	2,95	79	3,44
-	SMD_3693	132	2,99	102	2,80
glmU	SMD_3694	133	2,37	96	3,24
-	SMD_3695	210	2,36	141	2,42
-	SMD_3696	147	2,29	163	2,60
atpC	SMD_3697	1787	1,87	2763	2,18
atpD	SMD_3698	2635	2,09	5377	1,99
atpG	SMD_3699	1103	2,05	2385	2,03

atpA	SMD_3700	1482	2,29	3067	2,17
atpH	SMD_3701	526	2,52	976	2,51
atpF	SMD_3702	1314	2,62	2551	2,45
atpE	SMD_3703	1240	2,63	3759	2,52
atpB	SMD_3704	297	3,60	329	4,01
-	SMD_3705	190	4,66	172	6,13
-	SMD_3707	19	3,63	17	5,88
lpdA	SMD_3708	416	2,40	550	2,54
DLAT	SMD_3709	341	2,81	433	2,64
-	SMD_3711	1145	3,74	2082	3,79
-	SMD_3714	154	2,68	165	2,95
-	SMD_3715	50	2,84	24	4,79
-	SMD_3716	26	4,61	18	4,49
-	SMD_3717	142	3,48	220	2,69
hemC	SMD_3718	25	4,76	31	4,89
-	SMD_3719	48	2,78	41	2,87
estB	SMD_3721	46	3,76	62	3,72
-	SMD_3722	43	3,59	32	4,27
cirA	SMD_3723	36	3,06	19	6,26
-	SMD_3725	20	4,53	21	5,65
-	SMD_3733	33	3,95	41	2,77
-	SMD_3735	67	2,59	113	2,55
gst5	SMD_3746	298	2,97	210	3,74
-	SMD_3747	88	2,46	57	3,25
-	SMD_3748	46	1,41	22	3,41
hnsB	SMD_3749	21	1,75	26	1,72
-	SMD_3755	112	2,23	108	2,73
-	SMD_3757	24	6,31	23	16,66
-	SMD_3758	30	3,35	18	3,56
-	SMD_3760	84	2,89	47	3,64
-	SMD_3761	37	1,31	20	2,98
-	SMD_3762	74	3,77	30	6,04
rpoD	SMD_3765	311	2,69	314	2,92
ribA	SMD_3768	44	3,01	67	3,37
-	SMD_3769	40	2,49	29	3,87

waaE	SMD_3772	98	3,19	85	3,81
-	SMD_3773	155	3,20	136	3,43
-	SMD_3774	106	3,04	94	3,45
rsmB	SMD_3775	114	2,88	143	2,96
def	SMD_3777	63	3,56	72	2,68
-	SMD_3778	22	5,58	24	5,33
smf	SMD_3779	25	2,99	20	3,63
smg	SMD_3780	52	4,84	73	3,16
-	SMD_3781	41	4,28	58	4,04
-	SMD_3782	86	2,82	60	3,57
topA	SMD_3783	120	2,47	96	3,06
-	SMD_3784	63	3,09	38	4,30
panE	SMD_3787	38	3,10	72	3,29
sppA	SMD_3789	149	3,09	146	3,24
-	SMD_3792	64	4,14	78	4,03
priA	SMD_3793	30	3,59	26	3,96
-	SMD_3802	124	4,13	84	6,29
aroG	SMD_3803	94	4,69	112	5,29
-	SMD_3804	20	1,77	17	4,64
-	SMD_3811	45	3,68	24	5,11
-	SMD_3812	55	3,51	38	4,36
groEL	SMD_3813	9271	3,46	8369	3,77
groES	SMD_3814	4349	3,64	4498	3,82
-	SMD_3817	30	2,61	39	3,84
-	SMD_3818	35	2,71	48	2,72
-	SMD_3824	34	3,05	22	4,28
-	SMD_3826	23	6,44	32	5,96
-	SMD_3827	35	5,12	46	3,49
-	SMD_3830	16	4,11	23	3,87
aroQ	SMD_3832	119	2,54	61	3,89
accB	SMD_3834	504	2,20	1013	2,14
accC	SMD_3835	886	1,94	1507	2,02
-	SMD_3836	145	2,17	176	3,29
-	SMD_3837	59	2,87	52	3,32
prmA	SMD_3838	56	3,20	45	4,03

-	SMD_3839	44	3,96	49	3,43
cdsA2	SMD_3841	98	2,28	38	4,47
-	SMD_3842	68	2,72	40	3,07
purH	SMD_3847	77	2,86	90	3,22
rpoH	SMD_3850	46	4,52	55	4,41
ung	SMD_3851	180	2,45	223	2,84
-	SMD_3852	128	2,71	126	2,50
ftsE	SMD_3854	121	3,34	133	3,25
rhlB	SMD_3855	125	3,22	155	2,84
-	SMD_3856	62	3,95	40	4,04
trxA	SMD_3857	306	1,93	470	2,62
-	SMD_3858	809	2,62	635	2,98
rho	SMD_3859	425	2,51	423	2,64
-	SMD_3860	32	4,00	26	5,90
icd	SMD_3868	242	2,89	293	2,75
-	SMD_3869	29	3,30	34	4,05
-	SMD_3871	48	3,71	53	3,42
-	SMD_3872	36	3,05	26	3,97
smeI	SMD_3873	22	3,84	17	4,04
smeK	SMD_3875	33	3,10	25	3,69
-	SMD_3876	16	3,55	18	2,95
aas	SMD_3878	36	3,48	36	3,95
-	SMD_3879	574	3,03	642	3,41
-	SMD_3880	607	2,74	1703	2,42
-	SMD_3881	102	2,59	174	3,54
-	SMD_3882	103	3,70	65	4,50
nudC	SMD_3883	84	2,76	60	4,34
-	SMD_3884	90	4,13	56	4,27
-	SMD_3885	142	2,54	153	2,78
-	SMD_3888	57	3,92	60	3,11
-	SMD_3889	70	3,41	74	4,12
bfr2	SMD_3890	168	3,36	157	3,13
-	SMD_3891	78	1,38	36	4,98
nudH	SMD_3892	65	3,11	66	3,43
rpsI	SMD_3895	896	2,40	1404	2,62

rplM	SMD_3896	3778	2,33	5388	2,49
-	SMD_3897	104	3,32	102	3,20
sugE2	SMD_3898	180	3,24	172	2,99
speD	SMD_3899	227	2,79	289	2,61
crp	SMD_3900	244	3,11	229	3,44
-	SMD_3901	203	3,04	192	3,62
-	SMD_3902	114	3,38	110	3,43
trpC	SMD_3903	109	3,25	127	3,39
trpD	SMD_3904	121	2,95	131	2,66
pabA	SMD_3905	99	2,98	127	3,45
trpE	SMD_3907	41	4,46	31	6,54
-	SMD_3908	38	3,22	48	3,91
rpe	SMD_3910	57	2,46	196	1,91
purC	SMD_3912	22	4,62	22	4,58
-	SMD_3913	26	2,67	16	4,95
phaAB	SMD_3914	35	2,76	30	3,80
phaD	SMD_3916	96	3,08	88	2,51
phaE	SMD_3917	131	2,50	73	2,64
phaF	SMD_3918	64	1,41	120	3,22
yrbG	SMD_3920	130	3,06	93	3,30
-	SMD_3922	210	2,12	110	2,35
hmgA	SMD_3923	131	2,13	107	2,56
-	SMD_3924	103	2,72	93	3,27
-	SMD_3926	31	4,40	42	4,34
-	SMD_3927	238	2,49	334	1,97
-	SMD_3928	296	2,20	403	2,59
-	SMD_3929	55	2,81	22	4,76
pdhA	SMD_3930	238	2,31	326	2,50
pdhB	SMD_3931	414	2,03	572	2,47
-	SMD_3932	641	2,07	808	2,18
-	SMD_3933	460	2,14	808	1,93
-	SMD_3938	77	3,05	65	3,19
-	SMD_3942	101	1,31	46	3,78
-	SMD_3946	44	5,63	33	6,89
-	SMD_3949	56	2,37	41	3,42

speA	SMD_3951	142	2,75	156	2,98
speE	SMD_3952	355	2,67	551	2,50
lpxO	SMD_3953	135	3,33	126	3,48
-	SMD_3954	19	3,14	19	2,94
-	SMD_3956	116	3,42	83	4,49
glnB3	SMD_3957	156	3,42	272	2,65
-	SMD_3959	468	3,10	769	3,28
ubiA	SMD_3995	18	2,79	23	4,56
-	SMD_3997	22	3,65	22	4,28
agaS	SMD_4001	22	3,55	16	1,76
agaR	SMD_4004	33	2,48	32	3,70
nagA2	SMD_4005	34	2,27	27	3,97
-	SMD_4006	27	3,96	26	3,50
-	SMD_4007	60	1,48	42	3,14
bioF	SMD_4013	50	3,06	34	3,24
-	SMD_4016	71	2,87	61	4,21
-	SMD_4017	90	3,14	62	3,37
-	SMD_4018	56	4,06	61	3,88
-	SMD_4019	52	3,87	72	3,72
gidA	SMD_4020	55	3,74	62	3,65
smeC	SMD_4021	25	4,00	22	5,05
-	SMD_4027	49	2,71	37	3,84
-	SMD_4028	57	3,38	42	3,19
-	SMD_4029	48	2,81	45	3,52
-	SMD_4030	87	2,59	84	2,77
-	SMD_4032	98	2,44	125	1,89
-	SMD_4033	82	2,55	57	3,28
-	SMD_4034	182	2,87	179	2,82
-	SMD_4035	173	2,89	179	2,78
-	SMD_4036	126	3,14	97	3,37
ilvD	SMD_4038	33	3,36	39	2,63
aroE	SMD_4041	53	3,72	85	2,95
-	SMD_4042	72	2,99	97	3,51
hemB	SMD_4044	111	2,72	344	2,20
-	SMD_4045	36	3,75	60	3,21

-	SMD_4046	121	2,81	160	3,08
-	SMD_4047	121	3,19	116	4,19
-	SMD_4048	55	3,12	64	3,95
-	SMD_4049	135	2,53	112	3,03
-	SMD_4050	108	2,92	108	2,80
gst6	SMD_4051	36	3,97	16	4,04
-	SMD_4052	182	2,88	197	2,69
-	SMD_4053	66	4,02	66	3,91
-	SMD_4056	38	2,40	55	2,44
-	SMD_4057	35	2,66	36	2,76
-	SMD_4058	40	1,77	37	3,42
-	SMD_4063	72	2,93	57	3,34
-	SMD_4064	54	3,64	45	3,45
-	SMD_4065	89	2,59	76	4,12
-	SMD_4066	41	3,44	29	4,86
-	SMD_4068	77	3,72	91	3,73
tatD	SMD_4069	78	2,85	68	4,00
hrpB	SMD_4070	31	3,65	42	3,34
-	SMD_4071	142	3,35	206	3,17
-	SMD_4073	45	3,03	30	4,90
-	SMD_4075	98	10,69	92	10,37
rtcB	SMD_4076	66	7,95	60	8,16
-	SMD_4077	96	7,60	93	8,45
-	SMD_4078	59	7,78	53	7,72
-	SMD_4083	43	3,31	38	3,95
-	SMD_4085	66	3,70	38	6,04
kefB	SMD_4086	73	2,81	71	3,79
-	SMD_4087	83	3,10	78	3,38
ycgL	SMD_4088	66	4,60	26	5,78
-	SMD_4089	162	2,25	313	1,91
fabF2	SMD_4090	140	2,11	156	2,20
fabG5	SMD_4091	142	2,15	190	2,25
-	SMD_4092	97	1,31	120	2,33
-	SMD_4093	168	2,34	234	2,29
-	SMD_4094	63	3,25	68	2,14

-	SMD_4095	106	2,52	167	2,11
-	SMD_4096	97	2,45	142	2,43
-	SMD_4097	143	2,18	126	2,71
-	SMD_4098	190	1,27	188	2,45
-	SMD_4099	94	2,15	123	2,08
-	SMD_4101	115	2,76	140	2,99
-	SMD_4102	156	2,94	252	2,82
-	SMD_4103	186	2,76	222	2,81
acpP2	SMD_4104	138	2,56	219	2,01
-	SMD_4106	61	3,04	54	4,00
-	SMD_4107	36	3,49	36	4,34
-	SMD_4114	73	2,91	96	2,80
-	SMD_4116	23	3,48	17	3,02
-	SMD_4118	28	3,86	53	3,29
hemF	SMD_4119	33	3,50	31	3,56
-	SMD_4120	49	3,32	40	4,03
polA	SMD_4122	79	2,83	86	3,28
-	SMD_4125	107	2,34	156	2,99
uvrD	SMD_4127	88	3,11	111	3,51
-	SMD_4128	53	3,45	33	4,70
rpmG	SMD_4131	319	3,79	446	3,96
rpmB	SMD_4132	2171	3,31	3322	3,77
-	SMD_4133	33	4,38	55	4,63
smmP	SMD_4139	28	3,30	24	4,03
smmQ	SMD_4140	72	2,86	89	2,52
-	SMD_4141	107	3,34	86	3,56
DPM1	SMD_4143	107	3,56	52	7,41
-	SMD_4144	35	3,65	25	3,95
-	SMD_4145	232	2,67	239	2,50
parB	SMD_4146	159	2,63	98	3,43
parA	SMD_4147	95	3,18	93	3,17
gidB	SMD_4148	23	4,21	21	4,76
-	SMD_4150	277	3,29	203	3,09
xthA3	SMD_4151	78	3,26	62	3,38
acsA	SMD_4155	119	3,48	140	3,63

-	SMD_4156	108	3,99	85	4,92
-	SMD_4157	72	3,31	48	3,30
-	SMD_4158	46	2,59	50	2,83
-	SMD_4159	217	2,56	335	2,45
hscC	SMD_4160	41	3,20	39	3,76
-	SMD_4161	450	2,31	298	3,26
-	SMD_4162	111	2,39	93	2,83
-	SMD_4164	153	2,20	140	2,45
-	SMD_4165	87	2,62	91	3,40
glyS	SMD_4166	177	2,54	190	2,92
glyQ	SMD_4167	88	3,14	111	3,56
-	SMD_4169	48	3,62	44	4,03
tatC	SMD_4171	104	3,39	78	4,15
tatB	SMD_4172	102	3,95	108	3,71
tatE	SMD_4173	218	3,46	91	5,24
-	SMD_4174	69	5,37	90	4,33
-	SMD_4177	44	3,21	46	3,84
-	SMD_4190	76	2,46	60	3,10
-	SMD_4191	136	2,86	111	2,91
-	SMD_4192	146	2,64	141	3,11
-	SMD_4193	16	4,03	19	4,30
-	SMD_4198	303	2,20	198	2,72
-	SMD_4199	215	2,23	287	2,23
-	SMD_4200	149	2,65	269	2,45
-	SMD_4201	110	2,45	133	2,68
folE	SMD_4202	191	2,94	273	3,02
-	SMD_4206	24	3,40	25	4,72
-	SMD_4208	66	3,47	36	2,50
recD	SMD_4209	52	2,84	74	2,80
recB	SMD_4210	96	2,31	108	2,39
recC	SMD_4211	44	2,76	36	3,57
-	SMD_4212	57	3,80	47	4,33
-	SMD_4213	38	2,57	28	3,95
-	SMD_4214	122	2,43	92	4,10
-	SMD_4215	187	2,63	250	2,84

-	SMD_4216	249	2,59	331	2,69
-	SMD_4217	169	2,68	249	2,45
btuE	SMD_4218	306	2,32	381	2,18
rmuC	SMD_4220	69	2,71	46	2,86
-	SMD_4222	21	2,75	24	3,53
-	SMD_4225	43	4,20	49	3,42
-	SMD_4228	69	2,57	80	3,53
-	SMD_4229	100	2,79	92	3,94
rlmI	SMD_4230	224	2,97	255	3,31
-	SMD_4233	40	3,82	29	6,66
glpQ	SMD_4234	57	4,88	51	6,81
trmE	SMD_4235	54	3,03	78	3,75
-	SMD_4236	33	3,17	48	3,39
yidC	SMD_4237	259	2,76	282	3,11
rpmH	SMD_4239	236	3,84	298	3,52

Genes grouped in operons are framed with a thick line

Table S3. Overview of *S. maltophilia* mRNA half-lives as a funtion of their functional category

Category	Subcategory	Median Half life in D457	No of genes in subcategory
Respiration	Electron donating reactions	2,02	19
Cell Wall and Capsule	Capsular and extracellular polysaccharides	2,27	11
Carbohydrates	Monosaccharides	2,28	15
Fatty Acids, Lipids, and Isoprenoids	Fatty acids	2,34	20
Fatty Acids, Lipids, and Isoprenoids	Isoprenoids	2,43	13
Carbohydrates	One-carbon Metabolism	2,50	22
Virulence, Disease and Defense	Resistance to antibiotics and toxic compounds	2,52	8
RNA Metabolism	RNA processing and modification	2,52	31
Carbohydrates	Central carbohydrate metabolism	2,52	55
Carbohydrates	Aminosugars	2,52	8
Carbohydrates	Organic acids	2,55	12
Amino Acids and Derivatives	Branched-chain amino acids	2,56	29
Protein Metabolism	Protein processing and modification	2,59	11
Cell Wall and Capsule	Gram-Negative cell wall components	2,59	11
Fatty Acids, Lipids, and Isoprenoids	Fatty Acids, Lipids, and Isoprenoids - no subcategory	2,61	8
Carbohydrates	Fermentation	2,64	9
Cofactors, Vitamins, Prosthetic Groups, Pigments	NAD and NADP	2,70	12
Protein Metabolism	Protein biosynthesis	2,72	30
Regulation and Cell signaling	Regulation and Cell signaling - no subcategory	2,72	11
Amino Acids and Derivatives	Alanine, serine, and glycine	2,74	16
Cell Wall and Capsule	Cell Wall and Capsule - no subcategory	2,74	29
Amino Acids and Derivatives	Lysine, threonine, methionine, and cysteine	2,80	31
Fatty Acids, Lipids, and Isoprenoids	Phospholipids	2,81	16
Nucleosides and Nucleotides	Purines	2,81	26
Protein Metabolism	Protein degradation	2,81	14
Amino Acids and Derivatives	Aromatic amino acids and derivatives	2,82	21
Cofactors, Vitamins, Prosthetic Groups, Pigments	Folate and pterines	2,84	34

Nucleosides and Nucleotides	Detoxification	2,89	9
Nucleosides and Nucleotides	Pyrimidines	2,89	19
DNA Metabolism	DNA repair	2,91	16
Stress Response	Oxidative stress	2,98	25
Cofactors, Vitamins, Prosthetic Groups, Pigments	Riboflavin, FMN, FAD	3,14	8

Table S4. Functional categories of genes analyzed in the article

Name	Gene	Half time D457	Half time ALB001	EC number	Category	Subcategory
gyrB	SMD_0004	2,60	2,66	EC_5.99.1.3	DNA Metabolism	DNA replication
gyrB	SMD_0004	2,60	2,66	EC_5.99.1.3	Virulence, Disease and Defense	Resistance to antibiotics and toxic compounds
aspC	SMD_0018	2,92	3,16	EC_2.6.1.57	Cofactors, Vitamins, Prosthetic Groups, Pigments	Folate and pterines
aspC	SMD_0018	2,92	3,16	EC_2.6.1.57	Metabolism of Aromatic Compounds	Metabolism of central aromatic intermediates
-	SMD_0025	3,27	2,51	EC_1.1.1.1	Carbohydrates	Fermentation
-	SMD_0025	3,27	2,51	EC_1.1.1.1	Cofactors, Vitamins, Prosthetic Groups, Pigments	Folate and pterines
-	SMD_0025	3,27	2,51	EC_1.1.1.1	Fatty Acids, Lipids, and Isoprenoids	Phospholipids
phhA	SMD_0029	3,32	4,69	EC_1.14.16.1	Amino Acids and Derivatives	Aromatic amino acids and derivatives
phhA	SMD_0029	3,32	4,69	EC_1.14.16.1	Cofactors, Vitamins, Prosthetic Groups, Pigments	Folate and pterines
fpg	SMD_0041	3,85	3,11	EC_3.2.2.23	DNA Metabolism	DNA repair
tdk	SMD_0043	3,93	4,60	EC_2.7.1.21	Nucleosides and Nucleotides	Pyrimidines
pyrF	SMD_0054	3,32	2,86	EC_4.1.1.23	Nucleosides and Nucleotides	Pyrimidines
plsB	SMD_0057	3,06	4,14	EC_2.3.1.15	Fatty Acids, Lipids, and Isoprenoids	Phospholipids
-	SMD_0061	3,38	4,01	EC_4.2.3.3	Carbohydrates	Central carbohydrate metabolism
gltB	SMD_0064	3,18	3,01	EC_1.4.1.13	Nitrogen Metabolism	Nitrogen Metabolism
plsC	SMD_0096	4,11	3,81	EC_2.3.1.51	Fatty Acids, Lipids, and Isoprenoids	Phospholipids
glnA	SMD_0103	3,71	3,97	EC_6.3.1.2	Cell Wall and Capsule	Cell Wall and Capsule
glnA	SMD_0103	3,71	3,97	EC_6.3.1.2	Nitrogen Metabolism	Nitrogen Metabolism
ampDII	SMD_0107	3,61	3,36	EC_3.5.1.28	Cell Wall and Capsule	Cell Wall and Capsule
ampDII	SMD_0107	3,61	3,36	EC_3.5.1.28	Regulation and Cell signaling	Regulation and Cell signaling
mltA	SMD_0108	4,01	5,58	EC_3.2.1.-	Cell Wall and Capsule	Cell Wall and Capsule
sodC	SMD_0114	2,79	2,82	EC_1.15.1.1	Stress Response	Oxidative stress
sodC2	SMD_0115	2,64	2,63	EC_1.15.1.1	Stress Response	Oxidative stress
-	SMD_0118	4,30	3,61	EC_2.3.1.16	Amino Acids and Derivatives	Branched-chain amino acids
-	SMD_0118	4,30	3,61	EC_2.3.1.16, EC_2.3.1.9	Carbohydrates	One-carbon Metabolism
-	SMD_0118	4,30	3,61	EC_2.3.1.9	Carbohydrates	Fermentation
-	SMD_0118	4,30	3,61	EC_2.3.1.16	Cofactors, Vitamins, Prosthetic Groups, Pigments	Biotin
-	SMD_0118	4,30	3,61	EC_2.3.1.16, EC_2.3.1.9	Fatty Acids, Lipids, and Isoprenoids	Fatty Acids, Lipids, and Isoprenoids
-	SMD_0118	4,30	3,61	EC_2.3.1.9	Fatty Acids, Lipids, and Isoprenoids	Isoprenoids
gpsA	SMD_0126	2,41	2,40	EC_1.1.1.94	Carbohydrates	Sugar alcohols
gpsA	SMD_0126	2,41	2,40	EC_1.1.1.94	Fatty Acids, Lipids, and Isoprenoids	Phospholipids
-	SMD_0156	3,09	4,13	EC_2.6.1.1	Amino Acids and Derivatives	Lysine, threonine, methionine, and cysteine
-	SMD_0169	2,64	3,40	EC_1.5.5.1	Carbohydrates	Fermentation
-	SMD_0169	2,64	3,40	EC_1.5.5.1	Respiration	Electron accepting reactions
truC	SMD_0188	3,99	3,75	EC_4.2.1.70	RNA Metabolism	RNA processing and modification

dcp	SMD_0192	3,06	3,12	EC_3.4.15.5	Protein Metabolism	Protein degradation
lepB2	SMD_0195	4,59	3,71	EC_3.4.21.89	Protein Metabolism	Protein processing and modification
-	SMD_0202	3,08	3,16	EC_4.6.1.1	Regulation and Cell signaling	Regulation and Cell signaling
-	SMD_0205	2,17	2,34	EC_6.4.1.4	Amino Acids and Derivatives	Branched-chain amino acids
-	SMD_0205	2,17	2,34	EC_6.4.1.4	Carbohydrates	One-carbon Metabolism
-	SMD_0206	2,19	2,05	EC_6.4.1.4	Amino Acids and Derivatives	Branched-chain amino acids
-	SMD_0206	2,19	2,05	EC_6.4.1.4	Carbohydrates	One-carbon Metabolism
ivd	SMD_0207	2,27	2,95	EC_1.3.8.4	Amino Acids and Derivatives	Branched-chain amino acids
RRM1	SMD_0215	2,87	3,09	EC_1.17.4.1	Nucleosides and Nucleotides	Nucleosides and Nucleotides
RRM2	SMD_0216	2,17	2,50	EC_1.17.4.1	Nucleosides and Nucleotides	Nucleosides and Nucleotides
purU	SMD_0222	3,19	4,03	EC_3.5.1.10	Carbohydrates	One-carbon Metabolism
purU	SMD_0222	3,19	4,03	EC_3.5.1.10	Cofactors, Vitamins, Prosthetic Groups, Pigments	Folate and pterines
pncA	SMD_0223	3,01	3,58	EC_3.5.1.19	Cofactors, Vitamins, Prosthetic Groups, Pigments	NAD and NADP
pncA	SMD_0223	3,01	3,58	EC_3.5.1.19	Stress Response	Oxidative stress
-	SMD_0225	4,54	5,55	EC_2.4.2.12	Cofactors, Vitamins, Prosthetic Groups, Pigments	NAD and NADP
-	SMD_0226	4,68	5,89	EC_2.7.7.1, EC_3.6.1.13	Cofactors, Vitamins, Prosthetic Groups, Pigments	NAD and NADP
-	SMD_0230	2,58	2,56	EC_1.3.99.12	Amino Acids and Derivatives	Branched-chain amino acids
-	SMD_0232	2,56	2,11	EC_3.1.2.4	Amino Acids and Derivatives	Branched-chain amino acids
mmsB	SMD_0233	2,94	2,77	EC_1.1.1.31	Amino Acids and Derivatives	Branched-chain amino acids
birA	SMD_0265	4,21	3,87	EC_6.3.4.15	Cofactors, Vitamins, Prosthetic Groups, Pigments	Biotin
trpS	SMD_0280	3,12	3,32	EC_6.1.1.2	Protein Metabolism	Protein biosynthesis
katA	SMD_0296	3,48	4,83	EC_1.11.1.6	Stress Response	Oxidative stress
-	SMD_0297	3,26	2,88	EC_3.4.11.23	Protein Metabolism	Protein degradation
argS	SMD_0318	3,27	3,23	EC_6.1.1.19	Protein Metabolism	Protein biosynthesis
dfp	SMD_0321	3,37	3,41	EC_4.1.1.36, EC_6.3.2.5	Cofactors, Vitamins, Prosthetic Groups, Pigments	Coenzyme A
dut	SMD_0322	1,41	3,27	EC_3.6.1.23	Nucleosides and Nucleotides	Detoxification
algC	SMD_0323	2,59	2,61	EC_5.4.2.8	Carbohydrates	Monosaccharides
pyrE	SMD_0330	2,35	2,49	EC_2.4.2.10	Nucleosides and Nucleotides	Pyrimidines
ampN	SMD_0331	2,80	3,30	EC_3.1.11.2	DNA Metabolism	DNA repair
-	SMD_0334	2,63	3,76	EC_2.7.1.-	Cell Wall and Capsule	Cell Wall and Capsule
tyrS	SMD_0336	2,70	3,10	EC_6.1.1.1	Protein Metabolism	Protein biosynthesis
ctpA	SMD_0351	3,33	4,47	EC_3.4.21.102	Miscellaneous	Miscellaneous
nadR	SMD_0355	2,34	2,39	EC_2.7.1.22, EC_2.7.7.1	Cofactors, Vitamins, Prosthetic Groups, Pigments	NAD and NADP
dnaG	SMD_0371	2,76	3,67	EC_2.7.7.-	Cell Division and Cell Cycle	Cell Division and Cell Cycle
folB	SMD_0377	2,57	3,70	EC_4.1.2.25	Cofactors, Vitamins, Prosthetic Groups, Pigments	Folate and pterines
glnE	SMD_0399	2,80	4,63	EC_2.7.7.42	Nitrogen Metabolism	Nitrogen Metabolism
aceE2	SMD_0412	2,30	2,40	EC_1.2.4.1	Amino Acids and Derivatives	Lysine, threonine, methionine, and cysteine

aceE2	SMD_0412	2,30	2,40	EC_1.2.4.1	Carbohydrates	Central carbohydrate metabolism
aceE2	SMD_0412	2,30	2,40	EC_1.2.4.1	Cofactors, Vitamins, Prosthetic Groups, Pigments	Folate and pterines
msrB	SMD_0479	3,43	3,03	EC_1.8.4.12	Protein Metabolism	Protein processing and modification
dadA	SMD_0483	3,52	3,87	EC_1.4.99.1	Carbohydrates	Central carbohydrate metabolism
dadA	SMD_0483	3,52	3,87	EC_1.4.99.1	Respiration	Electron donating reactions
-	SMD_0484	2,96	2,99	EC_5.1.1.1	Amino Acids and Derivatives	Alanine, serine, and glycine
-	SMD_0484	2,96	2,99	EC_5.1.1.1	Carbohydrates	Central carbohydrate metabolism
rsuA	SMD_0490	2,79	2,98	EC_4.2.1.70	RNA Metabolism	RNA processing and modification
fabB	SMD_0492	2,33	2,32	EC_2.3.1.41	Fatty Acids, Lipids, and Isoprenoids	Fatty acids
fabA	SMD_0493	2,19	2,23	EC_4.2.1.59	Fatty Acids, Lipids, and Isoprenoids	Fatty acids
-	SMD_0498	3,24	4,19	EC_2.1.1.-	Membrane Transport	Protein and nucleoprotein secretion system, Type IV
gph	SMD_0499	3,64	3,71	EC_3.1.3.18	Carbohydrates	Central carbohydrate metabolism
gph	SMD_0499	3,64	3,71	EC_3.1.3.18	DNA Metabolism	DNA repair
-	SMD_0501	2,86	3,67	EC_3.1.3.73	Miscellaneous	Miscellaneous
-	SMD_0520	2,91	3,46	EC_5.2.1.2	Cofactors, Vitamins, Prosthetic Groups, Pigments	Folate and pterines
-	SMD_0520	2,91	3,46	EC_5.2.1.2	Metabolism of Aromatic Compounds	Metabolism of central aromatic intermediates
-	SMD_0520	2,91	3,46	EC_5.2.1.2	Metabolism of Aromatic Compounds	Metabolism of Aromatic Compounds
-	SMD_0520	2,91	3,46	EC_2.5.1.18	Stress Response	Oxidative stress
cbsB	SMD_0527	2,67	2,57	EC_4.2.1.22	Amino Acids and Derivatives	Lysine, threonine, methionine, and cysteine
cbsB	SMD_0527	2,67	2,57	EC_4.2.1.22	Amino Acids and Derivatives	Alanine, serine, and glycine
metC	SMD_0528	2,64	2,47	EC_4.4.1.1	Amino Acids and Derivatives	Lysine, threonine, methionine, and cysteine
metC	SMD_0528	2,64	2,47	EC_4.4.1.1	Amino Acids and Derivatives	Alanine, serine, and glycine
-	SMD_0533	1,96	1,84	EC_5.1.3.2	Cell Wall and Capsule	Capsular and extracellular polysacchrides
-	SMD_0533	1,96	1,84	EC_5.1.3.2	Protein Metabolism	Protein processing and modification
rfbB	SMD_0544	2,35	2,32	EC_4.2.1.46	Cell Wall and Capsule	Capsular and extracellular polysacchrides
rmlA	SMD_0545	2,19	2,32	EC_2.7.7.24	Cell Wall and Capsule	Capsular and extracellular polysacchrides
rmlC	SMD_0546	1,97	2,23	EC_5.1.3.13	Cell Wall and Capsule	Capsular and extracellular polysacchrides
rfbD	SMD_0547	2,05	2,31	EC_1.1.1.133	Cell Wall and Capsule	Capsular and extracellular polysacchrides
manA	SMD_0548	2,12	2,30	EC_2.7.7.22	Carbohydrates	Monosaccharides
spgM	SMD_0549	2,47	2,51	EC_5.4.2.8	Carbohydrates	Monosaccharides
lpsI	SMD_0550	2,74	3,22	EC_2.8.3.5	Amino Acids and Derivatives	Branched-chain amino acids
lpsI	SMD_0550	2,74	3,22	EC_2.8.3.5	Carbohydrates	One-carbon Metabolism
lpsI	SMD_0550	2,74	3,22	EC_2.8.3.5	Metabolism of Aromatic Compounds	Metabolism of central aromatic intermediates
lpsJ	SMD_0551	2,20	2,52	EC_2.8.3.5	Amino Acids and Derivatives	Branched-chain amino acids
lpsJ	SMD_0551	2,20	2,52	EC_2.8.3.5	Carbohydrates	One-carbon Metabolism
lpsJ	SMD_0551	2,20	2,52	EC_2.8.3.5	Metabolism of Aromatic Compounds	Metabolism of central aromatic intermediates
proS	SMD_0559	3,00	3,15	EC_6.1.1.15	Protein Metabolism	Protein biosynthesis

pssA	SMD_0562	4,56	3,03	EC_2.7.8.8	Fatty Acids, Lipids, and Isoprenoids	Phospholipids
valS	SMD_0566	2,33	2,49	EC_6.1.1.9	Protein Metabolism	Protein biosynthesis
pepA	SMD_0569	2,92	2,72	EC_3.4.11.1	Carbohydrates	Central carbohydrate metabolism
pepA	SMD_0569	2,92	2,72	EC_3.4.11.1	Protein Metabolism	Protein degradation
purL	SMD_0576	2,55	2,59	EC_6.3.5.3	Nucleosides and Nucleotides	Purines
wecB	SMD_0599	3,01	3,12	EC_5.1.3.14	Cell Wall and Capsule	Capsular and extracellular polysacchrides
glyA	SMD_0607	3,32	3,13	EC_2.1.2.1	Amino Acids and Derivatives	Alanine, serine, and glycine
glyA	SMD_0607	3,32	3,13	EC_2.1.2.1	Carbohydrates	One-carbon Metabolism
glyA	SMD_0607	3,32	3,13	EC_2.1.2.1	Cofactors, Vitamins, Prosthetic Groups, Pigments	Folate and pterines
ribD	SMD_0611	3,08	4,61	EC_1.1.1.193, EC_3.5.4.26	Cofactors, Vitamins, Prosthetic Groups, Pigments	Riboflavin, FMN, FAD
ribE	SMD_0615	3,56	4,17	EC_2.5.1.9	Cofactors, Vitamins, Prosthetic Groups, Pigments	Riboflavin, FMN, FAD
ribB	SMD_0616	3,20	3,49	EC_3.5.4.25	Cofactors, Vitamins, Prosthetic Groups, Pigments	Riboflavin, FMN, FAD
ribB	SMD_0616	3,20	3,49	EC_3.5.4.25	Cofactors, Vitamins, Prosthetic Groups, Pigments	Folate and pterines
ribH	SMD_0617	3,30	3,04	EC_2.5.1.9	Cofactors, Vitamins, Prosthetic Groups, Pigments	Riboflavin, FMN, FAD
thiL	SMD_0619	3,48	2,10	EC_2.7.4.16	Cofactors, Vitamins, Prosthetic Groups, Pigments	Cofactors, Vitamins, Prosthetic Groups, Pigments
thiL	SMD_0619	3,48	2,10	EC_2.7.4.16	Cofactors, Vitamins, Prosthetic Groups, Pigments	Folate and pterines
ftsI	SMD_0638	2,81	2,93	EC_2.4.1.129	Cell Division and Cell Cycle	Cell Division and Cell Cycle
ftsI	SMD_0638	2,81	2,93	EC_2.4.1.129	Cell Wall and Capsule	Cell Wall and Capsule
ftsI	SMD_0638	2,81	2,93	EC_2.4.1.129	RNA Metabolism	RNA processing and modification
murE	SMD_0639	2,33	2,40	EC_6.3.2.13	Cell Wall and Capsule	Cell Wall and Capsule
murF	SMD_0640	2,18	2,27	EC_6.3.2.10	Cell Wall and Capsule	Cell Wall and Capsule
mraY	SMD_0641	1,90	2,11	EC_2.7.8.13	Cell Wall and Capsule	Cell Wall and Capsule
murG	SMD_0643	2,09	2,30	EC_2.4.1.227	Cell Wall and Capsule	Cell Wall and Capsule
murC	SMD_0644	2,05	2,01	EC_6.3.2.8	Cell Wall and Capsule	Cell Wall and Capsule
ddlB	SMD_0645	2,00	2,00	EC_6.3.2.4	Cell Wall and Capsule	Cell Wall and Capsule
ftsZ	SMD_0648	3,28	2,92	EC_3.4.24.-	Cell Division and Cell Cycle	Cell Division and Cell Cycle
metF	SMD_0654	3,71	4,36	EC_1.5.1.20	Amino Acids and Derivatives	Lysine, threonine, methionine, and cysteine
metF	SMD_0654	3,71	4,36	EC_1.5.1.20	Carbohydrates	One-carbon Metabolism
metF	SMD_0654	3,71	4,36	EC_1.5.1.20	Cofactors, Vitamins, Prosthetic Groups, Pigments	Folate and pterines
sahH	SMD_0664	2,37	2,22	EC_3.3.1.1	Amino Acids and Derivatives	Lysine, threonine, methionine, and cysteine
ppc	SMD_0666	2,96	3,20	EC_4.1.1.31	Carbohydrates	Central carbohydrate metabolism
metK	SMD_0668	2,61	2,22	EC_2.5.1.6	Amino Acids and Derivatives	Lysine, threonine, methionine, and cysteine
-	SMD_0672	2,71	4,23	EC_2.1.1.-	Membrane Transport	Protein and nucleoprotein secretion system, Type IV
rbsK	SMD_0675	2,59	2,56	EC_2.7.1.15	Carbohydrates	Monosaccharides
dgkA	SMD_0687	2,90	2,90	EC_2.7.1.107	Fatty Acids, Lipids, and Isoprenoids	Phospholipids
lgt	SMD_0693	2,78	3,85	EC_2.4.99.-	Protein Metabolism	Protein processing and modification
thyA	SMD_0694	2,72	2,88	EC_2.1.1.45	Cofactors, Vitamins, Prosthetic Groups, Pigments	Folate and pterines

thyA	SMD_0694	2,72	2,88	EC_2.1.1.45	Nucleosides and Nucleotides	Pyrimidines
surA	SMD_0701	2,59	2,72	EC_5.2.1.8	Cell Wall and Capsule	Gram-Negative cell wall components
surA	SMD_0701	2,59	2,72	EC_5.2.1.8	Protein Metabolism	Protein folding
ubiH	SMD_0706	3,17	2,82	EC_1.14.13.-	Cofactors, Vitamins, Prosthetic Groups, Pigments	Quinone cofactors
ubiF	SMD_0707	2,83	3,28	EC_1.14.13.-	Cofactors, Vitamins, Prosthetic Groups, Pigments	Quinone cofactors
glnS	SMD_0713	3,04	3,17	EC_6.1.1.18	Protein Metabolism	Protein biosynthesis
msrA2	SMD_0715	2,59	3,38	EC_1.8.4.11	Protein Metabolism	Protein processing and modification
talB	SMD_0716	2,49	2,46	EC_2.2.1.2	Carbohydrates	Central carbohydrate metabolism
talB	SMD_0716	2,49	2,46	EC_2.2.1.2	Carbohydrates	Monosaccharides
ahpC	SMD_0720	4,72	5,18	EC_1.11.1.15	Sulfur Metabolism	Sulfur Metabolism
nudF	SMD_0728	2,71	2,86	EC_3.6.1.13	Nucleosides and Nucleotides	Detoxification
-	SMD_0731	2,89	3,46	EC_2.4.2.4	Nucleosides and Nucleotides	Pyrimidines
ilvE	SMD_0740	2,75	2,81	EC_2.6.1.42	Amino Acids and Derivatives	Branched-chain amino acids
ilvE	SMD_0740	2,75	2,81	EC_2.6.1.42	Amino Acids and Derivatives	Alanine, serine, and glycine
ilvE	SMD_0740	2,75	2,81	EC_2.6.1.42	Carbohydrates	Central carbohydrate metabolism
ipk	SMD_0753	2,43	2,84	EC_2.7.1.148	Fatty Acids, Lipids, and Isoprenoids	Isoprenoids
prs	SMD_0755	3,48	3,70	EC_2.7.6.1	Carbohydrates	Central carbohydrate metabolism
prs	SMD_0755	3,48	3,70	EC_2.7.6.1	Nucleosides and Nucleotides	Purines
pth	SMD_0757	2,28	2,52	EC_3.1.1.29	Dormancy and Sporulation	Dormancy and Sporulation
pth	SMD_0757	2,28	2,52	EC_3.1.1.29	Protein Metabolism	Protein biosynthesis
-	SMD_0766	3,11	3,38	EC_3.4.19.5	Protein Metabolism	Protein degradation
rpoB	SMD_0778	2,35	2,33	EC_2.7.7.6	RNA Metabolism	Transcription
rpoB	SMD_0778	2,35	2,33	EC_2.7.7.6	Virulence, Disease and Defense	Invasion and intracellular resistance
rpoC	SMD_0779	2,11	2,11	EC_2.7.7.6	RNA Metabolism	Transcription
rpoC	SMD_0779	2,11	2,11	EC_2.7.7.6	Virulence, Disease and Defense	Invasion and intracellular resistance
rpoA	SMD_0809	2,32	2,29	EC_2.7.7.6	RNA Metabolism	Transcription
aroH	SMD_0813	3,43	3,68	EC_2.5.1.54	Amino Acids and Derivatives	Aromatic amino acids and derivatives
ppiB	SMD_0818	2,54	2,53	EC_5.2.1.8	Potassium metabolism	Potassium metabolism
mdh	SMD_0819	2,41	2,42	EC_1.1.1.37	Carbohydrates	Central carbohydrate metabolism
mdh	SMD_0819	2,41	2,42	EC_1.1.1.37	Carbohydrates	One-carbon Metabolism
rluA	SMD_0820	3,18	3,21	EC_4.2.1.70	Protein Metabolism	Protein biosynthesis
rluA	SMD_0820	3,18	3,21	EC_4.2.1.70	RNA Metabolism	RNA processing and modification
prpE	SMD_0821	2,61	2,89	EC_6.2.1.17	Carbohydrates	Organic acids
gst	SMD_0824	2,96	3,23	EC_2.5.1.18	Stress Response	Oxidative stress
kbl	SMD_0833	2,70	2,98	EC_2.3.1.29	Amino Acids and Derivatives	Alanine, serine, and glycine
tdh	SMD_0835	2,57	2,41	EC_1.1.1.103	Amino Acids and Derivatives	Lysine, threonine, methionine, and cysteine
tdh	SMD_0835	2,57	2,41	EC_1.1.1.103	Amino Acids and Derivatives	Alanine, serine, and glycine

folC	SMD_0838	2,80	2,97	EC_6.3.2.12, EC_6.3.2.17	Cofactors, Vitamins, Prosthetic Groups, Pigments	Folate and pterines
folC	SMD_0838	2,80	2,97	EC_6.3.2.12, EC_6.3.2.17	Virulence, Disease and Defense	Bacteriocins, ribosomally synthesized antibacterial peptides
purF	SMD_0841	2,77	2,90	EC_2.4.2.14	Nucleosides and Nucleotides	Purines
purF	SMD_0841	2,77	2,90	EC_2.4.2.14	Virulence, Disease and Defense	Bacteriocins, ribosomally synthesized antibacterial peptides
ppx	SMD_0849	4,15	5,24	EC_3.6.1.11	Phosphorus Metabolism	Phosphorus Metabolism
ppk	SMD_0850	3,03	3,27	EC_2.7.4.1	Nucleosides and Nucleotides	Purines
ppk	SMD_0850	3,03	3,27	EC_2.7.4.1	Phosphorus Metabolism	Phosphorus Metabolism
-	SMD_0856	2,07	2,38	EC_1.1.1.41	Carbohydrates	Central carbohydrate metabolism
tig	SMD_0869	2,85	2,94	EC_5.2.1.8	RNA Metabolism	RNA processing and modification
clpP	SMD_0870	2,56	2,62	EC_3.4.21.92	Protein Metabolism	Protein degradation
clpP	SMD_0870	2,56	2,62	EC_3.4.21.92	Regulation and Cell signaling	Regulation and Cell signaling
lon	SMD_0872	2,06	2,61	EC_3.4.21.53	Protein Metabolism	Protein degradation
ppiD	SMD_0879	2,72	2,80	EC_5.2.1.8	Protein Metabolism	Protein folding
mltD	SMD_0933	4,61	5,30	EC_3.2.1.-	Cell Wall and Capsule	Cell Wall and Capsule
gloB	SMD_0934	5,04	6,16	EC_3.1.2.6	Carbohydrates	Central carbohydrate metabolism
gloB	SMD_0934	5,04	6,16	EC_3.1.2.6	Stress Response	Oxidative stress
-	SMD_0935	3,43	3,37	EC_2.1.1.-	Stress Response	Oxidative stress
rnhA	SMD_0936	2,94	3,28	EC_3.1.26.4	RNA Metabolism	RNA processing and modification
dnaX	SMD_0964	2,70	3,08	EC_2.7.7.7	DNA Metabolism	DNA uptake, competence
-	SMD_0967	3,76	2,37	EC_3.6.1.17	Nucleosides and Nucleotides	Pyrimidines
fabH2	SMD_0976	2,34	2,78	EC_2.3.1.41	Fatty Acids, Lipids, and Isoprenoids	Fatty acids
fabD	SMD_0978	3,06	3,08	EC_2.3.1.39	Carbohydrates	Organic acids
fabD	SMD_0978	3,06	3,08	EC_2.3.1.39	Fatty Acids, Lipids, and Isoprenoids	Fatty acids
fabG	SMD_0979	2,72	2,89	EC_1.1.1.100	Fatty Acids, Lipids, and Isoprenoids	Fatty acids
fabF	SMD_0981	2,25	2,66	EC_2.3.1.41	Fatty Acids, Lipids, and Isoprenoids	Fatty acids
proC	SMD_1011	2,66	2,66	EC_1.5.1.2	Amino Acids and Derivatives	Proline and 4-hydroxyproline
tag	SMD_1019	3,75	3,19	EC_3.2.2.20	DNA Metabolism	DNA repair
pyrB	SMD_1023	3,07	2,95	EC_2.1.3.2	Nucleosides and Nucleotides	Pyrimidines
ptsI	SMD_1027	2,81	2,69	EC_2.7.3.9	Carbohydrates	Monosaccharides
-	SMD_1031	2,59	3,65	EC_2.7.1.-	Regulation and Cell signaling	Regulation and Cell signaling
kdsC	SMD_1038	2,60	3,51	EC_3.1.3.45	Cell Wall and Capsule	Gram-Negative cell wall components
kdsD	SMD_1039	3,01	3,62	EC_5.3.1.13	Cell Wall and Capsule	Gram-Negative cell wall components
murA	SMD_1041	2,88	2,88	EC_2.5.1.7	Cell Wall and Capsule	Cell Wall and Capsule
purN	SMD_1045	3,46	3,38	EC_2.1.2.2	Cofactors, Vitamins, Prosthetic Groups, Pigments	Folate and pterines
purN	SMD_1045	3,46	3,38	EC_2.1.2.2	Nucleosides and Nucleotides	Purines
purM	SMD_1049	3,48	3,64	EC_6.3.3.1	Nucleosides and Nucleotides	Purines
-	SMD_1075	1,95	2,39	EC_2.8.1.7	Amino Acids and Derivatives	Alanine, serine, and glycine

-	SMD_1075	1,95	2,39	EC_2.8.1.7	Miscellaneous	Plant-Prokaryote DOE project
-	SMD_1075	1,95	2,39	EC_2.8.1.7	RNA Metabolism	RNA processing and modification
lysA	SMD_1089	2,79	2,87	EC_2.7.2.4, EC_4.1.1.20	Amino Acids and Derivatives	Lysine, threonine, methionine, and cysteine
murD	SMD_1091	2,19	1,89	EC_6.3.2.9	Cell Wall and Capsule	Cell Wall and Capsule
ispB	SMD_1096	3,51	2,98	EC_2.5.1.1, EC_2.5.1.10	Fatty Acids, Lipids, and Isoprenoids	Isoprenoids
-	SMD_1143	2,29	2,83	EC_3.2.2.1	Nucleosides and Nucleotides	Purines
-	SMD_1143	2,29	2,83	EC_3.2.2.1	RNA Metabolism	RNA processing and modification
mqo	SMD_1155	3,30	4,35	EC_1.1.5.4	Carbohydrates	Central carbohydrate metabolism
-	SMD_1174	2,34	3,04	EC_3.4.16.4	Cell Wall and Capsule	Cell Wall and Capsule
-	SMD_1174	2,34	3,04	EC_3.4.16.4	Protein Metabolism	Protein degradation
tesB	SMD_1185	2,71	3,79	EC_3.1.2.-	Fatty Acids, Lipids, and Isoprenoids	Fatty acids
-	SMD_1186	2,02	2,82	EC_4.2.1.17	Amino Acids and Derivatives	Branched-chain amino acids
-	SMD_1186	2,02	2,82	EC_4.2.1.17	Fatty Acids, Lipids, and Isoprenoids	Fatty Acids, Lipids, and Isoprenoids
-	SMD_1188	2,52	2,31	EC_4.2.1.11	Carbohydrates	Central carbohydrate metabolism
-	SMD_1188	2,52	2,31	EC_4.2.1.11	Carbohydrates	One-carbon Metabolism
ribF	SMD_1196	2,56	3,14	EC_2.7.1.26, EC_2.7.7.2	Cofactors, Vitamins, Prosthetic Groups, Pigments	Riboflavin, FMN, FAD
ileS	SMD_1197	2,39	2,29	EC_6.1.1.5	Protein Metabolism	Protein biosynthesis
lspA	SMD_1198	1,82	2,35	EC_3.4.23.36	Protein Metabolism	Protein processing and modification
ispH	SMD_1199	2,20	2,24	EC_1.17.1.2	Fatty Acids, Lipids, and Isoprenoids	Isoprenoids
cyoA	SMD_1213	2,45	2,22	EC_1.10.3.-	Respiration	Electron accepting reactions
cyoB	SMD_1214	2,19	2,09	EC_1.10.3.-	Respiration	Electron accepting reactions
cyoC	SMD_1215	2,14	1,99	EC_1.10.3.-	Respiration	Electron accepting reactions
cyoD	SMD_1216	1,73	2,28	EC_1.10.3.-	Respiration	Electron accepting reactions
-	SMD_1225	3,29	2,29	EC_4.1.3.38	Amino Acids and Derivatives	Aromatic amino acids and derivatives
-	SMD_1225	3,29	2,29	EC_4.1.3.38	Cofactors, Vitamins, Prosthetic Groups, Pigments	Folate and pterines
katA2	SMD_1237	3,18	3,60	EC_1.11.1.6	Stress Response	Oxidative stress
nfi	SMD_1267	3,72	5,10	EC_3.1.21.7	DNA Metabolism	DNA repair
gpmA	SMD_1268	3,09	3,27	EC_5.4.2.1	Carbohydrates	Central carbohydrate metabolism
gpmA	SMD_1268	3,09	3,27	EC_5.4.2.1	Miscellaneous	Miscellaneous
gltX	SMD_1279	2,43	2,92	EC_6.1.1.17	Cofactors, Vitamins, Prosthetic Groups, Pigments	Tetrapyrroles
gltX	SMD_1279	2,43	2,92	EC_6.1.1.17	Protein Metabolism	Protein biosynthesis
-	SMD_1293	2,94	3,36	EC_1.14.13.-	Cofactors, Vitamins, Prosthetic Groups, Pigments	Quinone cofactors
-	SMD_1305	3,57	3,33	EC_3.5.2.6	Virulence, Disease and Defense	Resistance to antibiotics and toxic compounds
accA	SMD_1321	2,20	2,24	EC_6.4.1.2	Fatty Acids, Lipids, and Isoprenoids	Fatty acids
dnaE	SMD_1323	2,40	2,61	EC_2.7.7.7	Phages, Prophages, Transposable elements, Plasmids	Phages, Prophages
rnhB	SMD_1324	2,43	2,19	EC_3.1.26.4	RNA Metabolism	RNA processing and modification
lpxB	SMD_1325	2,38	2,60	EC_2.4.1.182	Cell Wall and Capsule	Gram-Negative cell wall components

lpxA	SMD_1326	2,22	2,44	EC_2.3.1.129	Cell Wall and Capsule	Gram-Negative cell wall components
fabZ	SMD_1327	2,46	2,46	EC_4.2.1.-	Fatty Acids, Lipids, and Isoprenoids	Fatty acids
lpxD	SMD_1328	2,24	2,68	EC_2.3.1.-	Cell Wall and Capsule	Gram-Negative cell wall components
dxr	SMD_1331	2,01	2,35	EC_1.1.1.267	Fatty Acids, Lipids, and Isoprenoids	Isoprenoids
cdsA	SMD_1332	2,40	2,62	EC_2.7.7.41	Fatty Acids, Lipids, and Isoprenoids	Phospholipids
uppS	SMD_1333	2,49	2,42	EC_2.5.1.31	Fatty Acids, Lipids, and Isoprenoids	Isoprenoids
map	SMD_1345	2,41	2,24	EC_3.4.11.18	Protein Metabolism	Protein biosynthesis
glnD	SMD_1346	2,44	2,63	EC_2.7.7.59	Nitrogen Metabolism	Nitrogen Metabolism
dapD	SMD_1347	2,12	1,98	EC_2.3.1.117	Amino Acids and Derivatives	Lysine, threonine, methionine, and cysteine
dapE	SMD_1350	2,42	2,34	EC_3.5.1.18	Amino Acids and Derivatives	Lysine, threonine, methionine, and cysteine
parC	SMD_1356	2,44	2,55	EC_5.99.1.-	DNA Metabolism	DNA replication
parC	SMD_1356	2,44	2,55	EC_5.99.1.-	Virulence, Disease and Defense	Resistance to antibiotics and toxic compounds
rnt	SMD_1375	3,51	3,54	EC_3.1.13.-	RNA Metabolism	RNA processing and modification
nth	SMD_1387	1,52	1,39	EC_4.2.99.18	DNA Metabolism	DNA repair
crt2	SMD_1389	3,48	3,09	EC_4.2.1.17	Amino Acids and Derivatives	Branched-chain amino acids
crt2	SMD_1389	3,48	3,09	EC_4.2.1.17	Fatty Acids, Lipids, and Isoprenoids	Fatty Acids, Lipids, and Isoprenoids
nadC	SMD_1437	2,30	2,89	EC_2.4.2.19	Cofactors, Vitamins, Prosthetic Groups, Pigments	NAD and NADP
purE	SMD_1439	2,75	3,85	EC_4.1.1.21	Nucleosides and Nucleotides	Purines
purK	SMD_1440	2,51	3,03	EC_4.1.1.21	Nucleosides and Nucleotides	Purines
lpxK	SMD_1467	2,10	2,06	EC_2.7.1.130	Cell Wall and Capsule	Gram-Negative cell wall components
kdsB	SMD_1468	2,28	2,10	EC_2.7.7.38	Cell Wall and Capsule	Gram-Negative cell wall components
pgsA	SMD_1472	2,31	2,27	EC_2.7.8.5	Fatty Acids, Lipids, and Isoprenoids	Phospholipids
pgsA	SMD_1472	2,31	2,27	EC_2.7.8.5	Regulation and Cell signaling	Regulation of virulence
-	SMD_1583	2,15	2,57	EC_2.8.1.7	Amino Acids and Derivatives	Alanine, serine, and glycine
-	SMD_1583	2,15	2,57	EC_2.8.1.7	Miscellaneous	Plant-Prokaryote DOE project
-	SMD_1583	2,15	2,57	EC_2.8.1.7	RNA Metabolism	RNA processing and modification
-	SMD_1634	2,34	3,25	EC_1.4.1.13	Nitrogen Metabolism	Nitrogen Metabolism
parE	SMD_1648	2,00	2,44	EC_5.99.1.-	DNA Metabolism	DNA replication
parE	SMD_1648	2,00	2,44	EC_5.99.1.-	Virulence, Disease and Defense	Resistance to antibiotics and toxic compounds
pyrG	SMD_1651	2,75	2,40	EC_6.3.4.2	Nucleosides and Nucleotides	Pyrimidines
kdsA	SMD_1652	2,64	2,85	EC_2.5.1.55	Cell Wall and Capsule	Gram-Negative cell wall components
eno	SMD_1655	2,60	2,81	EC_4.2.1.11	Carbohydrates	One-carbon Metabolism
eno	SMD_1655	2,60	2,81	EC_4.2.1.11	Carbohydrates	Central carbohydrate metabolism
ispD	SMD_1657	2,14	2,54	EC_2.7.7.60	Fatty Acids, Lipids, and Isoprenoids	Isoprenoids
ispF	SMD_1658	1,79	2,34	EC_4.6.1.12	Fatty Acids, Lipids, and Isoprenoids	Isoprenoids
truD	SMD_1659	2,30	1,98	EC_4.2.1.-	RNA Metabolism	RNA processing and modification
surE	SMD_1661	2,99	3,24	EC_3.1.3.5	Nucleosides and Nucleotides	Purines

surE	SMD_1661	2,99	3,24	EC_3.1.3.5	Nucleosides and Nucleotides	Detoxification
surE	SMD_1661	2,99	3,24	EC_3.1.3.5	Nucleosides and Nucleotides	Pyrimidines
pcm	SMD_1662	3,10	2,17	EC_2.1.1.77	Membrane Transport	Membrane Transport
pcm	SMD_1662	3,10	2,17	EC_2.1.1.77	Protein Metabolism	Protein processing and modification
pcm	SMD_1662	3,10	2,17	EC_2.1.1.77	Secondary Metabolism	Secondary Metabolism
ftsH	SMD_1669	2,48	3,02	EC_3.4.24.-	Cofactors, Vitamins, Prosthetic Groups, Pigments	Folate and pterines
folP	SMD_1671	2,49	2,67	EC_2.5.1.15	Cofactors, Vitamins, Prosthetic Groups, Pigments	Folate and pterines
lexA	SMD_1677	3,98	5,66	EC_3.4.21.88	DNA Metabolism	DNA repair
alaS	SMD_1680	2,73	2,81	EC_6.1.1.7	Protein Metabolism	Protein biosynthesis
-	SMD_1707	1,51	2,75	EC_1.11.1.15	Sulfur Metabolism	Sulfur Metabolism
dapA	SMD_1709	3,31	3,46	EC_4.3.3.7	Amino Acids and Derivatives	Lysine, threonine, methionine, and cysteine
pcnB	SMD_1714	2,72	2,65	EC_2.7.7.19	RNA Metabolism	RNA processing and modification
folK	SMD_1715	1,52	2,96	EC_2.7.6.3	Cofactors, Vitamins, Prosthetic Groups, Pigments	Folate and pterines
panB	SMD_1716	2,68	2,49	EC_2.1.2.11	Cofactors, Vitamins, Prosthetic Groups, Pigments	Coenzyme A
panC	SMD_1717	2,78	2,60	EC_6.3.2.1	Cofactors, Vitamins, Prosthetic Groups, Pigments	Folate and pterines
panC	SMD_1717	2,78	2,60	EC_6.3.2.1	Cofactors, Vitamins, Prosthetic Groups, Pigments	Coenzyme A
panD	SMD_1718	2,62	2,72	EC_4.1.1.11	Cofactors, Vitamins, Prosthetic Groups, Pigments	Folate and pterines
panD	SMD_1718	2,62	2,72	EC_4.1.1.11	Cofactors, Vitamins, Prosthetic Groups, Pigments	Coenzyme A
pgi	SMD_1719	2,46	2,71	EC_5.3.1.9	Carbohydrates	Central carbohydrate metabolism
-	SMD_1721	3,42	4,85	EC_2.7.3.-	Regulation and Cell signaling	Regulation and Cell signaling
ispG	SMD_1722	2,33	3,74	EC_1.17.7.1	Fatty Acids, Lipids, and Isoprenoids	Isoprenoids
kgdA	SMD_1725	1,70	2,37	EC_4.1.2.14	Carbohydrates	Central carbohydrate metabolism
edd	SMD_1726	2,33	2,28	EC_4.2.1.12	Carbohydrates	Central carbohydrate metabolism
zwf	SMD_1729	3,30	3,15	EC_1.1.1.49	Carbohydrates	Central carbohydrate metabolism
sdhA	SMD_1734	2,22	2,47	EC_1.3.99.1	Carbohydrates	Central carbohydrate metabolism
sdhA	SMD_1734	2,22	2,47	EC_1.3.99.1	Respiration	Electron donating reactions
sdhB	SMD_1735	1,97	1,92	EC_1.3.99.1	Carbohydrates	Central carbohydrate metabolism
sdhB	SMD_1735	1,97	1,92	EC_1.3.99.1	Carbohydrates	One-carbon Metabolism
sdhB	SMD_1735	1,97	1,92	EC_1.3.99.1	Cofactors, Vitamins, Prosthetic Groups, Pigments	Folate and pterines
coaD	SMD_1747	2,86	3,10	EC_2.7.7.3	Cofactors, Vitamins, Prosthetic Groups, Pigments	Coenzyme A
ggt	SMD_1750	2,38	2,35	EC_2.3.2.2	Stress Response	Oxidative stress
-	SMD_1754	3,01	3,77	EC_2.5.1.18	Stress Response	Oxidative stress
upp	SMD_1755	2,87	2,95	EC_2.4.2.9	Nucleosides and Nucleotides	Pyrimidines
pdxK	SMD_1798	2,95	2,84	EC_2.7.1.35	Cofactors, Vitamins, Prosthetic Groups, Pigments	Pyridoxine
queA	SMD_1808	3,11	3,06	EC_5.-.-.-	Miscellaneous	Plant-Prokaryote DOE project
queA	SMD_1808	3,11	3,06	EC_5.-.-.-	RNA Metabolism	RNA processing and modification
tgt	SMD_1809	2,83	2,61	EC_2.4.2.29	Miscellaneous	Plant-Prokaryote DOE project

tgt	SMD_1809	2,83	2,61	EC_2.4.2.29	RNA Metabolism	RNA processing and modification
apt	SMD_1822	1,99	2,53	EC_2.4.2.7	Nucleosides and Nucleotides	Purines
apt	SMD_1822	1,99	2,53	EC_2.4.2.7	Regulation and Cell signaling	Regulation and Cell signaling
-	SMD_1846	2,27	2,00	EC_2.3.1.16	Amino Acids and Derivatives	Branched-chain amino acids
-	SMD_1846	2,27	2,00	EC_2.3.1.16, EC_2.3.1.9	Carbohydrates	One-carbon Metabolism
-	SMD_1846	2,27	2,00	EC_2.3.1.9	Carbohydrates	Fermentation
-	SMD_1846	2,27	2,00	EC_2.3.1.16	Cofactors, Vitamins, Prosthetic Groups, Pigments	Biotin
-	SMD_1846	2,27	2,00	EC_2.3.1.16, EC_2.3.1.9	Fatty Acids, Lipids, and Isoprenoids	Fatty Acids, Lipids, and Isoprenoids
-	SMD_1846	2,27	2,00	EC_2.3.1.9	Fatty Acids, Lipids, and Isoprenoids	Isoprenoids
-	SMD_1847	1,82	2,25	EC_1.1.1.35, EC_4.2.1.17	Amino Acids and Derivatives	Branched-chain amino acids
-	SMD_1847	1,82	2,25	EC_1.1.1.35	Carbohydrates	Fermentation
-	SMD_1847	1,82	2,25	EC_1.1.1.35	Fatty Acids, Lipids, and Isoprenoids	Fatty acids
-	SMD_1847	1,82	2,25	EC_4.2.1.17	Fatty Acids, Lipids, and Isoprenoids	Fatty Acids, Lipids, and Isoprenoids
ndk	SMD_1849	2,49	2,51	EC_2.7.4.6	Nucleosides and Nucleotides	Pyrimidines
ndk	SMD_1849	2,49	2,51	EC_2.7.4.6	Nucleosides and Nucleotides	Purines
rlmN	SMD_1850	2,14	2,65	EC_2.1.1.-	RNA Metabolism	RNA processing and modification
folD	SMD_1858	2,76	2,93	EC_1.5.1.5, EC_3.5.4.9	Carbohydrates	One-carbon Metabolism
folD	SMD_1858	2,76	2,93	EC_1.5.1.5	Cofactors, Vitamins, Prosthetic Groups, Pigments	Folate and pterines
guaB	SMD_1859	2,60	3,14	EC_1.1.1.205	Nucleosides and Nucleotides	Purines
guaA	SMD_1861	2,02	2,19	EC_6.3.5.2	Nucleosides and Nucleotides	Purines
hsdR	SMD_1862	2,72	3,13	EC_3.1.21.3	DNA Metabolism	DNA Metabolism
hsdM	SMD_1863	2,22	2,26	EC_2.1.1.72	DNA Metabolism	DNA Metabolism
hsdS	SMD_1864	1,84	1,85	EC_3.1.21.3	DNA Metabolism	DNA Metabolism
pyrD	SMD_1915	2,73	2,65	EC_1.3.98.1	Carbohydrates	One-carbon Metabolism
-	SMD_1918	2,48	2,17	EC_1.1.1.100	Fatty Acids, Lipids, and Isoprenoids	Fatty acids
-	SMD_1919	2,40	2,32	EC_1.2.1.22	Carbohydrates	Central carbohydrate metabolism
-	SMD_1919	2,40	2,32	EC_1.2.1.22	Fatty Acids, Lipids, and Isoprenoids	Phospholipids
-	SMD_1934	2,20	3,26	EC_3.5.1.24	Virulence, Disease and Defense	Resistance to antibiotics and toxic compounds
thrB	SMD_1940	2,29	2,74	EC_2.7.1.39	Amino Acids and Derivatives	Lysine, threonine, methionine, and cysteine
thrC	SMD_1941	2,96	2,96	EC_4.2.3.1	Amino Acids and Derivatives	Lysine, threonine, methionine, and cysteine
hisS	SMD_1943	2,63	2,73	EC_6.1.1.21	Protein Metabolism	Protein biosynthesis
hisB	SMD_1949	1,43	1,76	EC_4.2.1.19, EC_3.1.3.15	Amino Acids and Derivatives	Histidine Metabolism
hisA	SMD_1951	2,35	1,80	EC_5.3.1.16	Amino Acids and Derivatives	Histidine Metabolism
hisA	SMD_1951	2,35	1,80	EC_5.3.1.16	Amino Acids and Derivatives	Aromatic amino acids and derivatives
hisF	SMD_1952	2,50	1,52	EC_4.1.3.-	Amino Acids and Derivatives	Histidine Metabolism
-	SMD_1956	2,31	2,99	EC_2.7.1.59	Carbohydrates	Aminosugars
actP	SMD_1960	1,99	2,87	EC_3.6.3.4	Membrane Transport	Cation transporters

actP	SMD_1960	1,99	2,87	EC_3.6.3.4	Virulence, Disease and Defense	Resistance to antibiotics and toxic compounds
scrK	SMD_1967	2,04	2,04	EC_2.7.1.4	Carbohydrates	Monosaccharides
-	SMD_1968	2,01	1,86	EC_5.3.1.7	Carbohydrates	Monosaccharides
bmnA	SMD_1969	2,28	2,06	EC_3.2.1.25	Carbohydrates	Monosaccharides
masA	SMD_1970	2,92	2,31	EC_3.1.3.77	Amino Acids and Derivatives	Lysine, threonine, methionine, and cysteine
mtnD	SMD_1971	3,29	2,62	EC_1.13.11.54	Amino Acids and Derivatives	Lysine, threonine, methionine, and cysteine
mtnB	SMD_1972	2,54	3,03	EC_4.2.1.109	Amino Acids and Derivatives	Lysine, threonine, methionine, and cysteine
serA	SMD_1976	2,80	3,12	EC_1.1.1.95	Amino Acids and Derivatives	Alanine, serine, and glycine
serA	SMD_1976	2,80	3,12	EC_1.1.1.95	Cofactors, Vitamins, Prosthetic Groups, Pigments	Pyridoxine
hmgcL	SMD_1988	2,55	2,96	EC_4.1.3.4	Amino Acids and Derivatives	Branched-chain amino acids
-	SMD_1990	1,44	2,28	EC_4.2.1.18	Amino Acids and Derivatives	Branched-chain amino acids
dapB	SMD_1998	2,37	2,32	EC_1.17.1.8	Amino Acids and Derivatives	Lysine, threonine, methionine, and cysteine
carA	SMD_1999	2,78	2,86	EC_6.3.5.5	Cell Division and Cell Cycle	Cell Division and Cell Cycle
carA	SMD_1999	2,78	2,86	EC_6.3.5.5	Nucleosides and Nucleotides	Pyrimidines
carB	SMD_2000	2,11	2,36	EC_6.3.5.5	Cell Division and Cell Cycle	Cell Division and Cell Cycle
carB	SMD_2000	2,11	2,36	EC_6.3.5.5	Nucleosides and Nucleotides	Pyrimidines
recJ	SMD_2004	3,06	3,08	EC_3.1.-.-	DNA Metabolism	DNA repair
lysS	SMD_2013	2,67	2,66	EC_6.1.1.6	Protein Metabolism	Protein biosynthesis
fadD	SMD_2017	2,97	3,55	EC_6.2.1.3	Cofactors, Vitamins, Prosthetic Groups, Pigments	Biotin
fadD	SMD_2017	2,97	3,55	EC_6.2.1.3	Fatty Acids, Lipids, and Isoprenoids	Fatty acids
betA	SMD_2018	2,84	2,85	EC_1.1.99.1	Stress Response	Osmotic stress
acn	SMD_2022	2,49	2,57	EC_4.2.1.3	Carbohydrates	Central carbohydrate metabolism
acn	SMD_2022	2,49	2,57	EC_4.2.1.3	Carbohydrates	One-carbon Metabolism
acn	SMD_2022	2,49	2,57	EC_4.2.1.3, EC_4.2.1.99	Carbohydrates	Organic acids
acnB	SMD_2025	2,27	2,28	EC_4.2.1.3	Carbohydrates	Central carbohydrate metabolism
acnB	SMD_2025	2,27	2,28	EC_4.2.1.3, EC_4.2.1.99	Carbohydrates	Organic acids
cheA	SMD_2036	2,30	2,47	EC_2.7.3.-	Motility and Chemotaxis	Flagellar motility in Prokaryota
cheA2	SMD_2043	2,36	2,29	EC_2.7.3.-	Motility and Chemotaxis	Motility and Chemotaxis
trxB	SMD_2112	2,85	2,50	EC_1.8.1.9	Nucleosides and Nucleotides	Pyrimidines
trxB	SMD_2112	2,85	2,50	EC_1.8.1.9	Sulfur Metabolism	Sulfur Metabolism
lspA2	SMD_2136	1,14	1,34	EC_3.4.23.36	Protein Metabolism	Protein processing and modification
-	SMD_2165	2,51	3,17	EC_2.7.3.-	Stress Response	Oxidative stress
-	SMD_2220	2,18	1,70	EC_3.3.2.1	Amino Acids and Derivatives	Aromatic amino acids and derivatives
-	SMD_2220	2,18	1,70	EC_3.3.2.1	Cofactors, Vitamins, Prosthetic Groups, Pigments	NAD and NADP
-	SMD_2225	2,09	2,72	EC_2.7.3.9	Carbohydrates	Monosaccharides
fruK	SMD_2226	1,31	1,57	EC_2.7.1.56	Carbohydrates	Monosaccharides
fruA	SMD_2227	2,04	2,61	EC_2.7.1.69	Carbohydrates	Monosaccharides

metE	SMD_2261	4,69	3,83	EC_2.1.1.14	Amino Acids and Derivatives	Lysine, threonine, methionine, and cysteine
sodA2	SMD_2474	3,88	2,74	EC_1.15.1.1	Stress Response	Oxidative stress
cdh	SMD_2539	3,59	1,92	EC_3.6.1.26	Fatty Acids, Lipids, and Isoprenoids	Phospholipids
dld	SMD_2543	1,93	2,08	EC_1.1.2.5	Carbohydrates	Organic acids
dld	SMD_2543	1,93	2,08	EC_1.1.2.5	Respiration	Electron donating reactions
lctD	SMD_2544	1,91	2,23	EC_1.1.2.3	Carbohydrates	Organic acids
lctD	SMD_2544	1,91	2,23	EC_1.1.2.3	Respiration	Electron donating reactions
adi	SMD_2577	2,52	3,87	EC_4.1.1.18	Amino Acids and Derivatives	Lysine, threonine, methionine, and cysteine
pssA2	SMD_2607	3,31	2,30	EC_2.7.8.8	Fatty Acids, Lipids, and Isoprenoids	Phospholipids
pps	SMD_2613	2,94	2,96	EC_2.7.9.2	Carbohydrates	Central carbohydrate metabolism
-	SMD_2620	3,21	4,45	EC_1.6.99.-	Cofactors, Vitamins, Prosthetic Groups, Pigments	Riboflavin, FMN, FAD
serS	SMD_2677	3,04	3,07	EC_6.1.1.11	Amino Acids and Derivatives	Alanine, serine, and glycine
serS	SMD_2677	3,04	3,07	EC_6.1.1.11	Protein Metabolism	Protein biosynthesis
aroA	SMD_2680	2,59	2,65	EC_2.5.1.19	Amino Acids and Derivatives	Aromatic amino acids and derivatives
pheA	SMD_2681	2,63	2,85	EC_4.2.1.51, EC_5.4.99.5	Amino Acids and Derivatives	Aromatic amino acids and derivatives
serC	SMD_2682	3,17	3,57	EC_2.6.1.52	Amino Acids and Derivatives	Alanine, serine, and glycine
serC	SMD_2682	3,17	3,57	EC_2.6.1.52	Cofactors, Vitamins, Prosthetic Groups, Pigments	Pyridoxine
hutH	SMD_2691	3,58	3,05	EC_4.3.1.3	Amino Acids and Derivatives	Histidine Metabolism
-	SMD_2713	2,56	2,68	EC_1.6.5.5	Respiration	Respiration
gyrA	SMD_2717	2,67	2,97	EC_5.99.1.3	DNA Metabolism	DNA replication
gyrA	SMD_2717	2,67	2,97	EC_5.99.1.3	Virulence, Disease and Defense	Resistance to antibiotics and toxic compounds
mtnA	SMD_2718	2,70	3,96	EC_5.3.1.23	Amino Acids and Derivatives	Lysine, threonine, methionine, and cysteine
-	SMD_2723	2,82	3,97	EC_2.6.1.57	Amino Acids and Derivatives	Aromatic amino acids and derivatives
asnC	SMD_2731	2,87	2,88	EC_6.1.1.22	Protein Metabolism	Protein biosynthesis
-	SMD_2735	2,17	2,82	EC_4.2.1.1	Regulation and Cell signaling	Regulation and Cell signaling
nbaC	SMD_2736	2,06	2,24	EC_1.13.11.6	Cofactors, Vitamins, Prosthetic Groups, Pigments	NAD and NADP
-	SMD_2737	2,64	2,83	EC_3.7.1.3	Cofactors, Vitamins, Prosthetic Groups, Pigments	NAD and NADP
sbcB	SMD_2739	2,31	2,50	EC_3.1.11.1	DNA Metabolism	DNA repair
-	SMD_2743	2,90	3,33	EC_3.1.3.5	Nucleosides and Nucleotides	Pyrimidines
-	SMD_2743	2,90	3,33	EC_3.1.3.5	Nucleosides and Nucleotides	Purines
-	SMD_2744	2,76	2,78	EC_2.7.1.23	Cofactors, Vitamins, Prosthetic Groups, Pigments	NAD and NADP
metH2	SMD_2753	3,25	3,05	EC_2.1.1.13	Amino Acids and Derivatives	Lysine, threonine, methionine, and cysteine
metH	SMD_2754	2,42	2,55	EC_2.1.1.13	Amino Acids and Derivatives	Lysine, threonine, methionine, and cysteine
-	SMD_2760	2,92	1,99	EC_5.2.1.8	Protein Metabolism	Protein folding
fumC	SMD_2769	3,05	3,12	EC_4.2.1.2	Carbohydrates	Central carbohydrate metabolism
purB	SMD_2770	3,24	3,13	EC_4.3.2.2	Nucleosides and Nucleotides	Purines
sucA	SMD_2774	2,38	2,31	EC_1.2.4.2	Carbohydrates	Central carbohydrate metabolism

sucB	SMD_2775	2,21	2,02	EC_2.3.1.61	Carbohydrates	Central carbohydrate metabolism
odhL	SMD_2776	1,99	1,90	EC_1.8.1.4	Carbohydrates	Central carbohydrate metabolism
-	SMD_2796	2,96	2,64	EC_3.1.1.32	Fatty Acids, Lipids, and Isoprenoids	Triacylglycerols
gst4	SMD_2798	4,24	3,06	EC_2.5.1.18	Stress Response	Oxidative stress
-	SMD_2800	2,57	2,44	EC_4.2.1.2	Carbohydrates	Central carbohydrate metabolism
-	SMD_2800	2,57	2,44	EC_4.2.1.2	Carbohydrates	One-carbon Metabolism
fpr	SMD_2804	3,01	2,63	EC_1.18.1.2	Respiration	Respiration
-	SMD_2806	2,84	2,50	EC_3.4.15.5	Protein Metabolism	Protein degradation
sodA	SMD_2814	2,98	3,06	EC_1.15.1.1	Stress Response	Oxidative stress
rluC	SMD_2822	2,45	3,00	EC_4.2.1.70	Protein Metabolism	Protein biosynthesis
rluC	SMD_2822	2,45	3,00	EC_4.2.1.70	RNA Metabolism	RNA processing and modification
rnE	SMD_2823	2,48	2,71	EC_3.1.26.12	Protein Metabolism	Protein biosynthesis
rnE	SMD_2823	2,48	2,71	EC_3.1.26.12	RNA Metabolism	RNA processing and modification
rluB	SMD_2836	2,52	2,48	EC_4.2.1.70	RNA Metabolism	RNA processing and modification
metX	SMD_2851	3,27	3,43	EC_2.3.1.31	Amino Acids and Derivatives	Lysine, threonine, methionine, and cysteine
cydA	SMD_2855	2,61	2,20	EC_1.10.3.-	Respiration	Electron accepting reactions
cydB	SMD_2856	1,91	1,88	EC_1.10.3.-	Respiration	Electron accepting reactions
proA	SMD_2865	2,72	2,34	EC_1.2.1.41	Amino Acids and Derivatives	Proline and 4-hydroxyproline
proB	SMD_2866	1,92	2,23	EC_2.7.2.11	Amino Acids and Derivatives	Proline and 4-hydroxyproline
cysS	SMD_2877	3,21	2,97	EC_6.1.1.16	Protein Metabolism	Protein biosynthesis
cysS	SMD_2877	3,21	2,97	EC_6.1.1.16	Regulation and Cell signaling	Regulation and Cell signaling
pyrC	SMD_2882	2,61	2,77	EC_3.5.2.3	Nucleosides and Nucleotides	Pyrimidines
pyrC	SMD_2882	2,61	2,77	EC_3.5.2.3	Regulation and Cell signaling	Regulation and Cell signaling
amiC	SMD_2901	3,93	4,47	EC_3.5.1.28	Cell Wall and Capsule	Cell Wall and Capsule
amiC	SMD_2901	3,93	4,47	EC_3.5.1.28	Regulation and Cell signaling	Regulation and Cell signaling
ex7L	SMD_2905	2,79	3,03	EC_3.1.11.6	DNA Metabolism	DNA repair
dxs	SMD_2922	2,76	3,64	EC_2.2.1.7	Cofactors, Vitamins, Prosthetic Groups, Pigments	Cofactors, Vitamins, Prosthetic Groups, Pigments
dxs	SMD_2922	2,76	3,64	EC_2.2.1.7	Cofactors, Vitamins, Prosthetic Groups, Pigments	Pyridoxine
dxs	SMD_2922	2,76	3,64	EC_2.2.1.7	Fatty Acids, Lipids, and Isoprenoids	Isoprenoids
pheT	SMD_2947	2,51	2,69	EC_6.1.1.20	Protein Metabolism	Protein biosynthesis
pheS	SMD_2948	2,93	3,60	EC_6.1.1.20	Protein Metabolism	Protein biosynthesis
thrS	SMD_2952	2,66	2,79	EC_6.1.1.3	Protein Metabolism	Protein biosynthesis
chiA2	SMD_2957	2,43	2,97	EC_3.2.1.14	Carbohydrates	Aminosugars
pnp	SMD_2958	2,22	2,22	EC_2.7.7.8	RNA Metabolism	RNA processing and modification
truB	SMD_2960	2,38	2,01	EC_4.2.1.70	Cofactors, Vitamins, Prosthetic Groups, Pigments	Riboflavin, FMN, FAD
truB	SMD_2960	2,38	2,01	EC_4.2.1.70	RNA Metabolism	RNA processing and modification
nuoN	SMD_2967	1,98	1,91	EC_1.6.5.3	Respiration	Electron donating reactions

nuoM	SMD_2968	1,91	1,85	EC_1.6.5.3	Respiration	Electron donating reactions
nuoL	SMD_2969	1,80	1,92	EC_1.6.5.3	Respiration	Electron donating reactions
nuoK	SMD_2970	1,92	2,09	EC_1.6.5.3	Respiration	Electron donating reactions
nuoJ	SMD_2971	2,12	2,08	EC_1.6.5.3	Respiration	Electron donating reactions
nuoI	SMD_2972	1,99	1,66	EC_1.6.5.3	Respiration	Electron donating reactions
nuoH	SMD_2973	2,06	2,00	EC_1.6.5.3	Respiration	Electron donating reactions
nuoG	SMD_2974	2,02	2,05	EC_1.6.5.3	Respiration	Electron donating reactions
nuoF	SMD_2975	2,01	1,91	EC_1.6.5.3	Respiration	Electron donating reactions
nuoE	SMD_2976	1,97	1,83	EC_1.6.5.3	Respiration	Electron donating reactions
nuoD	SMD_2977	2,19	2,29	EC_1.6.5.3	Respiration	Electron donating reactions
nuoC	SMD_2978	2,59	2,56	EC_1.6.5.3	Respiration	Electron donating reactions
nuoB	SMD_2979	2,73	2,95	EC_1.6.5.3	Respiration	Electron donating reactions
nuoA	SMD_2980	3,35	3,58	EC_1.6.5.3	Respiration	Electron donating reactions
tpiA	SMD_2983	3,14	3,01	EC_5.3.1.1	Carbohydrates	Central carbohydrate metabolism
-	SMD_2985	3,35	3,80	EC_3.1.6.6	Stress Response	Osmotic stress
glmM	SMD_2988	2,46	2,66	EC_5.4.2.10	Cell Wall and Capsule	Capsular and extracellular polysacchrides
glmM	SMD_2988	2,46	2,66	EC_5.4.2.10	Cell Wall and Capsule	Cell Wall and Capsule
accD	SMD_2989	2,43	2,65	EC_6.4.1.2	Fatty Acids, Lipids, and Isoprenoids	Fatty acids
accD	SMD_2989	2,43	2,65	EC_6.4.1.2	Virulence, Disease and Defense	Bacteriocins, ribosomally synthesized antibacterial peptides
trpA	SMD_2990	2,15	2,78	EC_4.2.1.20	Amino Acids and Derivatives	Aromatic amino acids and derivatives
trpA	SMD_2990	2,15	2,78	EC_4.2.1.20	Secondary Metabolism	Plant Hormones
trpB	SMD_2992	3,53	3,39	EC_4.2.1.20	Amino Acids and Derivatives	Aromatic amino acids and derivatives
trpB	SMD_2992	3,53	3,39	EC_4.2.1.20	Secondary Metabolism	Plant Hormones
truA	SMD_3000	2,29	2,40	EC_4.2.1.70	RNA Metabolism	RNA processing and modification
truA	SMD_3000	2,29	2,40	EC_4.2.1.70	Virulence, Disease and Defense	Bacteriocins, ribosomally synthesized antibacterial peptides
asd	SMD_3003	2,80	2,46	EC_1.2.1.11	Amino Acids and Derivatives	Lysine, threonine, methionine, and cysteine
-	SMD_3004	2,69	2,72	EC_1.1.1.215	Carbohydrates	Monosaccharides
-	SMD_3004	2,69	2,72	EC_1.1.1.26, EC_1.1.1.79, E	Carbohydrates	Central carbohydrate metabolism
-	SMD_3004	2,69	2,72	EC_1.1.1.81	Carbohydrates	Organic acids
aroC	SMD_3005	2,58	3,18	EC_4.2.3.5	Amino Acids and Derivatives	Aromatic amino acids and derivatives
psd	SMD_3008	2,48	2,81	EC_4.1.1.65	Fatty Acids, Lipids, and Isoprenoids	Phospholipids
mltD	SMD_3010	2,53	2,80	EC_3.2.1.-	Cell Wall and Capsule	Cell Wall and Capsule
dcd	SMD_3025	3,05	3,16	EC_3.5.4.13	Nucleosides and Nucleotides	Pyrimidines
slyD	SMD_3040	2,44	2,43	EC_5.2.1.8	Potassium metabolism	Potassium metabolism
slyD	SMD_3040	2,44	2,43	EC_5.2.1.8	Protein Metabolism	Protein folding
slyD	SMD_3040	2,44	2,43	EC_5.2.1.8	Protein Metabolism	Protein processing and modification
phoA	SMD_3044	2,31	2,51	EC_3.1.3.1	Phosphorus Metabolism	Phosphorus Metabolism

xseB	SMD_3047	2,98	4,38	EC_3.1.11.6	DNA Metabolism	DNA repair
ispA	SMD_3048	2,98	3,67	EC_2.5.1.1, EC_2.5.1.10	Fatty Acids, Lipids, and Isoprenoids	Isoprenoids
rnG	SMD_3054	2,66	3,63	EC_3.1.4.-	RNA Metabolism	RNA processing and modification
nadD	SMD_3063	1,80	2,57	EC_2.7.7.18	Cofactors, Vitamins, Prosthetic Groups, Pigments	NAD and NADP
leuS	SMD_3066	2,86	3,08	EC_6.1.1.4	Protein Metabolism	Protein biosynthesis
metG	SMD_3080	2,47	2,83	EC_6.1.1.10	Miscellaneous	Plant-Prokaryote DOE project
metG	SMD_3080	2,47	2,83	EC_6.1.1.10	Protein Metabolism	Protein biosynthesis
-	SMD_3081	3,02	2,66	EC_3.1.3.3	Amino Acids and Derivatives	Alanine, serine, and glycine
vdh	SMD_3104	3,24	2,75	EC_1.4.1.9	Amino Acids and Derivatives	Branched-chain amino acids
-	SMD_3106	2,94	2,51	EC_2.3.1.16	Amino Acids and Derivatives	Branched-chain amino acids
-	SMD_3106	2,94	2,51	EC_2.3.1.16, EC_2.3.1.9	Carbohydrates	One-carbon Metabolism
-	SMD_3106	2,94	2,51	EC_2.3.1.9	Carbohydrates	Fermentation
-	SMD_3106	2,94	2,51	EC_2.3.1.16	Cofactors, Vitamins, Prosthetic Groups, Pigments	Biotin
-	SMD_3106	2,94	2,51	EC_2.3.1.16, EC_2.3.1.9	Fatty Acids, Lipids, and Isoprenoids	Fatty Acids, Lipids, and Isoprenoids
-	SMD_3106	2,94	2,51	EC_2.3.1.9	Fatty Acids, Lipids, and Isoprenoids	Isoprenoids
mtaP	SMD_3117	2,88	2,63	EC_2.4.2.28	Amino Acids and Derivatives	Lysine, threonine, methionine, and cysteine
hpt	SMD_3118	2,40	2,51	EC_2.4.2.8	Cofactors, Vitamins, Prosthetic Groups, Pigments	Folate and pterines
hpt	SMD_3118	2,40	2,51	EC_2.4.2.8	Nucleosides and Nucleotides	Purines
rumA	SMD_3122	2,91	3,47	EC_2.1.1.-	Regulation and Cell signaling	Regulation of virulence
rumA	SMD_3122	2,91	3,47	EC_2.1.1.-	RNA Metabolism	RNA processing and modification
rnc	SMD_3126	2,24	2,13	EC_3.1.26.3	RNA Metabolism	RNA processing and modification
lepB	SMD_3128	2,07	2,61	EC_3.4.21.89	Protein Metabolism	Protein processing and modification
-	SMD_3133	2,96	3,51	EC_1.1.1.35, EC_4.2.1.17	Amino Acids and Derivatives	Branched-chain amino acids
-	SMD_3133	2,96	3,51	EC_1.1.1.35, EC_5.1.2.3	Carbohydrates	Fermentation
-	SMD_3133	2,96	3,51	EC_1.1.1.35	Fatty Acids, Lipids, and Isoprenoids	Fatty acids
-	SMD_3133	2,96	3,51	EC_4.2.1.17, EC_5.1.2.3	Fatty Acids, Lipids, and Isoprenoids	Fatty Acids, Lipids, and Isoprenoids
gcvP	SMD_3153	2,73	2,47	EC_1.4.4.2	Amino Acids and Derivatives	Alanine, serine, and glycine
purA	SMD_3164	3,36	3,56	EC_6.3.4.4	Nucleosides and Nucleotides	Purines
pbpC	SMD_3174	2,13	2,51	EC_2.4.2.-	Cell Wall and Capsule	Cell Wall and Capsule
prpD	SMD_3176	3,32	3,30	EC_4.2.1.79	Carbohydrates	Organic acids
acnA	SMD_3180	2,13	2,26	EC_4.2.1.79	Carbohydrates	Organic acids
prpC	SMD_3181	2,62	2,60	EC_2.3.3.5	Carbohydrates	Organic acids
prpB	SMD_3182	2,47	1,88	EC_4.1.3.30	Carbohydrates	Organic acids
aroB	SMD_3189	2,81	3,23	EC_4.2.3.4	Amino Acids and Derivatives	Aromatic amino acids and derivatives
aroB	SMD_3189	2,81	3,23	EC_4.2.3.4	Membrane Transport	Protein and nucleoprotein secretion system, Type IV
hemE	SMD_3191	3,20	3,56	EC_4.1.1.37	Cofactors, Vitamins, Prosthetic Groups, Pigments	Tetrapyrroles
-	SMD_3220	3,44	4,13	EC_3.5.2.6	Virulence, Disease and Defense	Resistance to antibiotics and toxic compounds

gcvT	SMD_3225	2,47	2,70	EC_2.1.2.10	Amino Acids and Derivatives	Alanine, serine, and glycine
nudE	SMD_3230	2,32	3,10	EC_3.6.1.-	Nucleosides and Nucleotides	Detoxification
bioA	SMD_3231	2,88	2,74	EC_2.6.1.62	Cofactors, Vitamins, Prosthetic Groups, Pigments	Biotin
rsmE	SMD_3232	2,57	2,76	EC_2.1.1.-	RNA Metabolism	RNA processing and modification
rsmE	SMD_3232	2,57	2,76	EC_2.1.1.-	Stress Response	Oxidative stress
rsmE	SMD_3232	2,57	2,76	EC_2.1.1.-	Stress Response	Heat shock
gshB	SMD_3242	3,80	4,67	EC_6.3.2.3	Stress Response	Oxidative stress
gshB	SMD_3242	3,80	4,67	EC_6.3.2.3	Stress Response	Heat shock
mrcB	SMD_3248	2,89	3,12	EC_2.4.1.129	Cell Wall and Capsule	Cell Wall and Capsule
mrcB	SMD_3248	2,89	3,12	EC_2.4.1.129	Membrane Transport	Protein and nucleoprotein secretion system, Type IV
relA	SMD_3251	3,03	2,96	EC_2.7.6.5	Regulation and Cell signaling	Regulation of virulence
-	SMD_3254	3,59	3,98	EC_1.16.3.1	Stress Response	Oxidative stress
ruvC	SMD_3318	2,68	2,39	EC_3.1.22.4	DNA Metabolism	DNA recombination
aspS	SMD_3323	2,89	3,15	EC_6.1.1.12	Protein Metabolism	Protein biosynthesis
otsA	SMD_3346	2,87	3,46	EC_2.4.1.15	Carbohydrates	Di- and oligosaccharides
rluD	SMD_3350	2,52	2,50	EC_4.2.1.70	Protein Metabolism	Protein biosynthesis
rluD	SMD_3350	2,52	2,50	EC_4.2.1.70	RNA Metabolism	RNA processing and modification
nadE	SMD_3353	2,85	3,02	EC_6.3.1.5	Cofactors, Vitamins, Prosthetic Groups, Pigments	NAD and NADP
sucD	SMD_3354	1,94	1,94	EC_6.2.1.5	Carbohydrates	Central carbohydrate metabolism
sucD	SMD_3354	1,94	1,94	EC_6.2.1.5	Carbohydrates	One-carbon Metabolism
sucC	SMD_3355	1,95	1,92	EC_6.2.1.5	Carbohydrates	Central carbohydrate metabolism
sucC	SMD_3355	1,95	1,92	EC_6.2.1.5	Carbohydrates	One-carbon Metabolism
coaE	SMD_3362	2,77	4,14	EC_2.7.1.24	Cofactors, Vitamins, Prosthetic Groups, Pigments	Coenzyme A
trmB	SMD_3379	2,37	3,37	EC_2.1.1.33	RNA Metabolism	RNA processing and modification
alf1	SMD_3400	2,55	2,44	EC_4.1.2.13	Carbohydrates	Central carbohydrate metabolism
pykA	SMD_3401	3,12	3,14	EC_2.7.1.40	Carbohydrates	Central carbohydrate metabolism
pykA	SMD_3401	3,12	3,14	EC_2.7.1.40	Carbohydrates	Organic acids
pgk	SMD_3403	2,33	2,92	EC_2.7.2.3	Carbohydrates	Central carbohydrate metabolism
gap	SMD_3406	2,52	2,96	EC_1.2.1.12	Carbohydrates	Central carbohydrate metabolism
gap	SMD_3406	2,52	2,96	EC_1.2.1.12	Cofactors, Vitamins, Prosthetic Groups, Pigments	Pyridoxine
gap	SMD_3406	2,52	2,96	EC_1.2.1.12	Stress Response	Oxidative stress
tktA	SMD_3416	2,36	2,52	EC_2.2.1.1	Carbohydrates	Central carbohydrate metabolism
mrcA	SMD_3429	3,08	3,81	EC_2.4.1.129	Cell Wall and Capsule	Cell Wall and Capsule
mrcA	SMD_3429	3,08	3,81	EC_2.4.1.129	Membrane Transport	Protein and nucleoprotein secretion system, Type IV
gltA	SMD_3438	2,18	2,10	EC_2.3.3.1	Carbohydrates	Central carbohydrate metabolism
gltA	SMD_3438	2,18	2,10	EC_2.3.3.1	Carbohydrates	One-carbon Metabolism
iunH	SMD_3440	2,74	2,90	EC_3.2.2.1	Nucleosides and Nucleotides	Purines

iunH	SMD_3440	2,74	2,90	EC_3.2.2.1	RNA Metabolism	RNA processing and modification
rpoZ	SMD_3444	3,58	4,22	EC_2.7.7.6	RNA Metabolism	Transcription
gmk	SMD_3445	3,37	3,25	EC_2.7.4.8	Nucleosides and Nucleotides	Purines
fdnG	SMD_3446	5,60	5,59	EC_1.2.1.2	Respiration	Electron donating reactions
fdnG	SMD_3446	5,60	5,59	EC_1.2.1.2	Respiration	Respiration
selA	SMD_3450	4,09	3,53	EC_2.9.1.1	Protein Metabolism	Selenoproteins
selD	SMD_3453	4,54	5,20	EC_2.7.9.3	Protein Metabolism	Selenoproteins
selD	SMD_3453	4,54	5,20	EC_2.7.9.3	RNA Metabolism	RNA processing and modification
rph	SMD_3455	2,52	3,10	EC_2.7.7.56	RNA Metabolism	RNA processing and modification
rph	SMD_3455	2,52	3,10	EC_2.7.7.56	Stress Response	Heat shock
-	SMD_3457	2,89	2,89	EC_3.6.1.15	Nucleosides and Nucleotides	Detoxification
-	SMD_3457	2,89	2,89	EC_3.6.1.15	Stress Response	Heat shock
pepQ	SMD_3461	2,96	2,74	EC_3.4.13.9	Protein Metabolism	Protein degradation
rpiA	SMD_3469	2,14	2,22	EC_5.3.1.6	Carbohydrates	Central carbohydrate metabolism
rpiA	SMD_3469	2,14	2,22	EC_5.3.1.6	Carbohydrates	Monosaccharides
thiE	SMD_3473	2,72	2,63	EC_2.5.1.3	Cofactors, Vitamins, Prosthetic Groups, Pigments	Cofactors, Vitamins, Prosthetic Groups, Pigments
thiE	SMD_3473	2,72	2,63	EC_2.5.1.3	Cofactors, Vitamins, Prosthetic Groups, Pigments	Folate and pterines
hemL	SMD_3474	2,90	2,92	EC_5.4.3.8	Cofactors, Vitamins, Prosthetic Groups, Pigments	Tetrapyrroles
mpl	SMD_3488	3,26	3,18	EC_6.3.2.-	Cell Wall and Capsule	Cell Wall and Capsule
adk	SMD_3489	2,47	2,43	EC_2.7.4.3	Nucleosides and Nucleotides	Purines
pfk	SMD_3490	3,77	3,17	EC_2.7.1.11	Carbohydrates	Central carbohydrate metabolism
pfk	SMD_3490	3,77	3,17	EC_2.7.1.11	Carbohydrates	Aminosugars
ppa	SMD_3505	2,49	2,30	EC_3.6.1.1	Phosphorus Metabolism	Phosphorus Metabolism
-	SMD_3514	3,65	3,94	EC_2.3.2.2	Stress Response	Oxidative stress
-	SMD_3516	2,62	2,52	EC_1.1.1.86	Amino Acids and Derivatives	Branched-chain amino acids
-	SMD_3516	2,62	2,52	EC_1.1.1.86	Cofactors, Vitamins, Prosthetic Groups, Pigments	Coenzyme A
leuA	SMD_3520	3,50	2,46	EC_2.3.3.13	Amino Acids and Derivatives	Branched-chain amino acids
leuC	SMD_3522	2,59	2,89	EC_4.2.1.33	Amino Acids and Derivatives	Branched-chain amino acids
leuD	SMD_3523	1,77	1,43	EC_4.2.1.33	Amino Acids and Derivatives	Branched-chain amino acids
leuB	SMD_3524	2,21	2,40	EC_1.1.1.85	Amino Acids and Derivatives	Branched-chain amino acids
pcm2	SMD_3530	2,59	3,15	EC_2.1.1.77	Membrane Transport	Membrane Transport
pcm2	SMD_3530	2,59	3,15	EC_2.1.1.77	Protein Metabolism	Protein processing and modification
pcm2	SMD_3530	2,59	3,15	EC_2.1.1.77	Secondary Metabolism	Secondary Metabolism
kdtA	SMD_3533	3,15	4,96	EC_2.-.-.-	Cell Wall and Capsule	Gram-Negative cell wall components
htrB	SMD_3534	3,69	3,72	EC_2.3.1.-	Cell Wall and Capsule	Gram-Negative cell wall components
maeB	SMD_3541	2,55	2,70	EC_1.1.1.40	Carbohydrates	Central carbohydrate metabolism
fabG3	SMD_3551	1,63	2,31	EC_1.1.1.36	Carbohydrates	One-carbon Metabolism

fabG3	SMD_3551	1,63	2,31	EC_1.1.1.36	Carbohydrates	Fermentation
fabG3	SMD_3551	1,63	2,31	EC_1.1.1.36	Fatty Acids, Lipids, and Isoprenoids	Fatty Acids, Lipids, and Isoprenoids
-	SMD_3555	3,62	4,09	EC_2.6.1.1	Amino Acids and Derivatives	Lysine, threonine, methionine, and cysteine
amn	SMD_3561	3,19	4,05	EC_3.2.2.4	Nucleosides and Nucleotides	Purines
-	SMD_3575	2,94	2,65	EC_3.1.2.12	Stress Response	Detoxification
adhC	SMD_3577	3,10	2,60	EC_1.1.1.284	Stress Response	Detoxification
-	SMD_3586	2,45	2,06	EC_1.1.1.1	Carbohydrates	Fermentation
-	SMD_3586	2,45	2,06	EC_1.1.1.1	Cofactors, Vitamins, Prosthetic Groups, Pigments	Folate and pterines
-	SMD_3586	2,45	2,06	EC_1.1.1.1	Fatty Acids, Lipids, and Isoprenoids	Phospholipids
-	SMD_3587	2,89	4,05	EC_6.3.2.2	Stress Response	Oxidative stress
-	SMD_3603	3,59	4,00	EC_3.2.1.-	Cell Wall and Capsule	Cell Wall and Capsule
nagA	SMD_3612	2,15	2,20	EC_3.5.1.25	Carbohydrates	Aminosugars
nagA	SMD_3612	2,15	2,20	EC_3.5.1.25	Cell Wall and Capsule	Capsular and extracellular polysacchrides
-	SMD_3613	2,62	2,20	EC_3.5.99.6	Carbohydrates	Aminosugars
nahB	SMD_3618	3,45	3,42	EC_3.2.1.52	Carbohydrates	Aminosugars
-	SMD_3622	3,64	4,06	EC_2.7.6.3	Cofactors, Vitamins, Prosthetic Groups, Pigments	Folate and pterines
-	SMD_3630	2,62	2,74	EC_1.1.1.44	Carbohydrates	Central carbohydrate metabolism
-	SMD_3630	2,62	2,74	EC_1.1.1.44	Carbohydrates	Monosaccharides
dacC	SMD_3638	2,39	2,30	EC_3.4.16.4	Cell Wall and Capsule	Cell Wall and Capsule
dacC	SMD_3638	2,39	2,30	EC_3.4.16.4	Protein Metabolism	Protein degradation
mltB	SMD_3640	3,42	4,36	EC_3.2.1.-	Cell Wall and Capsule	Cell Wall and Capsule
ubiE	SMD_3651	3,67	3,38	EC_2.1.1.-	Cofactors, Vitamins, Prosthetic Groups, Pigments	Quinone cofactors
codA	SMD_3652	3,19	2,83	EC_3.5.4.1	Amino Acids and Derivatives	Amino Acids and Derivatives
codA	SMD_3652	3,19	2,83	EC_3.5.4.1	Nucleosides and Nucleotides	Pyrimidines
hslV	SMD_3662	2,68	2,96	EC_3.4.25.-	Protein Metabolism	Protein degradation
dapF	SMD_3665	2,82	3,12	EC_5.1.1.7	Amino Acids and Derivatives	Lysine, threonine, methionine, and cysteine
-	SMD_3669	3,26	4,11	EC_2.6.1.1	Amino Acids and Derivatives	Lysine, threonine, methionine, and cysteine
prlC	SMD_3679	2,31	2,89	EC_3.4.24.70	Protein Metabolism	Protein degradation
-	SMD_3681	2,84	3,42	EC_2.5.1.47	Amino Acids and Derivatives	Lysine, threonine, methionine, and cysteine
-	SMD_3682	2,58	3,21	EC_4.4.1.5	Carbohydrates	Central carbohydrate metabolism
-	SMD_3682	2,58	3,21	EC_4.4.1.5	Stress Response	Oxidative stress
-	SMD_3684	2,47	3,54	EC_1.1.1.205	Nucleosides and Nucleotides	Purines
glmS	SMD_3685	2,74	2,58	EC_2.6.1.16	Cell Wall and Capsule	Capsular and extracellular polysacchrides
glmS	SMD_3685	2,74	2,58	EC_2.6.1.16	Cell Wall and Capsule	Cell Wall and Capsule
glmU	SMD_3694	2,37	3,24	EC_2.3.1.157, EC_2.7.7.23	Cell Wall and Capsule	Capsular and extracellular polysacchrides
glmU	SMD_3694	2,37	3,24	EC_2.3.1.157, EC_2.7.7.23	Cell Wall and Capsule	Cell Wall and Capsule
lpdA	SMD_3708	2,40	2,54	EC_1.8.1.4	Carbohydrates	Central carbohydrate metabolism

DLAT	SMD_3709	2,81	2,64	EC_2.3.1.12	Carbohydrates	Central carbohydrate metabolism
DLAT	SMD_3709	2,81	2,64	EC_2.3.1.12	Cofactors, Vitamins, Prosthetic Groups, Pigments	Folate and pterines
hemC	SMD_3718	4,76	4,89	EC_2.5.1.61	Cofactors, Vitamins, Prosthetic Groups, Pigments	Tetrapyrroles
-	SMD_3722	3,59	4,27	EC_2.4.1.-	Stress Response	Osmotic stress
gst5	SMD_3746	2,97	3,74	EC_2.5.1.18	Stress Response	Oxidative stress
ribA	SMD_3768	3,01	3,37	EC_3.5.4.25	Cofactors, Vitamins, Prosthetic Groups, Pigments	Riboflavin, FMN, FAD
ribA	SMD_3768	3,01	3,37	EC_3.5.4.25	Cofactors, Vitamins, Prosthetic Groups, Pigments	Folate and pterines
def	SMD_3777	3,56	2,68	EC_3.5.1.88	Protein Metabolism	Protein biosynthesis
topA	SMD_3783	2,47	3,06	EC_5.99.1.2	DNA Metabolism	DNA replication
aroG	SMD_3803	4,69	5,29	EC_2.5.1.54	Amino Acids and Derivatives	Aromatic amino acids and derivatives
-	SMD_3817	2,61	3,84	EC_1.8.1.8	Protein Metabolism	Protein folding
-	SMD_3817	2,61	3,84	EC_1.8.1.8	Respiration	Respiration
-	SMD_3826	6,44	5,96	EC_3.4.16.4	Cell Wall and Capsule	Cell Wall and Capsule
-	SMD_3826	6,44	5,96	EC_3.4.16.4	Protein Metabolism	Protein degradation
aroQ	SMD_3832	2,54	3,89	EC_4.2.1.10	Amino Acids and Derivatives	Aromatic amino acids and derivatives
aroQ	SMD_3832	2,54	3,89	EC_4.2.1.10	Metabolism of Aromatic Compounds	Peripheral pathways for catabolism of aromatic compounds
accC	SMD_3835	1,94	2,02	EC_6.3.4.14	Fatty Acids, Lipids, and Isoprenoids	Fatty acids
prmA	SMD_3838	3,20	4,03	EC_2.1.1.-	Protein Metabolism	Protein biosynthesis
prmA	SMD_3838	3,20	4,03	EC_2.1.1.-	Stress Response	Heat shock
cdsA2	SMD_3841	2,28	4,47	EC_2.7.7.41	Fatty Acids, Lipids, and Isoprenoids	Phospholipids
-	SMD_3842	2,72	3,07	EC_2.3.1.51	Fatty Acids, Lipids, and Isoprenoids	Phospholipids
purH	SMD_3847	2,86	3,22	EC_2.1.2.3	Cofactors, Vitamins, Prosthetic Groups, Pigments	Folate and pterines
purH	SMD_3847	2,86	3,22	EC_2.1.2.3, EC_3.5.4.10	Nucleosides and Nucleotides	Purines
icd	SMD_3868	2,89	2,75	EC_1.1.1.42	Carbohydrates	Central carbohydrate metabolism
icd	SMD_3868	2,89	2,75	EC_1.1.1.42	Cofactors, Vitamins, Prosthetic Groups, Pigments	Folate and pterines
aas	SMD_3878	3,48	3,95	EC_2.3.1.51	Fatty Acids, Lipids, and Isoprenoids	Phospholipids
nudC	SMD_3883	2,76	4,34	EC_3.6.1.22	Nucleosides and Nucleotides	Detoxification
nudH	SMD_3892	3,11	3,43	EC_3.6.1.-	Miscellaneous	Miscellaneous
nudH	SMD_3892	3,11	3,43	EC_3.6.1.-	Nucleosides and Nucleotides	Detoxification
-	SMD_3897	3,32	3,20	EC_1.14.13.-	Cofactors, Vitamins, Prosthetic Groups, Pigments	Quinone cofactors
trpC	SMD_3903	3,25	3,39	EC_4.1.1.48	Amino Acids and Derivatives	Aromatic amino acids and derivatives
trpD	SMD_3904	2,95	2,66	EC_2.4.2.18	Amino Acids and Derivatives	Aromatic amino acids and derivatives
trpD	SMD_3904	2,95	2,66	EC_2.4.2.18	Secondary Metabolism	Plant Hormones
pabA	SMD_3905	2,98	3,45	EC_2.6.1.85, EC_4.1.3.27	Amino Acids and Derivatives	Aromatic amino acids and derivatives
pabA	SMD_3905	2,98	3,45	EC_2.6.1.85	Cofactors, Vitamins, Prosthetic Groups, Pigments	Folate and pterines
trpE	SMD_3907	4,46	6,54	EC_4.1.3.27	Amino Acids and Derivatives	Aromatic amino acids and derivatives
rpe	SMD_3910	2,46	1,91	EC_5.1.3.1	Carbohydrates	Central carbohydrate metabolism

purC	SMD_3912	4,62	4,58	EC_6.3.2.6	Nucleosides and Nucleotides	Purines
hmgA	SMD_3923	2,13	2,56	EC_1.13.11.5	Cofactors, Vitamins, Prosthetic Groups, Pigments	Folate and pterines
hmgA	SMD_3923	2,13	2,56	EC_1.13.11.5	Metabolism of Aromatic Compounds	Metabolism of central aromatic intermediates
-	SMD_3924	2,72	3,27	EC_1.13.11.27	Amino Acids and Derivatives	Aromatic amino acids and derivatives
-	SMD_3924	2,72	3,27	EC_1.13.11.27	Cofactors, Vitamins, Prosthetic Groups, Pigments	Folate and pterines
-	SMD_3924	2,72	3,27	EC_1.13.11.27	Metabolism of Aromatic Compounds	Metabolism of central aromatic intermediates
-	SMD_3929	2,81	4,76	EC_1.13.11.11	Amino Acids and Derivatives	Aromatic amino acids and derivatives
-	SMD_3929	2,81	4,76	EC_1.13.11.11	Cofactors, Vitamins, Prosthetic Groups, Pigments	NAD and NADP
pdhA	SMD_3930	2,31	2,50	EC_1.2.4.4	Amino Acids and Derivatives	Branched-chain amino acids
pdhA	SMD_3930	2,31	2,50	EC_1.2.4.4	Carbohydrates	Central carbohydrate metabolism
pdhB	SMD_3931	2,03	2,47	EC_1.2.4.4	Amino Acids and Derivatives	Branched-chain amino acids
pdhB	SMD_3931	2,03	2,47	EC_1.2.4.4	Carbohydrates	Central carbohydrate metabolism
-	SMD_3933	2,14	1,93	EC_2.3.1.168	Amino Acids and Derivatives	Branched-chain amino acids
-	SMD_3933	2,14	1,93	EC_2.3.1.168	Carbohydrates	Central carbohydrate metabolism
agaS	SMD_4001	3,55	1,76	EC_5.3.1.-	Carbohydrates	Aminosugars
nagA2	SMD_4005	2,27	3,97	EC_3.5.1.25	Carbohydrates	Aminosugars
nagA2	SMD_4005	2,27	3,97	EC_3.5.1.25	Cell Wall and Capsule	Capsular and extracellular polysacchrides
bioF	SMD_4013	3,06	3,24	EC_2.3.1.47	Cofactors, Vitamins, Prosthetic Groups, Pigments	Biotin
-	SMD_4018	4,06	3,88	EC_4.3.1.19	Amino Acids and Derivatives	Lysine, threonine, methionine, and cysteine
-	SMD_4018	4,06	3,88	EC_4.3.1.19	Amino Acids and Derivatives	Branched-chain amino acids
-	SMD_4018	4,06	3,88	EC_4.3.1.19	Amino Acids and Derivatives	Alanine, serine, and glycine
-	SMD_4035	2,89	2,78	EC_6.3.1.8	Stress Response	Oxidative stress
ilvD	SMD_4038	3,36	2,63	EC_4.2.1.9	Amino Acids and Derivatives	Branched-chain amino acids
aroE	SMD_4041	3,72	2,95	EC_1.1.1.25	Amino Acids and Derivatives	Aromatic amino acids and derivatives
hemB	SMD_4044	2,72	2,20	EC_4.2.1.24	Cofactors, Vitamins, Prosthetic Groups, Pigments	Tetrapyrroles
hemB	SMD_4044	2,72	2,20	EC_4.2.1.24	Regulation and Cell signaling	Regulation and Cell signaling
gst6	SMD_4051	3,97	4,04	EC_2.5.1.18	Stress Response	Oxidative stress
-	SMD_4077	7,60	8,45	EC_1.8.1.9	Nucleosides and Nucleotides	Pyrimidines
-	SMD_4077	7,60	8,45	EC_1.8.1.9	Sulfur Metabolism	Sulfur Metabolism
fabF2	SMD_4090	2,11	2,20	EC_2.3.1.41	Fatty Acids, Lipids, and Isoprenoids	Fatty acids
fabG5	SMD_4091	2,15	2,25	EC_1.1.1.100	Fatty Acids, Lipids, and Isoprenoids	Fatty acids
-	SMD_4092	1,31	2,33	EC_4.2.1.59	Fatty Acids, Lipids, and Isoprenoids	Fatty acids
-	SMD_4093	2,34	2,29	EC_2.3.1.41	Fatty Acids, Lipids, and Isoprenoids	Fatty acids
-	SMD_4099	2,15	2,08	EC_4.3.1.3	Amino Acids and Derivatives	Histidine Metabolism
-	SMD_4102	2,94	2,82	EC_4.2.1.-	Fatty Acids, Lipids, and Isoprenoids	Fatty acids
hemF	SMD_4119	3,50	3,56	EC_1.3.3.3	Cofactors, Vitamins, Prosthetic Groups, Pigments	Tetrapyrroles
polA	SMD_4122	2,83	3,28	EC_2.7.7.7	DNA Metabolism	DNA repair

xthA3	SMD_4151	3,26	3,38	EC_3.1.11.2	DNA Metabolism	DNA repair
acsA	SMD_4155	3,48	3,63	EC_6.2.1.1	Carbohydrates	Central carbohydrate metabolism
glyS	SMD_4166	2,54	2,92	EC_6.1.1.14	Protein Metabolism	Protein biosynthesis
glyQ	SMD_4167	3,14	3,56	EC_6.1.1.14	Protein Metabolism	Protein biosynthesis
-	SMD_4169	3,62	4,03	EC_6.3.5.2	Nucleosides and Nucleotides	Purines
-	SMD_4193	4,03	4,30	EC_3.2.1.-	Cell Wall and Capsule	Cell Wall and Capsule
folE	SMD_4202	2,94	3,02	EC_3.5.4.16	Cofactors, Vitamins, Prosthetic Groups, Pigments	Folate and pterines
recD	SMD_4209	2,84	2,80	EC_3.1.11.5	DNA Metabolism	DNA repair
recB	SMD_4210	2,31	2,39	EC_3.1.11.5	DNA Metabolism	DNA repair
recC	SMD_4211	2,76	3,57	EC_3.1.11.5	DNA Metabolism	DNA repair
-	SMD_4229	2,79	3,94	EC_3.4.13.19	Protein Metabolism	Protein degradation
glpQ	SMD_4234	4,88	6,81	EC_3.1.4.46	Carbohydrates	Sugar alcohols

Table S5. Statistical analysis of RNA half-lives of RNAs grouped in functional categories

Statistical test	Kruskal-Wallis
P value	< 0.0001
Exact or approximate P value?	Gaussian Approximation
P value summary	***
Do the medians vary signif. (P < 0.05)	Yes
Number of groups	13
Kruskal-Wallis statistic	62,52

**Dunn's Multiple Comparison Test**	**Difference in rank sum**	**Significant? P < 0.05?**	**Summary**
Virulence disease and defense vs Stress response	-204,5	Yes	*
Virulence disease and defense vs RNA metabolism	-39,04	No	ns
Virulence disease and defense vs Respiration	68,67	No	ns
Virulence disease and defense vs Regulation and cell signaling	-116,8	No	ns
Virulence disease and defense vs Protein metabolism	-96,14	No	ns
Virulence disease and defense vs Nucleosides and nucleotides	-125,1	No	ns
Virulence disease and defense vs Fatty acids and lipids	-29,47	No	ns
Virulence disease and defense vs DNA metabolism	-92,90	No	ns
Virulence disease and defense vs Cofactors, vitamins, pigments	-140,1	No	ns
Virulence disease and defense vs Cell wall and capsule	-46,68	No	ns
Virulence disease and defense vs Carbohydrates	-30,40	No	ns
Virulence disease and defense vs Amino acids and derivatives	-86,75	No	ns
Stress response vs RNA metabolism	165,4	Yes	*
Stress response vs Respiration	273,1	Yes	***
Stress response vs Regulation and cell signaling	87,70	No	ns
Stress response vs Protein metabolism	108,3	No	ns
Stress response vs Nucleosides and nucleotides	79,34	No	ns
Stress response vs Fatty acids and lipids	175,0	Yes	**
Stress response vs DNA metabolism	111,6	No	ns
Stress response vs Cofactors, vitamins, pigments	64,36	No	ns
Stress response vs Cell wall and capsule	157,8	Yes	*
Stress response vs Carbohydrates	174,1	Yes	***
Stress response vs Amino acids and derivatives	117,7	No	ns

RNA metabolism vs Respiration	107,7	No	ns
RNA metabolism vs Regulation and cell signaling	-77,71	No	ns
RNA metabolism vs Protein metabolism	-57,09	No	ns
RNA metabolism vs Nucleosides and nucleotides	-86,07	No	ns
RNA metabolism vs Fatty acids and lipids	9,575	No	ns
RNA metabolism vs DNA metabolism	-53,86	No	ns
RNA metabolism vs Cofactors, vitamins, pigments	-101,0	No	ns
RNA metabolism vs Cell wall and capsule	-7,633	No	ns
RNA metabolism vs Carbohydrates	8,643	No	ns
RNA metabolism vs Amino acids and derivatives	-47,71	No	ns
Respiration vs Regulation and cell signaling	-185,4	No	ns
Respiration vs Protein metabolism	-164,8	Yes	**
Respiration vs Nucleosides and nucleotides	-193,8	Yes	***
Respiration vs Fatty acids and lipids	-98,14	No	ns
Respiration vs DNA metabolism	-161,6	No	ns
Respiration vs Cofactors, vitamins, pigments	-208,8	Yes	***
Respiration vs Cell wall and capsule	-115,3	No	ns
Respiration vs Carbohydrates	-99,07	No	ns
Respiration vs Amino acids and derivatives	-155,4	Yes	**
Regulation and cell signaling vs Protein metabolism	20,61	No	ns
Regulation and cell signaling vs Nucleosides and nucleotides	-8,361	No	ns
Regulation and cell signaling vs Fatty acids and lipids	87,28	No	ns
Regulation and cell signaling vs DNA metabolism	23,85	No	ns
Regulation and cell signaling vs Cofactors, vitamins, pigments	-23,34	No	ns
Regulation and cell signaling vs Cell wall and capsule	70,07	No	ns
Regulation and cell signaling vs Carbohydrates	86,35	No	ns
Regulation and cell signaling vs Amino acids and derivatives	30,00	No	ns
Protein metabolism vs Nucleosides and nucleotides	-28,97	No	ns
Protein metabolism vs Fatty acids and lipids	66,67	No	ns
Protein metabolism vs DNA metabolism	3,239	No	ns
Protein metabolism vs Cofactors, vitamins, pigments	-43,95	No	ns
Protein metabolism vs Cell wall and capsule	49,46	No	ns
Protein metabolism vs Carbohydrates	65,74	No	ns

Protein metabolism vs Amino acids and derivatives	9,389	No	ns
Nucleosides and nucleotides vs Fatty acids and lipids	95,64	No	ns
Nucleosides and nucleotides vs DNA metabolism	32,21	No	ns
Nucleosides and nucleotides vs Cofactors, vitamins, pigments	-14,98	No	ns
Nucleosides and nucleotides vs Cell wall and capsule	78,44	No	ns
Nucleosides and nucleotides vs Carbohydrates	94,71	No	ns
Nucleosides and nucleotides vs Amino acids and derivatives	38,36	No	ns
Fatty acids and lipids vs DNA metabolism	-63,43	No	ns
Fatty acids and lipids vs Cofactors, vitamins, pigments	-110,6	No	ns
Fatty acids and lipids vs Cell wall and capsule	-17,21	No	ns
Fatty acids and lipids vs Carbohydrates	-0,9322	No	ns
Fatty acids and lipids vs Amino acids and derivatives	-57,28	No	ns
DNA metabolism vs Cofactors, vitamins, pigments	-47,19	No	ns
DNA metabolism vs Cell wall and capsule	46,22	No	ns
DNA metabolism vs Carbohydrates	62,50	No	ns
DNA metabolism vs Amino acids and derivatives	6,150	No	ns
Cofactors, vitamins, pigments vs Cell wall and capsule	93,41	No	ns
Cofactors, vitamins, pigments vs Carbohydrates	109,7	Yes	**
Cofactors, vitamins, pigments vs Amino acids and derivatives	53,34	No	ns
Cell wall and capsule vs Carbohydrates	16,28	No	ns
Cell wall and capsule vs Amino acids and derivatives	-40,07	No	ns
Carbohydrates vs Amino acids and derivatives	-56,35	No	ns

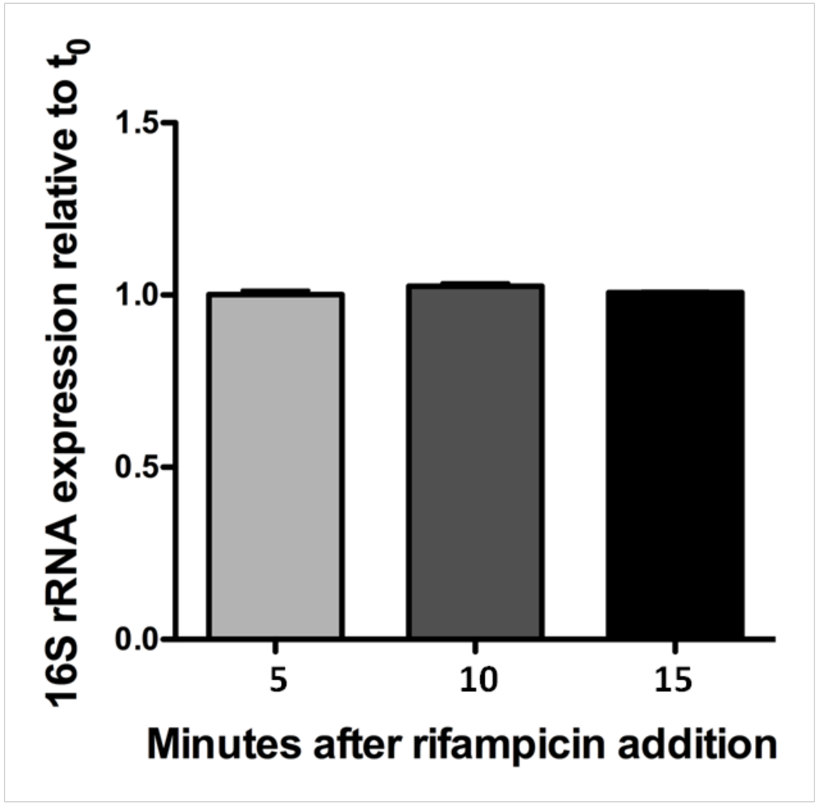

Figure S1. Expression of 16S rRNA at 5, 10 and 15 minutes after transcriptional arrest by rifampicin, relative to 16S rRNA expression at t(0) (time point of rifampicin addition). 16S rRNA expression was measured in *S. maltophilia* D457 by RT-qPCR in 2 replicate RNA decay series of rifampicin addition, using a constant amount of RNA.

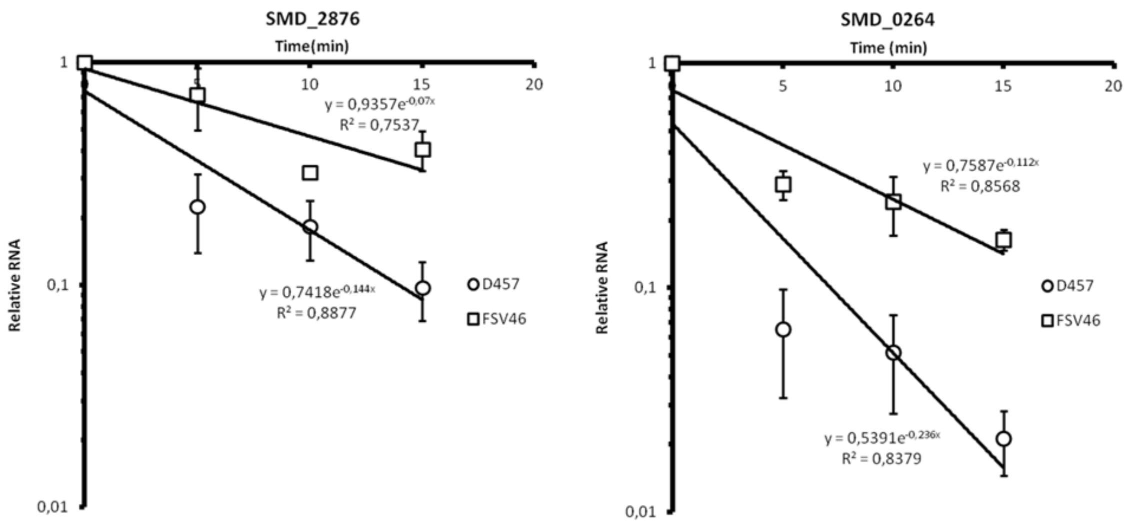

Gene	Strain	Expression (RPKM)	mRNA Half-life by RNA-Seq (min)	mRNA Half-life by RT-qPCR (min)
SMD_0264	D457	135	2,72	2,94
	ALB001	111	6,02	6,19
	**Half-life ratio ALB001/D457**		**2,21**	**2,11**
SMD_2876	D457	298	4,42	4,82
	ALB001	284	8,79	9,92
	**Half-life ratio ALB001/D457**		**1,99**	**2,06**

Figure S2. RT-qPCR verifies that SMD_2876 and SMD_0264 RNAs have two-fold half-life in *rng*-deficient mutant in comparison to the wild-type strain. The relative amount of SMD_2876 and SMD_0264 mRNAs was measured in *S. maltophilia* D457 and ALB001 by RT-qPCR in 2 replicate RNA decay series of rifampicin addition. Values indicate the decrease of the mRNA levels observed as a function of time after rifampicin addition (time 0); the standard error is shown. Half-life values calculated by both procedures (RNA-Seq and RT-qPCR) are shown.
